# Supplementary material for: Fine-tuning the efficiency of para-hydrogen-induced hyperpolarization by rational N-heterocyclic carbene design
Source: Nat Commun. 2018 Oct 12;9:4251. doi: 10.1038/s41467-018-06766-1 (PMC6185983; doi:10.1038/s41467-018-06766-1)
Supplement: Supplementary file 1 — Supplementary Information [file 41467_2018_6766_MOESM1_ESM.pdf]

**Supplementary Information**

# Fine-tuning the efficiency of *para*-hydrogen-induced hyperpolarization by rational *N*-heterocyclic carbene design

*Rayner et al.*

## Supplementary Methods

## Synthetic procedures and characterisation data

Water is distilled water. Brine refers to a saturated aqueous solution of NaCl. THF was freshly distilled from sodium and benzophenone ketyl or dried using a Grubbs solvent purification system. Petrol refers to the fraction of petroleum ether boiling in the range 40-60 °C. All reactions were carried out under $O_2$-free Ar or $N_2$ using oven-dried and/or flame-dried glassware.

Flash column chromatography was carried out using Fluka Chemie GmbH silica (220-440 mesh). Thin layer chromatography was carried out using Merck $F_{254}$ aluminium-backed silica plates. $^1H$ (400 MHz) and $^{13}C$ (100.6 MHz) NMR spectra were recorded on a Bruker-400 instrument with an internal deuterium lock. Chemical shifts are quoted as parts per million and referenced to $CHCl_3$ ($\delta_H$ 7.27), $(CH_3)_2SO$ ($\delta_H$ 2.54), $CDCl_3$ ($\delta_C$ 77.0) or $(CD_3)_2SO$ ($\delta_C$ 40.45). $^{13}C$ NMR spectra were recorded with broadband proton decoupling. $^{13}C$ NMR spectra were assigned using DEPT experiments where necessary. Coupling constants ($J$) are quoted in Hertz. Electrospray high and low resolution mass spectra were recorded on a Bruker Daltronics microOTOF spectrometer.

All compounds were purchased from Sigma-Aldrich, Fluorochem or Alfa-Aesar and used as supplied unless otherwise stated. The following compounds were synthesised according to literature procedures; methyl-4,6-$d_2$-nicotinate,[1] [IrCl(COD)(IMes)] (**1**),[2] [IrCl(COD)($d_{22}$-IMes)] (***d*₂₂-1**),[1] [IrCl(COD)(IPr)] (**5**),[3] $d_{22}$-IMes.HCl.[4] 1,3-bis-(2,6-diethylphenyl)imidazolium chloride (**S5**),[5] methyl-2,4,5,6-$d_4$-nicotinate,[1] 4,5-bis(phenyl-$d_5$)-3,6-$d_2$-pyridazine.[6]

### General Procedure A – Aniline/glyoxal condensation

Glyoxal (1.0 eq.) and formic acid (2 drops) were added sequentially to a stirred solution of the aniline (2.0 eq.) in MeOH or EtOH at rt. The resulting solution was stirred at rt for 16 h during which time a yellow precipitate formed. The precipitate was filtered, washed with MeOH or EtOH and dried under vacuum to give the ethylenediimine.

### General Procedure B – Imidazolium formation with paraformaldehyde

A solution of paraformaldehyde (1.1 eq.) in 4 M $HCl_{(dioxane)}$ (1.5 eq.) was added dropwise to a stirred solution of the ethylenediimine (1.0 eq.) in EtOAc at rt under $N_2$. The resulting solution was stirred at rt for 16 h during which time an off white precipitate formed. Then, the precipitate was filtered, washed with EtOAc and dried under vacuum to give the imidazolium chloride.

### General Procedure C – Catalyst formation

$KO^tBu$ (2.4 eq.) was added to a stirred solution of the carbene (2.2 eq.) in THF at rt under $N_2$. The resulting suspension was stirred at rt for 30 min. Then, a solution of $[Ir(COD)Cl]_2$ (1.0

eq.) was added and the resulting solution was stirred at rt for 2 h. The solvent was removed under reduced pressure to give the crude product. Purification by flash column chromatography on silica with $CH_2Cl_2$ gave the complex.

**General Procedure D – Formamidine formation**

Triethylorthoformate (1.0 eq.) and the aniline (2.0 eq.) were heated at 160 °C for 3 h. The resulting solution was cooled to rt and hexane was added and the resulting suspension was stirred at rt for 1 h. The precipitate was filtered and washed with hexane to give the formamidine.

**General Procedure E – Formamidine Alkylation**

Sodium hydride (60% dispersion in mineral oil, 1.2 eq.) was added to a stirred solution of the formamidine (1.0 eq.) in DMF at 0 °C. The resulting solution was stirred at 0 °C for 30 min. Then, bromoacetaldehyde diethyl acetal (2.0 eq.) was added and the resulting solution was heated at 80 °C for 2 h. After cooling to rt, a saturated solution of $NH_4Cl_{(aq)}$ was added. The resulting solution was extracted with EtOAc (3 x 30 mL) and the combined organic layers were dried ($MgSO_4$) and concentrated under reduced pressure to give the crude product. Purification by flash column chromatography on silica with 95:5 hexane-EtOAc gave the product.

**General Procedure F – Imidazolium formation from the diethyl acetal**

37% $HCl_{(aq)}$ (1.5 eq.) was added to a stirred solution of the diethyl acetal (1.0 eq.) and acetic anhydride (3.0 eq.) in toluene at rt. The resulting solution was heated to 110 °C for 16 h. After cooling to rt, the solvent was removed under reduced pressure to give the crude product. Precipitation from MeOH-$Et_2O$ gave the carbene salt.

## Synthesis of 2

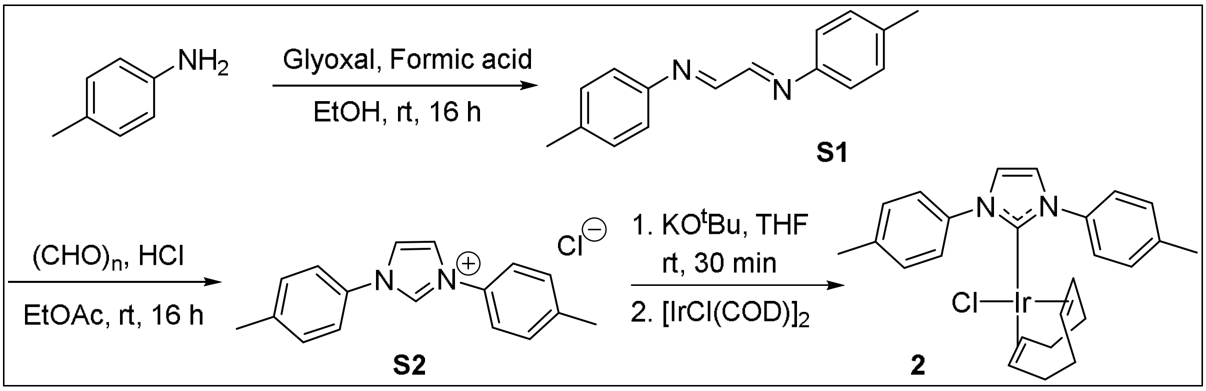


Supplementary Figure 1: Synthesis of **2**.

### 4-Methyl-N-[2-[(4-methylphenyl)imino]ethylidine]aniline S1

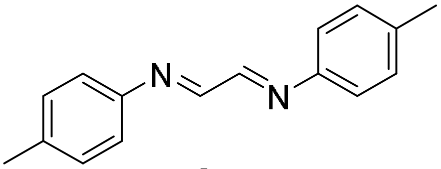

**S1**

Using General Procedure A, *p*-toluidine (2.35 g, 22.0 mmol, 2.0 eq.), glyoxal (1.28 mL of a 40% solution in $H_2O$, 11.0 mmol, 1.0 eq.), formic acid (2 drops) in MeOH (25 mL) gave ethylenediimine **S1** (2.05 g, 79%) as a yellow powder,**$^1$H NMR** (500 MHz, $CDCl_3$) $\delta$ 8.44 (s, 2H), 7.26 (br. s, 8H), 2.41 (s, 6H); **$^{13}$C NMR** (126 MHz, $CDCl_3$) $\delta$ 159.1 (s), 147.6 (s), 138.1 (s), 130.0 (s), 121.3 (s), 21.2 (s); **MS** (ESI) m/z 237 [(M + H)$^+$, 100]; **HRMS** m/z calculated for $C_{16}H_{17}N_2$ (M + H)$^+$ 237.1386, found 237.1383 (+1.1 ppm error).

### 1,3-Bis(4-methylphenyl)imidazolium chloride S2

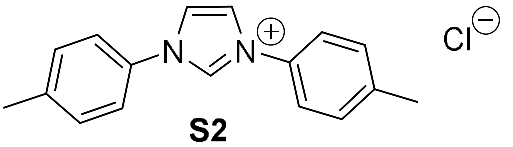

**S2**

Using General Procedure B, ethylenediimine **S3** (520 mg, 2.20 mmol, 1.0 eq), paraformaldehyde (74 mg, 2.42 mmol, 1.1 eq.), HCl (0.83 mL of a 4 M solution in 1,4-dioxane, 3.30 mmol, 1.5 eq.) in EtOAc (20 mL) gave carbene salt **S2** (419 mg, 67%) as an off-white powder,**$^1$H NMR** (400 MHz, $CDCl_3$) $\delta$ 11.67 (s, 1H), 7.96 (d, $J$ = 8.0 Hz, 4H), 7.76 (s, 2H), 7.41 (d, $J$ = 8.0 Hz, 4H ), 2.43 (s, 6H); **$^{13}$C NMR** (100.6 MHz, $CD_3OD$) $\delta$ 140.9 (s), 132.6 (s), 130.6 (s), 129.9 (s), 122.2 (s), 121.9 (s), 19.7 (s); **MS** (ESI) m/z 249 [(M − Cl)$^+$, 100]; **HRMS** m/z calculated for $C_{17}H_{17}N_2$ (M − Cl)$^+$ 249.3192, found 249.3199 (+2.8 ppm error). Spectroscopic data consistent with those reported in the literature.[7]

**[IrCl(COD)(1,3-bis(4-methylphenyl)imidazol-2-ylidine)] (2)**

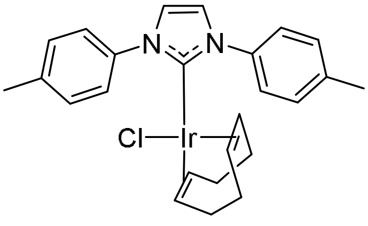


**2**

Using General Procedure C, carbene salt **S2** (142 mg, 0.50 mmol, 2.0 eq), KO$^t$Bu (70 mg, 0.63 mmol, 2.5 eq.), $[IrCl(COD)]_2$ (168 mg, 0.25 mmol, 1.0 eq.) in THF (10 mL) gave complex **2** (204 mg, 70%) as a yellow crystalline solid, **$^1$H NMR** (400 MHz, $CDCl_3$) $\delta$ 7.97 (d, $J$ = 8.0 Hz, 2H), 7.32 (d, $J$ = 8.0 Hz, 2H), 7.29 (s, 2H), 4.52-4.47 (m, 2H), 2.47 (s, 6H), 2.43-2.40 (m, 2H), 1.89-1.77 (m, 2H), 1.51-1.41 (m, 4H), 1.27-1.17 (m, 2H); **$^{13}$C NMR** (100.6 MHz, $CDCl_3$) $\delta$ 180.8 (s), 137.9 (s), 137.7 (s), 129.1 (s), 125.4 (s), 121.9 (s), 83.1 (s), 51.7 (s), 32.9 (s), 29.2 (s), 21.2 (s); **MS** (ESI) m/z 549 [(M − Cl)$^+$, 100]; **HRMS** m/z calculated for $C_{25}H_{28}{}^{193}IrN_2$ (M − Cl)$^+$ 549.1882, found 549.1873 (+0.1 ppm error).

**Synthesis of 3**

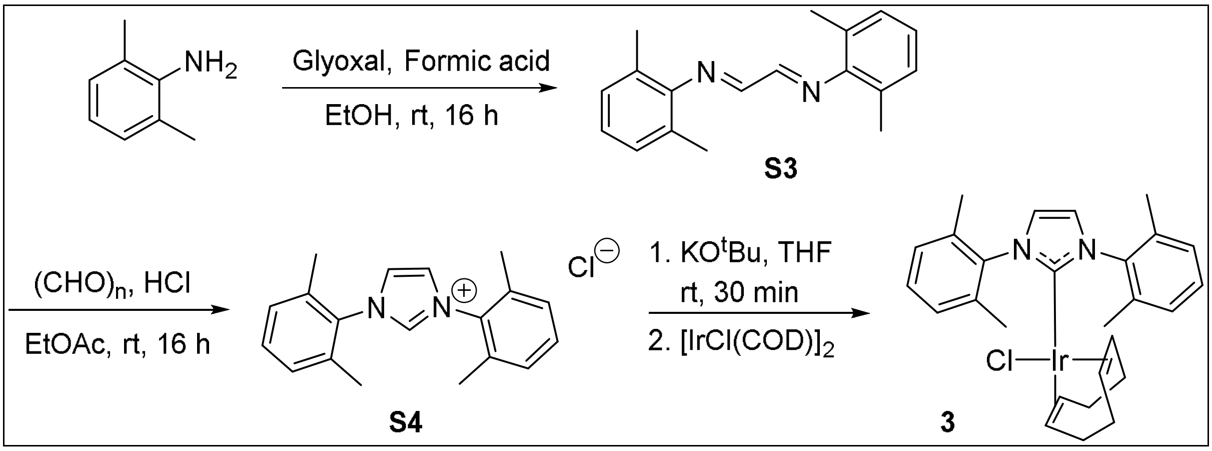


Supplementary Figure 2: Synthesis of **3**.

**N-[2-[(2,6-dimethylphenyl)imino]ethylidine]-2,6-dimethylaniline S3**

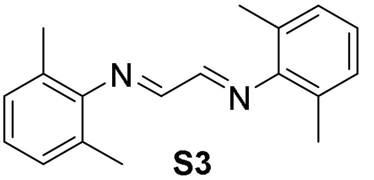

**S3**

Using General Procedure A, 2,6-dimethylaniline (2.71 mL, 22.0 mmol, 2.0 eq.), glyoxal (1.28 mL of a 40% solution in $H_2O$, 11.0 mmol, 1.0 eq.), formic acid (2 drops) in MeOH (20 mL) gave ethylenediimine **S3** (2.12g, 73%) as a yellow powder,**$^1$H NMR** (400 MHz, $CDCl_3$) $\delta$ 8.15 (s, 2H), 7.13 (d, $J$ = 7.5, 4H), 7.03 (t, $J$ = 7.5, 2H), 2.22 (s, 12H); **$^{13}$C NMR** (100.6 MHz, $CDCl_3$) $\delta$ 163.47 (s), 149.87 (s), 128.30 (s), 126.45 (s), 124.79 (s), 18.25 (s); **MS** (ESI) m/z

265 [(M + H)$^+$, 100]; **HRMS** m/z calculated for $C_{18}H_{21}N_2$ (M + H)$^+$ 265.1699, found 265.1704 (−1.3 ppm error).

**1,3-Bis-(2,6-dimethylphenyl)imidazolium chloride S4**

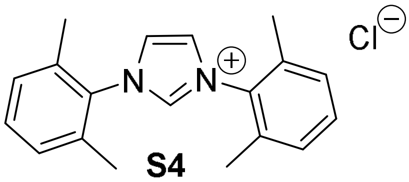

**S4**

Using General Procedure B, ethylenediimine **S3** (880 mg, 3.35 mmol, 1.0 eq), paraformaldehyde (113 mg, 3.69 mmol, 1.1 eq.), HCl (1.27 mL of a 4 M solution in 1,4-dioxane, 5.03 mmol, 1.5 eq.) in EtOAc (30 mL) gave carbene salt **S4** (762 mg, 72%) as a white powder, **$^1$H NMR** (400 MHz, $(CD_3)_2SO$) $\delta$ 9.73 (s, 1H), 8.35 (d, *J* = 1.4 Hz, 2H)), 7.51 (t, *J* = 7.7 Hz, 2H), 7.41 (d, *J* = 7.6 Hz, 4H), 2.18 (s, 12H); **$^{13}$C NMR** (100.6 MHz, $(CD_3)_2SO$) $\delta$ 138.8 (s), 135.2 (s), 133.9 (s), 131.3 (s), 129.4 (s), 125.2 (s), 17.4 (s); **MS** (ESI) m/z 277 [(M − Cl)$^+$, 100]; **HRMS** m/z calculated for $C_{19}H_{21}N_2$ (M − Cl)$^+$ 277.1699, found 277.1705 (−1.9 ppm error).

**[IrCl(COD)(1,3-bis(2,6-dimethylphenyl)imidazole-2-ylidine)] (3)**

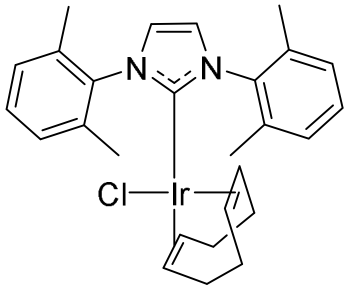

**3**

Using General Procedure C, carbene salt **S4** (200 mg, 0.72 mmol, 2.2 eq), KO$^t$Bu (89 mg, 0.79 mmol, 2.5 eq.), $[IrCl(COD)]_2$ (221 mg, 0.33 mmol, 1.0 eq.) in THF (10 mL) gave complex **3** (384 mg, 95%) as a yellow crystalline solid,**$^1$H NMR** (400 MHz, $CDCl_3$) $\delta$ 7.33 (t, *J* = 8 Hz, 2H), 7.21 (br t, *J* = 8 Hz, 4H), 7.03 (s, 2H), 4.20-4.15 (m, 2H), 3.00-2.95 (m, 2H), 2.43 (s, 6H), 2.24 (s, 6H), 1.78-1.61 (m, 4H), 1.41-1.23 (m, 4H); **$^{13}$C NMR** (100.6 MHz, $CDCl_3$) $\delta$ 180.6 (s), 138.4 (s), 137.8 (s), 134.8 (s), 129.0 (s), 128.8 (s), 127.5 (s), 123.2 (s), 82.9 (s), 51.6 (s), 33.5 (s), 28.9 (s), 19.8 (s), 18.3 (s); **MS** (ESI) m/z 577 [(M − Cl)$^+$, 100]; **HRMS** m/z calculated for $C_{27}H_{32}{}^{193}IrN_2$ (M − Cl)$^+$ 577.2190, found 577.2198 (+1.6 ppm error).

## Synthesis of 4

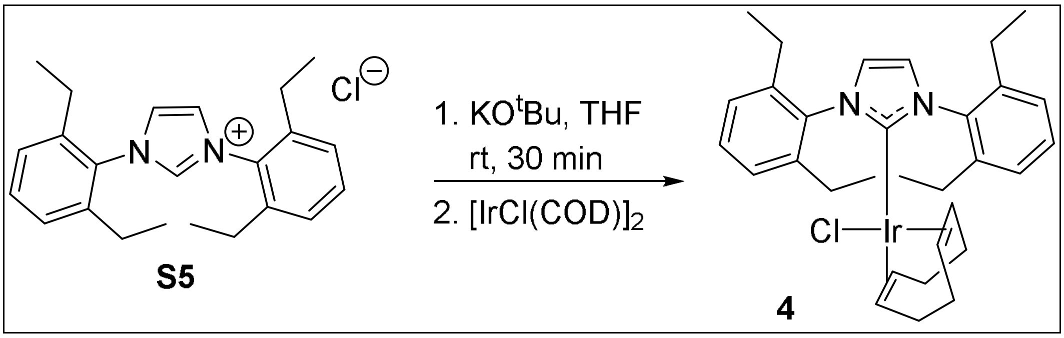


Supplementary Figure 3: Synthesis of **4**.

**[IrCl(COD)(1,3-bis(2,6-diethylphenyl)imidazol-2-ylidine)] (4)**

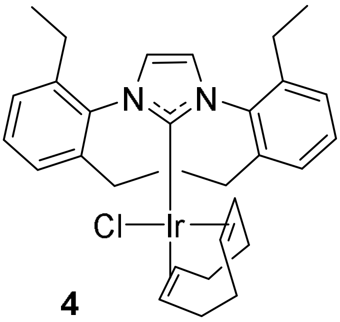


**4**

Using General Procedure C, carbene salt **S5**[5] (369mg, 1.00 mmol, 2.0 eq), KO$^t$Bu (140 mg, 1.25 mmol, 2.5 eq.), [IrCl(COD)]$_2$ (335 mg, 0.50 mmol, 1.0 eq.) in THF (10 mL) gave complex **4** (402 mg, 60%) as a yellow crystalline solid, **$^1$H NMR** (400 MHz, $CDCl_3$) $\delta$ 7.47 (t, $J$ = 8 Hz, 2H), 7.33-7.25 (m, 4H), 7.08 (s, 2H), 4.16-4.10 (m, 2H), 3.00 – 2.87 (m, 4H), 2.81-2.69 (m, 2H), 2.63-2.53 (m, 2H), 2.49-2.38 (m, 2H), 1.70-1.54 (m, 4H), 1.37-1.15 (m, 16H); **$^{13}$C NMR** (100.6 MHz, $CDCl_3$) $\delta$ 181.7 (s), 143.3 (s), 140.6 (s), 137.0 (s), 129.5 (s), 126.9 (s), 125.4 (s), 123.5 (s), 82.6 (s), 51.3 (s), 33.5 (s), 28.8 (s), 25.6 (s), 24.5 (s), 15.3 (s), 15.1 (s); **MS** (ESI) m/z 633 [(M – Cl)$^+$, 100]; **HRMS** m/z calculated for $C_{31}H_{40}{}^{193}IrN_2$ (M – Cl)$^+$ 633.2815, found 633.2811 (+4.2 ppm error).

## Synthesis of 6

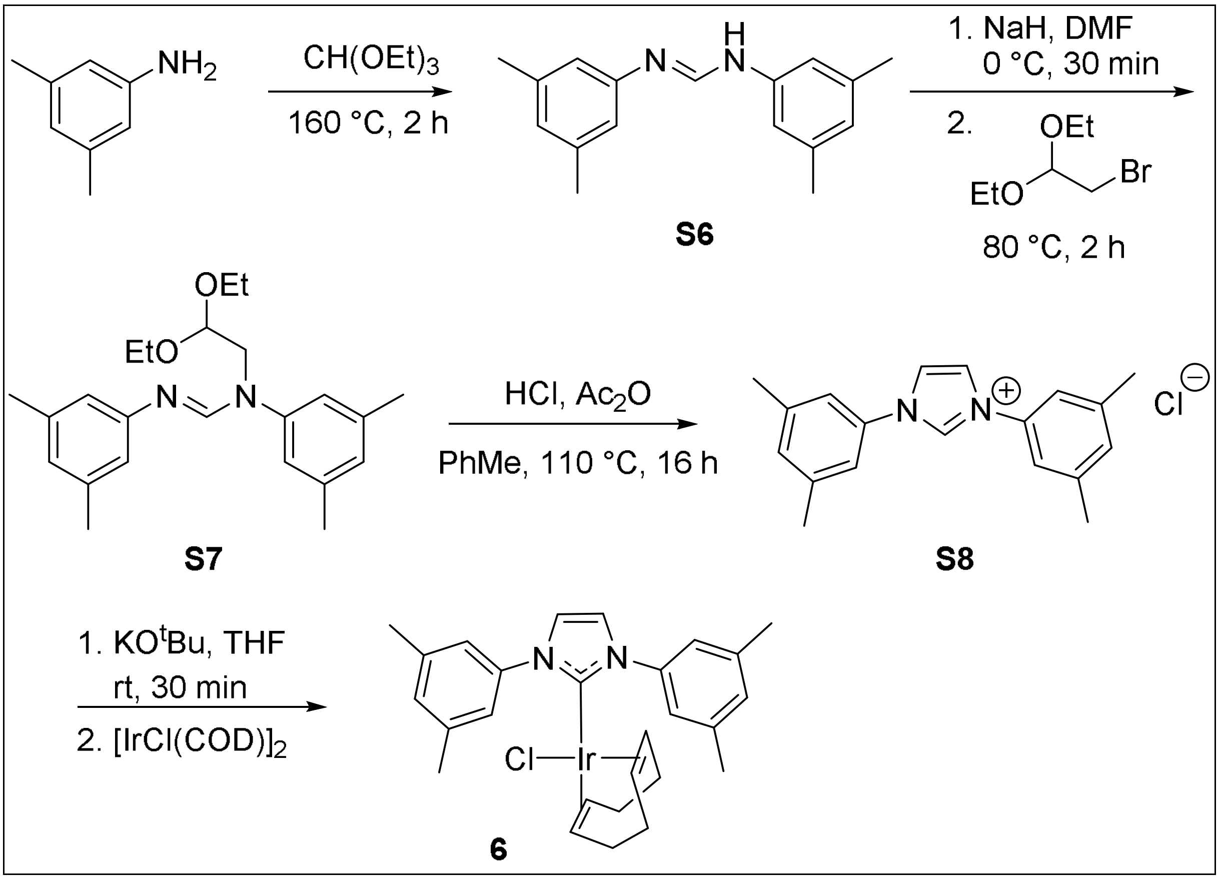


Supplementary Figure 4: Synthesis of **6**.

**N-N-Bis[(3,5-dimethylphenyl)methanimidamide S6**

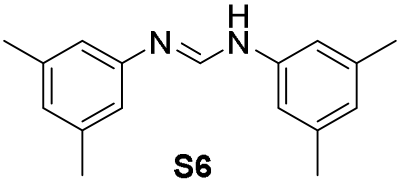


Using General Procedure D, 3,5-dimethylaniline (2.00 mL, 16.0 mmol, 2.0 eq.), triethylorthoformate (1.34 mL, 8.0 mmol, 1.0 eq.) gave formamidine **S6** (1.14 g, 56%) as a white powder, **$^{1}$H NMR** (400 MHz, $CDCl_3$) $\delta$ 8.20 (s, 1H), 6.75 (s, 2H), 6.68 (s, 4H), 2.32 (s, 12H); **$^{13}$C NMR** (100.6 MHz, $CDCl_3$) $\delta$ 148.22 (s), 145.02 (s), 139.09 (s), 125.05 (s), 116.64 (s), 21.35 (s); **MS** (ESI) m/z 253 [(M + H)$^{+}$, 100]; **HRMS** m/z calculated for $C_{17}H_{21}N_2$ (M + H)$^{+}$ 253.1699, found 253.1698 (+0.7 ppm error).

**N-(2,2-Diethoxyethyl)-N,N'-bis(3,5-dimethylphenyl)methanimidamide S7**

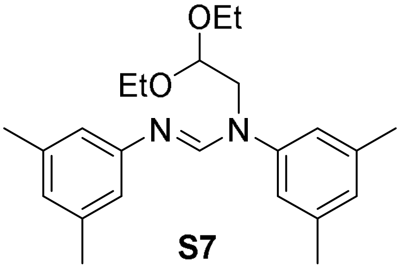

**S7**

Using General Procedure E, formamidine **S6** (500 mg, 1.98 mmol, 1.0 eq), sodium hydride (95 mg of a 60% dispersion in mineral oil, 2.38 mmol, 1.2 eq.), bromoacetaldehyde diethyl acetal (0.60 mL, 3.96 mmol, 2.0 eq.) in DMF (40 mL) gave the crude product. Purification by flash column chromatography on silica with 95:5 hexane-EtOAc as eluent gave diethyl acetal **S7** (544 mg, 75%) as a colourless oil, **$^1$H NMR** (400 MHz, $CD_3OD$) $\delta$ 8.00 (s, 1H), 6.97 (s, 2H), 6.81 (s, 1H), 6.75 (s, 1H), 6.68 (s, 2H), 5.09 (t, $J_{HH}$ = 5.4 Hz, 1H), 4.04 (d, $J_{HH}$ = 5.4 Hz, 2H), 3.81-3.74 (m, 2H), 3.63-3.56 (m, 2H), 2.33 (br s, 12H), 1.21 (t, $J_{HH}$ = 7.1 Hz, 6H); **$^{13}$C NMR** (100.6 MHz, $CD_3OD$) $\delta$ 151.53 (s), 150.53 (s), 145.25 (s), 138.93 (s), 138.66 (s), 126.20 (s), 124.93 (s), 119.83 (s), 118.98 (s), 99.85 (s), 63.47 (s), 51.41 (s), 21.38 (s), 21.33 (s), 15.34 (s); **MS** (ESI) m/z 369 [(M + H)$^+$, 100]; **HRMS** m/z calculated for $C_{23}H_{33}N_2O_2$ (M + H)$^+$ 369.2537, found 369.2523 (+4.4 ppm error).

**1,3-Bis-(3,5-dimethylphenyl)imidazolium chloride S8**

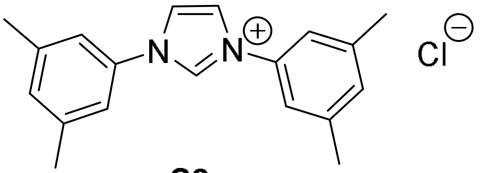

**S8**

Using General Procedure F, diethyl acetal **S7** (387 mg, 1.05 mmol, 1.0 eq), acetic anhydride (0.30 mL, 3.15 mmol, 3.0 eq.), 37% $HCl_{(aq)}$ (0.13 mL, 1.58 mmol, 1.5 eq.) in toluene (15 mL) gave carbene salt **S8** (247 mg, 75%) as a white powder, **$^1$H NMR** (400 MHz, $(CD_3)_2SO$) $\delta$ 10.49 (s, 1H), 8.61 (s, 2H), 7.67 (s, 4H), 7.25 (s, 2H), 2.41 (s, 12H). **$^{13}$C NMR** (100.6 MHz, $(CD_3)_2SO$) $\delta$ 140.2 (s), 135.0 (s), 134.4 (s), 131.5 (s), 122.1 (s), 119.7 (s), 21.3 (s); **MS (**ESI) m/z 277 [(M − Cl)$^+$, 100]; **HRMS** m/z calculated for $C_{19}H_{21}N_2$ (M − Cl)$^+$ 277.1699, found 277.1691 (+3.3 ppm error).

**[IrCl(COD)(1,3-bis(3,5-dimethylphenyl)imidazol-2-ylidine)] 6**

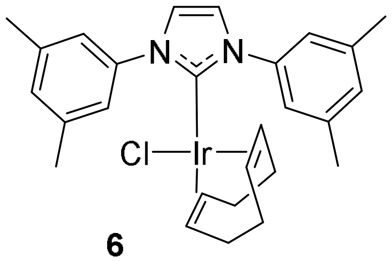


**6**

$Ag_2O$ (74 mg, 0.32 mmol, 2.2 eq.) and $[IrCl(COD)]_2$ (98 mg, 0.15 mmol, 1.0 eq.) were added sequentially to a stirred solution of carbene salt **S8** (100 mg, 0.32 mmol, 2.2 eq) in $CH_2Cl_2$ (10 mL) at rt under $N_2$. The resulting solution was stirred at rt for 3 h and the filtered through Celite. The eluent was concentrated under reduced pressure to give the crude product. Purification by flash column chromatography on silica with $CH_2Cl_2$ as eluent gave complex **6** (52 mg, 28%) as a yellow crystalline solid,$^{1}$**H NMR** (400 MHz, $CDCl_3$) $\delta$ 7.78 (s, 4H), 7.28 (s, 2H), 7.07 (s, 2H), 4.53 (m, 2H), 2.44 (s, 12H), 2.41 (m, 2H), 1.93-1.80 (m, 2H), 1.57-1.44 (m, 4H), 1.29-1.17 (m, 2H); $^{13}$**C NMR** (100.6 MHz, $CDCl_3$) $\delta$ 180.63 (s), 139.93 (s), 138.30 (s), 129.32 (s), 123.04 (s), 121.53 (s), 82.24 (s), 51.61 (s), 33.00 (s), 29.29 (s), 21.33 (s); **MS (**ESI) m/z 591 [(M − Cl)$^+$, 100]; **HRMS** m/z calculated for $C_{28}H_{34}{}^{193}IrN_2$ (M − Cl)$^+$ 591.2351, found 591.2362 (+1.8 ppm error).

**Synthesis of 7**

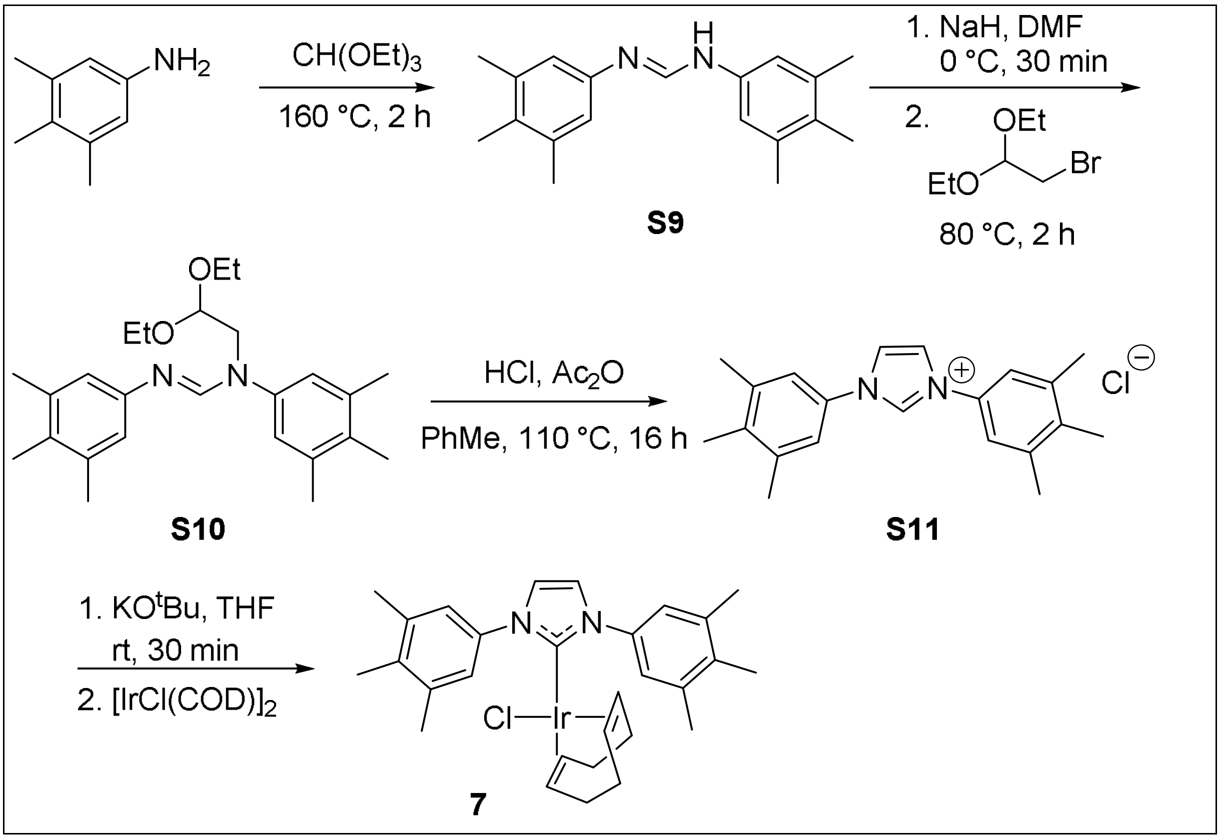


Supplementary Figure 5: Synthesis of **7**.

**N-N-Bis[(3,4,5-trimethylphenyl)methanimidamide S9**

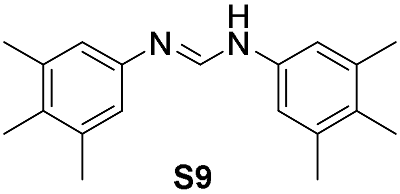

**S9**

Using General Procedure D, 3,4,5-trimethylaniline (1.00 g, 7.40 mmol, 2.0 eq.), triethylorthoformate (0.62 mL, 3.70 mmol, 1.0 eq.) gave formamidine **S9** (964 mg, 93%) as a white powder, **$^{1}$H NMR** (400 MHz, $CD_3OD$) $\delta$ 8.17 (s, 1H), 6.72 (s, 4H), 2.28 (s, 12H), 2.16 (s, 6H); **$^{13}$C NMR** (100.6 MHz, $CD_3OD$) $\delta$ 148.51 (s), 142.46 (s), 137.43 (s), 129.86 (s), 118.14 (s), 20.69 (s), 14.81 (s); **MS (**ESI) m/z 281 [$(M + H)^+$, 100]; **HRMS** m/z calculated for $C_{19}H_{25}N_2$ $(M + H)^+$ 281.2012, found 281.2008 (+1.6 ppm error).

**N-(2,2-Diethoxyethyl)-N,N'-bis(3,4,5-trimethylphenyl)methanimidamide S10**

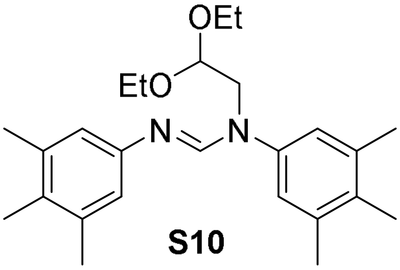

**S10**

Using General Procedure E, formamidine **S9** (500 mg, 1.78 mmol, 1.0 eq), sodium hydride (86 mg of a 60% dispersion in mineral oil, 2.15 mmol, 1.2 eq.), bromoacetaldehyde diethyl acetal (0.54 mL, 3.56 mmol, 2.0 eq.) in DMF (40 mL) gave the crude product. Purification by flash column chromatography on silica with 95:5 hexane-EtOAc as eluent gave diethyl acetal **S10** (644 mg, 91%) as a colourless oil, **$^{1}$H NMR** (400 MHz, $CD_3OD$) $\delta$ 7.98 (s, 1H), 7.00 (s, 2H), 6.72 (s, 2H), 5.09 (t, $J_{HH}$ = 5.4 Hz, 1H), 4.01 (d, $J_{HH}$ = 5.5 Hz, 2H), 3.80-3.74 (m, 2H), 3.64-3.56 (m, 2H), 2.30 (br. s, 12H), 2.17 (br. s, 6H), 1.22 (t, $J_{HH}$ = 7.0 Hz, 6H); **$^{13}$C NMR** (100.6 MHz, $CD_3OD$) $\delta$ 150.52 (s), 148.81 (s), 142.51 (s), 137.38 (s), 137.12 (s), 131.43 (s), 129.74 (s), 121.33 (s), 120.27 (s), 99.85 (s), 63.51 (s), 51.54 (s), 20.76 (s), 20.69 (s), 15.38 (s), 14.90 (s), 14.87 (s); **MS (**ESI) m/z 397 [$(M + H)^+$, 100]; **HRMS** m/z calculated for $C_{25}H_{37}N_2O_2$ $(M + H)^+$ 397.2850, found 397.2544 (+2.8 ppm error).

**1,3-Bis-(3,4,5-trimethylphenyl)imidazolium chloride S11**

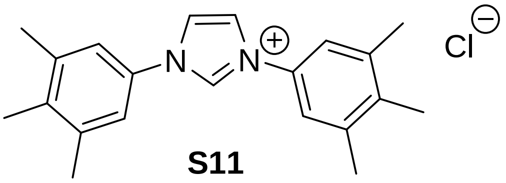

**S11**

Using General Procedure F, diethyl acetal **S10** (359 mg, 0.90 mmol, 1.0 eq), acetic anhydride (0.26 mL, 3.71 mmol, 3.0 eq.), 37% $HCl_{(aq)}$ (0.11 mL, 1.35 mmol, 1.5 eq.) in toluene (15 mL) gave carbene salt **S11** (205 mg, 67%) as a white powder, **$^{1}$H NMR** (400 MHz, $(CD_3)_2SO$) $\delta$ 10.29 (s, 1H), 8.53 (s, 2H), 7.62 (s, 4H), 2.39 (s, 12H), 2.24 (s, 6H); **$^{13}$C NMR** (100.6 MHz, $(CD_3)_2SO$) $\delta$ 139.7 (s), 137.5 (s), 133.8 (s), 132.2 (s) 122.0 (s), 120.7 (s), 20.8 (s), 15.7 (s); **MS (**ESI) m/z 305 [(M − Cl)$^+$, 100]; **HRMS** m/z calculated for $C_{21}H_{25}N_2$ (M − Cl)$^+$ 305.2012, found 305.2000 (+3.6 ppm error).

**[IrCl(COD)(1,3-bis(3,4,5-trimethylphenyl)imidazol-2-ylidine)] 7**

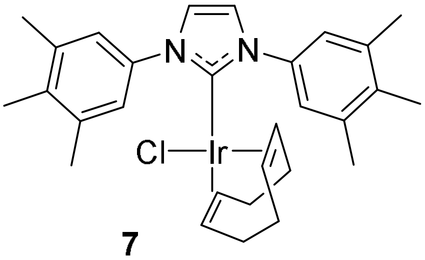


**7**

$Ag_2O$ (61 mg, 0.26 mmol, 2.2 eq.) and $[IrCl(COD)]_2$ (81 mg, 0.12 mmol, 1.0 eq.) were added sequentially to a stirred solution of carbene salt **S11** (90 mg, 0.26 mmol, 2.2 eq) in $CH_2Cl_2$ (10 mL) at rt under $N_2$. The resulting solution was stirred at rt for 3 h and the filtered through Celite. The eluent was concentrated under reduced pressure to give the crude product. Purification by flash column chromatography on silica with $CH_2Cl_2$ as eluent gave complex **7** (104 mg, 68%) as a yellow crystalline solid,**$^{1}$H NMR** (400 MHz, $CDCl_3$) $\delta$ 7.80 (s, 4H), 7.25 (s, 2H), 4.51 (m, 2H), 2.46 (m, 2H), 2.41 (s, 12H), 2.26 (s, 6H), 1.92-1.80 (m, 2H), 1.57-1.44 (m, 4H), 1.30-1.17 (m, 2H); **$^{13}$C NMR** (100.6 MHz, $CDCl_3$) $\delta$ 180.09 (s), 137.20 (s), 136.72 (s), 134.62 (s), 124.07 (s), 121.45 (s), 81.89 (s), 51.51 (s), 33.00 (s), 29.28 (s), 20.63 (s), 15.26 (s); **MS (**ESI) m/z 605 [(M − Cl)$^+$, 100]; **HRMS** m/z calculated for $C_{29}H_{36}{}^{193}IrN_2$ (M − Cl)$^+$ 605.2508, found 605.2501 (−1.2 ppm error).

**Synthesis of 8**

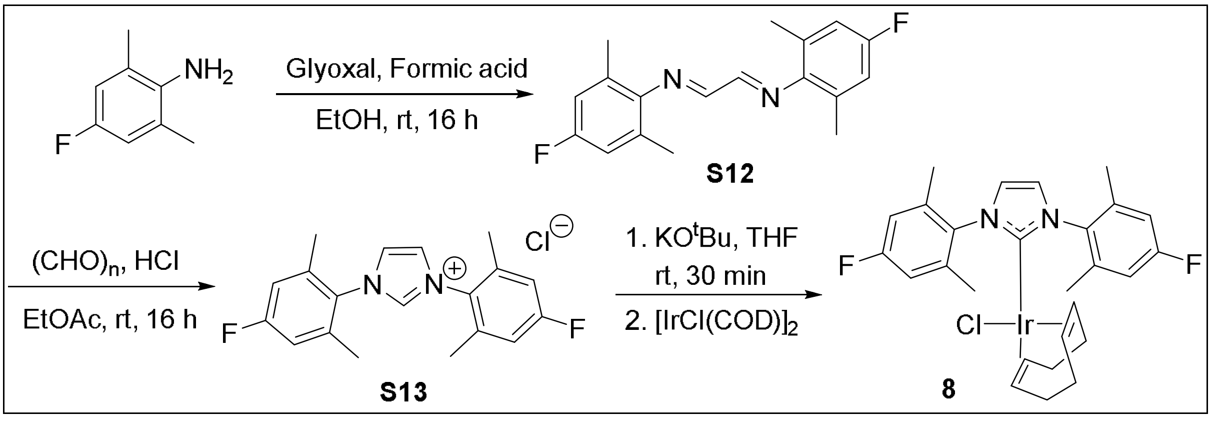


Supplementary Figure 6: Synthesis of **8**.

**4-Fluoro-N-[2-[(4-fluoro-2,6-dimethylphenyl)imino]ethylidine]-2,6-dimethylaniline S12**

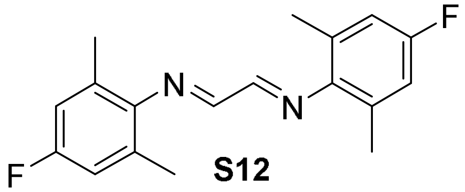

Using General Procedure A, 4-fluoro-2,6-dimethylaniline (1.00 g, 7.19 mmol, 2.0 eq.), glyoxal (419 µL of a 40% solution in $H_2O$, 3.59 mmol, 1.0 eq.), formic acid (2 drops) in EtOH (20 mL) gave ethylenediimine **S12** (560 mg, 52%) as a yellow powder, $^{1}$**H NMR** (400 MHz, $CDCl_3$) 8.10 (s, 2H), 6.81 (d, $J_{H\text{-}F}$ = 9.2 Hz), 2.19 (s, 12H); $^{13}$**C NMR** (101 MHz, $CDCl_3$) 164.0 (d, $J_{C\text{-}F}$ = 1.1 Hz), 159.9 (d, $J_{C\text{-}F}$ = 242.6 Hz), 145.9 (d, $J_{C\text{-}F}$ = 2.5 Hz), 128.9 (d, $J_{C\text{-}F}$ = 8.3 Hz), 114.9 (d, $J_{C\text{-}F}$ = 22.0 Hz), 18.6 (d, $J_{C\text{-}F}$ = 1.2 Hz); **MS** (ESI) m/z 301 [(M + H)$^+$, 100]; **HRMS** m/z calculated for $C_{18}H_{19}F_2N_2$ (M + H)$^+$ 301.1495, found 301.1511 (+5.0 ppm error).

**1,3-Bis-(4-fluoro-2,6-dimethylphenyl)imidazolium chloride S13**

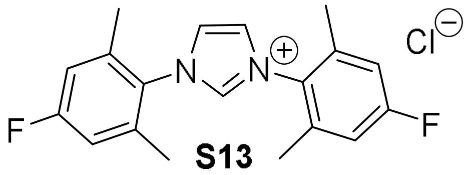

Using General Procedure B, ethylenediimine **S12** (750 mg, 2.33 mmol, 1.0 eq), paraformaldehyde (77 mg, 2.57 mmol, 1.1 eq.), HCl (0.88 mL of a 4 M solution in 1,4-dioxane, 3.50 mmol, 1.5 eq.) in EtOAc (15 mL) gave carbene salt **S13** (683 mg, 84%) as a white powder,$^{1}$**H NMR** (400 MHz, $(CD_3)_2SO$) $\delta$ 9.78 (s, 1H), 8.345 (s, 2H), 7.35 (d, $J_{H\text{-}F}$ = 9.0 Hz, 4H), 2.20 (s, 12H); $^{13}$**C NMR** (100.6 MHz, $(CD_3)_2SO$) $\delta$ 162.8 (d, $J_{C\text{-}F}$ = 248 Hz), 139.6 (s), 138.4 (d, $J_{C\text{-}F}$ = 10 Hz), 130.2 (d, $J_{C\text{-}F}$ = 3 Hz), 125.3, 115.9 (d, $J_{C\text{-}F}$ = 23 Hz), 17.6 (s); **MS** (ESI) m/z 313 [(M − Cl)$^+$, 100]; **HRMS** m/z calculated for $C_{19}H_{19}F_2N_2$ (M − Cl)$^+$ 313.1511, found 313.1501 (+2.9 ppm error).

**[IrCl(COD)(1,3-bis(4-fluoro-2,6-dimethylphenyl)imidazol-2-ylidine)] 8**

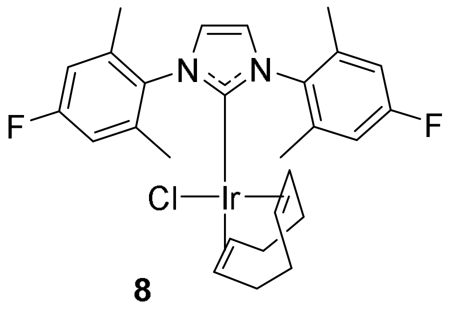

Using General Procedure C, carbene salt **S13** (320 mg, 0.88 mmol, 2.2 eq), KO$^t$Bu (112 mg, 1.00 mmol, 2.5 eq.), [IrCl(COD)]$_2$ (268 mg, 0.40 mmol, 1.0 eq.) in THF (10 mL) gave complex **8** (488 mg, 94%) as a yellow crystalline solid,$^1$**H NMR** (400 MHz, CDCl$_3$) $\delta$ 7.00 (s, 2H), 6.92 (br t, $J$ = 8.8 Hz, 4H), 4.27-4.21 (m, 2H), 2.96-2.91 (m, 2H), 2.40 (s, 6H), 2.22 (s, 6H), 1.83-1.66 (m, 4H), 1.45-1.27 (m, 4H); $^{13}$**C NMR** (100.6 MHz, CDCl$_3$) $\delta$ 181.7 (s), 162.2 (d, $J_{C\text{-}F}$ = 247 Hz), 140.3 (d, $J_{C\text{-}F}$ = 9 Hz), 137.1 (d, $J_{C\text{-}F}$ = 9 Hz), 134.4 (s), 123.4 (s), 115.4 (d, $J_{C\text{-}F}$ = 21 Hz), 114.0 (d, $J_{C\text{-}F}$ = 21 Hz), 83.7 (s), 51.6 (s), 33.5 (s), 28.9 (s), 20.1 (s), 15.5 (s); **MS** (ESI) m/z 613 [(M − Cl)$^+$, 100]; **HRMS** m/z calculated for $C_{27}H_{30}F_2{}^{193}IrN_2$ (M − Cl)$^+$ 613.2001, found 613.1992 (+2.3 ppm error).

**Synthesis of 9**

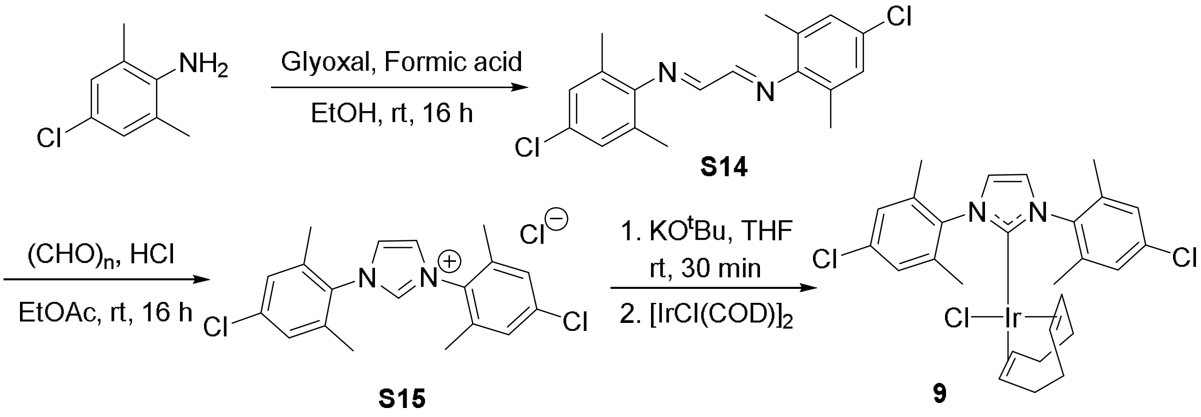


Supplementary Figure 7: Synthesis of **9**.

**4-Chloro-N-[2-[(4-Chloro-2,6-dimethylphenyl)imino]ethylidine]-2,6-dimethylaniline S14**

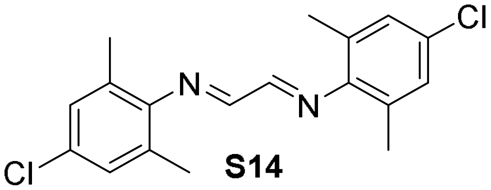


Using General Procedure A, 4-chloro-2,6-dimethylaniline (1.00 g, 6.43 mmol, 2.0 eq.), glyoxal (375 µL of a 40% solution in $H_2O$, 3.59 mmol, 1.0 eq.), formic acid (2 drops) in EtOH (20 mL) gave ethylenediimine **S14** (976 mg, 91%) as a yellow powder,$^1$**H NMR** (500 MHz, CDCl$_3$) $\delta$ 8.10 (s, 2H), 7.11 (s, 4H), 2.18 (s, 12H); $^{13}$**C NMR** (126 MHz, CDCl$_3$) $\delta$ 163.67 (s), 148.19 (s), 129.83 (s), 128.37 (s), 128.08 (s), 18.19 (s); **MS** (ESI) m/z 333 [(M + H)$^+$, 100]; **HRMS** m/z calculated for $C_{18}H_{19}Cl_2N_2$ (M + H)$^+$ 333.0920, found 333.0911 (+3.0 ppm error).

**1,3-Bis-(4-chloro-2,6-dimethylphenyl)imidazolium chloride S15**

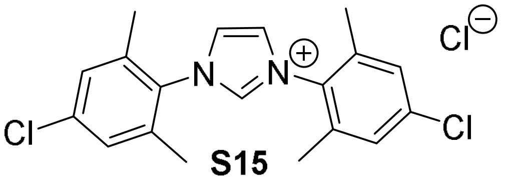


Using General Procedure B, ethylenediimine **S14** (700 mg, 2.10 mmol, 1.0 eq), paraformaldehyde (70 mg, 2.31 mmol, 1.1 eq.), HCl (0.79 mL of a 4 M solution in 1,4-dioxane, 3.15 mmol, 1.5 eq.) in EtOAc (15 mL) gave carbene salt **S15** (625 mg, 78%) as a white powder,**$^1$H NMR** (400 MHz, $CD_3OD$) $\delta$ 8.17 (s, 2H, CH), 7.47 (br s, 4H), 2.25 (s, 12H) ; **$^{13}$C NMR** (100.6 MHz, $CD_3OD$) $\delta$ 137.0 (s), 136.6 (s), 132.0 (s), 128.8 (s), 127.0 (s), 125.1 (s), 16.1 (s); **MS** (ESI) m/z 345 [(M − Cl)$^+$, 100]; **HRMS** m/z calculated for $C_{19}H_{19}Cl_2N_2$ (M − Cl)$^+$ 345.0920, found 345.0904 (+4.1 ppm error).

**[IrCl(COD)(1,3-bis(4-chloro-2,6-dimethylphenyl)imidazol-2-ylidine)] 9**

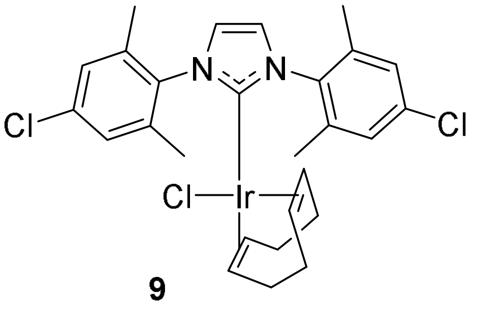


Using General Procedure C, carbene salt **S15** (219 mg, 0.55 mmol, 2.2 eq), KO$^t$Bu (68 mg, 0.60 mmol, 2.4 eq.), $[IrCl(COD)]_2$ (168 mg, 0.25 mmol, 1.0 eq.) in THF (10 mL) gave complex **9** (263 mg, 77%) as a yellow crystalline solid, **$^1$H NMR** (400 MHz, $CDCl_3$) $\delta$ 7.23 (s, 2H), 7.21 (s, 2H), 7.00 (s, 2H), 4.29-4.22 (m, 2H), 2.94-2.88 (m, 2H), 2.40 (s, 6H), 2.20 (s, 6H), 1.82-1.65 (m, 4H), 1.47-1.28 (m, 4H); **$^{13}$C NMR** (100.6 MHz, $CDCl_3$) $\delta$ 181.3 (s), 139.6 (s), 136.9 (s), 136.5 (s), 134.5 (s), 128.8 (s), 127.4 (s), 123.3 (s), 84.1 (s), 51.7 (s), 33.5 (s), 28.9 (s), 19.8 (s), 18.3 (s); **MS** (ESI) m/z 645 [(M − Cl)$^+$, 100]; **HRMS** m/z calculated for $C_{27}H_{30}{}^{35}Cl_2{}^{193}IrN_2$ (M − Cl)$^+$ 645.1410, found 645.1392 (+1.3 ppm error).

## Synthesis of 10

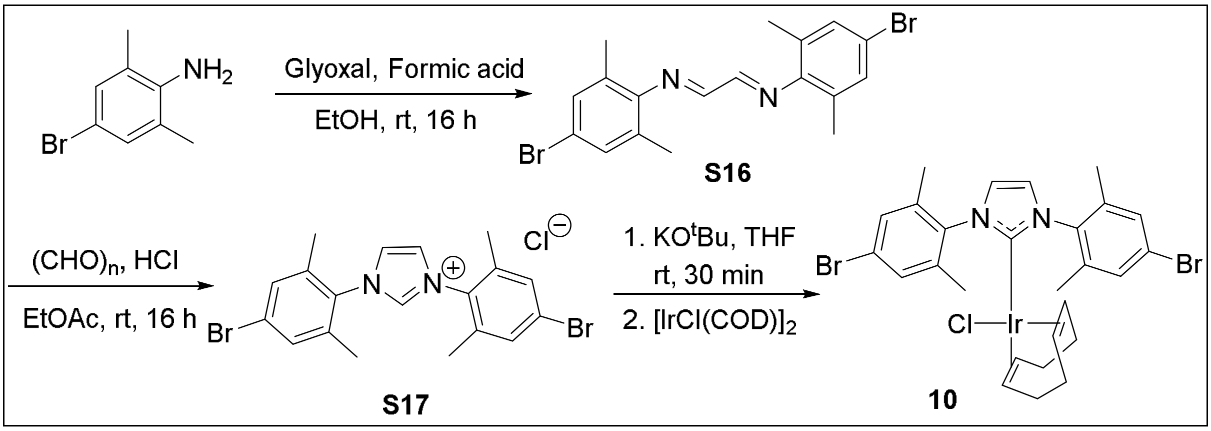


Supplementary Figure 8: Synthesis of **10**.

**4-Bromo-N-[2-[(4-bromo-2,6-dimethylphenyl)imino]ethylidine]-2,6-dimethylaniline S16**

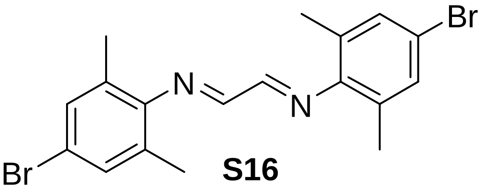


Using General Procedure A, 4-bromo-2,6-dimethylaniline (5.60 g, 28.0 mmol, 2.0 eq.), glyoxal (158 mL of a 40% solution in $H_2O$, 14.0 mmol, 1.0 eq.), formic acid (2 drops) in EtOH (30 mL) gave ethylenediimine **S16** (5.23 mg, 88%) as a yellow powder, **$^1$H NMR** (500 MHz, $CDCl_3$) *δ* 8.09 (s, 2H), 7.26 (s, 4H), 2.18 (s, 12H); **$^{13}$C NMR** (126 MHz, $CDCl_3$) *δ* 163.58 (s), 148.69 (s), 130.98 (s), 128.67 (s), 117.77 (s), 18.10 (s); **MS** (ESI) m/z 421 [(M + H)$^+$, 100]; **HRMS** m/z calculated for $C_{18}H_{19}Br_2N_2$ (M + H)$^+$ 420.9909, found 420.9911 (+1.5 ppm error).

**1,3-Bis-(4-bromo-2,6-dimethylphenyl)imidazolium chloride S17**

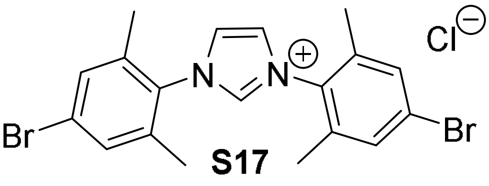


Using General Procedure B, ethylenediimine **S16** (3.10 mg, 7.40 mmol, 1.0 eq), paraformaldehyde (289 mg, 9.62 mmol, 1.3 eq.), HCl (2.78 mL of a 4 M solution in 1,4-dioxane, 11.1 mmol, 1.5 eq.) in EtOAc (40 mL) gave carbene salt **S17** (2.92 g, 84%) as an off-white powder,**$^1$H NMR** (400 MHz, $CD_3OD$) *δ* 9.60 (t, *J* = 1.5Hz, 1H), 8.16 (d, J = 1.5 Hz, 2H), 7.62 (s, 4H), 2.24 (s, 12H); **$^{13}$C NMR** (100.6 MHz, $CD_3OD$) *δ* 138.5 (s), 137.1 (s),

132.5 (s), 131.8 (s), 125.1 (s), 124.8 (s), 16.0 (s); **MS** (ESI) m/z 432 [(M − Cl)$^+$, 100]; **HRMS** m/z calculated for $C_{19}H_{19}Br_2N_2$ (M + H)$^+$ 432.9909, found 432.9903 (+2.1 ppm error).

**[IrCl(COD)(1,3-bis(4-bromo-2,6-dimethylphenyl)imidazol-2-ylidine)] 10**

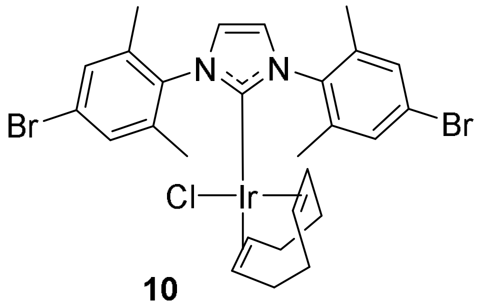

**10**

Using General Procedure C, carbene salt **S17** (259 mg, 0.55 mmol, 2.2 eq), KO$^t$Bu (68 mg, 0.60 mmol, 2.4 eq.), $[IrCl(COD)]_2$ (168 mg, 0.25 mmol, 1.0 eq.) in THF (10 mL) gave complex **10** (319 mg, 83%) as a yellow crystalline solid, **$^1$H NMR** (400 MHz, $CDCl_3$) $\delta$ 7.39 (s, 2H), 7.37 (s, 2H), 6.99 (s, 2H), 4.29-4.24 (m, 2H), 2.94-2.88 (m, 2H), 2.39 (s, 6H), 2.20 (s, 6H), 1.83-1.66 (m, 4H), 1.47-1.28 (m, 4H); **$^{13}$C NMR** (100.6 MHz, $CDCl_3$) $\delta$ 181.2 (s), 139.9 (s), 137.5 (s), 136.8 (s), 131.7 (s), 130.4 (s), 123.3 (s), 122.8 (s), 84.2 (s), 51.8 (s), 33.5 (s), 28.9 (s), 19.7 (s), 18.2 (s); **MS** (ESI) m/z 733 [(M − Cl)$^+$, 100]; **HRMS** m/z calculated for $C_{27}H_{32}{}^{79}Br_2{}^{193}IrN_2$ (M − Cl)$^+$ 733.0400, found 733.0373 (−0.8 ppm error).

**Synthesis of 11**

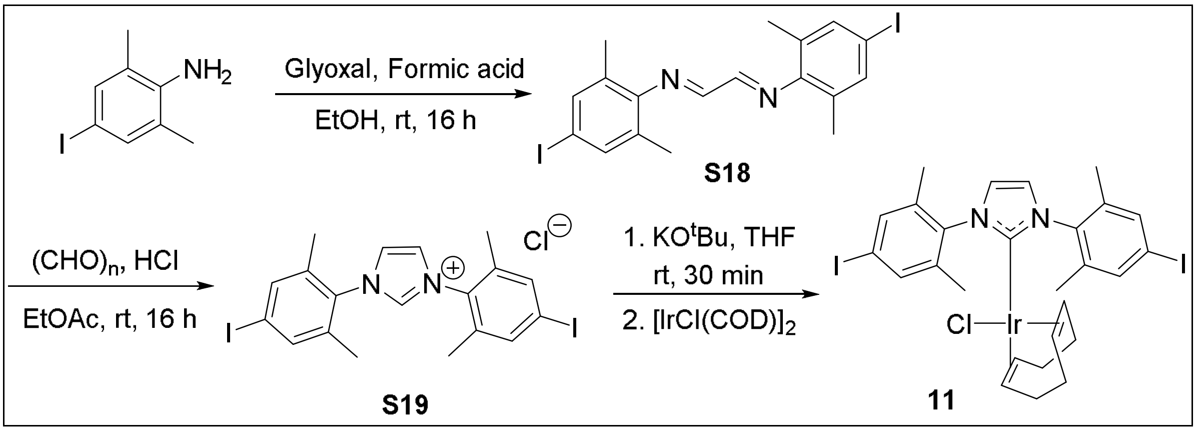


Supplementary Figure 9: Synthesis of **11**.

**4-Iodo-N-[2-[(4-iodo-2,6-dimethylphenyl)imino]ethylidine]-2,6-dimethylaniline S18**

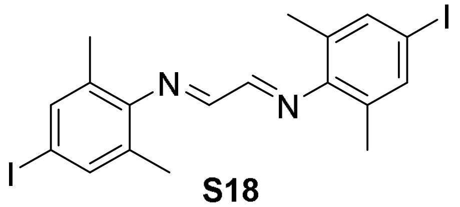

**S18**

Using General Procedure A, 4-iodo-2,6-dimethylaniline (1.00 g, 4.05 mmol, 2.0 eq.), glyoxal (237 µL of a 40% solution in $H_2O$, 2.03 mmol, 1.0 eq.), formic acid (2 drops) in EtOH (20 mL) gave ethylenediimine **S18** (838 mg, 80%) as a yellow powder, **$^1$H NMR** (500 MHz, $CDCl_3$) *δ* 8.12 (s, 2H); **$^{13}$C NMR** (126 MHz, $CDCl_3$) *δ* 163.49 (s), 149.46 (s), 136.96 (s), 128.85 (s), 89.07 (s), 17.86 (s); **MS** (ESI) m/z 538.9 [$(M + Na)^+$, 100], 516.9 [$(M + H)^+$, 100]; **HRMS** m/z calculated for $C_{18}H_{19}I_2N_2$ $(M + H)^+$ 516.9632, found 516.9618 (+1.8 ppm error).

**1,3-Bis-(4-iodo-2,6-dimethylphenyl)imidazolium chloride S19**

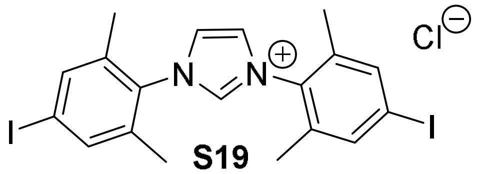

**S19**

Using General Procedure B, ethylenediimine **S18** (425 mg, 0.82 mmol, 1.0 eq), paraformaldehyde (27 mg, 0.91 mmol, 1.1 eq.), HCl (0.30 mL of a 4 M solution in 1,4-dioxane, 1.23 mmol, 1.5 eq.) in EtOAc (15 mL) gave carbene salt **S19** (282 mg, 62%) as an off-white powder, **$^1$H NMR** (400 MHz, $(CD_3)_2SO$) *δ* 9.70 (br. s, 1H), 8.31 (s br., 2H), 7.84 (s, 4H), 2.13 (s, 12H); **$^{13}$C NMR** (100.6 MHz, $(CD_3)_2SO$) *δ* 138.90 (s), 137.79 (s), 137.51 (s), 133.74 (s), 124.97 (s), 98.33 (s), 16.93 (s); **MS** (ESI) m/z 528 [$(M - Cl)^+$, 100]; **HRMS** m/z calculated for $C_{19}H_{19}I_2N_2$ $(M - Cl)^+$ 528.9632, found 528.9621 (+2.0 ppm error).

**[IrCl(COD)(1,3-bis(4-iodo-2,6-dimethylphenyl)imidazol-2-ylidine)] 11**

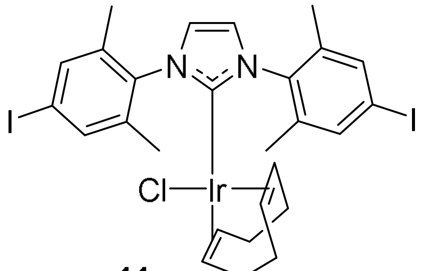

**11**

Using General Procedure C, carbene salt **S19** (200 mg, 0.35 mmol, 2.0 eq), KO$^t$Bu (51 mg, 0.45 mmol, 2.5 eq.), $[IrCl(COD)]_2$ (120 mg, 0.18 mmol, 1.0 eq.) in THF (10 mL) gave complex **11** (202 mg, 65%) as a yellow crystalline solid, **$^1$H NMR** (400 MHz, $CDCl_3$) *δ* 7.60 (s, 2H, 7.59 (s, 2H), 7.00 (s, 2H), 4.27 (m, 2H), 2.91 (m, 2H), 2.38 (s, 6H), 2.18 (s, 6H), 1.76 (m, 4H), 1.44 (m, 2H), 1.34 (m, 2H); **$^{13}$C NMR** (100.6 MHz, $CDCl_3$ ) *δ* 181.1 (s), 140.0 (s), 138.3 (s), 137.8 (s), 136.9 (s), 136.5 (s), 123.3 (s), 94.9 (s), 84.2 (s), 51.8 (s), 33.5 (s), 28.9 (s), 19.5 (s), 18.0 (s); **MS** (ESI) m/z 829 [$(M - Cl)^+$, 100]; **HRMS** m/z calculated for $C_{27}H_{32}I_2{}^{193}IrN_2$ $(M - Cl)^+$ 829.0122, found 829.0120 (−0.2 ppm error).

## Synthesis of 12

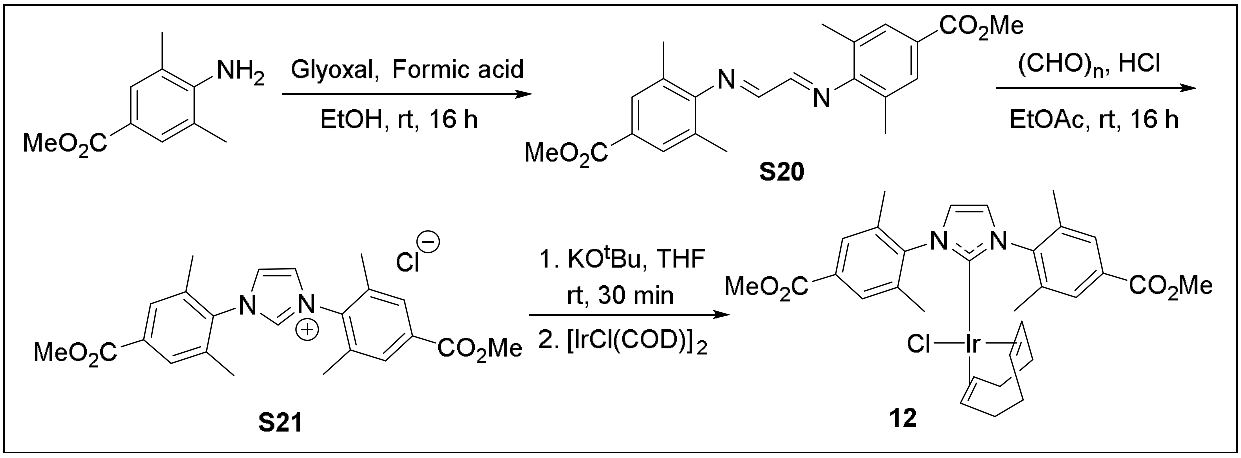


Supplementary Figure 10: Synthesis of **12**.

**Methyl-4-[[-2-{[4-(methoxycarbonyl)-2,6-dimethylphenyl]imino}ethylidene]amino]-3,5-dimethylbenzoate S20**

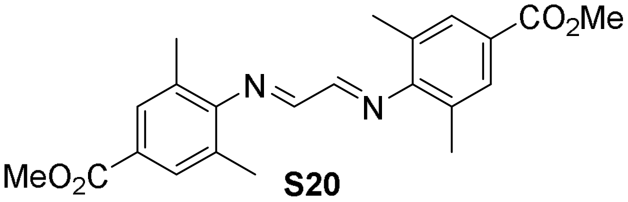


Using General Procedure A, 4-amino-3,5-dimethylbenzoic acid methyl ester (1.00 g, 5.58 mmol, 2.0 eq.), glyoxal (326 µL of a 40% solution in $H_2O$, 2.79 mmol, 1.0 eq.), formic acid (2 drops) in EtOH (20 mL) gave ethylenediimine **S20** (976 mg, 91%) as a yellow powder, $^{1}$**H NMR** (400 MHz, $CDCl_3$) $\delta$ 8.12 (s, 2H), 7.82 (s, 4H), 3.94 (s, 6H), 2.22 (s, 12H); $^{13}$**C NMR** (100.6 MHz, $CDCl_3$) $\delta$ 167.0 (s), 163.1 (s), 153.7 (s), 129.7 (s), 126.4 (s), 52.0 (s), 18.2 (s); **MS** (ESI) m/z 403 [$(M + Na)^+$, 100], 381 [$(M + H)^+$, 30]; **HRMS** m/z calculated for $C_{22}H_{25}N_2O_4$ $(M + H)^+$ 381.1809, found 381.1798 (+3.4 ppm error).

**1,3-Bis-[4-(methoxycarbonyl)-2,6-dimethylphenyl]imidazolium chloride S21**

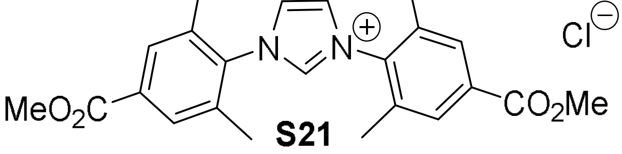


Using General Procedure B, ethylenediimine **S20** (400 mg, 1.05 mmol, 1.0 eq), paraformaldehyde (35 mg, 1.16 mmol, 1.1 eq.), HCl (0.39 mL of a 4 M solution in 1,4-dioxane, 1.58 mmol, 1.5 eq.) in EtOAc (10 mL) gave carbene salt **S21** (341 mg, 76%) as an off-white powder, $^{1}$**H NMR** (400 MHz, $CD_3OD$) $\delta$ 9.68 (br s, 1H), 8.23 (d, $J_{HH}$ = 1.4 Hz, 2H), 8.04 (s, 4H), 3.98 (s, 6H), 2.32 (s, 12H); $^{13}$**C NMR** (100.6 MHz, $CD_3OD$) $\delta$ 165.7 (s), 138.3 (s), 136.8 (s), 135.6 (s), 132.6 (s) 129.8 (s), 125.0 (s), 51.7 (s), 16.3 (s); **MS** (ESI) m/z

393 [(M − Cl)$^+$, 100]; **HRMS** m/z calculated for $C_{23}H_{25}N_2O_4$ (M − Cl)$^+$ 393.1809, found 393.1794 (+5.0 ppm error).

**[IrCl(COD)(1,3-bis(4-(methoxycarbonyl)-2,6-dimethylphenyl)imidazol-2-ylidine)] 12**

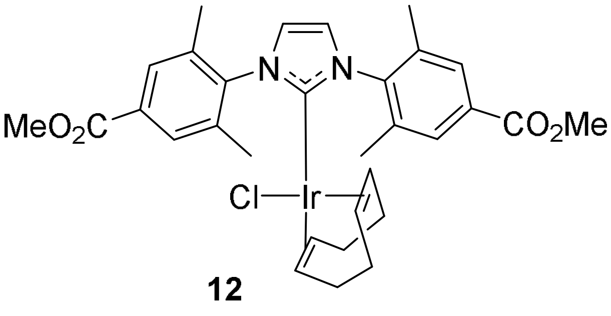


**12**

Using General Procedure C, carbene salt **S21** (200 mg, 0.47 mmol, 2.0 eq), KO$^t$Bu (65 mg, 0.58 mmol, 2.5 eq.), $[IrCl(COD)]_2$ (154 mg, 0.23 mmol, 1.0 eq.) in THF (10 mL) gave complex **12** (281 mg, 84%) as a yellow crystalline solid, **$^1$H NMR** (400 MHz, $CDCl_3$) $\delta$ 7.90 (s, 4H), 7.05 (s, 2H), 4.28-4.20 (m, 2H), 3.97 (s, 6H), 2.92-2.88 (m, 2H), 2.47 (s, 6H), 2.29 (s, 6H), 1.75-1.64 (m, 4H), 1.42-1.38 (m, 2H), 1.32-1.27 (m, 2H); **$^{13}$C NMR** (100.6 MHz, $CDCl_3$ ) $\delta$ 180.5 (s), 166.6 (s), 142.1 (s), 138.3 (s), 135.2 (s), 130.4 (s), 130.0 (s), 128.9 (s), 123.2 (s), 84.4 (s), 52.3 (s), 51.9 (s), 33.5 (s), 28.8 (s), 19.9 (s), 18.5 (s); **MS** (ESI) m/z 693 [(M − Cl)$^+$, 100]; **HRMS** m/z calculated for $C_{31}H_{36}{}^{193}IrN_2O_4$ (M − Cl)$^+$ 693.2299, found 693.2312 (−1.2 ppm error).

__**Synthesis of 13**__

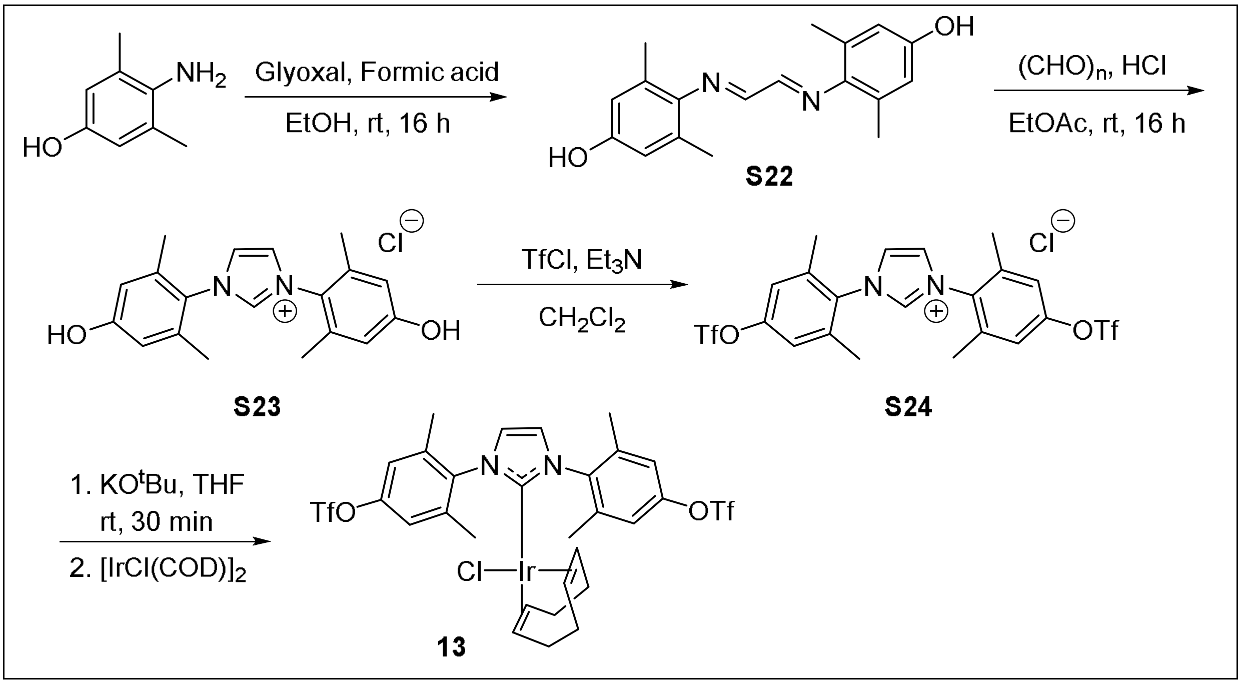


Supplementary Figure 11: Synthesis of **13**.

**4-[-2-[(4-hydroxy-2,6-dimethylphenyl)imino]ethylidene]amino]-3,5-dimethylphenol S22**

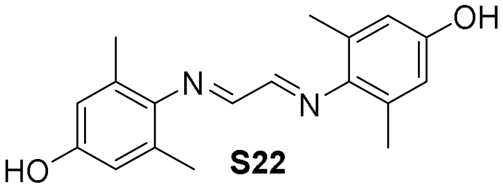

**S22**

Using General Procedure A, 4-amino-3,5-dimethylphenol (1.00 g, 7.23 mmol, 2.0 eq.), glyoxal (425 µL of a 40% solution in $H_2O$, 3.64 mmol, 1.0 eq.), formic acid (2 drops) in EtOH (20 mL) gave ethylenediimine **S22** (759 mg, 70%) as a yellow powder, $^{1}$**H NMR** (400 MHz, $(CD_3)_2SO$) $\delta$ 9.16 (s, 2H), 8.05 (s, 2H), 6.52 (s, 4H), 2.08 (s, 12H); $^{13}$**C NMR** (100.6 MHz, $(CD_3)_2SO$) $\delta$ 163.3 (s), 154.8 (s), 142.0 (s), 128.6 (s), 115.4 (s), 18.8 (s); **MS** (ESI) m/z 297 [(M + H)$^+$, 100]]; **HRMS** m/z calculated for $C_{18}H_{21}N_2O_2$ (M + H)$^+$ 297.1598, found 297.1601 (−1.2 ppm error).

**1,3-Bis-(4-hydroxy-2,6-dimethylphenyl)imidazolium chloride S23**

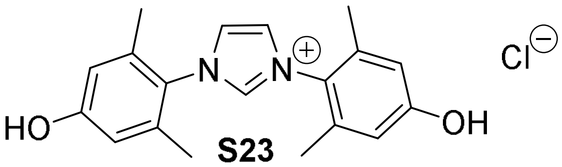

**S23**

Using General Procedure B, ethylenediimine **S22** (500 mg, 1.69 mmol, 1.0 eq), paraformaldehyde (57 mg, 1.86 mmol, 1.1 eq.), HCl (0.63 mL of a 4 M solution in 1,4-dioxane, 2.53 mmol, 1.5 eq.) in EtOAc (15 mL) gave carbene salt **S23** (502 mg, 86%) as an off-white powder, $^{1}$**H NMR** (400 MHz, $(CD_3)_2SO$) 10.3 (s, 2H), 9.62 (s, 1H), 8.21 (s, 2H), 6.78 (s, 4H), 2.05 (s, 12H); $^{13}$**C NMR** (100.6 MHz, $(CD_3)_2SO$) 159.0 (s), 139.1 (s), 135.7 (s), 125.1 (s), 124.8 (s), 115.2 (s), 17.1 (s).; **MS** (ESI) m/z 309 [(M − Cl)$^+$, 100]; **HRMS** m/z calculated for $C_{19}H_{21}N_2O_2$ (M − Cl)$^+$ 309.1598, found 309.1603 (−2.2 ppm error).

**[IrCl(COD)(1,3-bis(4-(trifluoromethanesulfonyl)-2,6-dimethylphenyl)imidazol-2-ylidine)] 13**

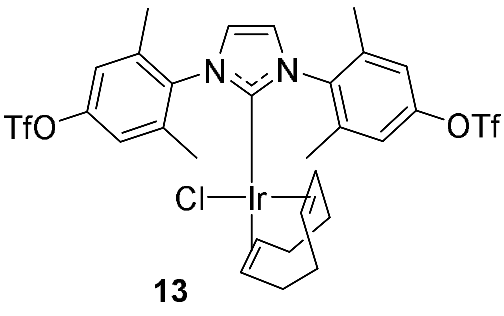

**13**

To a suspension of carbene salt **S23** (300 mg, 0.87 mmol, 1.0 eq.) and $K_2CO_3$ (300 mg, 2.2 mmol, 2.5 eq.) in THF (10 mL) was added trifluoromethanesulfonyl chloride (300 µL, 2.8 mmol, 3.2 eq.) and the reaction mixture was stirred at room temperature for 2 days. Water (20

mL) was added and the mixture was extracted with $CH_2Cl_2$ (2 × 20 mL), dried over $MgSO_4$ and concentrated under reduced pressure. The residue was purified by flash column chromatography with 1:9 MeOH-$CH_2Cl_2$ as eluent to give the bis-trifluoromethanesulfonyl carbene salt which was used directly without further purification in the subsequent reaction. This residue was dissolved in THF (20 mL) and KO$^t$Bu (90 mg, 0.80 mmol) was added, followed by $[Ir(COD)Cl]_2$ (250 mg, 0.37 mmol). The reaction mixture was stirred at room temperature for 18 hours, then concentrated under reduced pressure and purified by flash chromatography with $CH_2Cl_2$ as eluent to give complex **13** (15 mg, 2%) as a yellow solid, **$^1$H NMR** (500 MHz, $CDCl_3$) 7.14 (s, 4H), 7.05 (s, 2H), 4.25-4.24 (m, 2H), 2.82-2.81 (m, 2H), 2.44 (s, 6H), 2.25 (s, 6H), 1.77-1.62 (m, 4H), 1.45-1.28 (m, 4H); **$^{13}$C NMR** (125 MHz, $CDCl_3$) 181.8 (s), 149.1 (s), 141.2 (s), 138.2 (s), 137.8 (s), 123.4 (s), 121.5 (s), 120.3 (s), 118.9 (q, $J$ = 320.9 Hz), 85.3 (s), 52.0 (s), 33.5 (s), 28.9 (s), 20.3 (s), 18.8 (s); **HRMS** (ESI) *m/z* $[M + H]^+$ calculated for $C_{29}H_{30}F_6{}^{193}IrN_2O_6S_2$ $(M – Cl)^+$ 873.1073, found 873.1077 (–0.9 ppm error).

## Synthesis of 14

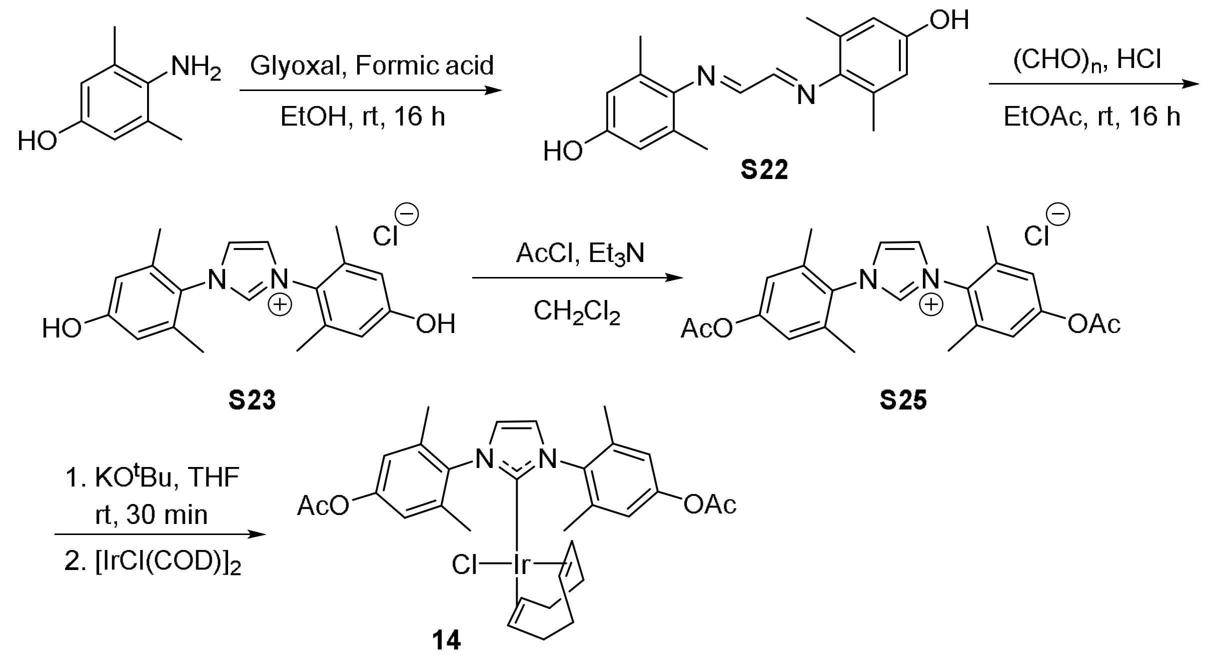


Supplementary Figure 12: Synthesis of **14**.

**[IrCl(COD)(1,3-bis(4-acetoxy-2,6-dimethylphenyl)imidazole-2-ylidine)] 14**

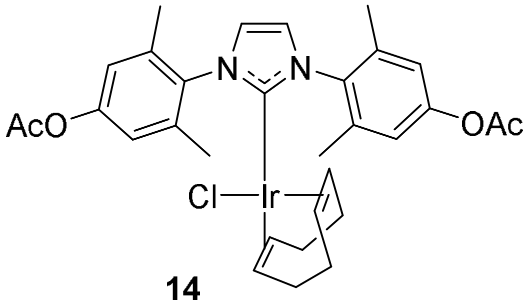


**14**

Acetyl chloride (43 µL, 0.61 mmol, 4.2 eq.) was added to a stirred solution of carbene salt **S23** (100 mg, 0.29 mmol, 2.0 eq) and $Et_3N$ (113 µL, 0.81 mmol, 5.6 eq.) in $CH_2Cl_2$ (10 mL) at rt under $N_2$. The resulting solution was stirred at rt for 18 h. Then, a 10% solution of $NaHCO_{3(aq)}$ was added and the two layers separated. The organic layer was dried ($MgSO_4$) and concentrated under reduced pressure to give a crude residue that contained **S25**. The residue was dissolved in THF (10 mL) and $KO^tBu$ (81 mg, 0.73 mmol, 5.0 eq.) and $[IrCl(COD)]_2$ (100 mg, 0.15 mmol, 1.0 eq.) were added sequentially at rt under $N_2$. The resulting solution was stirred at rt for 2 h and then concentrated under reduced pressure to give the crude product. Purification by flash column chromatography on silica with $CH_2Cl_2$ as eluent gave complex **14** (81 mg, 37%) as a yellow crystalline solid, **$^1$H NMR** (500 MHz, $CD_2Cl_2$) $\delta$ 7.10 (s, 2H), 6.98 (br. s, 4H), 4.14 (m, 2H), 2.99 (m, 2H), 2.41 (s, 6H), 2.35 (s, 6H), 2.25 (s, 6H), 1.76 (m, 4H), 1.41 (m, 2H), 1.33 (m, 2H); **$^{13}$C NMR** (126 MHz, $CD_2Cl_2$) $\delta$ 180.8 (s), 169.3 (s), 150.6 (s), 139.6 (s), 139.4 (s), 136.1 (s), 123.6 (s), 121.5 (s), 120.6 (s), 83.1 (s), 51.9 (s), 33.4 (s), 28.8 (s), 20.9 (s), 19.6 (s), 18.3 (s); **MS** (ESI) m/z 577 [$(M - Cl)^+$, 100]; **HRMS** m/z calculated for $C_{27}H_{32}{}^{193}IrN_2$ $(M - Cl)^+$ 577.2190, found 577.2198 (+1.6 ppm error).

**Synthesis of 15**

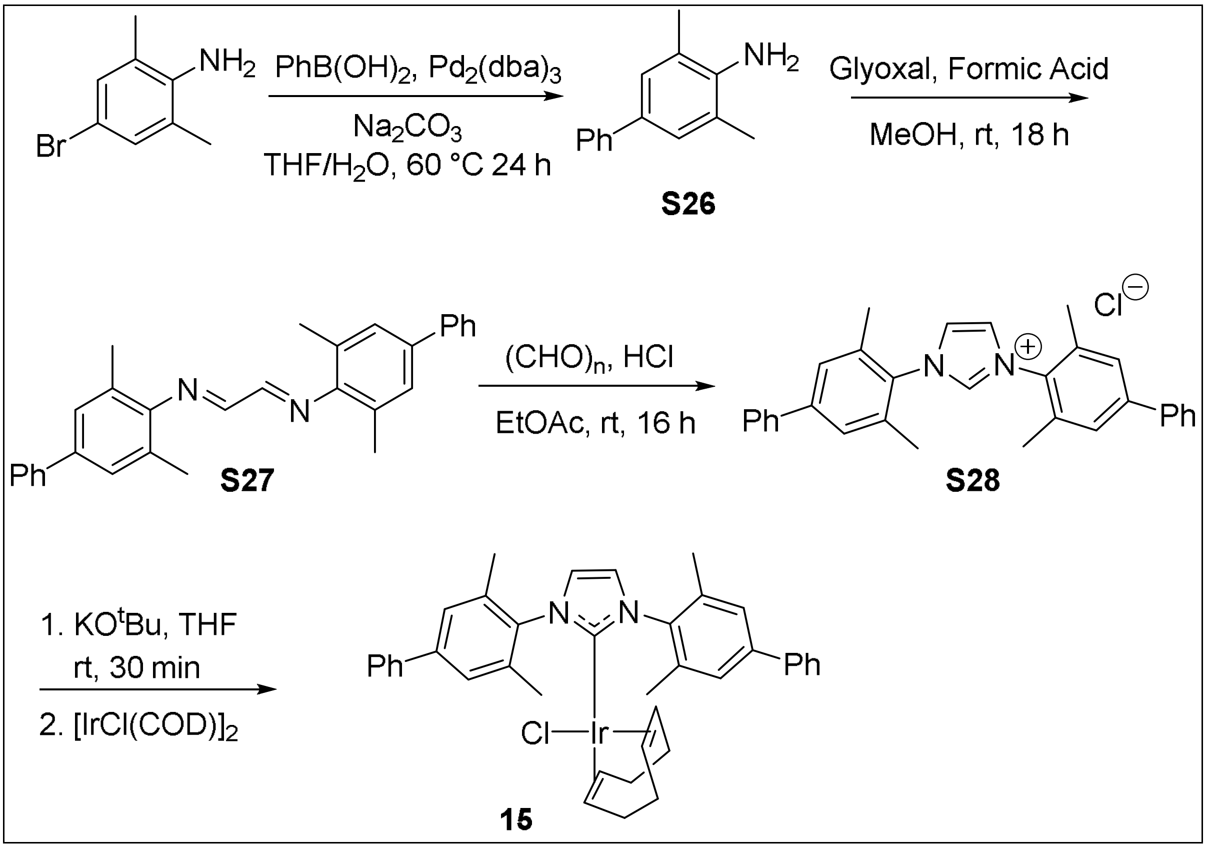


Supplementary Figure 13: Synthesis of **15**.

**4-Phenyl-2,6-dimethylaniline S26**

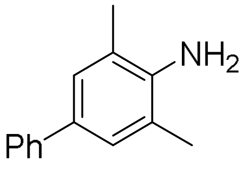

**S26**

4-Bromo-2,6-dimethylaniline (500 mg, 2.5 mmol, 1.0 eq.) and phenylboronic acid (640 mg, 5.25 mmol, 2.1 eq.) were dissolved in THF (10 mL). A 2 M solution of $Na_2CO_{3(aq)}$ (10 mL) was added and nitrogen gas was bubbled through the resulting biphasic mixture for 30 min. $Pd_2(dba)_3.CHCl_3$ (65 mg, 0.125 mmol, 5 mol%) was added and the reaction mixture was stirred under nitrogen at 60 °C for 24 h. The reaction mixture was then cooled to room temperature and quenched with saturated aqueous ammonium chloride. The mixture was extracted with EtOAc (3 x 15 mL), dried over $MgSO_4$ and concentrated under reduced pressure to give the crude product. Purification by flash column chromatography on silica with 9:1 hexane-EtOAc as eluent gave 4-phenyl-2,6-dimethylaniline **S26** (377 mg, 76%) as a colourless oil, **$^{1}$H NMR** (400 MHz, $CDCl_3$) $\delta$ 7.58 (d, $J$ = 8.3, 2H), 7.42 (t, $J$ = 7.6, 2H), 7.29 (t, $J$ = 7.2, 1H), 7.25 (s, 2H), 3.68 (br. s, 2H), 2.29 (s, 6H); **$^{13}$C NMR** (100.6 MHz, $CDCl_3$) $\delta$ 142.3 (s), 141.5 (s), 131.1 (s), 128.6 (s), 127.1 (s), 126.5 (s), 126.1 (s), 122.0 (s), 17.8 (s); **MS**

(ESI) m/z 198 [(M + H)$^+$, 100]; **HRMS** m/z calculated for $C_{14}H_{16}N$ (M + H)$^+$ 198.1277, found 198.1280 (−1.0 ppm error).

**(*N*)-*N*-[(2)-2-[(2,6-dimethyl-4-phenylphenyl)imino]ethylidene]-2,6-dimethyl-4-phenylaniline S27**

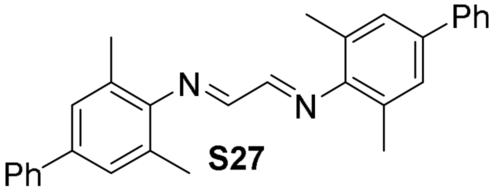

**S27**

Using General Procedure A, aniline **S26** (266 mg, 1.35 mmol, 2.0 eq.), glyoxal (77 µL of a 40% solution in $H_2O$, 0.67 mmol, 1.0 eq.), formic acid (2 drops) in MeOH (10 mL) gave ethylenediimine **S27** (236 mg, 84%) as a yellow powder, **$^1$H NMR** (400 MHz, $CDCl_3$) $\delta$ 8.22 (s, 2H), 7.66 (d, *J* = 7.5, 4H), 7.48 (t, *J* = 7.5, 4H), 7.40 (s, 4H), 7.37 (t, *J* = 7.5, 2H), 2.30 (s, 12H); **$^{13}$C NMR** (100.6 MHz, $CDCl_3$) $\delta$ 163.49 (s), 149.14 (s), 140.80 (s), 137.78 (s), 128.72 (s), 127.11 (s), 127.09 (s), 127.07 (s), 126.93 (s), 18.49 (s); **MS** (ESI) m/z 417 [(M + H)$^+$, 100]; **HRMS** m/z calculated for $C_{30}H_{29}N_2$ (M + H)$^+$ 417.2325, found 417.2331 (−0.6 ppm error).

**1,3-Bis-(2,6-dimethyl-4-phenylphenyl)imidazolium chloride S28**

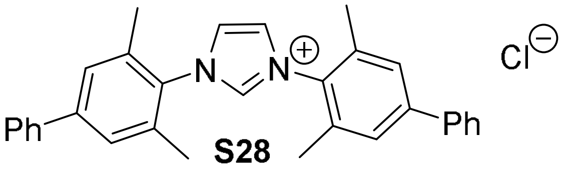

**S28**

Using General Procedure B, ethylenediimine **S27** (500 mg, 1.20 mmol, 1.0 eq), paraformaldehyde (40 mg, 1.32 mmol, 1.1 eq.), HCl (0.45 mL of a 4 M solution in 1,4-dioxane, 1.80 mmol, 1.5 eq.) in EtOAc (10 mL) gave carbene salt **S28** (425 mg, 76%) as a white powder, **$^1$H NMR** (400 MHz, $CDCl_3$) $\delta$ 11.27 (s, 1H), 7.66 (s, 2H), 7.60 (d, *J* = 8.1, 4H), 7.49 (t, *J* = 7.3, 4H), 7.47 (s, 4H), 7.43 (t, *J* = 7.3, 2H), 2.36 (s, 12H); **$^{13}$C NMR** (100.6 MHz, $CDCl_3$) $\delta$ 144.31 (s), 140.43 (s), 139.47 (s), 134.86 (s), 132.12 (s), 128.96 (s), 128.23 (s), 128.15 (s), 127.34 (s), 124.14 (s), 18.12 (s); **MS** (ESI) m/z 429 [(M − Cl)$^+$, 100]; **HRMS** m/z calculated for $C_{31}H_{29}N_2$ (M − Cl)$^+$ 429.2325, found 429.2338 (−1.8 ppm error).

**[IrCl(COD)(1,3-bis(2,6-dimethyl-4-phenylphenyl)imidazol-2-ylidine)] 15**

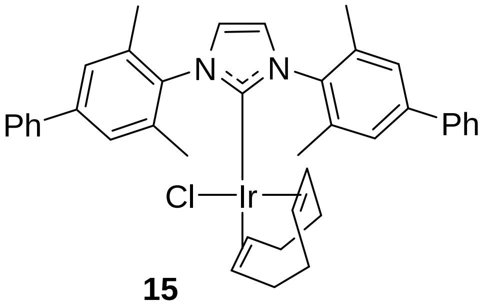

**15**

Using General Procedure C, carbene salt **S28** (70 mg, 0.15 mmol, 2.0 eq), KO$^t$Bu (21 mg, 0.19 mmol, 2.5 eq.), $[IrCl(COD)]_2$ (50 mg, 0.075 mmol, 1.0 eq.) in THF (10 mL) gave complex **15** (61 mg, 53%) as a yellow crystalline solid, **$^1$H NMR** (400 MHz, $CDCl_3$) $\delta$ 7.69 (d, $J$ = 7.5, 4H), 7.52-7.40 (m, 10H), 7.10 (s, 2H), 4.22 (br. s, 2H), 3.07 (br. s, 2H), 2.53 (s, 6H), 2.31 (s, 6H), 1.78-1.65 (m, 4H), 1.41-1.25 (m, 4H); **$^{13}$C NMR** (100.6 MHz, $CDCl_3$) $\delta$ 181.17 (s), 141.76 (s), 140.44 (s), 138.07 (s), 137.72 (s), 135.13 (s), 128.82 (s), 127.61 (s), 127.23 (s), 126.21 (s), 123.34 (s), 83.08 (s), 51.59 (s), 33.52 (s), 28.93 (s), 20.01 (s), 18.56 (s); **MS** (ESI) m/z 729 [(M − Cl)$^+$, 100]; **HRMS** m/z calculated for $C_{39}H_{40}{}^{193}IrN_2$ (M − Cl)$^+$ 729.2820, found 729.2860 (+4.0 ppm error).

**Synthesis of 16**

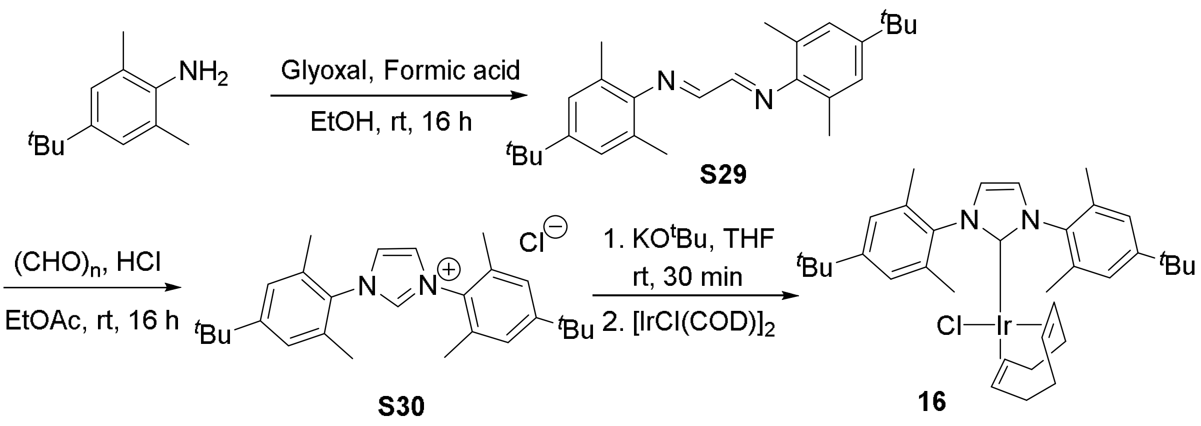


Supplementary Figure 14: Synthesis of **16**.

**1,3-Bis-(4-*tert*-butyl-2,6-dimethylphenyl)imidazolium chloride S30**

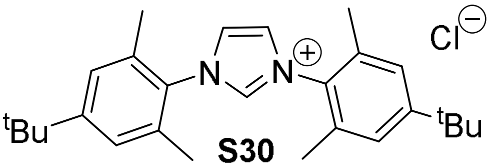

**S30**

Glyoxal (330 µL, 2.83 mmol, 1.0 eq.) and formic acid (2 drops) were added sequentially to a stirred solution of 4-*tert*-butyl-2,6-dimethylaniline (1.00 g, 5.65 mmol, 2.0 eq.) EtOH (15 mL) at rt. The resulting solution was stirred at rt for 16 h. The reaction mixture was concentrated under reduced pressure to give a residue which contained the intermediate

ethylenediimine **S29**. The residue was dissolved in EtOAc (15 mL) and a solution of paraformaldehyde (93 mg, 3.11 mmol, 1.1 eq.) in HCl (1.06 ml of a 4 M solution in dioxane, 4.25 mmol, 1.5 eq.) was added dropwise at rt. The solution was stirred at rt for 16 h, during which time a precipitate formed. The precipitate was filtered, washed with EtOAc and dried under reduced pressure to a crude product that contained carbene salt **S30** (577 mg, 48%, ca. 85% pure) as an off white powder which could be used in the next step without further purification, **$^{1}$H NMR** (400 MHz, $CDCl_3$) $\delta$ 10.02 (s, 1H), 7.71 (s, 2H), 7.23 (s, 4H), 2.20 (s, 12H), 1.33 (s, 18H); **MS** (ESI) m/z 389 [(M − Cl)$^+$, 100]; **HRMS** m/z calculated for $C_{27}H_{37}N_2$ (M − Cl)$^+$ 389.2957, found 389.2951 (−1.5 ppm error).

**[IrCl(COD)(1,3-bis(4-*tert*-butyl-2,6-dimethylphenyl)imidazole-2-ylidine)] 16**

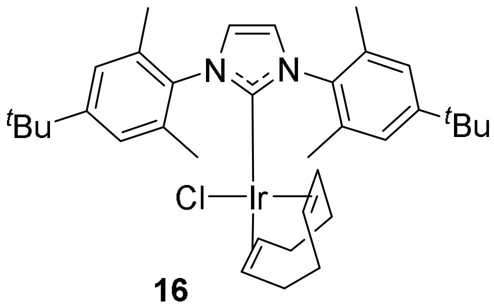


**16**

Using General Procedure C, the crude mixture containing carbene salt **S30** (243 mg, 0.55 mmol, 2.2 eq), KO$^t$Bu (71 mg, 0.63 mmol, 2.5 eq.), $[IrCl(COD)]_2$ (168 mg, 0.25 mmol, 1.0 eq.) in THF (10 mL) gave complex **16** (337 mg, 93%) as a yellow crystalline solid,**$^{1}$H NMR** (400 MHz, $CDCl_3$) $\delta$ 7.23 (s, 2H), 7.19 (s, 2H), 7.04 (s, 2H), 4.16-4.09 (m, 2H), 3.02-2.97 (m, 2H), 2.43 (s, 6H), 2.19 (s, 6H), 1.70-1.54 (m, 4H), 1.39 (s, 18H), 1.33-1.19 (m, 4H); **$^{13}$C NMR** (100.6 MHz, $CDCl_3$) $\delta$ 181.3 (s), 151.9 (s), 136.9 (s), 135.9 (s), 134.1 (s), 125.8 (s), 124.4 (s), 123.1 (s), 82.1 (s), 51.2 (s), 34.6 (s), 33.5 (s), 31.4 (s), 28.9 (s), 20.0 (s), 18.5 (s); **MS** (ESI) m/z 689 [(M − Cl)$^+$, 100]; **HRMS** m/z calculated for $C_{35}H_{48}{}^{193}IrN_2$ (M − Cl)$^+$ 689.3441, found 689.3455 (−1.6 ppm error).

## Synthesis of 17

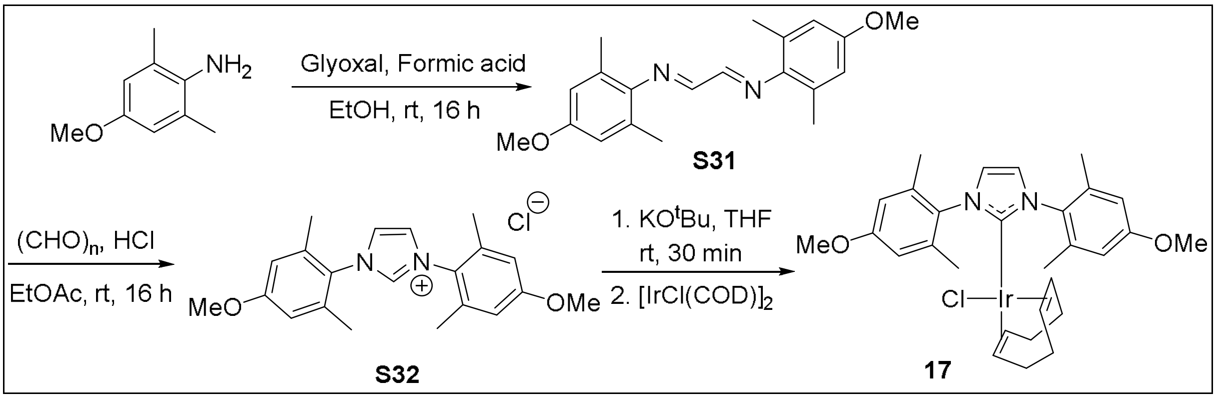


Supplementary Figure 15: Synthesis of **17**.

**_N_-4-Methoxy-[2-[(4-methoxyl-2,6-dimethylphenyl)imino]ethylidine]-2,6-dimethylaniline S31**

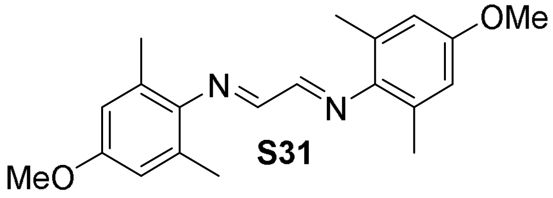


Using General Procedure A, 4-methoxy-2,6-dimethylaniline[8] (2.00 g, 13.2 mmol, 2.0 eq.), glyoxal (0.96 mL of a 40% solution in $H_2O$, 6.6 mmol, 1.0 eq.), formic acid (2 drops) in EtOH (15 mL) gave ethylenediimine **S31** (1.60g, 75%) as a yellow powder, **$^{1}$H NMR** (400 MHz, $CDCl_3$) 8.12 (s, 2H), 6.66 (s, 4H), 3.80 (s, 6H), 2.22 (s, 12H); **$^{13}$C NMR** (101 MHz, $CDCl_3$) 163.5 (s), 156.8 (s), 143.4 (s), 128.7 (s), 113.8 (s), 55.4 (s), 18.8 (s); **MS** (ESI) m/z 325 [$(M + H)^+$, 100]; **HRMS** m/z calculated for $C_{20}H_{25}N_2O_2$ $(M + H)^+$ 325.1911, found 325.1899 (+2.8 ppm error).

**1,3-Bis-(4-methoxy-2,6-dimethylphenyl)imidazolium chloride S32**

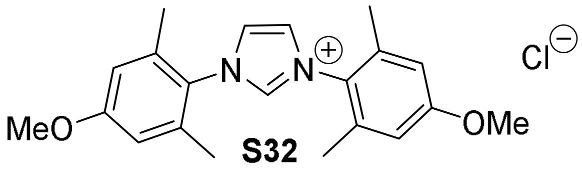


Using General Procedure B, ethylenediimine **S31** (200 mg, 0.61 mmol, 1.0 eq), paraformaldehyde (20 mg, 0.67 mmol, 1.1 eq.), HCl (0.18 mL of a 4 M solution in 1,4-dioxane, 0.74 mmol, 1.2 eq.) in EtOAc (30 mL) gave carbene salt **S32** (192 mg, 84%) as a colourless solid. **$^{1}$H NMR** (400 MHz, $CDCl_3$) $\delta$ 10.87 (s, 1H), 7.61 (s, 2H), 6.72 (s, 4H), 3.83 (s, 6H), 2.20 (s, 12H); **$^{13}$C NMR** (100.6 MHz, $CDCl_3$) $\delta$ 160.8 (s), 140.4 (s), 135.9 (s), 126.0 (s), 124.6 (s), 114.3 (s), 55.5 (s), 18.1 (s); **MS** (ESI) m/z 337 [$(M - Cl)^+$, 100]; **HRMS** m/z calculated for $C_{21}H_{25}N_2O_2$ $(M - Cl)^+$ 337.1911, found 337.1901 (+2.9 ppm error).

**[IrCl(COD)(1,3-bis(4-methoxy-2,6-dimethylphenyl)imidazol-2-ylidine)] 17**

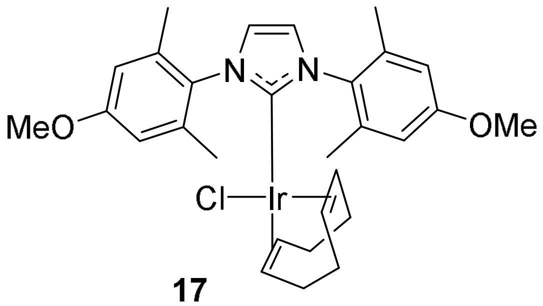


**17**

Using General Procedure C, carbene salt **S32** (22 mg, 0.059 mmol, 2.2 eq), KO$^t$Bu (7.5 mg, 0.067 mmol, 2.5 eq.), $[IrCl(COD)]_2$ (18 mg, 0.027 mmol, 1.0 eq.) in THF (5 mL) gave complex **17** (15 mg, 40%) as a yellow crystalline solid, **$^{1}$H NMR** (400 MHz, $CDCl_3$) $\delta$ 6.98

(s, 2H), 6.75 (s, 2H), 6.73 (s, 2H), 4.21-4.16 (m, 2H), 3.88 (s, 6H), 3.05-3.00 (m, 2H), 2.40 (s, 6H), 2.20 (s, 6H), 1.80-1.69 (m, 4H), 1.41-1.25 (m, 4H); **$^{13}$C NMR** (100.6 MHz, $CDCl_3$) $\delta$ 181.5 (s), 159.3 (s), 139.0 (s), 136.0 (s), 131.7 (s), 123.5 (s), 113.2 (s), 113.0 (s), 82.6 (s), 54.4 (s), 51.4 (s), 33.6 (s), 29.0 (s), 20.2 (s), 18.6 (s); **MS** (ESI) m/z 637 [$(M - Cl)^+$, 100]; **HRMS** m/z calculated for $C_{29}H_{36}{}^{193}IrN_2O_2$ $(M - Cl)^+$ 637.2402, found 637.2393 (+4.8 ppm error).

## <u>Synthesis of 18</u>

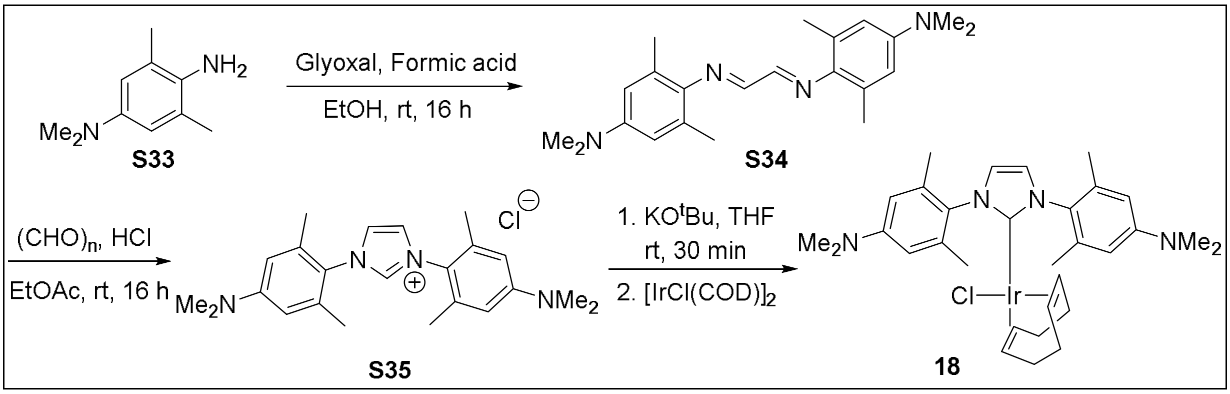


Supplementary Figure 16: Synthesis of **18**.

### 1-*N*,1-*N*,3,5-tetramethylbenzene-1,4-diamine S33

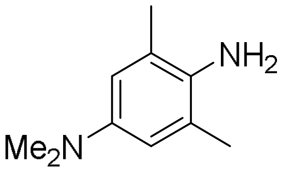


**S33**

5-Fluoro-2,3-dimethyl-2-nitrobenzene (1.00 g, 5.91 mmol, 1.0 eq.) was added to a stirred suspension of dimethylamine (14.78 mL of a 2.0 M solution in THF, 29.55 mmol, 5.0 eq.) and $K_2CO_3$ (1.63 g, 11.82 mmol, 2.0 eq.) in a 20 mL microwave vial. The vial was sealed and heated at 120 °C for 18 h. After cooling to rt, water (20 mL) was added and the mixture was extracted with EtOAc (3 x 30 mL). The combined organic layers were dried ($MgSO_4$) and concentrated under reduced pressure to give the crude product. This residue was dissolved in EtOH (15 mL) and zinc powder (1.82 g, 27.85 mmol, 5.0 eq) and AcOH (2.25 mL) were added sequentially to the reaction mixture. The resulting suspension was stirred at rt for 4 h. Then, 1 M $NaOH_{(aq)}$ (40 mL) was added and the mixture was extracted with hexane (5 x 20 mL). The combined organic layers were dried ($MgSO_4$) and concentrated under reduced pressure to give the crude product. Purification by flash column chromatography on silica with 1:1-1:9 hexane-EtOAc gave aniline **S33** (901 mg, 93%), **$^1$H NMR** (400 MHz, $CDCl_3$) $\delta$ 6.55 (s, 2H), 2.85 (s, 6H), 2.22 (s, 6H); **$^{13}$C NMR** (100.6 MHz, $CDCl_3$) $\delta$ 144.3, 134.8, 123.1, 115.2, 42.4, 18.2; **MS** (ESI) m/z 165 [$(M + H)^+$, 100]; **HRMS** m/z calculated for $C_{10}H_{17}N_2$ $(M + H)^+$ 165.1386, found 165.1381 (+3.2 ppm error).

**4-*N*-[2-{[4-(dimethylamino)-2,6-dimethylphenyl]imino}ethylidine]-1-*N*,1-*N*,3,5-tetramethylbenzene-1,4-diamine S34**

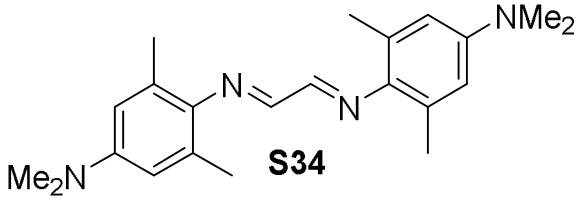


Using General Procedure A, aniline **S33** (350 g, 2.13 mmol, 2.0 eq.), glyoxal (125 µL of a 40% solution in $H_2O$, 1.07 mmol, 1.0 eq.), formic acid (2 drops) in EtOH (10 mL) gave ethylenediimine **S34** (361 mg, 96%) as a yellow powder, **$^1$H NMR** (400 MHz, $CDCl_3$) $\delta$ 8.13 (s, 2H), 6.52 (s, 4H), 2.97 (s, 12 H), 2.26 (s, 12H); **$^{13}$C NMR** (100.6 MHz, $CDCl_3$) $\delta$ 162.5 (s), 148.2 (s), 140.3 (s), 128.9 (s), 112.8 (s), 40.9 (s), 19.2 (s); **MS** (ESI) m/z 351 [$(M + H)^+$, 100]; **HRMS** m/z calculated for $C_{22}H_{31}N_4$ $(M + H)^+$ 351.2543, found 351.2532 (+2.5 ppm error).

**1,3-Bis-[4-(dimethylamine)-2,6-dimethylphenyl]imidazolium chloride S35**

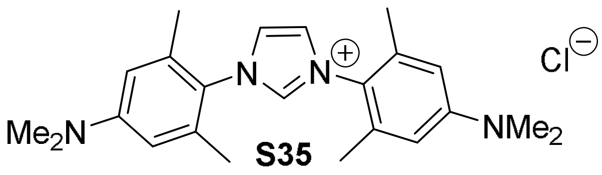


Using General Procedure B, ethylenediimine **S34** (90 mg, 0.26 mmol, 1.0 eq), paraformaldehyde (9 mg, 0.29 mmol, 1.1 eq.), HCl (0.10 mL of a 4 M solution in 1,4-dioxane, 0.40 mmol, 1.5 eq.) in EtOAc (10 mL) gave carbene salt **S35** (90 mg, 93%) as a white powder, **$^1$H NMR** (400 MHz, $CD_2Cl_2$) 10.4 (s, 1H), 7.50 (s, 2H), 6.48 (s, 4H), 2.98 (s, 12H), 2.13 (s, 12H); **$^{13}$C NMR** (101 MHz, $CD_2Cl_2$) 151.9 (s), 140.5 (s), 135.3 (s), 125.1 (s), 122.3 (s), 111.8 (s), 40.4 (s), 18.3 (s); **MS** (ESI) m/z 363 [$(M - Cl)^+$, 100]; **HRMS** m/z calculated for $C_{23}H_{31}N_4$ $(M - Cl)^+$ 363.2543, found 363.2533 (+3.5 ppm error).

**[IrCl(COD)(1,3-bis{4-(dimethylamino)-2,6-dimethylphenyl}imidazol-2-ylidine)] 18**

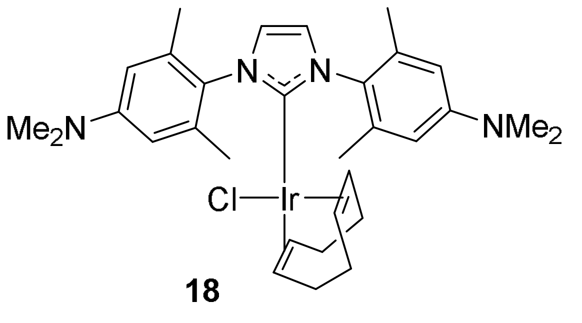


Using General Procedure C, carbene salt **S35** (90 mg, 0.23 mmol, 2.0 eq), KO$^t$Bu (30 mg, 0.26 mmol, 2.5 eq.), $[IrCl(COD)]_2$ (80 mg, 0.12 mmol, 1.0 eq.) in THF (10 mL) gave complex **18** (152 mg, 91%) as a yellow crystalline solid, **$^1$H NMR** (400 MHz, $CDCl_3$) $\delta$ 6.84

(s, 2H), 6.45 (s, 2H), 6.41 (s, 2H), 4.10-4.03 (m, 2H), 3.01-2.97 (m, 2H), 2.92 (s, 12H), 2.28 (s, 6H), 2.08 (s, 6H), 1.72-1.59 (m, 4H), 1.30-1.15 (m, 4H); $^{13}$**C NMR** (100.6 MHz, $CDCl_3$) $\delta$ 181.5 (s), 150.4 (s), 138.0 (s), 135.1 (s), 128.4 (s),123.6 (s), 112.2 (s), 110.8 (s), 81.7 (s), 51.2 (s), 40.6 (s), 33.7 (s), 29.0 (s), 20.2 (s), 18.7 (s); **MS** (ESI) m/z 663 [(M − H)$^+$, 100]; **HRMS** m/z calculated for $C_{29}H_{36}{}^{193}IrN_2O_2$ (M + H)$^+$ 637.2402, found 637.2393 (+3.7 ppm error).

**Synthesis of 19**

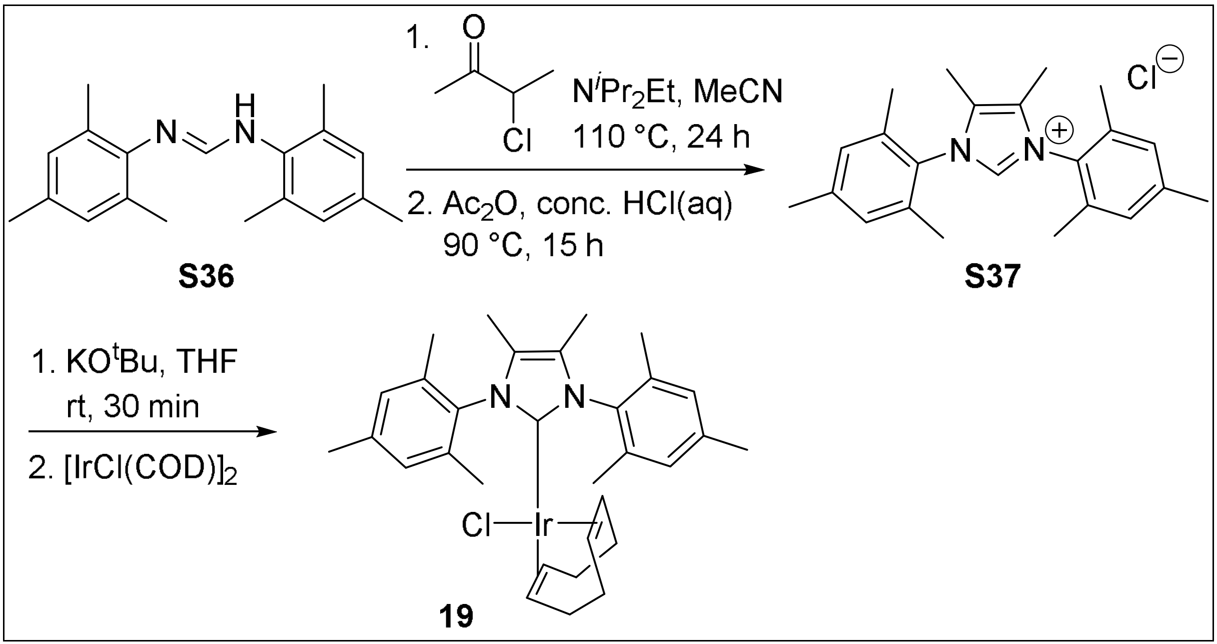


Supplementary Figure 17: Synthesis of **19**.

**1,3-Bis-(2,4,6-trimethylphenyl)-4,5-dimethylimidazolium chloride S37**

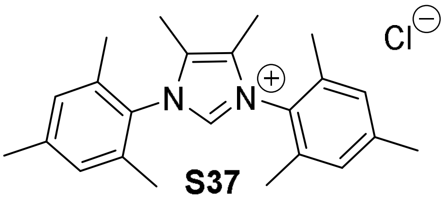

**S37**

$NiPr_2Et$ (1.50 mL, 8.57 mmol, 1.2 eq.) and 3-chloro-2-butanone (1.49 mL, 14.28 mmol, 2.0 eq.) were added sequentially to a stirred suspension of *N,N'*-dimesitylformamidine **S36** (2.00 g, 7.14 mmol, in acetonitrile (15 mL) at rt. The resulting suspension was heated at 110 °C for 24 h. Then, the reaction was concentrated under reduced pressure and toluene (20 mL), 37% HCl(aq) (0.86 mL) and acetic anhydride (1.98 mL, 2.38 mmol, 3.0 eq.) were added. The reaction mixture was heated at 90 °C for 15 h. After cooling to rt, water (20 mL) was added and the solution stirred for 10 min at rt. The two layers were separated and the aqueous layer was extracted with $CH_2Cl_2$ (3 x 25 mL). The combined organic layers were washed with 1 M $HCl_{(aq)}$, dried (MgSO4) and concentrated under reduced pressure to give the crude product. Recrystallization from $CH_2Cl_2$-EtOAc gave carbene salt **S37** (1.30 g, 49%) as a white powder, $^1$**H NMR** (400 MHz, $CDCl_3$) $\delta$ 10.18 (s, 1H), 7.01 (s, 4H), 2.31 (s, 6H), 2.06 (s, 12H), 2.04 (s, 6H); $^{13}$**C NMR** (100.6 MHz, $CDCl_3$) $\delta$ 141.3 (s), 136.9 (s), 134.6 (s), 129.9 (s),

128.8 (s), 127.7 (s), 21.2 (s), 17.5 (s), 8.6 (s); **MS** (ESI) m/z 333 [(M − Cl)$^+$, 100]; **HRMS** m/z calculated for $C_{19}H_{21}N_2$ (M − Cl)$^+$ 333.2325, found 333.2319 (+1.2 ppm error). Spectroscopic data consistent with those reported in the literature.[9]

**[IrCl(COD)(1,3-bis(2,4,6-trimethylphenyl)-4,5-dimethylimidazol-2-ylidine)] 19**

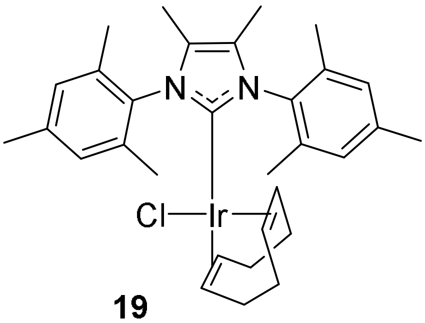


**19**

Using General Procedure C, carbene salt **S37** (200 mg, 0.54 mmol, 2.0 eq), KO$^t$Bu (76 mg, 0.68 mmol, 2.5 eq.), $[IrCl(COD)]_2$ (181 mg, 0.27 mmol, 1.0 eq.) in THF (10 mL) gave complex **19** (241 mg, 67%) as a yellow crystalline solid, **$^1$H NMR** (400 MHz, $CDCl_3$) $\delta$ 7.03 (s, 2H), 6.99 (s, 2H), 4.08-4.02 (m, 2H), 3.08-3.03 (m, 2H), 2.38 (s, 6H), 2.31 (s, 6H), 2.06 (s, 6H), 1.84 (s, 6H), 1.68-1.59 (m, 4H), 1.32-1.19 (m, 4H); **$^{13}$C NMR** (100.6 MHz, $CDCl_3$) $\delta$ 178.7 (s), 138.4 (s), 137.7 (s), 134.8 (s), 134.2 (s), 129.6 (s), 128.0 (s), 125.8 (s), 81.1 (s), 50.8 (s), 33.5 (s), 28.9 (s), 21.2 (s), 19.7 (s), 18.2 (s), 9.2 (s); **MS** (ESI) m/z 633 [(M − Cl)$^+$, 100]; **HRMS** m/z calculated for $C_{31}H_{40}{}^{193}IrN_2$ (M − Cl)$^+$ 633.2815, found 633.2819 (+0.2 ppm error).

__Synthesis of 20__

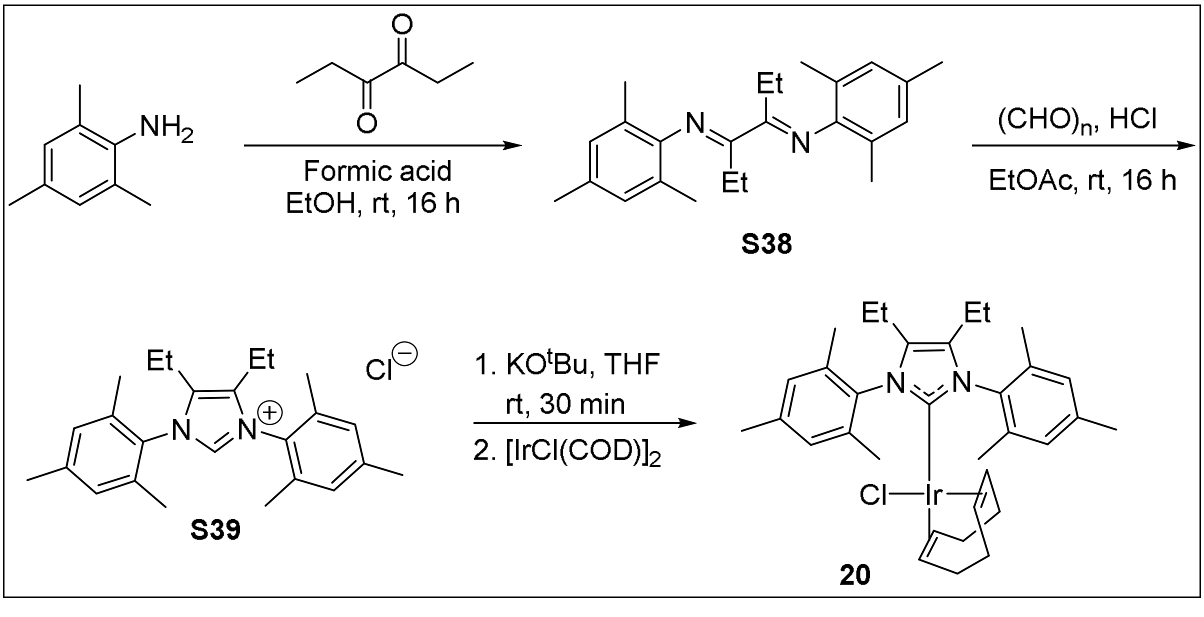


Supplementary Figure 18: Synthesis of **20**.

**2,4,6-trimethyl-N-[4-[(2,4,6-trimethylphenyl)imino]hexan-3-ylidene]aniline S38**

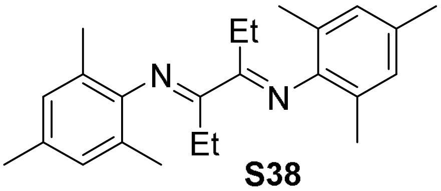


**S38**

Formic acid (2 drops) was added to a stirred solution of 2,4,6-trimethylaniline (2.80 mL, 20.0 mmol, 2.0 eq.) and 3,4-hexanedione (1.22 mL, 10.0 mmol, 1.0 eq.) in MeOH (20 mL) at rt. The resulting solution was heated to 70 °C for 16 h before cooling to rt. The reaction mixture was concentrated under reduced pressure to give the crude product. Recrystallization from $^{i}$PrOH gave diimine **S38** (2.62, 75%) as a yellow powder, $^{1}$**H NMR** (400 MHz, $CDCl_3$) $\delta$ 6.94 (s, 4H), 2.58 (q, J = 7.4 Hz, 4H), 2.34 (s, 6H), 2.09 (s, 12H), 1.09 (t, *J* = 7.4 Hz, 6H); $^{13}$**C NMR** (100.6 MHz, $CDCl_3$) $\delta$ 172.0 (s), 145.8 (s), 132.3 (s), 128.7 (s), 124.6 (s), 22.4 (s), 20.8 (s), 18.2 (s), 11.2 (s); **MS** (ESI) m/z 371 [$(M + Na)^+$, 60], 349 [$(M + H)^+$, 100]; **HRMS** m/z calculated for $C_{24}H_{33}N_2$ $(M + H)^+$ 349.2644, found 349.2653 (+1.0 ppm error).

**1,3-Bis-(2,4,6-trimethylphenyl)-4,5-diethylimidazolium chloride S39**

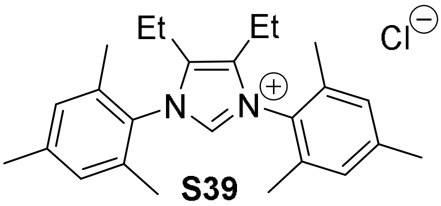


**S39**

Using General Procedure B, diimine **S38** (400 mg, 1.15 mmol, 1.0 eq), paraformaldehyde (41 mg, 1.38 mmol, 1.2 eq.), HCl (0.43 mL of a 4 M solution in 1,4-dioxane, 1.73 mmol, 1.5 eq.) in EtOAc (10 mL) gave carbene salt **S39** (309 mg, 68%) as a pale yellow powder, $^{1}$**H NMR** (400 MHz, $CDCl_3$) $\delta$ 10.55 (s, 1H), 7.06 (s, 4H), 2.50 (q, *J* = 8.0 Hz, 4H), 2.36 (s, 6H), 2.15 (s, 12H), 1.06 (t, *J* = 8.0 Hz, 6H); $^{13}$**C NMR** (100.6 MHz, $CDCl_3$) $\delta$ 141.3 (s), 135.2 (s), 134.7 (s), 132.5 (s), 130.0 (s), 128.8 (s), 21.2 (s) 17.7 (s), 16.6 (s), 13.5 (s); **MS** (ESI) m/z 361 [$(M - Cl)^+$, 100]; **HRMS** m/z calculated for $C_{25}H_{33}N_2$ $(M - Cl)^+$ 361.2638, found 361.2642 (−1.2 ppm error).

**[IrCl(COD)(1,3-bis(2,4,6-trimethylphenyl)-4,5-diethylimidazol-2-ylidine)] 20**

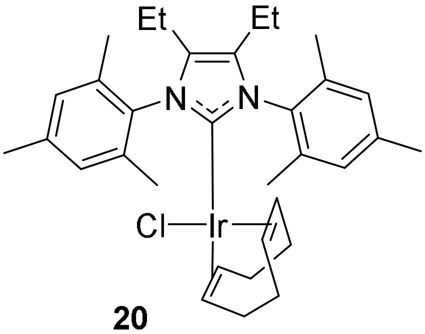


**20**

Using General Procedure C, carbene salt **S39** (184 mg, 0.46 mmol, 2.0 eq), KO$^t$Bu (64 mg, 0.58 mmol, 2.5 eq.), $[IrCl(COD)]_2$ (154 mg, 0.23 mmol, 1.0 eq.) in THF (10 mL) gave complex **20** (203 mg, 63%) as a yellow crystalline solid, **$^1$H NMR** (400 MHz, $CDCl_3$) $\delta$ 7.03 (s, 2H), 6.98 (s, 2H), 4.08-4.02 (m, 2H), 3.00-2.94 (m, 2H), 2.38 (s, 6H), 2.34 (s, 6H), 2.24 (q, $J$ = 8 Hz, 4H), 2.08 (s, 6H), 1.71-1.60 (m, 4H), 1.31-1.17 (m, 4H), 0.95 (t, $J$ = 8 Hz, 6H); **$^{13}$C NMR** (100.6 MHz, $CDCl_3$) $\delta$ 178.5 (s), 138.3 (s), 137.7 (s), 135.0 (s), 134.2 (s), 131.7 (s), 129.6 (s), 128.1 (s), 81.0 (s), 50.9 (s), 33.6 (s), 28.9 (s), 21.8 (s), 20.1 (s), 18.5 (s), 17.1 (s), 14.0 (s); **MS** (ESI) m/z 661 [(M − Cl)$^+$, 100]; **HRMS** m/z calculated for $C_{33}H_{44}IrN_2$ (M − Cl)$^+$ 661.3128, found 661.3130 (−0.1 ppm error).

**<u>Synthesis of 21</u>**

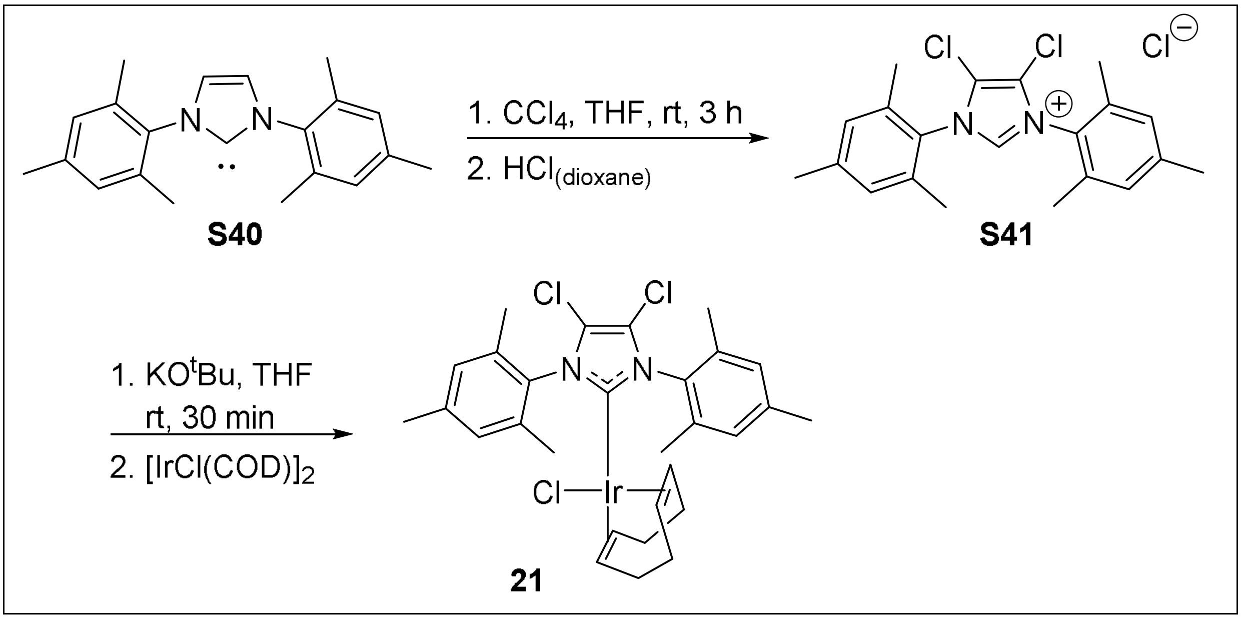


Supplementary Figure 19: Synthesis of **21**.

**1,3-Bis-(2,4,6-trimethylphenyl)-4,5-dichloroimidazolium chloride S41**

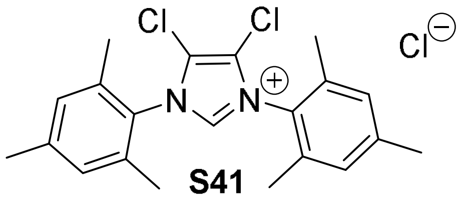


$CCl_4$ (195 µL, 2.00 mmol, 2.0 eq.) was added to a stirred solution of 1,3-bis(2,4,6-trimethylphenyl)-1,3-dihydro-2*H*-imidazol-2-ylidene **S40** (304 mg, 1.00 mmol, 1.0 eq.) in THF (10 mL) at rt under $N_{2(g)}$. The resulting solution was stirred at rt for 3 h and then HCl (0.5 mL of a 4 M solution in 1,4-dioxane, 2.00 mmol. 2.0 eq.) was added dropwise. The mixture was stirred for 30 min which resulting in the formation of a white precipitate. The precipitate was collected by vacuum filtration and washed with $Et_2O$ (3 x 10 mL) to give carbene salt **S41** (383 mg, 93%) as a white powder, **$^1$H NMR** (400 MHz, $(CD_3)_2SO$) $\delta$ 10.11 (s, 1H), 7.27 (s, 4H), 2.38 (s, 6H), 2.17 (s, 12H); **$^{13}$C NMR** (100.6 MHz, $(CD_3)_2SO$) $\delta$ 142.5

(s), 139.0 (s), 135.9 (s), 130.2 (s), 128.4 (s), 121.2 (s), 21.2 (s), 17.4 (s); **MS** (ESI) m/z 373 [(M($^{35}Cl_2$) − Cl)$^+$, 100], 375 [(M($^{35}Cl^{37}Cl$) − Cl)$^+$, 70], 377 [(M($^{37}Cl_2$) − Cl)$^+$, 10]; **HRMS** m/z calculated for $C_{21}H_{23}{}^{35}Cl_2N_2$ (M − Cl)$^+$ 373.1233, found 373.1238 (−1.6 ppm error). Spectroscopic data consistent with those reported in the literature.[9]

**[IrCl(COD)(1,3-bis(2,4,6-trimethylphenyl)-4,5-dichloroimidazol-2-ylidine)] 21**

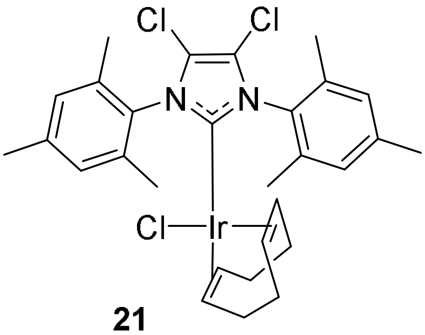


**21**

Using General Procedure C, carbene salt **S41** (100 mg, 0.24 mmol, 2.0 eq), KO$^t$Bu (32 mg, 0.29 mmol, 2.4 eq.), $[IrCl(COD)]_2$ (82 mg, 0.12 mmol, 1.0 eq.) in THF (10 mL) gave complex **21** (126 mg, 74%) as a yellow crystalline solid, **$^1$H NMR** (400 MHz, $CDCl_3$) $\delta$ 7.08 (s, 2H), 7.04 (s, 2H), 4.24-4.19 (m, 2H), 3.06-3.01 (m, 2H), 2.41 (s, 6H), 2.38 (s, 6H), 2.14 (s, 6H), 1.73-1.60 (m, 4H), 1.42-1.26 (m, 4H); **$^{13}$C NMR** (100.6 MHz, $CDCl_3$) $\delta$ 183.0 (s), 139.7 (s), 138.1 (s), 135.2 (s), 132.8 (s), 129.8 (s), 128.2 (s), 118.0 (s), 83.8 (s), 51.9 (s), 33.4 (s), 28.9 (s), 21.2 (s), 19.6 (s), 18.3 (s); **MS** (ESI) m/z 673 [(M − Cl)$^+$, 100]; **HRMS** m/z calculated for $C_{29}H_{34}Cl_2{}^{193}IrN_2$ (M − Cl)$^+$ 673.1723, found 673.1717 (−1.2 ppm error).

**Synthesis of 22**

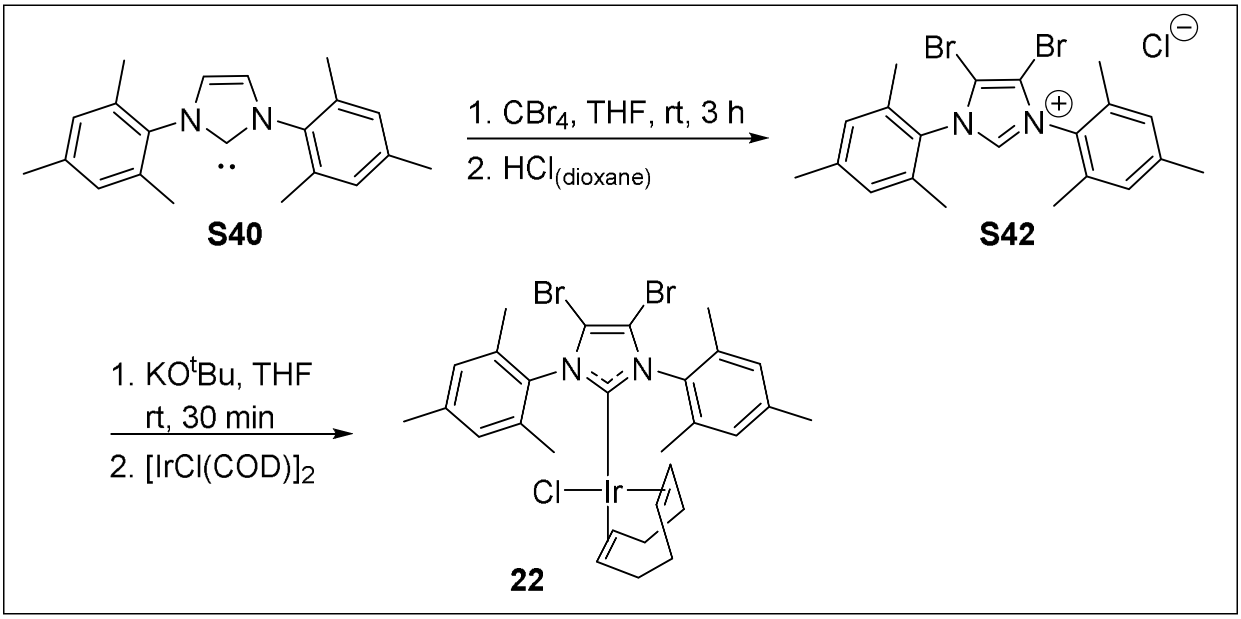


Supplementary Figure 20: Synthesis of **22**.

**1,3-Bis-(2,4,6-trimethylphenyl)-4,5-dibromoimidazolium chloride S40**

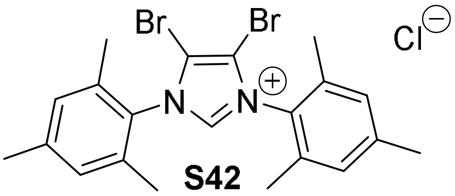


**S42**

$CBr_4$ (663 mg, 2.00 mmol, 2.0 eq.) was added to a stirred solution of 1,3-bis(2,4,6-trimethylphenyl)-1,3-dihydro-2H-imidazol-2-ylidene **S40** (304 mg, 1.00 mmol, 1.0 eq.) in THF (10 mL) at rt under $N_{2(g)}$. The resulting solution was stirred at rt for 3 h and then HCl (0.5 mL of a 4 M solution in 1,4-dioxane, 2.00 mmol. 2.0 eq.) was added dropwise. The mixture was stirred for 30 min which resulting in the formation of a white precipitate. The precipitate was collected by vacuum filtration and washed with $Et_2O$ (3 x 10 mL) to give carbene salt **S42** (303 mg, 61%) as a white powder, **$^1$H NMR** (400 MHz, $(CD_3)_2SO$) $\delta$ 10.13 (s, 1H), 7.26 (s, 4H), 2.38 (s, 6H), 2.12 (s, 12H); **$^{13}$C NMR** (100.6 MHz, $(CD_3)_2SO$) $\delta$ 142.3 (s), 141.0 (s), 135.6 (s), 130.1 (s), 129.8 (s), 113.6 (s), 21.2 (s), 17.4 (s); **MS** (ESI) m/z 461 $[(M - Cl)^+$, 50], 463 $[(M - Cl)^+$, 100], 465 $[(M - Cl)^+$, 50]; **HRMS** m/z calculated for $C_{21}H_{23}{}^{79}Br_2N_2$ $(M - Cl)^+$ 461.0222, found 461.0233 (−2.6 ppm error). Spectroscopic data consistent with those reported in the literature.[9]

**[IrCl(COD)(1,3-bis(2,4,6-trimethylphenyl)-4,5-dibromoimidazol-2-ylidine)] 22**

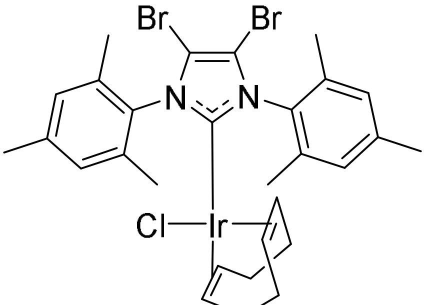


**22**

Using General Procedure C, carbene salt **S42** (50 mg, 0.12 mmol, 2.0 eq), KO$^t$Bu (17 mg, 0.15 mmol, 2.4 eq.), $[IrCl(COD)]_2$ (40 mg, 0.06 mmol, 1.0 eq.) in THF (10 mL) gave complex **22** (50 mg, 52%) as a yellow crystalline solid, **$^1$H NMR** (400 MHz, $CDCl_3$) $\delta$ 7.08 (s, 2H), 7.04 (s, 2H), 4.24-4.18 (m, 2H), 3.09-3.04 (m, 2H), 2.42 (s, 6H), 2.38 (s, 6H), 2.12 (s, 6H), 1.71-1.62 (m, 4H), 1.39-1.265(m, 4H); **$^{13}$C NMR** (100.6 MHz, $CDCl_3$) $\delta$ 185.1 (s), 139.6 (s), 137.9 (s), 135.1 (s), 134.2 (s), 129.8 (s), 128.1 (s), 108.9 (s), 83.5 (s), 51.8 (s), 33.4 (s), 28.9 (s), 21.3 (s), 19.7 (s), 18.4 (s); **HRMS** m/z calculated for $C_{29}H_{34}{}^{79}Br_2{}^{191}IrN_2$ $(M - Cl)^+$ 761.0713, found 761.0701 (−2.1 ppm error).

## Synthesis of $d_{18}$-3

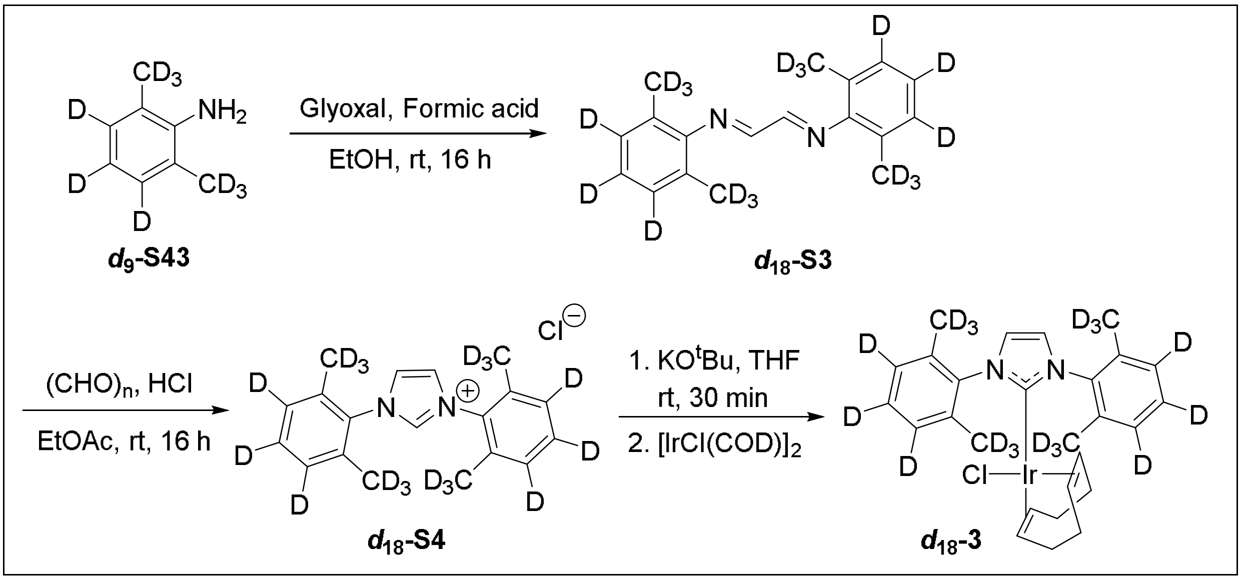


Supplementary Figure 21: Synthesis of ***d*₁₈-3**.

### N-[2-[(2,6-bis($d_3$-methyl)-3,4,5-$d_3$-phenyl)imino]ethylidine]-2,6-bis($d_3$-methyl)-3,4,5-$d_3$-aniline $d_{18}$-S3

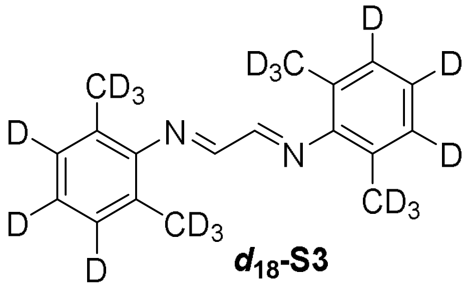

$d_{18}$-S3

Using General Procedure A, dimethylaniline **$d_9$-S43** (1.00 g, 7.69 mmol, 2.0 eq.), glyoxal (450 µL of a 40% solution in $H_2O$, 3.85 mmol, 1.0 eq.), formic acid (2 drops) in EtOH (20 mL) gave ethylenediimine **$d_{18}$-S3** (1.02 g, 94%) as a yellow powder,**$^1$H NMR** (400 MHz, $CDCl_3$) $\delta$ 8.21 (s, 2H); **$^{13}$C NMR** (100.6 MHz, $CDCl_3$) $\delta$ 163.5 (s), 150.0 (s), 127.9 (t, $J$ = 24.1 Hz, CD), 126.2 (s), 124.3 (t, $J$ = 24.2 Hz, CD), 17.4 (sept, $J$ = 19.3 Hz, $CD_3$); **MS** (ESI) m/z 305 [(M + Na)$^+$, 100], 283 [(M + H)$^+$, 80]; **HRMS** m/z calculated for $C_{18}H_3D_{18}N_2$ (M + H)$^+$ 283.2829, found 283.2829 (−0.5 ppm error).

### 1,3-Bis-[2,6-(bis-$d_3$-methyl)-3,4,5-$d_3$-phenyl]imidazolium chloride $d_{18}$-S4

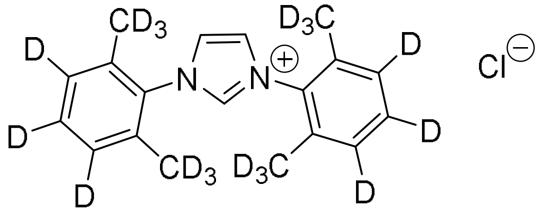

$d_{18}$-S4

Using General Procedure B, ethylenediimine ***d*₁₈-S3** (450 mg, 1.59 mmol, 1.0 eq), paraformaldehyde (53 mg, 1.76 mmol, 1.1 eq.), HCl (0.60 mL of a 4 M solution in 1,4-dioxane, 2.4 mmol, 1.5 eq.) in EtOAc (10 mL) gave carbene salt **$d_{18}$-S4** (322 mg, 61%) as a white powder, $^{1}$**H NMR** (400 MHz, $CDCl_3$) $\delta$ 10.72 (s, 1H), 7.71 (s, 2H); $^{13}$**C NMR** (100.6 MHz, $CDCl_3$) $\delta$ 138.5 (s), 134.3 (s), 133.0 (s), 130.6 (t, *J* = 26 Hz), 128.8 (t, *J* = 26 Hz), 124.7 (s), 16.9 (sept., *J* = 20 Hz); **MS** (ESI) m/z 295 [(M − Cl)$^+$, 100]; **HRMS** m/z calculated for $C_{19}H_3D_{18}N_2$ (M − Cl)$^+$ 295.2834, found 298.2839 (+1.7 ppm error).

**[IrCl(COD)(1,3-bis(2,6-bis(*d*$_3$-methyl)-3,4,5-*d*$_3$-phenyl)imidazol-2-ylidine)] *d*$_{18}$-3**

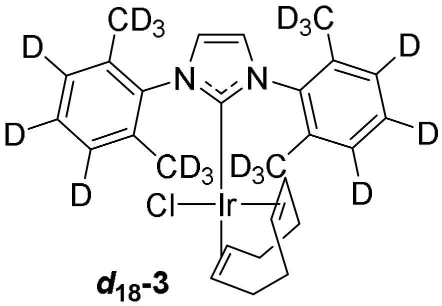


***d*$_{18}$-3**

Using General Procedure C, carbene salt **$d_{18}$-S4** (200 mg, 0.60 mmol, 2.0 eq), KO$^t$Bu (84 mg, 0.75 mmol, 2.5 eq.), $[IrCl(COD)]_2$ (201 mg, 0.30 mmol, 1.0 eq.) in THF (15 mL) gave complex **$d_{18}$-3** (339 mg, 90%) as a yellow crystalline solid, $^{1}$**H NMR** (400 MHz, $CDCl_3$) $\delta$ 7.03 (s, 2H), 4.19-4.13 (m, 2H), 3.00-2.95 (m, 2H), 1.77-1.62 (m, 4H), 1.44-1.24 (m, 4H); $^{13}$**C NMR** (100.6 MHz, $CDCl_3$) $\delta$ 180.6 (s), 138.5 (s), 137.6 (s), 134.6 (s), 128.6 (t, *J* = 25 Hz,) 128.5 (t, *J* = 26 Hz), 127.1 (t, *J* = 26 Hz), 123.2 (s), 83.9 (s), 51.5 (s), 33.5 (s), 28.9 (s), 18.9 (sept., *J* = 19 Hz), 17.5 (sept., *J* = 20 Hz); **HRMS** m/z calculated for $C_{27}H_{14}D_{18}{}^{193}IrN_2$ (M − Cl)$^+$ 595.3324, found 595.3331 (+1.2 ppm error).

**Synthesis of *d*$_{16}$-9**

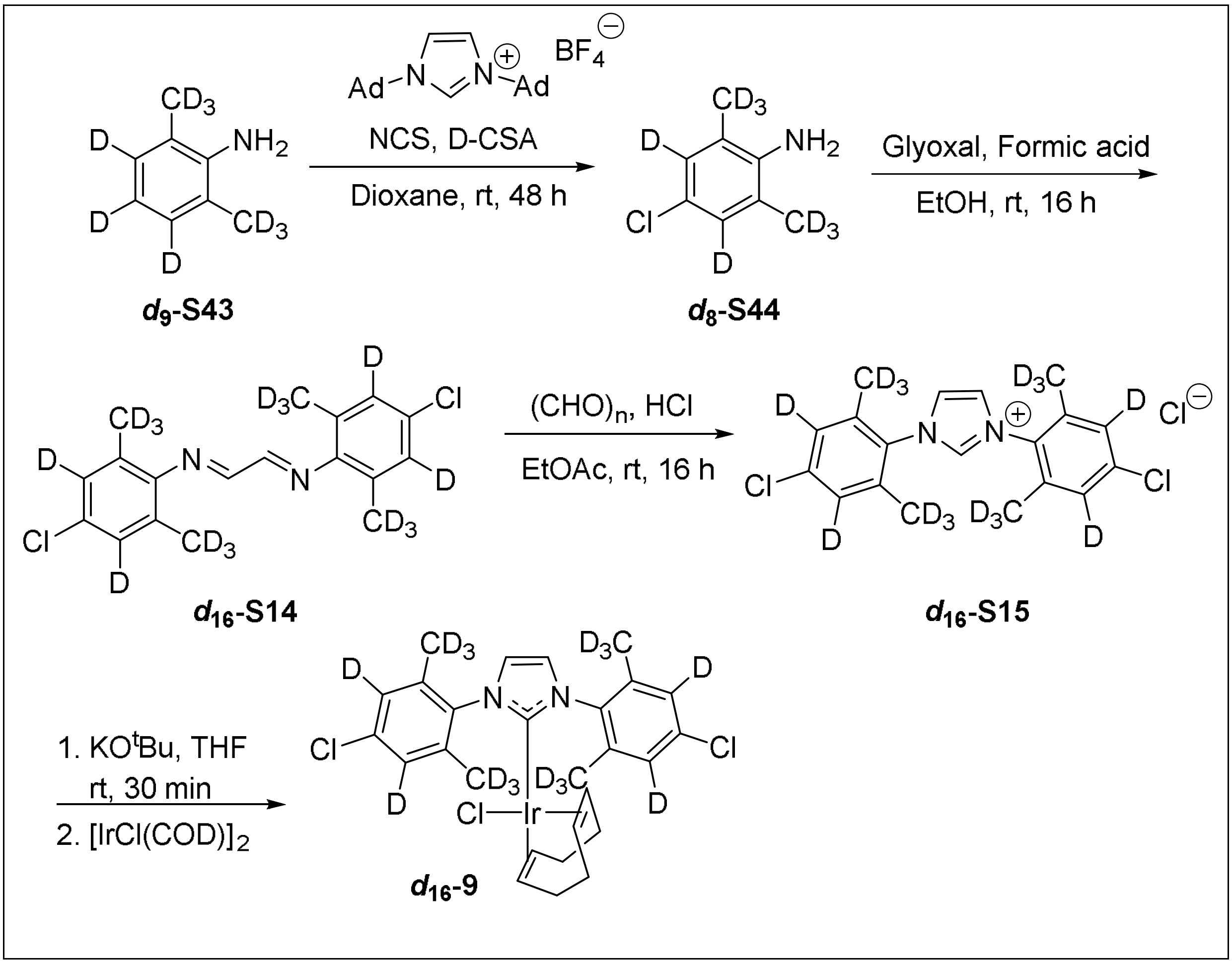


Supplementary Figure 22: Synthesis of ***d*$_{16}$-9**.

***d*$_8$-4-Chloro-2,6-dimethylaniline *d*$_8$-S44**

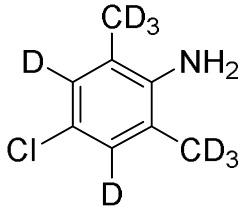


***d*$_8$-S44**

*N*-Chlorosuccinimide (1.35 g, 10.0 mmol, 1.3 eq.) was added to a stirred solution of d$_9$-dimethylaniline (1.00 g, 7.69 mmol, 1.0 eq.), D-camphor sulfonic acid (894 mg, 3.85 mmol, 0.5 eq.), 1,3-bis(1-adamantyl)imidazolium tetrafluoroborate (161 mg, 0.38 mmol, 5 mol%) in 1,4-dioxane (40 mL) at rt. The resulting solution was stirred at rt for 48 h. Then, a saturated solution of $NaHCO_{3(aq)}$ (40 mL) was added and the mixture was extracted with EtOAc (3 x 30 mL). The combined organic layers were dried ($MgSO_4$) and concentrated under reduced pressure to give the crude product. Purification by flash column chromatography with 9:1-8:2 hexane-EtOAc as eluent gave deuterated chloroaniline ***d*$_8$-S44** (1.06 g, 84%), **$^{13}$C NMR**

(100.6 MHz, $CDCl_3$) $\delta$ 141.4, 127.4 (t, $J$ = 23.7 Hz), 123.0, 122.0, 16.7 (sept., $J$ = 19.5 Hz); **MS** (ESI) m/z 163 [$(M + H)^+$, 100]; **HRMS** m/z calculated for $C_8H_3ClD_8N$ $(M + H)^+$ 163.1004, found 163.1006 (+2.3 ppm error).

**4-Chloro-*N*-(2-((4-chloro-2,6-bis-($d_3$-methyl)-3,5-$d_2$-phenyl)imino)ethylidene)-2,6-bis-$d_3$-methyl-3,5-$d_2$-aniline $d_{16}$-S14**

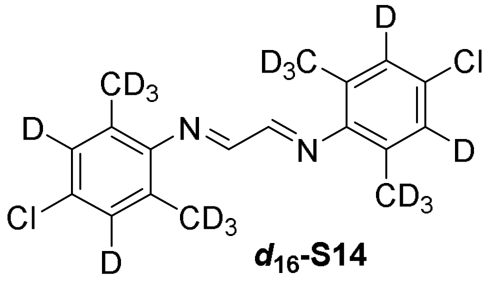


***d*$_{16}$-S14**

Using General Procedure A, aniline ***d*$_8$-S44** (350 g, 2.14 mmol, 2.0 eq.), glyoxal (125 µL of a 40% solution in $H_2O$, 1.07 mmol, 1.0 eq.), formic acid (2 drops) in EtOH (10 mL) gave ethylenediimine ***d*$_{16}$-S14** (354 mg, 95%) as a yellow powder, **$^1$H NMR** (400 MHz, $CDCl_3$) $\delta$ 8.10 (s, 2H); **$^{13}$C NMR** (100.6 MHz, $CDCl_3$) $\delta$ 163.7 (s), 148.3 (s), 129.7 (s), 128.2 (s), 127.8 (t, $J$ = 24 Hz, CD), 17.4 (sept., $J$ = 20 Hz, $CD_3$); **MS** (ESI) m/z 349 [$(M + H)^+$, 100]; **HRMS** m/z calculated for $C_{18}H_3Cl_2D_{16}N_2$ $(M + H)^+$ 349.1924, found 349.1902 (+11.2 ppm error).

**1,3-Bis-(4-chloro-2,6-bis($d_3$-methyl)-3,5-$d_2$-phenyl)imidazolium chloride $d_{16}$-S15**

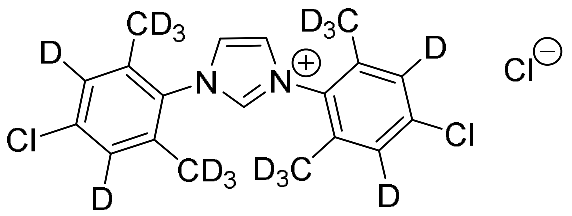


***d*$_{16}$-S15**

Using General Procedure B, ethylenediimine ***d*$_{16}$-S14** (250 mg, 0.72 mmol, 1.0 eq), paraformaldehyde (24 mg, 0.79 mmol, 1.1 eq.), HCl (0.27 mL of a 4 M solution in 1,4-dioxane, 1.08 mmol, 1.5 eq.) in EtOAc (10 mL) gave carbene salt ***d*$_{16}$-S15** (239 mg, 83%) as a white powder,**$^1$H NMR** (400 MHz, $(CD_3)_2SO$) $\delta$ 9.77 (s, 1H), 8.34 (s, 2H); **$^{13}$C NMR** (100.6 MHz, $(CD_3)_2SO$) $\delta$ 137.5 (s), 135.4 (s), 132.8 (s), 132.1 (s), 128.3 (t, $J$ = 27 Hz, CD), 125.2 (s), 17.3 (sept., $J$ = 20 Hz, $CD_3$); **MS** (ESI) m/z 361 [$(M - Cl)^+$, 100]; **HRMS** m/z calculated for $C_{19}H_3Cl_2D_{16}N_2$ $(M - Cl)^+$ 361.1924, found 361.1947 (+5.1 ppm error).

**[IrCl(COD)(1,3-bis(4-chloro-2,6-bis($d_3$-methyl)-3,5-$d_2$-phenyl)imidazol-2-ylidine)] ($d_{16}$-9)**

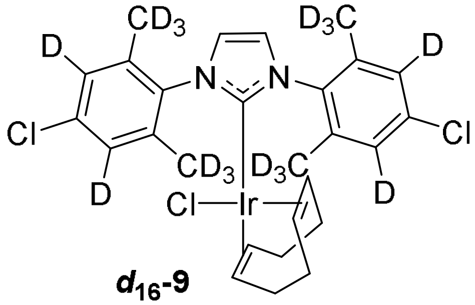


***d*₁₆-9**

Using General Procedure C, carbene salt ***d*$_{16}$-S15** (120 mg, 0.30 mmol, 2.0 eq), KO$^t$Bu (42 mg, 0.38 mmol, 2.5 eq.), [IrCl(COD)]$_2$ (100 mg, 0.15 mmol, 1.0 eq.) in THF (10 mL) gave complex ***d*$_{16}$-9** (161 mg, 77%) as a yellow crystalline solid, **$^1$H NMR** (400 MHz, CDCl$_3$) $\delta$ 7.00 (s, 2H), 4.28-4.22 (m, 2H), 2.94-2.89 (m, 2H), 1.81-1.62 (m, 4H), 1.46-1.29 (m, 4H); **$^{13}$C NMR** (100.6 MHz, CDCl$_3$) $\delta$ 181.1 (s), 141.4 (s), 139.4 (s), 137.0 (s), 136.3 (s), 134.3 (s), 127.4 (t, $J$ = 26 Hz), 123.0 (s), 122.0 (s), 84.0 (s), 51.8 (s), 33.5 (s), 28.9 (s), 16.8 (sept., $J$ = 19 Hz); **MS** (ESI) m/z 702 [(M − Cl + CH$_3$CN)$^+$, 100], 661 [(M − Cl)$^+$, 80]; **HRMS** m/z calculated for $C_{27}H_{14}Cl_2D_{16}{}^{193}IrN_2$ (M − Cl)$^+$ 661.2420, found 661.2421 (+3.8 ppm error).

**Synthesis of *d*$_{16}$-10**

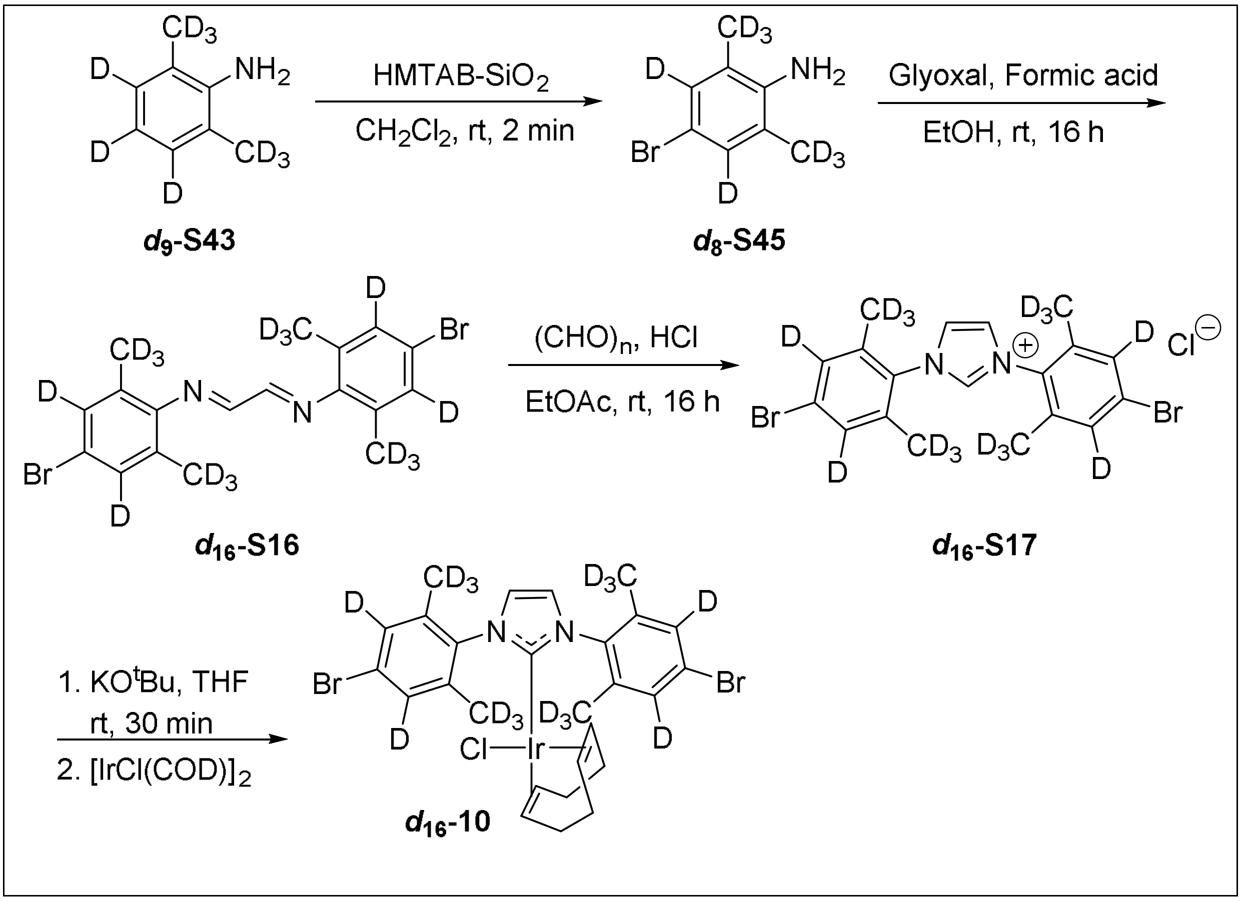


Supplementary Figure 23: Synthesis of ***d*$_{16}$-10**.

***d*8-4-Bromo-2,6-dimethylaniline *d*8-S45**

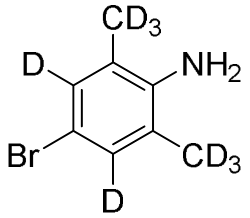

***d*8-S45**

Silica-supported-hexamethylenetetramine-bromine[10] (3.10 g, 3.85 mmol, 0.5 eq.) was added to a stirred solution of $d_9$-dimethylaniline (1.00 g, 7.69 mmol, 1.0 eq.) in $CH_2Cl_2$ (50 mL) at rt. The resulting solution was stirred at rt for 2 min. The mixture was filtered and washed with $CH_2Cl_2$ (2 x 50 mL) and the filtrate was collected, washed with 10% solution $NaHCO_3$(aq) (50 mL), dried ($MgSO_4$) and concentrated to give deuterated bromoaniline ***d*8-S45** (1.25 g, 78%) as a pale brown oil, **$^{13}$C NMR** (100.6 MHz, $CDCl_3$) $\delta$ 142.0 (s), 130.6 (t, $J$ = 25 Hz), 123.5 (s), 109.3 (s), 16.9 (sept., $J$ = 27.1 Hz); **MS** (ESI) m/z 208 [(M($^{79}$Br) + H)$^+$, 100], 210 [(M($^{81}$Br) + H)$^+$, 100]; **HRMS** m/z calculated for $C_8H_3D_8{}^{79}BrN$ (M + H)$^+$ 208.0577, found 208.0582 (+0.5 ppm error).

**4-Bromo-*N*-(2-((4-bromo-2,6-bis-(*d*3-methyl)-3,5-*d*2-phenyl)imino)ethylidene)-2,6-bis-*d*3-methyl-3,5-*d*2-aniline *d*16-S16**

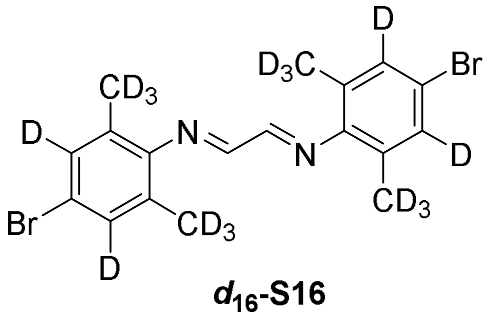

***d*16-S16**

Using General Procedure A, aniline ***d*8-S45** (1.00 g, 4.81 mmol, 2.0 eq.), glyoxal (282 µL of a 40% solution in $H_2O$, 2.41 mmol, 1.0 eq.), formic acid (2 drops) in EtOH (10 mL) gave ethylenediimine ***d*16-S16** (1.04 g, 98%) as a yellow powder, **$^1$H NMR** (400 MHz, $CDCl_3$) $\delta$ 8.09 (s, 2H); **$^{13}$C NMR** (100.6 MHz, $CDCl_3$) $\delta$ 163.6 (s), 148.8 (s), 130.7 (t, $J$ = 24.5 Hz, CD), 128.5 (s), 117.6 (s), 17.3 (sept, $J$ = 20 Hz, $CD_3$); **MS** (ESI) m/z 437 [(M($^{79}Br_2$) + H)$^+$, 50], 439 [(M($^{79}$Br$^{81}$Br) + H)$^+$, 100], 441 [(M($^{81}Br_2$) + H)$^+$, 50]; **HRMS** m/z calculated for $C_{18}H_3D_{16}{}^{79}Br_2N_2$ (M + H)$^+$ 437.0919, found 437.0928 (+2.0 ppm error).

**1,3-Bis(4-bromo-2,6-bis($d_3$-methyl)-3,5-$d_2$-phenyl)imidazolium chloride $d_{16}$-S17**

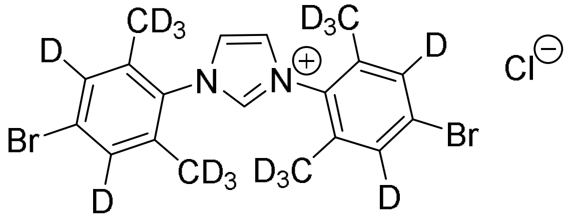


***d*₁₆-S17**

Using General Procedure B, ethylenediimine ***d*₁₆-S16** (438 mg, 1.0 mmol, 1.0 eq), paraformaldehyde (33 mg, 1.10 mmol, 1.1 eq.), HCl (0.38 mL of a 4 M solution in 1,4-dioxane, 1.50 mmol, 1.5 eq.) in EtOAc (20 mL) gave carbene salt ***d*₁₆-S17** (370 mg, 76%) as a white powder,$^{1}$**H NMR** (400 MHz, $(CD_3)_2SO$) $\delta$ 9.74 (s, 1H), 8.33 (s, 2H); $^{13}$**C NMR** (100.6 MHz, $(CD_3)_2SO$) $\delta$ 139.1 (s), 137.7 (s), 133.3 (s), 131.6 (t, $J$ = 26 Hz, CD), 125.1 (s), 124.1 (s), 16.4 (sept., $J$ = 20 Hz, $CD_3$); **MS** (ESI) m/z 449 [(M($^{79}Br_2$) − Cl)$^+$, 50], 451 [(M($^{79}Br^{81}Br$) + H)$^+$, 100], 453 [(M($^{81}Br_2$) + H)$^+$, 50]; **HRMS** m/z calculated for $C_{19}H_3D_{16}{}^{79}Br_2N_2$ (M − Cl)$^+$ 449.0919, found 449.0908 (−2.4 ppm error).

**1,3-Bis-[4-bromo-2,6-bis($d_3$-methyl)-3,5-$d_2$-phenyl]imidazolium chloride $d_{16}$-10**

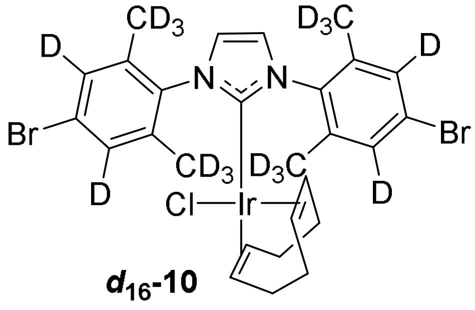


Using General Procedure C, carbene salt ***d*₁₆-S17** (120 mg, 0.26 mmol, 2.0 eq), KO$^t$Bu (37 mg, 0.33 mmol, 2.5 eq.), $[IrCl(COD)]_2$ (87 mg, 0.13 mmol, 1.0 eq.) in THF (10 mL) gave complex ***d*₁₆-10** (172mg, 84%) as a yellow crystalline solid, $^{1}$**H NMR** (400 MHz, $CDCl_3$) $\delta$ 7.0 (s, 2H), 4.30-4.24 (m, 2H), 2.95-2.88 (m, 2H), 1.84-1.67 (m, 4H), 1.49-1.40 (m, 2H), 1.38-1.29); $^{13}$**C NMR** (100.6 MHz, $CDCl_3$) $\delta$ 181.2 (s), 139.7 (s), 137.5 (s), 136.6 (s), 131.5 (t, $J$ = 21 Hz), 130.3 (t, $J$ = 21 Hz), 123.3 (s), 122.6 (s), 84.2 (s), 51.7 (s), 33.5 (s), 28.9 (s), 18.8 (sept., $J$ = 22 Hz), 17.4 (sept, $J$ = 22 Hz); **MS** (ESI) m/z 749 [(M − Cl)$^+$, 100]; **HRMS** m/z calculated for $C_{27}H_{14}D_{16}Br_2{}^{193}IrN_2$ (M − Cl)$^+$ 749.1404, found 749.1367 (+2.6 ppm error).

## Synthesis of $d_{22}$-12

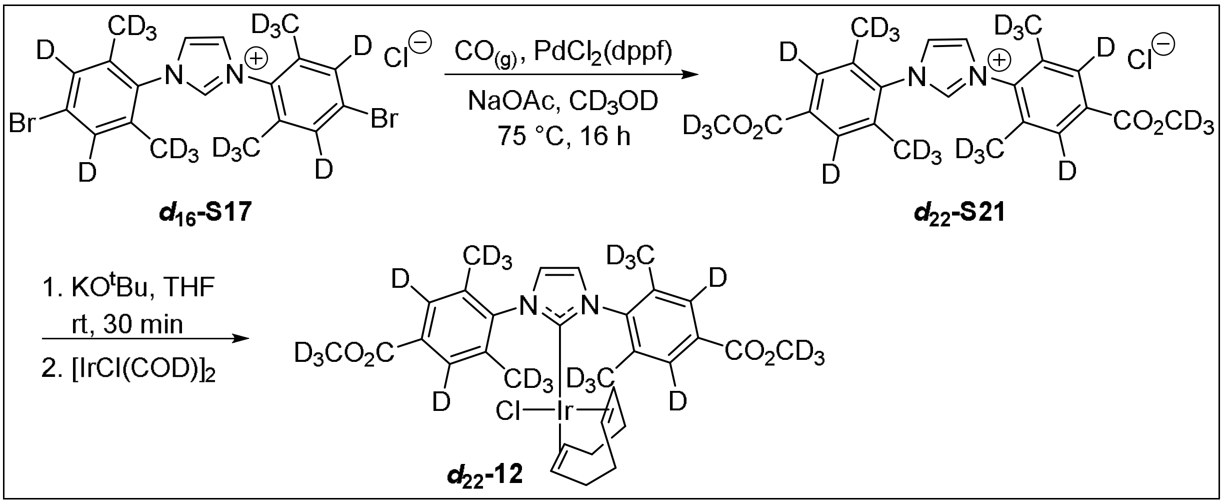


Supplementary Figure 24: Synthesis of ***d*$_{22}$-12**.

**1,3-Bis-[4-($d_3$-methoxycarbonyl)-2,6-bis-($d_3$-methyl)-3,5-$d_2$-phenyl]imidazolium chloride *d*$_{22}$-S21**

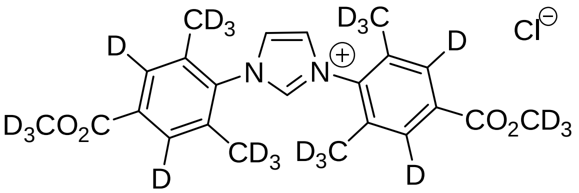

***d*$_{22}$-S21**

A solution of ***d*$_{16}$-S17** (130 mg, 0.27 mmol, 1.0 eq.), NaOAc (56 mg, 0.68 mmol, 2.5 eq.) and $PdCl_2$(dppf) (10 mg, 5 mol%) in $CD_3OD$ (10 mL) was heated to 75 °C under $CO_{(g)}$ (4 Bar) for 16 h. The reaction was allowed to cool, filtered through Celite® and concentrated under reduced pressure to give the crude product. Recrystallization from $CH_2Cl_2$-$Et_2O$ gave carbene salt ***d*$_{22}$-S21** (101 mg, 83%) as a white solid, **$^1$H NMR** (400 MHz, $CDCl_3$) $\delta$ 11.22 (s, 1H), 7.74 (s, 2H); **$^{13}$C NMR** (100.6 MHz, $CDCl_3$) $\delta$ 165.7 (s), 157.0 (s), 136.4 (s), 134.9 (s), 132.5 (s), 130.1 (t, $J$ = 27 Hz, CD), 124.3 (s), 51.9 (sept., $J$ = 24 Hz, $OCD_3$), 17.2 (sept, $J$ = 20 Hz, $CD_3$); **MS** (ESI) m/z 415 [$(M - Cl)^+$, 100]; **HRMS** m/z calculated for $C_{23}H_3D_{22}N_2O_4$ $(M - Cl)^+$ 415.3195, found 415.3193 (+0.0 ppm error).

**[IrCl(COD)(1,3-bis(4-($d_3$-methoxycarbonyl)-2,6-bis($d_3$-methyl)-3,5-$d_2$-phenyl)-4,5-dimethylimidazol-2-ylidine)] ($d_{22}$-12)**

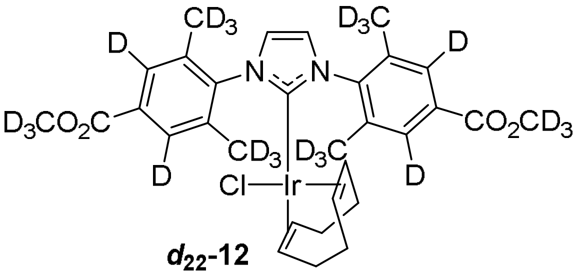

***d*$_{22}$-12**

Using General Procedure C, carbene salt ***d*₂₂-S21** (85 mg, 0.19 mmol, 2.0 eq), KO$^t$Bu (28 mg, 0.24 mmol, 2.5 eq.), $[IrCl(COD)]_2$ (63 mg, 0.094 mmol, 1.0 eq.) in THF (10 mL) gave complex ***d*$_{22}$-12** (84 mg, 59%) as a yellow crystalline solid, **$^1$H NMR** (400 MHz, $CDCl_3$) $\delta$ 7.05 (s, 2H), 4.27-4.20 (m, 2H), 2.95-2.87 (m, 2H), 1.78-1.61 (m, 4H), 1.45-1.35 (m, 2H), 1.32-1.26 (m, 2H); **$^{13}$C NMR** (100.6 MHz, $CDCl_3$) $\delta$ 180.5 (s), 166.7 (s), 142.2 (s), 138.1 (s), 135.1 (s), 130.3 (s), 129.9 (t, $J$ = 22 Hz), 128.9 (t, $J$ = 22 Hz), 123.2 (s), 84.4 (s), 52.9 (s), 51.7 (sept., $J$ = 25 Hz), 33.5 (s), 29.1 (s), 19.2 (sept., $J$ = 25 Hz), 17.7 (sept., $J$ = 25 Hz); **MS** (ESI) m/z 715 [(M − Cl)$^+$, 100]; **HRMS** m/z calculated for $C_{31}H_{14}D_{22}{}^{193}IrN_2O_4$ (M − Cl)$^+$ 715.3680, found 715.3693 (−0.7 ppm error).

## Synthesis of *d*$_{34}$-16

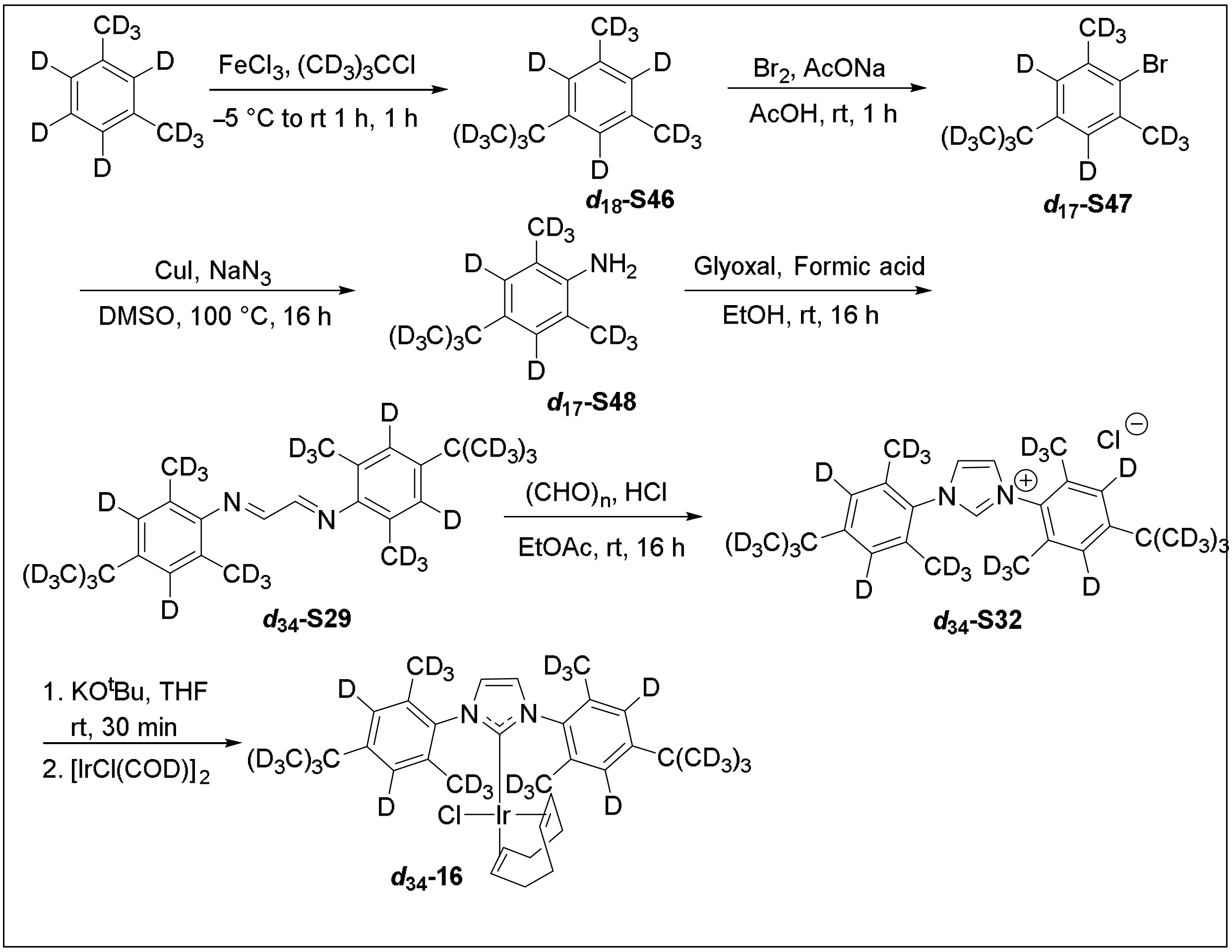


Supplementary Figure 25: Synthesis of ***d*$_{34}$-16**.

**1-($d_9$-*tert*-Butyl)-3,5-di($d_3$-methyl)-2,4,6-$d_3$-benzene $d_{18}$-S46**

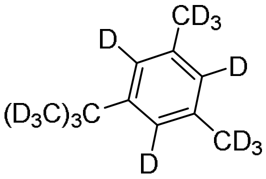


***d*18-S46**

*tert*-Butyl-$d_9$ chloride (4.37 g, 43.0 mmol, 1.0 eq.) was added dropwise to a stirred suspension of $FeCl_3$ (2.10 g, 12.9 mmol, 0.3 eq.) in *m*-xylene-$d_{10}$ (5.0 g, 43.0 mmol, 1.0 eq.) over 15 min at −5 °C. The resulting suspension was stirred at −5 °C for 15 min before being stirred at rt for 1 h. The reaction was then filtered through silica and washed with hexane. The filtrate was concentrated under reduced pressure to give 1-($d_9$-*tert*-butyl)-3,5-di($d_3$-methyl)-2,4,6-$d_3$-benzene **$d_{18}$-S46** (7.13 g, 92%) as a colourless oil which required no further purification, **$^{13}$C NMR** (125 MHz, $CDCl_3$) 156.0 (s), 137.1 (s), 126.8 (t, $J$ = 25.0 Hz), 122.9 (t, $J$ = 25.0 Hz), 33.8 (s), 30.3 (sept, $J$ = 18.0 Hz), 20.6 (sept, $J$ = 17.5 Hz).

**1-Bromo-4-($d_9$-*tert*-Butyl)-2,6-di($d_3$-methyl)-3,5-$d_2$-benzene $d_{17}$-S47**

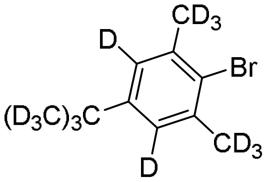


***d*17-S47**

A solution of bromine (767 µL, 14.97 mmol, 1.0 eq.) in AcOH (15 mL) was added dropwise to a stirred solution of **$d_{18}$-S48** (2.70 g, 14.97 mmol, 1.0 eq.) and NaOAc (1.80 g, 21.94 mmol, 1.45 eq) in AcOH (50 mL) over 15 min at rt. The resulting solution was stirred at rt for 1 h. Then, the volatiles were removed under reduced pressure and the residue was poured into water (30 mL). The precipitate was filtered and washed with $H_2O$ (3 x 15 mL). Recrystallization from EtOH gave 1-bromo-4-($d_9$-*tert*-butyl)-2,6-di($d_3$-methyl)-3,5-$d_2$-benzene **$d_{17}$-S47** (3.82 g, 99%) as a white crystalline solid, **$^{13}$C NMR** (125 MHz, $CDCl_3$) 149.5 (s), 137.4 (s), 125.1 (t, $J$ = 23 Hz), 124.4 (s), 41.0 (sept, $J$ = 20.5 Hz), 33.6 (s), 30.2 (sept, $J$ = 18 Hz), 23.2 (sept, $J$ = 18 Hz); Anal. Calcd for $C_{12}D_{17}Br$, C, 55.80, D, 13.26, Found: C, 55.40, D, 13.34.

**4-($d_9$-tert-Butyl)-2,6-di($d_3$-methyl)-3,5-$d_2$-aniline S48**

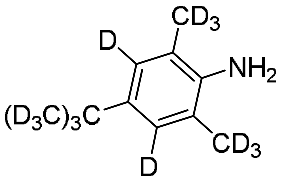


***d*17-S48**

$NaN_3$ (1.57 g, 24.16 mmol, 2.0 eq.) was added to a stirred solution of 1-bromo-4-($d_9$-*tert*-butyl)-2,6-di($d_3$-methyl)-3,5-$d_2$-benzene ***d*₁₇-S47** (3.00 g, 12.08 mmol, 1.0 eq.), CuI (2.30 g, 12.08 mmol, 1.0 eq) and DMEDA (1.69 mL, 15.70 mmol, 1.3 eq) in DMSO (25 mL) under $N_2$ at rt. The resulting suspension was heated at 100 °C for 3 h. The solution was then cooled to rt and a saturated solution of $NH_4Cl_{(aq)}$ (50 mL) and EtOAc (25 mL) were added. The two layers were separated and the aqueous layer was extracted with EtOAc (2 x 25 mL). The combined organic layers were dried ($MgSO_4$) and concentrated under reduced pressure to give the crude product. Purification by flash column chromatography on silica with hexane-EtOAc 95:5-85:15 as eluent gave 4-($d_9$-tert-butyl)-2,6-di($d_3$-methyl)-3,5-$d_2$-aniline **$d_{17}$-S48** (1.58 g, 67%) as a colourless oil, **$^1$H NMR** (500 MHz, $CDCl_3$) 3.53 (br s, 2H); **$^{13}$C NMR** (125 MHz, $CDCl_3$) 140.7 (s), 140.4 (s), 124.9 (t, $J$ = 23.6 Hz), 121.2 (s), 33.1 (s), 30.6 (sept, $J$ = 21.0 Hz), 17.1 (sept, $J$ = 21.0 Hz); **HRMS** (ESI) *m/z* $[M + H]^+$ calculated for $C_{12}H_3D_{17}N_2$ 195.2657, found 195.2661 (–2.5 ppm error).

**1,3-Bis-(4-($d_9$-*tert*-butyl)-2,6-di($d_3$-methyl)-4,5-$d_2$-phenyl)imidazolium chloride $d_{34}$-S30**

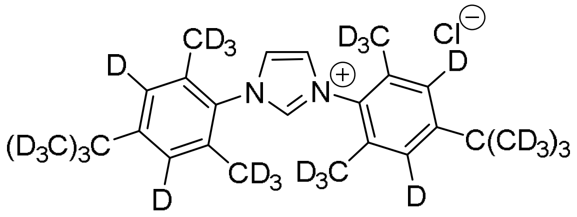


**$d_{34}$-S30**

Glyoxal (362 µL, 3.10 mmol, 1.0 eq.) and formic acid (2 drops) were added sequentially to a stirred solution of aniline **$d_{17}$-S48** (1.20 g, 6.20 mmol, 2.0 eq.) EtOH (15 mL) at rt. The resulting solution was stirred at rt for 16 h. The reaction mixture was concentrated under reduced pressure to give a residue which contained the intermediate ethylenediimine **$d_{34}$-S29**. The residue was dissolved in EtOAc (15 mL) and a solution of *para*formaldehyde (103 mg, 3.41 mmol, 1.1 eq.) in HCl (1.16 ml of a 4 M solution in dioxane, 4.65 mmol, 1.5 eq.) was added dropwise at rt. The solution was stirred at rt for 16 h, during which time a precipitate formed. The precipitate was filtered, washed with EtOAc and dried under reduced pressure to a crude product that contained carbene salt **$d_{34}$-S30** (825 mg, 58%, ca. 90% pure) as an off white powder which could be used in the next step without further purification, **$^1$H NMR** (500 MHz, $CD_2Cl_2$) $\delta$ 10.93 (t, $J$ = 1.5 Hz, 1H), 7.61 (d, $J$ = 1.5 Hz, 2H); **MS** (ESI) m/z 423 $[(M - Cl)^+$, 100]; **$^{13}$C NMR** (125 MHz, $CDCl_3$) $\delta$ 154.3 (s), 139.7 (s) 133.9 (s), 130.8 (s), 126.0 (t, $J$ = 19.4 Hz), 124.2 (s), 34.0 (s), 29.7 (sept, $J$ = 19 Hz), 17.0 (sept., $J$ = 19 Hz); **HRMS** m/z calculated for $C_{27}H_3D_{34}N_2$ $(M - Cl)^+$ 423.5085, found 423.5090 (−1.4 ppm error).

**[IrCl(COD)(1,3-bis(4-($d_9$-*tert*-butyl)-2,6-di($d_3$-methyl)-4,5-$d_2$-phenyl)imidazole-2-ylidine)] $d_{34}$-16**

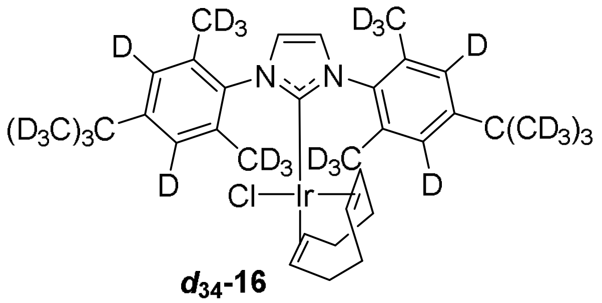

***d*34-16**

Using General Procedure C, carbene salt ***d*34-S30** (459 mg, 1.00 mmol, 2.2 eq), KO$^t$Bu (127 mg, 1.13 mmol, 2.5 eq.), [IrCl(COD)]$_2$ (302 mg, 0.45 mmol, 1.0 eq.) in THF (10 mL) gave complex ***d*34-16** (423 mg, 62%) as a yellow crystalline solid,$^1$**H NMR** (500 MHz, $CDCl_3$) $\delta$ 7.04 (s, 2H), 4.16-4.09 (m, 2H), 3.02-2.97 (m, 2H), 1.67-1.54 (m, 4H), 1.34-1.21 (m, 4H); $^{13}$**C NMR** (125 MHz, $CDCl_3$) $\delta$ 181.3 (s), 151.7 (s), 136.7 (s), 135.9 (s), 133.9 (s), 125.5 (t, $J$ = 19.4 Hz), 124.1 (t, $J$ = 23.2 Hz), 123.1 (s), 82.1 (s), 51.2 (s), 33.9 (s), 33.5 (s), 30.3 (sept, $J$ = 18.1 Hz), 28.9 (s), 19.0 (sept., $J$ = 18 Hz), 17.8 (sept., $J$ = 18 Hz); **MS** (ESI) m/z 723 [(M − Cl)$^+$, 100]; **HRMS** m/z calculated for $C_{35}H_{14}D_{34}{}^{193}IrN_2$ (M − Cl)$^+$ 723.5575, found 723.5584 (+0.4 ppm error).

## Synthesis of *d*28-18

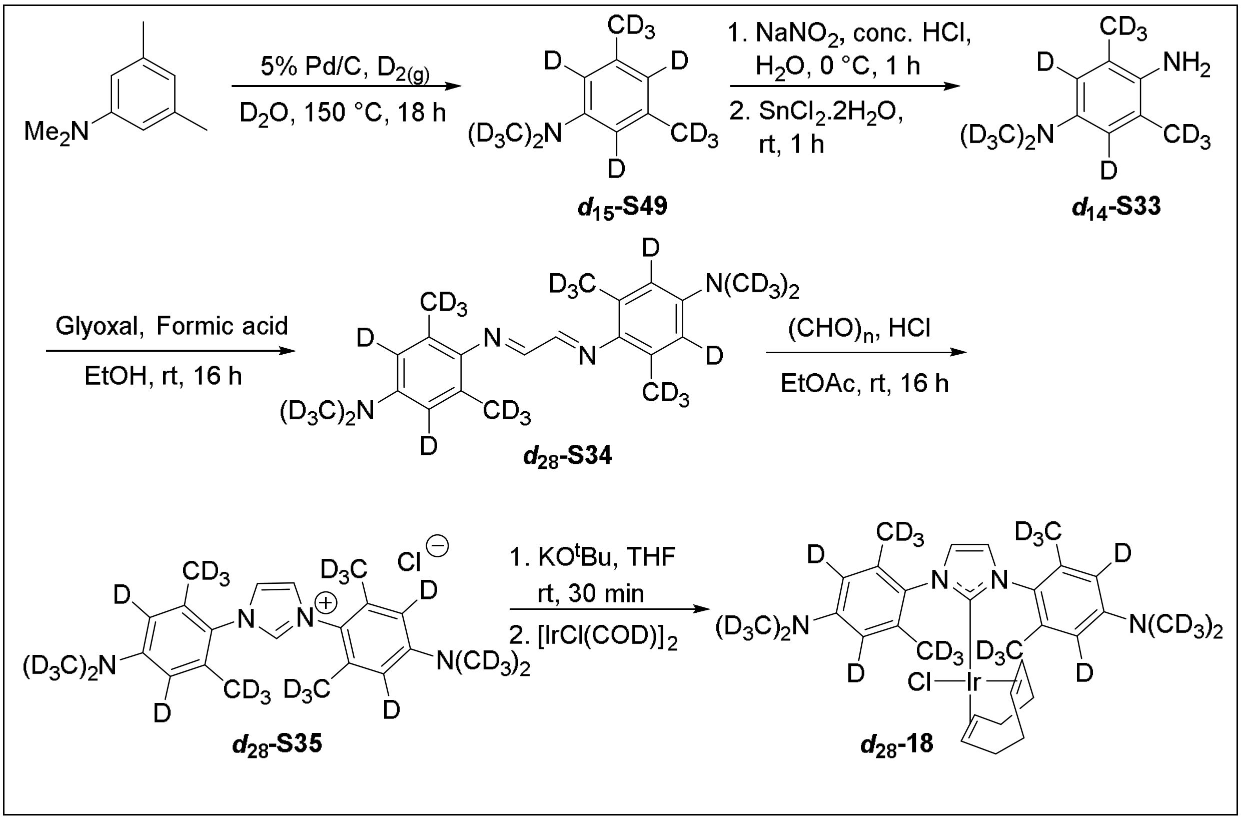


Supplementary Figure 26: Synthesis of ***d*28-18**.

***N*,*N*,3,5-tetra($d_3$-methyl)-2,4,6-$d_3$-aniline $d_{15}$-S49**

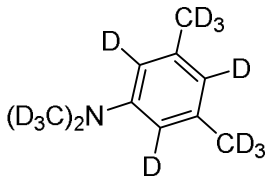


***d*15-49**

To a suspension of *N*,*N*,3,5-tetramethylaniline (5.0 mL, 31 mmol) in $D_2O$ (40 mL) in a 100 mL Parr reactor was added 5% Pd/C (500 mg, 10 wt%). The reactor was sealed, purged with $N_2$, then pressurised with $D_2$ (2 bar). The reaction mixture was stirred at 150 °C for 18 hours. The pressure was released and the mixture extracted with EtOAc (3 × 50 mL), and filtered through Celite, washing with EtOAc. The solvent was removed under reduced pressure and the residue was again subjected to analogous reaction and purification conditions as described above to give *N*,*N*,3,5-tetra($d_3$-methyl)-2,4,6-$d_3$-aniline ***d*15-S49** (2.5 g, 54%) as a light brown oil; **$^{13}$C NMR** (101 MHz, $CDCl_3$) 150.9 (s), 138.4 (s), 118.5 (t, *J* = 24.1 Hz), 110.5 (t, *J* = 23.6 Hz), 39.9 (sept, *J* = 20.2 Hz), 20.9 (sept, *J* = 19.5 Hz); **HRMS** (ESI) *m/z* $[M + H]^+$ calculated for $C_{10}HD_{15}N$ 165.2219, found 165.2219 (–0.1 ppm error).

**4-(*N*,*N*-di($d_3$-methyl))amino-2,6-di($d_3$-methyl)-3,5-$d_2$-aniline $d_{14}$-S33**

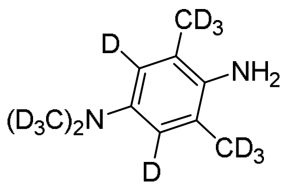


***d*14-S33**

To a solution of aniline ***d*15-S49** (2.5 g, 15 mmol) in concentrated HCl (15 mL) at 0 °C was added a solution of sodium nitrite (1.13 g, 16.4 mmol, 1.1 eq.) in $H_2O$ (3 mL) dropwise. The reaction mixture was stirred at 0 °C for 1 hour then filtered, washing with cold ethanol (3 × 5 mL) to give a yellow solid (600 mg). This residue was dissolved in ethanol (20 mL), then concentrated HCl (2 mL) and tin(II) chloride dihydrate (2.0 g, 8.8 mmol) were added. Stirred at room temperature for 1 hour then quenched by addition of aq. NaOH solution (30 mL, 1 M), extracted with $CH_2Cl_2$ (3 × 20 mL), dried over $MgSO_4$ and concentrated under reduced pressure. Purified by column chromatography with 1:1 EtOAc-petrol as eluent to give 4-(*N*,*N*-di($d_3$-methyl))amino-2,6-di($d_3$-methyl)-3,5-$d_2$-aniline ***d*14-S33** (360 mg, 13%) as a brown oil; **$^1$H NMR** (400 MHz, $CDCl_3$) 3.39 (br s, 2H); **$^{13}$C NMR** (101 MHz, $CDCl_3$) 143.8 (s), 134.6 (s), 122.5 (s), 114.5 (t, *J* = 23.6 Hz), 41.0 (sept, *J* = 20.5 Hz), 17.0 (sept, *J* = 19.2 Hz); **HRMS** (ESI) *m/z* $[M + H]^+$ calculated for $C_{10}H_3D_{14}N_2$ 179.2265, found 179.2268 (–3.0 ppm error).

**4-((*N*,*N*-bis($d_3$-methyl))amino)-N-(2-((4-((*N*,*N*-bis($d_3$-methyl))amino)-2,6-bis-($d_3$-methyl)-3,5-$d_2$-phenyl)imino)ethylidene)-2,6-bis-($d_3$-methyl)-3,5-$d_2$-aniline $d_{28}$-S34**

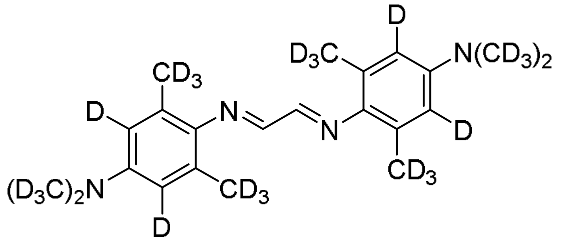


***d*₂₈-S34**

Using General Procedure A, aniline ***d*$_{14}$-S33** (300 mg, 1.69 mmol, 2.0 eq.), glyoxal (100 µL of a 40% solution in $H_2O$, 0.87 mmol, 1.0 eq.), formic acid (2 drops) in EtOH (8mL) gave ethylenediimine ***d*$_{28}$-S34** (170 mg, 53%) as a yellow solid; **$^1$H NMR** (400 MHz, $CDCl_3$) 8.16 (s, 2H); **$^{13}$C NMR** (101 MHz, $CDCl_3$) 162.5 (s), 148.2 (s), 140.4 (s), 128.8 (s), 112.5 (t, *J* = 24.4 Hz), 39.9 (sept, *J* = 20.8 Hz), 18.4 (sept, *J* = 19.3 Hz); **HRMS** (ESI) *m/z* $[M + H]^+$ calculated for $C_{22}H_3D_{28}N_4$ 379.4301, found 379.4304 (–1.2 ppm error).

**1,3-Bis-(4-((*N*,*N*-bis($d_3$-methyl))amino)-2,6-bis-($d_3$-methyl)-3,5-$d_2$-phenyl)imidazolium chloride $d_{28}$-S35**

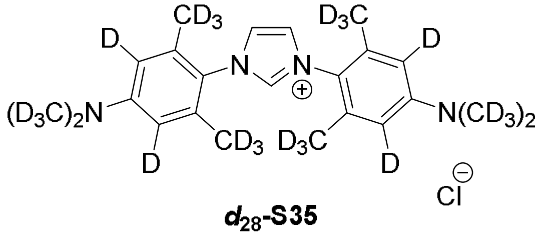


***d*$_{28}$-S35**

Using General Procedure B, ethylenediimine ***d*$_{28}$-S34** (150 mg, 0.40 mmol, 1.0 eq), paraformaldehyde (14 mg, 0.47 mmol, 1.2 eq.), HCl (0.15 mL of a 4 M solution in 1,4-dioxane, 0.60 mmol, 1.5 eq.) in EtOAc (15 mL) gave carbene salt ***d*$_{28}$-S35** (52 mg, 31%) as a light brown powder; **$^1$H NMR** (400 MHz, $CD_2Cl_2$) 9.64 (s, 1H), 7.57 (s, 2H); **$^{13}$C NMR** (101 MHz, $CD_2Cl_2$) 151.8 (s), 139.3 (s), 135.0 (s), 125.5 (s), 122.0 (s), 111.3 (t, *J* = 25.2 Hz), 39.4 (sept, *J* = 20.6 Hz), 17.3 (sept, *J* = 19.5 Hz); **HRMS** $[M – Cl]^+$ calculated for $C_{23}H_3D_{28}N_4$ 391.4301, found 391.4304 (–1.0 ppm error).

**[IrCl(COD)(1,3-bis(4-((*N*,*N*-bis($d_3$-methyl))amino))-2,6-bis($d_3$-methyl)-3,5-$d_2$-phenyl)-4,5-dimethylimidazol-2-ylidine)] $d_{28}$-18**

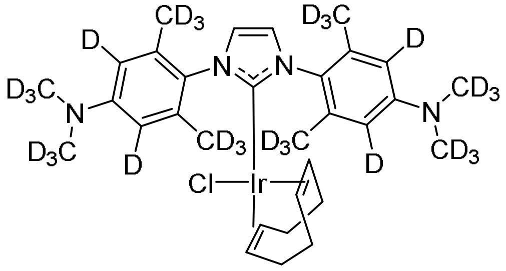


***d*$_{28}$-18**

Using General Procedure C, carbene salt ***d*$_{28}$-S35** (50 mg, 0.12 mmol, 2.2 eq), KO$^t$Bu (15 mg, 0.13 mmol, 2.4 eq.), $[IrCl(COD)]_2$ (36 mg, 0.054 mmol, 1.0 eq.) in THF (5 mL) gave complex ***d*$_{28}$-18** (32 mg, 37%) as a yellow crystalline solid, **$^1$H NMR** (400 MHz, $CDCl_3$) 6.9 (s, 2H), 4.13 (m, 2H), 3.05 (m, 2H), 1.77-1.63 (m, 4H), 1.36-1.21 (m , 4H); **$^{13}$C NMR** (101 MHz, $CDCl_3$) 181.5 (s), 150.4 (s), 137.8 (s), 135.0 (s), 128.5 (s), 123.7 (s), 111.9 (t, *J* = 22.9 Hz), 110.5 (t, *J* = 22.9 Hz), 81.7 (s), 51.3 (s), 39.8 (sept, *J* = 20.7 Hz), 33.7 (s), 29.1 (s), 19.4 (sept, *J* = 19.7 Hz), 18.0 (sept, *J* = 19.7 Hz); ); **MS** (ESI) m/z 691 [(M − Cl)$^+$, 100]; **HRMS** $[M – Cl]^+$ calculated for $C_{31}H_{14}D_{28}{}^{193}IrN_4$ 691.4791, found 691.4785 (+3.2 ppm error).

**Synthesis of $d_{22}$-19**

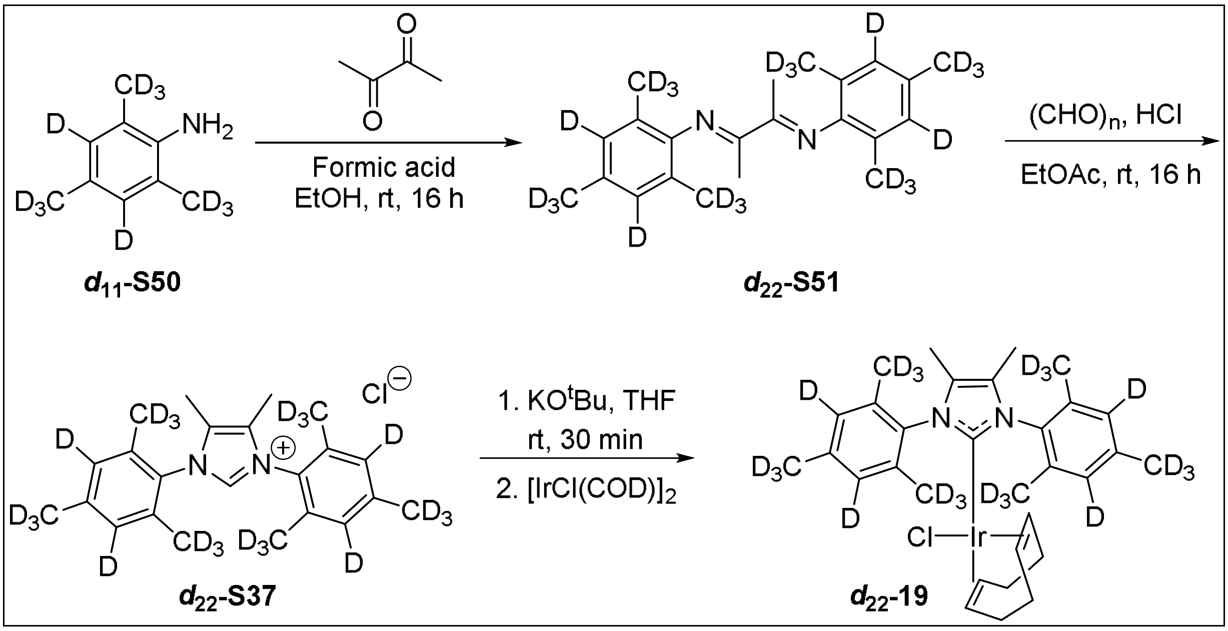


Supplementary Figure 27: Synthesis of ***d*$_{22}$-19**.

**2,4,6-tris($d_3$-methyl)-N-(3-((2,4,6-tris($d_3$-methyl)-3,5-$d_2$-phenyl)imino)butan-2-ylidene)-3,5-$d_2$-aniline $d_{22}$-S51**

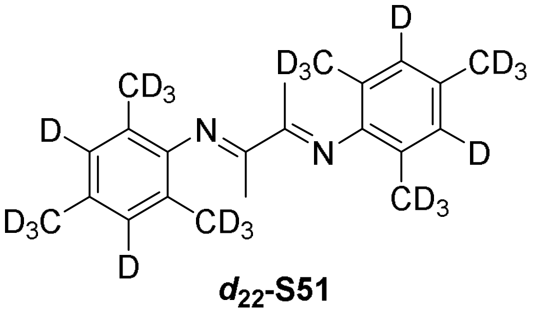

***$d_{22}$*-S51**

Formic acid (2 drops) was added to a stirred solution of $d_{11}$-trimethylaniline ***$d_{11}$*-S50**[4] (1.02 g, 7.0 mmol, 2.0 eq.) and 2,3-butanedione (367 µL, 3.5 mmol, 1.0 eq.) in MeOH (10 mL) at rt. The resulting solution was heated to 70 °C for 16 h during which time a precipitate formed. The precipitate was filtered and washed with EtOH (30 mL) and dried under reduced pressure to give diimine ***$d_{22}$*-S51** (1.19 g, 99%) as a yellow powder, **$^1$H NMR** (400 MHz, $CDCl_3$) $\delta$ 2.07 (s, 6H); **$^{13}$C NMR** (100.6 MHz, $CDCl_3$) $\delta$ 168.4 (s), 146.0 (s), 132.2 (s), 128.3 (t, $J$ = 21 Hz, CD), 124.4 (s), 19.8 (sept., $J$ = 19 Hz, $CD_3$), 16.9 (sept., $J$ = 19 Hz, $CD_3$), 15.8 (s); **MS** (ESI) m/z 365 [(M + Na)$^+$, 30], 343 [(M + H)$^+$, 100]; **HRMS** m/z calculated for $C_{22}H_7D_{22}N_2$ (M + H)$^+$ 343.3711, found 343.3707 (−0.2 ppm error).

**1,3-Bis-(2,4,6-tris($d_3$-methyl)-3,5-$d_2$-phenyl)-4,5-dimethylimidazolium chloride $d_{22}$-S37**

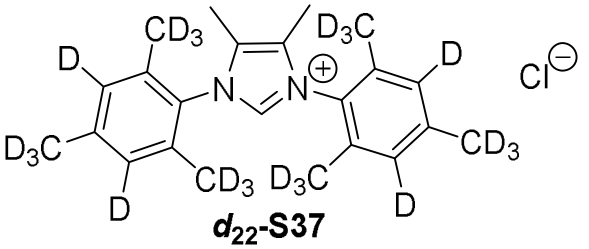

***$d_{22}$*-S37**

Using General Procedure B, diimine ***$d_{22}$*-S51** (500 mg, 1.46 mmol, 1.0 eq), paraformaldehyde (53 mg, 1.75 mmol, 1.2 eq.), HCl (0.55 mL of a 4 M solution in 1,4-dioxane, 2.19 mmol, 1.5 eq.) in EtOAc (10 mL) gave carbene salt ***$d_{22}$*-S37** (473 mg, 83%) as a pale yellow powder, **$^1$H NMR** (400 MHz, $CDCl_3$) $\delta$ 9.50 (s, 1H), 2.51 (s, 6H); **$^{13}$C NMR** (100.6 MHz, $CDCl_3$) $\delta$ 141.0 (s), 136.3 (s), 135.1 (s), 129.7 (t, $J$ = 25 Hz, CD), 129.5 (s) 128.3 (s), 16.4 (s), 8.6 (s); **MS** (ESI) m/z 355 [(M − Cl)$^+$, 100]; **HRMS** m/z calculated for $C_{23}H_7D_{22}N_2$ (M − Cl)$^+$ 355.3711, found 355.3718 (+0.7 ppm error).

**[IrCl(COD)(1,3-bis-(2,4,6-tris(*d*$_3$-methyl)-3,5-d$_2$-phenyl)-4,5-dimethylimidazol-2-ylidine)] *d*$_{22}$-19**

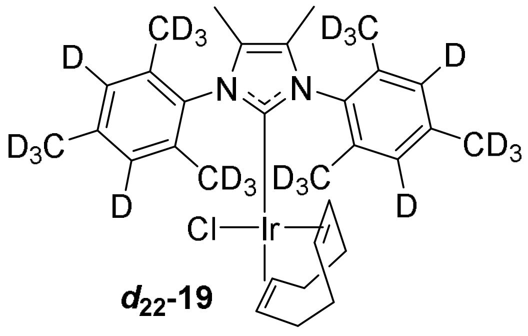


Using General Procedure C, carbene salt ***d*$_{22}$-S37** (161 mg, 0.41 mmol, 2.0 eq), KO$^t$Bu (60 mg, 0.53 mmol, 2.5 eq.), [IrCl(COD)]$_2$ (141 mg, 0.21 mmol, 1.0 eq.) in THF (10 mL) gave complex ***d*$_{22}$-19** (221 mg, 81%) as a yellow crystalline solid, **$^1$H NMR** (400 MHz, CDCl$_3$) $\delta$ 4.07-4.02 (m, 2H), 3.08-3.02 (m, 2H), 1.83 (s, 6H), 1.66-1.58 (m, 4H), 1.31-1.20 (m, 4H); **$^{13}$C NMR** (100.6 MHz, CDCl$_3$) $\delta$ 178.6 (s), 138.0 (s), 137.4 (s), 134.6 (s), 134.3 (s), 129.3 (t, $J$ = 24 Hz), 127.7 (t, $J$ = 23 Hz), 124.8 (s), 81.0 (s), 50.7 (s), 33.5 (s), 28.9 (s), 20.2 (sept., $J$ = 21 Hz), 18.8 (sept, $J$ = 18 Hz), 13.3 (sept, $J$ = 20 Hz), 9.2 (s); **MS** (ESI) m/z 655 [(M − Cl)$^+$, 100]; **HRMS** m/z calculated for $C_{31}H_{18}D_{22}{}^{193}IrN_2$ (M − Cl)$^+$ 655.4196, found 655.4195 (+1.7 ppm error).

## Synthesis of *d*$_{22}$-21

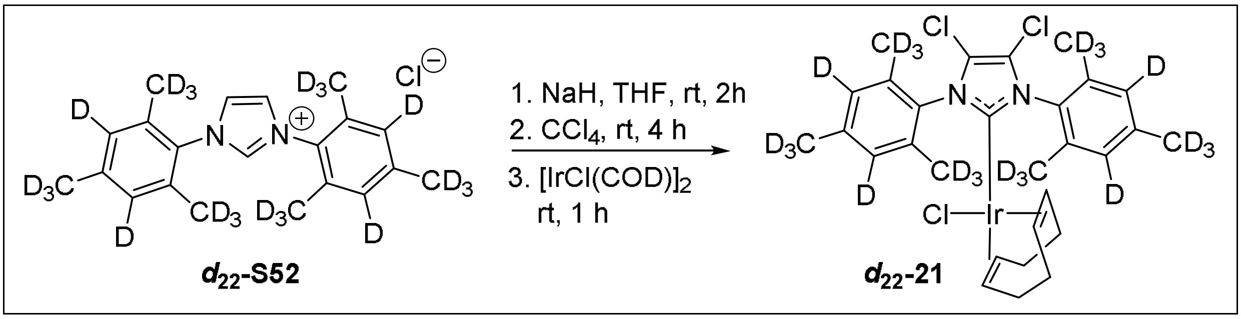


Supplementary Figure 28: Synthesis of ***d*$_{22}$-21**.

**[IrCl(COD)(1,3-bis(2,4,6-tris($d_3$-methyl)-3,5-$d_2$-phenyl)-4,5-dichloroimidazol-2-ylidine)] ($d_{22}$-21)**

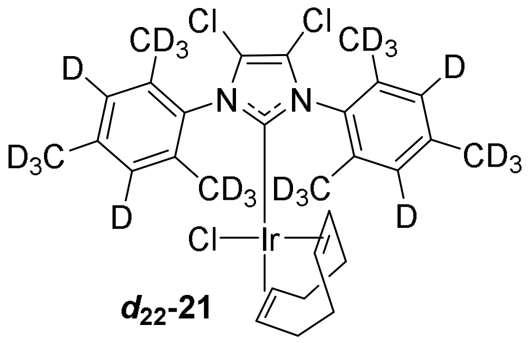

***d*₂₂-21**

Sodium hydride (33 mg of a 60% suspension in mineral oil, 0.83 mmol, 3.0 eq.) was added to a stirred suspension of $d_{22}$-IMes.HCl ***d*₂₂-S52**[1] (200 mg, 0.55 mmol, 2.0 eq) in THF (10 mL) at rt under $N_{2(g)}$. The resulting suspension was stirred at rt for 2 h. Then, $CCl_4$ (108 µL, 1.10 mmol, 4.0 eq.) was added dropwise and the resulting solution stirred at rt for 4 h. A solution of $[IrCl(COD)]_2$ (188 mg, 0.23 mmol, 1.0 eq.) in THF (3 mL) was added and the solution stirred at rt 1 h. The reaction was concentrated under reduced pressure to give the crude product. Purification by flash column chromatography with 4:6-0:10 hexane-$CH_2Cl_2$ gave complex ***d*₂₂-21** (304 mg, 76%) as a yellow crystalline solid, **$^{1}$H NMR** (400 MHz, $CDCl_3$) $\delta$ 4.26-4.18 (m, 2H), 3.08-2.98 (m, 2H), 1.72-1.60 (m, 4H), 1.43-1.26 (m, 4H); **$^{13}$C NMR** (100.6 MHz, $CDCl_3$) $\delta$ 183.0 (s), 139.4 (s), 137.8 (s), 135.0 (s), 132.9 (s), 129.5 (t, $J$ = 22 Hz), 127.9 (t, $J$ = 22 Hz), 118.0 (s), 83.7 (s), 51.9 (s), 33.4 (s), 28.9 (s), 20.3 (sept, $J$ = 21 Hz), 18.9 (sept, $J$ = 21 Hz), 17.5 (sept, $J$ = 21 Hz); **MS** (ESI) m/z 695 [$(M - Cl)^+$, 100]; **HRMS** $[M – Cl]^+$ calculated for $C_{29}H_{12}D_{22}{}^{35}Cl_2{}^{193}IrN_2$ 695.3104, found 695.3096 (+0.6 ppm error).

**Synthesis of 23**

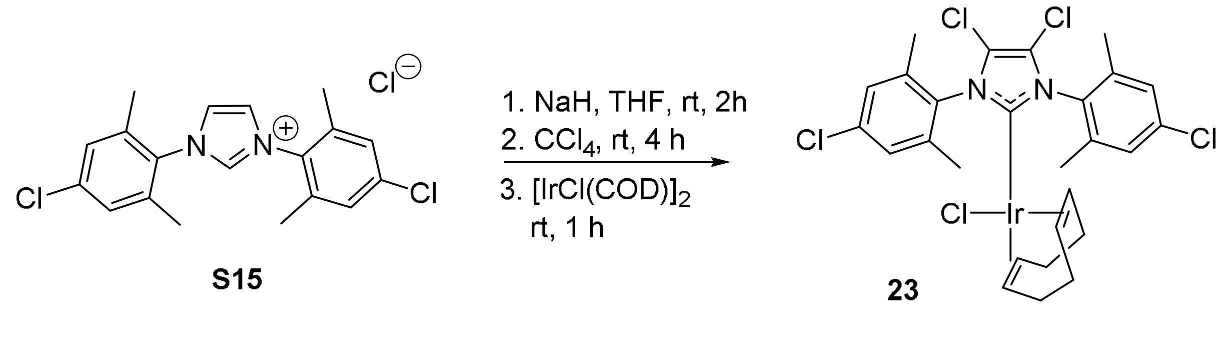


Supplementary Figure 29: Synthesis of **23**.

**[IrCl(COD)(1,3-bis(4-chloro-2,6-dimethylphenyl)-4,5-dichloroimidazol-2-ylidine)] (23)**

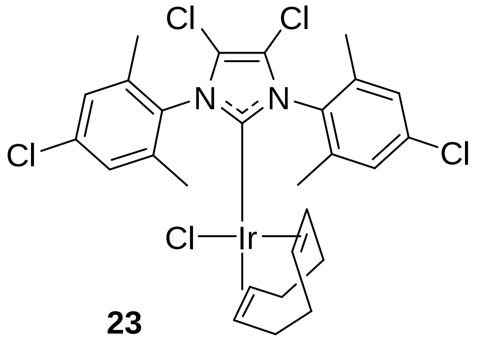


Sodium hydride (8 mg of a 60% suspension in mineral oil, 0.20 mmol, 3.0 eq.) was added to a stirred suspension of carbene salt **S15** (50 mg, 0.13 mmol, 2.0 eq) in THF (5 mL) at rt under $N_{2(g)}$. The resulting suspension was stirred at rt for 2 h. Then, $CCl_4$ (25 µL, 0.26 mmol, 4.0 eq.) was added dropwise and the resulting solution stirred at rt for 4 h. A solution of $[IrCl(COD)]_2$ (44 mg, 0.065 mmol, 1.0 eq.) in THF (1 mL) was added and the solution stirred at rt 1 h. The reaction was concentrated under reduced pressure to give the crude product. Purification by flash column chromatography with 4:6-0:10 hexane-$CH_2Cl_2$ gave complex **23** (61 mg, 63%) as a yellow crystalline solid, **$^1$H NMR** (400 MHz, $CDCl_3$) $\delta$ 7.27 (s, 2H), 7.24 (s, 2H), 4.34-4.29 (m, 2H), 2.97-2.93 (m, 2H), 2.40 (s, 6H), 2.15 (s, 4H), 1.77-1.64 (m, 4H), 1.47-1.33 (m, 4H); **$^{13}$C NMR** (100.6 MHz, $CDCl_3$) $\delta$ 183.5 (s), 140.5 (s), 137.4 (s), 135.5 (s), 133.7 (s), 129.1 (s), 127.5 (s), 118.2 (s), 85.4 (s), 52.2 (s), 33.4 (s), 28.9 (s), 19.8 (s), 18.4 (s); **MS** (ESI) m/z 713 [(M − Cl)$^+$, 100]; **HRMS** [M – Cl]$^+$ calculated for $C_{27}H_{28}{}^{35}Cl_2{}^{193}IrN_2$ 713.0630, found 713.0601 (+0.8 ppm error).

**Synthesis of *d*$_{16}$-23**

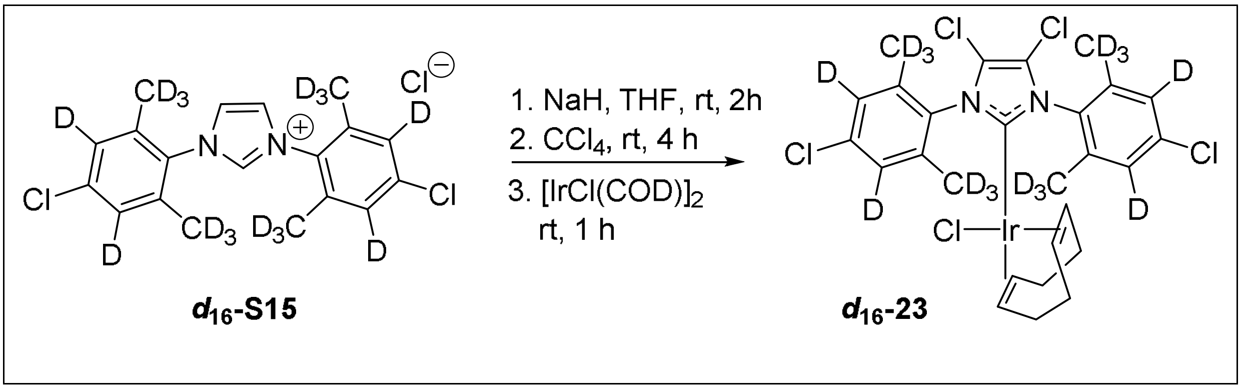


Supplementary Figure 30: Synthesis of ***d*$_{16}$-23**.

**[IrCl(COD)(1,3-bis(4-chloro-2,6-bis(*d*$_3$-methyl)-3,5-d$_2$-phenyl)-4,5-dichloroimidazol-2-ylidine)] (*d*$_{16}$-23)**

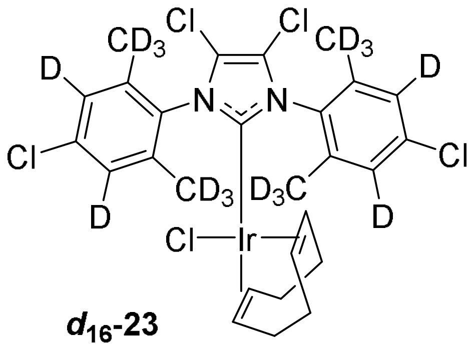


***d*$_{16}$-23**

Sodium hydride (8 mg of a 60% suspension in mineral oil, 0.20 mmol, 3.0 eq.) was added to a stirred suspension of carbene salt ***d*$_{22}$-S15** (52 mg, 0.13 mmol, 2.0 eq) in THF (5 mL) at rt under $N_{2(g)}$. The resulting suspension was stirred at rt for 2 h. Then, $CCl_4$ (25 µL, 0.26 mmol, 4.0 eq.) was added dropwise and the resulting solution stirred at rt for 4 h. A solution of $[IrCl(COD)]_2$ (44 mg, 0.065 mmol, 1.0 eq.) in THF (1 mL) was added and the solution stirred at rt 1 h. The reaction was concentrated under reduced pressure to give the crude product. Purification by flash column chromatography with 4:6-0:10 hexane-$CH_2Cl_2$ gave complex ***d*$_{16}$-23** (68 mg, 69%) as a yellow crystalline solid, **$^1$H NMR** (400 MHz, $CDCl_3$) $\delta$ 4.33-4.29 (m, 2H), 2.96-2.94 (m, 2H), 2.15 (s, 4H), 1.76-1.63 (m, 4H), 1.48-1.33 (m, 4H); **$^{13}$C NMR** (100.6 MHz, $CDCl_3$) $\delta$ 183.5 (s), 140.3 (s), 137.2 (s), 135.3 (s), 133.8 (s), 128.8 (t, $J$ = 23.5 Hz, CD), 127.2 (t, $J$ = 24.5 Hz, CD), 118.1 (s), 85.4 (s), 52.1 (s), 33.4 (s), 28.8 (s), 18.9 (sept., $J$ = 19 Hz, $CD_3$), 17.5 (sept., $J$ = 19 Hz, $CD_3$); **MS** (ESI) m/z 729 [$(M - Cl)^+$, 100]; **HRMS** $[M – Cl]^+$ calculated for $C_{27}H_{12}D_{16}{}^{35}Cl_2{}^{193}IrN_2$ 729.1635, found 719.1563 (+7.3 ppm error).

## Determination of Tolman Electronic Parameters (TEP)

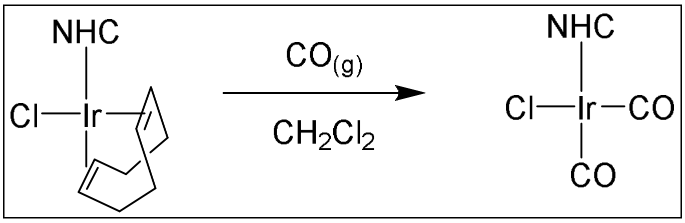


Supplementary Figure 31: Synthesis of $[IrCl(CO)_2(NHC)]$ complexes

$CO_{(g)}$ was bubbled through a solution of [IrCl(COD)(NHC)] in $CH_2Cl_2$ for 2 min. The resulting solution was concentrated under reduced pressure. The resulting crude solid was triturated with hexane to give the desired $[IrCl(CO)_2(NHC)]$ complex. $^1H$ and $^{13}C$ NMR data are detailed below and IR data can be found in Supplementary Table 1.

### $[IrCl(CO)_2(IMes)]$ S53

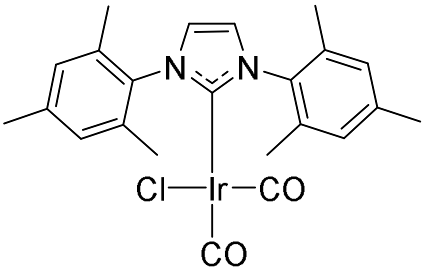


**$^1H$ NMR** (400 MHz, $CD_2Cl_2$) $\delta$ 7.19 (s, 2H), 7.08 (s, 4H), 2.42 (s, 6H), 2.24 (s, 12H); **$^{13}C$ NMR** (100.6 MHz, $CD_2Cl_2$) $\delta$ 180.3 (s), 175.4 (s), 168.4 (s), 139.6 (s), 135.3 (s), 134.9 (s), 129.1 (s), 123.9 (s), 20.9 (s), 18.2 (s);

### $[IrCl(CO)_2$(1,3-bis(4-methylphenyl)imidazol-2-ylidine)] S54

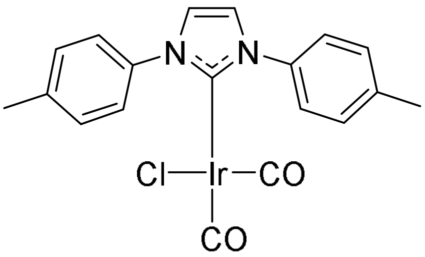


**$^1H$ NMR** (400 MHz, $CD_2Cl_2$) $\delta$ 7.69 (d, $J$ = 8.0 Hz, 4H), 7.41 (s, 2H), 7.39 (d, J = 8.0 Hz, 4H), 2.50 (s, 6H); **$^{13}C$ NMR** (100.6 MHz, $CD_2Cl_2$) $\delta$ 181.0 (s), 170.0 (s), 168.0 (s), 139.5 (s), 136.9 (s), 129.7 (s), 126.1 (s), 123.3 (s), 21.0 (s).

**IrCl(CO)$_2$(1,3-bis(2,6-dimethylphenyl)imidazol-2-ylidine)] S55**

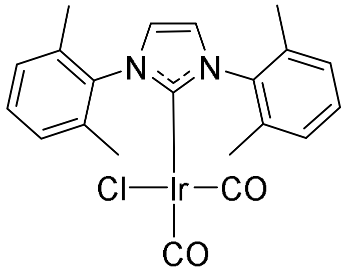


**$^1$H NMR** (400 MHz, $CD_2Cl_2$) $\delta$ 7.40 (t, $J$ = 7.6 Hz, 2H), 7.27 (d, J = 7.6 Hz, 4H), 7.25 (s, 2H), 2.29 (s, 12H); **$^{13}$C NMR** (100.6 MHz, $CD_2Cl_2$) $\delta$ 180.1 (s), 175.2 (s), 168.3 (s), 137.3 (s), 135.7 (s), 129.6 (s), 128.5 (s), 123.8 (s), 18.4 (s);

**[IrCl(CO)$_2$(1,3-bis(2,6-diethylphenyl)imidazol-2-ylidine)] S56**

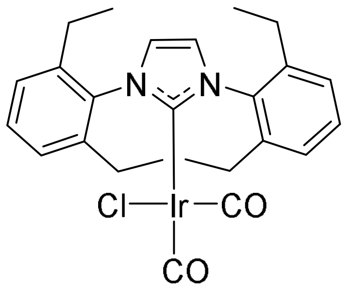


**$^1$H NMR** (400 MHz, $CD_2Cl_2$) $\delta$ 7.52 (t, $J$ = 7.7 Hz, 2H), 7.34 (d, $J$ = 7.7 Hz, 4H), 7.27 (s, 2H), 2.72 (dq, $J$ = 15.1, 7.5 Hz, 4H), 2.48 (dq, $J$ = 15.1, 7.5 Hz, 4H), 1.26 (t, $J$ = 7.9 Hz, 12H); **$^{13}$C NMR** (100.6 MHz, $CD_2Cl_2$) $\delta$ 180.1 (s), 177.8 (s), 168.5 (s), 146.1 (s), 134.7 (s), 130.5 (s), 125.0 (s), 124.0 (s), 28.9 (s), 26.1 (s), 22.3 (s);

**[IrCl(CO)$_2$(IPr)] S57**

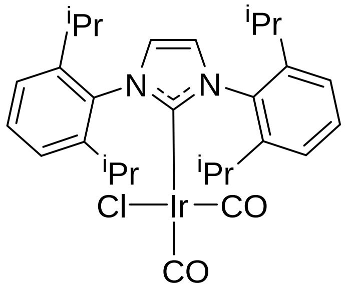


**$^1$H NMR** (400 MHz, $CD_2Cl_2$) $\delta$ 7.57 (t, $J$ = 7.7 Hz, 2H), 7.28 (d, $J$ = 7.7 Hz, 4H), 7.27 (s, 2H), 2.90 (sept., $J$ = 6.8 Hz, 4H), 1.41 (d, $J$ = 6.8 Hz, 12H), 1.17 (d, $J$ = 6.8 Hz, 12H); **$^{13}$C NMR** (100.6 MHz, $CD_2Cl_2$) $\delta$ 180.1 (s), 177.8 (s), 168.5 (s), 146.1 (s), 134.7 (s), 130.5 (s), 125.0 (s), 124.0 (s), 28.9 (s), 26.1 (s), 22.3 (s);

**[IrCl(CO)$_2$(1,3-bis(4,5-dimethylphenyl)imidazol-2-ylidine)] S58**

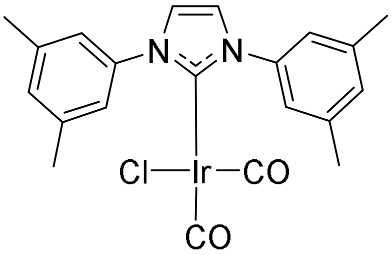


[1]**H NMR** (400 MHz, $CD_2Cl_2$) $\delta$ 7.43 (s, 4H), 7.39 (s, 2H), 7.19 (s, 2H), 2.45 (s, 12H); [13]**C NMR** (100.6 MHz, $CD_2Cl_2$) $\delta$ 181.0 (s), 172.8 (s), 168.1 (s), 139.2 (s), 139.1 (s), 130.6 (s), 124.0 (s), 123.1 (s), 21.0 (s);

**[IrCl(CO)$_2$(1,3-bis(3,4,5-trimethylphenyl)imidazol-2-ylidine)] S59**

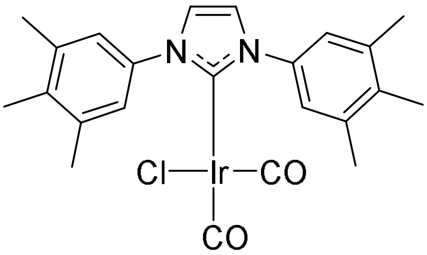


[1]**H NMR** (400 MHz, $CD_2Cl_2$) $\delta$ 7.44 (s, 4H), 7.35 (s, 2H), 2.41 (s, 12H), 2.29 (s, 6H); [13]**C NMR** (100.6 MHz, $CD_2Cl_2$) $\delta$ 181.2 (s), 172.4 (s), 168.3 (s), 137.6 (s), 136.4 (s, 2C), 125.0 (s), 123.1 (s), 20.4 (s), 15.1 (s);

**[IrCl(CO)$_2$(1,3-bis(4-fluoro-2,6-dimethylphenyl)imidazol-2-ylidine)] S60**

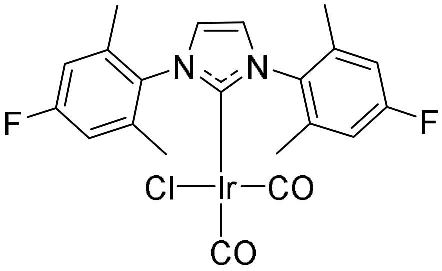


[1]**H NMR** (400 MHz, $CD_2Cl_2$) $\delta$ 7.24 (s, 2H), 7.69 (d, $J$ = 8.8 Hz, 4H), 2.28 (s, 12H); [13]**C NMR** (100.6 MHz, $CD_2Cl_2$) $\delta$ 179.9 (s), 176.3.7 (s), 168.3 (s), 162.5 (d, $J_{C\text{-}F}$ = 246 Hz), 138.4 (d, $J_{C\text{-}F}$ = 9 Hz), 133.3 (s), 124.1 (s), 115.0 (d, $J_{C\text{-}F}$ = 23 Hz), 18.6 (s);

**[IrCl(CO)$_2$(1,3-bis(4-chloro-2,6-dimethylphenyl)imidazol-2-ylidine)] S61**

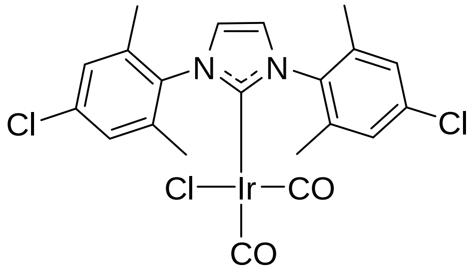


**$^{1}$H NMR** (400 MHz, $CD_2Cl_2$) $\delta$ 7.27 (s, 4H), 7.24 (s, 2H), 2.26 (s, 12H); **$^{13}$C NMR** (100.6 MHz, $CD_2Cl_2$) $\delta$ 179.9 (s), 175.7 (s), 168.2 (s), 137.7 (s), 135.8 (s), 135.0 (s), 128.4 (s), 124.0 (s), 18.4 (s);

**[IrCl(CO)$_2$(1,3-bis(4-bromo-2,6-dimethylphenyl)imidazol-2-ylidine)] S62**

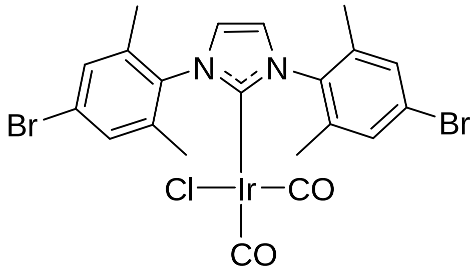


**$^{1}$H NMR** (400 MHz, $CD_2Cl_2$) $\delta$ 7.43 (s,4H), 7.23 (s, 2H), 2.26 (s, 12H); **$^{13}$C NMR** (100.6 MHz, $CD_2Cl_2$) $\delta$ 179.8 (s), 175.7 (s), 168.2 (s), 137.9 (s), 136.3 (s), 131.4 (s), 123.9 (s), 123.3 (s), 18.3 (s);

**[IrCl(CO)$_2$(1,3-bis(4-iodo-2,6-dimethylphenyl)imidazol-2-ylidine)] S63**

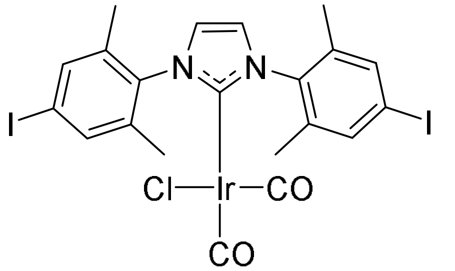


**$^{1}$H NMR** (400 MHz, $CD_2Cl_2$) $\delta$ 7.60 (s,4H), 7.18 (s, 2H), 2.19 (s, 12H); **$^{13}$C NMR** (100.6 MHz, $CD_2Cl_2$) $\delta$ 180.2 (s), 176.0 (s), 168.6 (s), 138.3 (s), 137.9 (s), 137.5 (s), 124.2 (s), 96.0 (s), 18.4 (s);

**[IrCl(CO)$_2$(1,3-bis(4-methoxycarbonyl-2,6-dimethylphenyl)imidazol-2-ylidine)] S64**

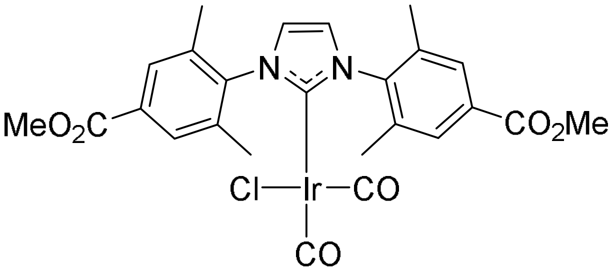


**$^{1}$H NMR** (400 MHz, $CD_2Cl_2$) 7.89 (s, 4H), 7.26 (s, 2H), 3.93 (s, 6H), 2.31 (s, 12H); **$^{13}$C NMR** (101 MHz, $CD_2Cl_2$) 180.1 (s), 175.7 (s), 168.5 (s), 166.6 (s), 141.2 (s), 136.7 (s), 131.8 (s), 130.0 (s), 124.1 (s), 52.6 (s), 18.9 (s);

**[IrCl(CO)$_2$(1,3-bis(4-(trifluoromethanesulfonyl)-2,6-dimethylphenyl)imidazol-2-ylidine)] S65**

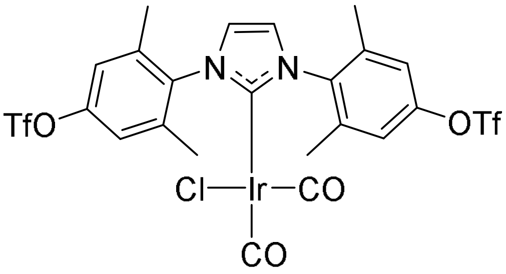


**$^{1}$H NMR** (400 MHz, $CD_2Cl_2$) $\delta$ 7.30 (s, 2H), 7.22 (s, 4H), 2.34 (s, 12H); **$^{13}$C NMR** (100.6 MHz, $CD_2Cl_2$) $\delta$ 179.5 (s), 176.2 (s), 168.0 (s), 149.5 (s), 139.0 (s), 136.9 (s), 124.0 (s), 121.1 (s), 118.7 (q, $J$ = 316 Hz, $CF_3$), 18.8 (s).

**[IrCl(CO)$_2$(1,3-bis(4-acetoxy-2,6-dimethylphenyl)imidazol-2-ylidine)] S66**

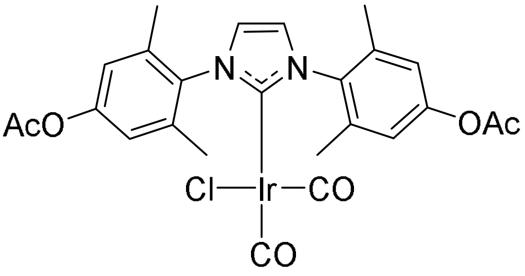


**$^{1}$H NMR** (400 MHz, $CD_2Cl_2$) $\delta$ 7.27 (s, 2H), 7.01 (s, 4H), 2.35 (s, 6H), 2.28 (s, 12H); **$^{13}$C NMR** (100.6 MHz, $CD_2Cl_2$) $\delta$ 179.9 (s), 175.7 (s), 168.3 (s), 151.1 (s), 137.4 (s), 134.7 (s), 124.1 (s), 121.5 (s), 21.0 (s), 18.6 (s);

**[IrCl(CO)$_2$(1,3-bis(4-phenyl-2,6-dimethylphenyl)imidazol-2-ylidine)] S67**

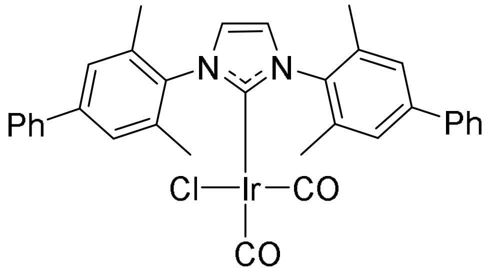


**$^{1}$H NMR** (400 MHz, $CD_2Cl_2$) *δ* 7.73 (d, *J* = 6.7 Hz, 4H), 7.75-7.51 (m, 8H), 7.44 (tt, *J* = 7.3, 2.1 Hz, 2H), 7.29 (s, 2H), 2.38 (s, 12H); **$^{13}$C NMR** (100.6 MHz, $CD_2Cl_2$) *δ* 180.2 (s), 175.5 (s), 168.5 (s), 142.2 (s), 139.9 (s), 136.6 (s), 136.1 (s), 128.9 (s), 128.6 (s), 127.8 (s), 127.1 (s), 124.0 (s), 18.6 (s);

**[IrCl(CO)$_2$(1,3-bis(4-(*tert*-butyl)-2,6-dimethylphenyl)imidazol-2-ylidine)] S68**

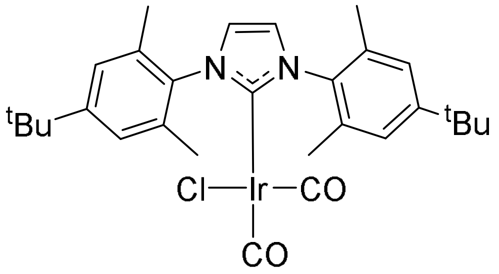


**$^{1}$H NMR** (400 MHz, $CD_2Cl_2$) *δ* 7.27 (s, 4H), 7.19 (s, 2H), 2.27 (s, 12H), 1.41 (s, 18H); **$^{13}$C NMR** (100.6 MHz, $CD_2Cl_2$) *δ* 180.4 (s), 175.2 (s), 168.5 (s), 152.5 (s), 134.9 (s), 125.6 (s), 124.0 (s), 34.5 (s), 31.0 (s), 18.5 (s);

**[IrCl(CO)$_2$(1,3-bis(4-methoxy-2,6-dimethylphenyl)imidazol-2-ylidine)] S69**

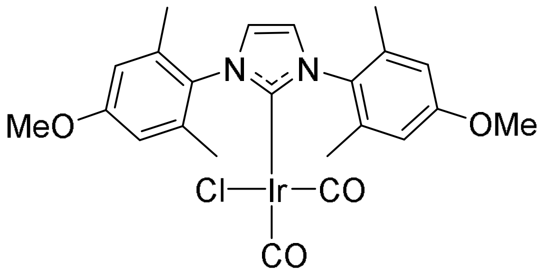


**$^{1}$H NMR** (400 MHz, $CD_2Cl_2$) *δ* 7.19 (s, 2H), 6.78 (s, 4H), 3.89 (s, 6H), 2.25 (s, 12H); **$^{13}$C NMR** (100.6 MHz, $CD_2Cl_2$) *δ* 180.4 (s), 176.1 (s), 168.5 (s), 159.8 (s), 137.0 (s), 130.4 (s), 124.1 (s), 113.4 (s), 55.3 (s), 18.6 (s);

**[IrCl(CO)$_2$(1,3-bis(4-dimethylamino-2,6-dimethylphenyl)imidazol-2-ylidine)] S70**

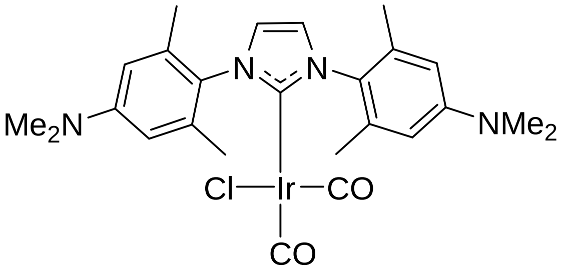


**$^{1}$H NMR** (400 MHz, $CD_2Cl_2$) 7.09 (s, 2H), 6.50 (s, 4H), 3.01 (s, 12H), 2.18 (s, 12H); **$^{13}$C NMR** (101 MHz, $CD_2Cl_2$) 181.1 (s), 176.5 (s), 169.1 (s), 151.1 (s), 136.3 (s), 127.2 (s), 124.7 (s), 111.6 (s), 40.5 (s), 19.1 (s);

**[IrCl(CO)$_2$(1,3-dimesityl-4,5-dimethylimidazol-2-ylidine)] S71**

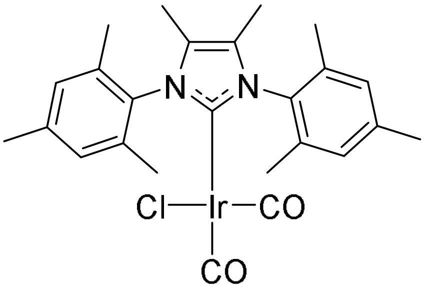


**$^{1}$H NMR** (400 MHz, $CD_2Cl_2$) $\delta$ 7.08 (s, 4H), 2.41 (s, 6H), 2.16 (s, 12 H), 1.89 (s, 6H); **$^{13}$C NMR** (100.6 MHz, $CD_2Cl_2$) $\delta$ 180.6 (s), 172.0 (s), 168.8 (s), 139.4 (s), 135.6 (s), 133.2 (s), 129.2 (s), 127.1 (s), 21.0 (s), 18.1 (s), 8.9 (s);

**[IrCl(CO)$_2$(1,3-dimesityl-4,5-diethylimidazol-2-ylidine)] S72**

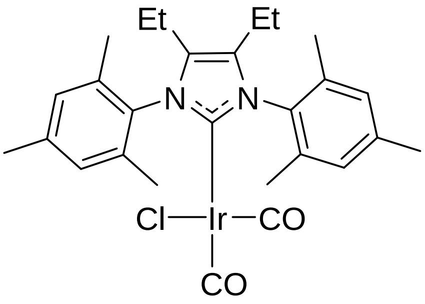


**$^{1}$H NMR** (400 MHz, $CD_2Cl_2$) $\delta$ 7.08 (s, 4H), 2.42 (s, 6H), 2.36 (q, $J$ = 7.6 Hz, 4H), 2.19 (s, 12 H), 0.98 (t, $J$ = 7.6, 6H); **$^{13}$C NMR** (100.6 MHz, $CD_2Cl_2$) $\delta$ 180.5 (s), 172.5 (s), 168.8 (s), 139.3 (s), 135.7 (s), 133.2 (s), 132.7 (s), 129.2 (s), 21.0 (s), 18.5 (s), 17.0 (s), 13.5 (s);

**[IrCl(CO)$_2$(1,3-dimesityl-4,5-dichlorolimidazol-2-ylidine)] S73**

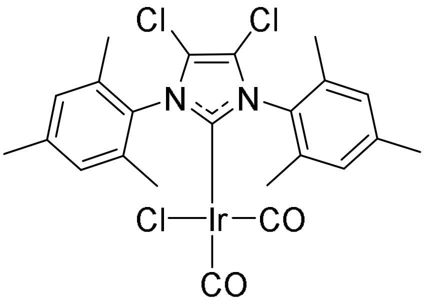


**$^{1}$H NMR** (400 MHz, $CD_2Cl_2$) $\delta$ 7.13 (s, 4H), 2.45 (s, 6H), 2.24 (s, 12 H), 0.98 (t, $J$ = 7.6, 6H); **$^{13}$C NMR** (100.6 MHz, $CD_2Cl_2$) $\delta$ 179.3 (s), 176.1 (s), 168.2 (s), 140.8 (s), 135.9 (s), 131.9 (s), 129.4 (s), 119.3 (s), 21.0 (s), 18.2 (s);

**[IrCl(CO)$_2$(1,3-dimesityl-4,5-dibromoimidazol-2-ylidine)] S74**

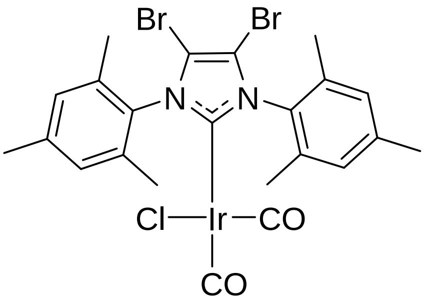


**$^{1}$H NMR** (400 MHz, $CD_2Cl_2$) $\delta$ 7.11 (s, 4H), 2.44 (s, 6H), 2.21 (s, 12 H); **$^{13}$C NMR** (100.6 MHz, $CD_2Cl_2$) $\delta$ 179.2 (s), 177.9 (s), 168.3 (s), 140.7 (s), 135.8 (s), 133.3 (s), 129.4 (s), 110.3 (s), 21.1 (s), 18.3 (s);

**[IrCl(CO)$_2$(1,3-bis(4-chloro-2,6-dimethylphenyl)-4,5-dichloroimidazol-2-ylidine)] S75**

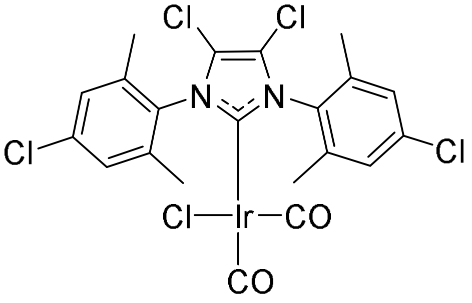


**$^{1}$H NMR** (400 MHz, $CD_2Cl_2$) $\delta$ 7.27 (s, 4H), 2.22 (s, 12H); **$^{13}$C NMR** (100.6 MHz, $CD_2Cl_2$) $\delta$ 179.2 (s), 176.9 (s), 168.4 (s), 138.7 (s), 136.5 (s), 133.3 (s), 129.1 (s), 120.0 (s), 18.7 (s);

The carbonyl frequency of the $[IrCl(CO)_2(NHC)]$ complexes were measured in a $CH_2Cl_2$ solution using a Bruker Tensor 37 FTIR spectrometer. The Tolman Electronic Parameter was then calculated from the average carbonyl frequencies using the Supplementary Equation 1is well established.[11, 12]

$$TEP\ (cm^{-1}) = 0.847[\nu_{CO}(average)] + 336$$

| Pre-Catalyst | CO Complex | $CO^1$ / $cm^{-1}$ | $CO^2$/ $cm^{-1}$ | Vac(CO) / $cm^{-1}$ | TEP / $cm^{-1}$ |
|---|---|---|---|---|---|
| **1** | **S53** | 2066.4 | 1979.9 | 2023.2 | 2049.7 |
| **2** | **S54** | 2065.6 | 1982.9 | 2024.3 | 2050.5 |
| **3** | **S55** | 2067.6 | 1981.1 | 2024.4 | 2050.6 |
| **4** | **S56** | 2066.5 | 1980.2 | 2023.4 | 2049.8 |
| **5** | **S57** | 2066.7 | 1980.8 | 2023.8 | 2050.1 |
| **6** | **S58** | 2064.8 | 1981.5 | 2023.2 | 2049.6 |
| **7** | **S59** | 2064.0 | 1980.7 | 2022.4 | 2048.9 |
| **8** | **S60** | 2068.6 | 1981.9 | 2025.3 | 2051.4 |
| **9** | **S61** | 2069.2 | 1982.0 | 2025.6 | 2051.7 |
| **10** | **S62** | 2069.5 | 1982.2 | 2025.9 | 2051.9 |
| **11** | **S63** | 2069.3 | 1982.4 | 2025.9 | 2051.9 |
| **12** | **S64** | 2069.7 | 1982.7 | 2026.2 | 2052.2 |
| **13** | **S65** | 2071.3 | 1983.5 | 2027.4 | 2053.2 |
| **14** | **S66** | 2068.7 | 1981.5 | 2025.1 | 2051.3 |
| **15** | **S67** | 2067.3 | 1980.8 | 2024.1 | 2050.4 |
| **16** | **S68** | 2065.9 | 1980.3 | 2023.1 | 2049.6 |
| **17** | **S69** | 2066.0 | 1979.6 | 2022.8 | 2049.3 |
| **18** | **S70** | 2063.7 | 1977.8 | 2020.8 | 2047.6 |
| **19** | **S71** | 2063.4 | 1977.1 | 2020.3 | 2047.2 |
| **20** | **S72** | 2063.0 | 1979.1 | 2021.1 | 2047.8 |
| **21** | **S73** | 2070.1 | 1983.7 | 2026.9 | 2052.8 |
| **22** | **S74** | 2069.7 | 1983.2 | 2026.5 | 2052.4 |
| **23** | **S75** | 2073.3 | 1985.6 | 2029.5 | 2054.9 |

Supplementary Table 1: TEP analysis for catalysts **1-22**

## X-Ray Crystallography

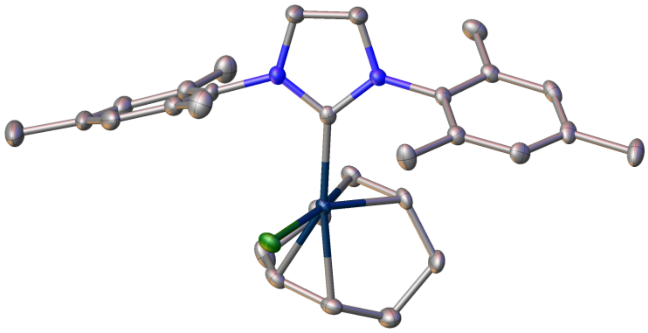

Supplementary Figure 32: ORTEP structure of **1**. Ellipsoids set at 30% probability; hydrogen atoms and solvent molecules have been omitted for clarity. Ir-$C_1$ Bond Length: 2.048 ± 0.004 Å. CCDC: 1820372

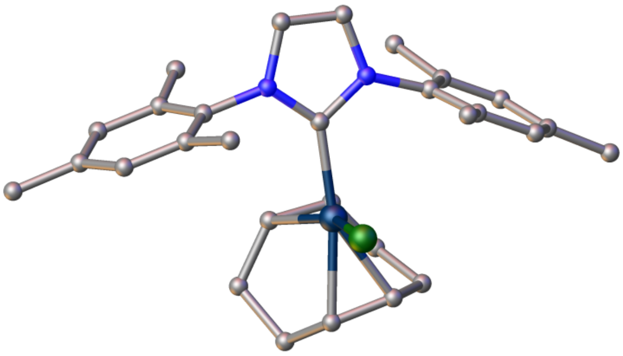

Supplementary Figure 33: ORTEP structure of ***d*₂₂-1**. Ellipsoids set at 30% probability; hydrogen and deuterium atoms have been omitted for clarity. Ir-$C_1$ Bond Length: 2.047 ± 0.002 Å. CCDC: 1823650

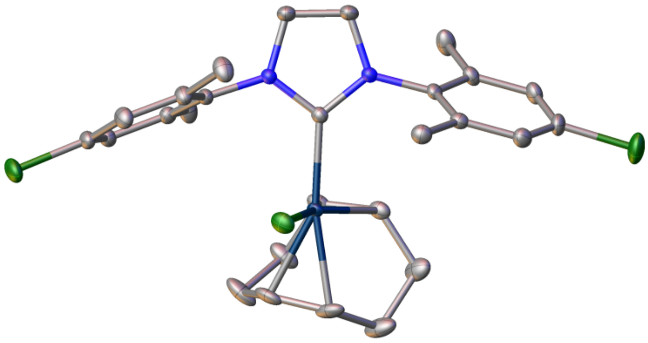

Supplementary Figure 34: ORTEP structure of **9**. Ellipsoids set at 30% probability; hydrogen atoms and solvent molecules have been omitted for clarity. Ir-$C_1$ Bond Length: 2.038 ± 0.002 Å.  CCDC: 1820373

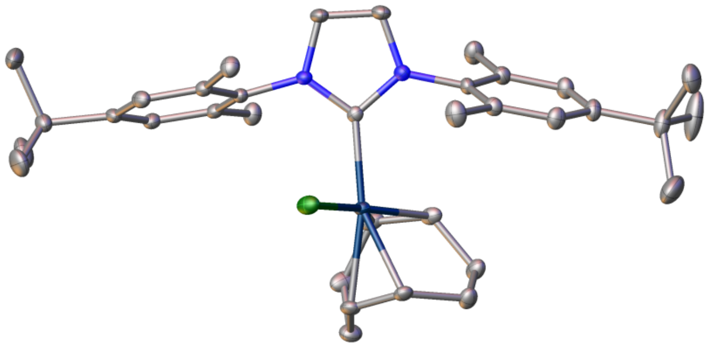

Supplementary Figure 35: ORTEP structure of **16**. Ellipsoids set at 30% probability; hydrogen atoms and solvent molecules have been omitted for clarity. Ir-$C_1$ Bond Length: 2.053 ± 0.002 Å. CCDC: 1820374

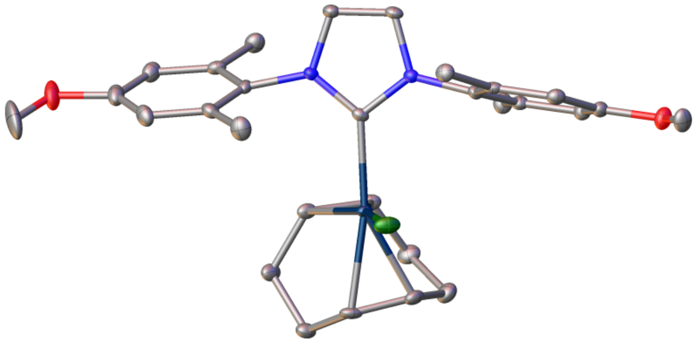

Supplementary Figure 36: ORTEP structure of **17**. Ellipsoids set at 30% probability; hydrogen atoms have been omitted for clarity. Ir-$C_1$ Bond Length: 2.048 ± 0.003 Å. CCDC: 1820375

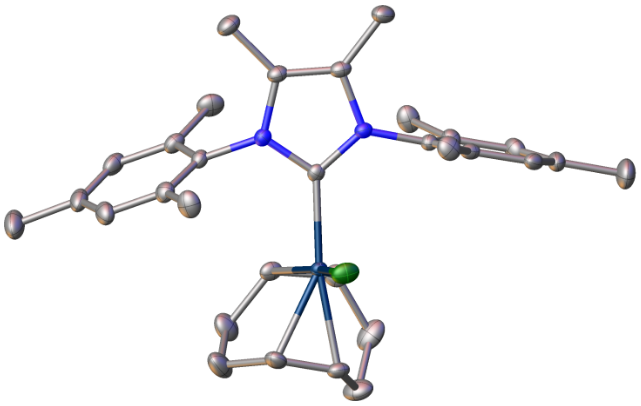

Supplementary Figure 37: ORTEP structure of **19**. Ellipsoids set at 30% probability; hydrogen atoms have been omitted for clarity. Ir-$C_1$ Bond Length: 2.056. CCDC: 1820376

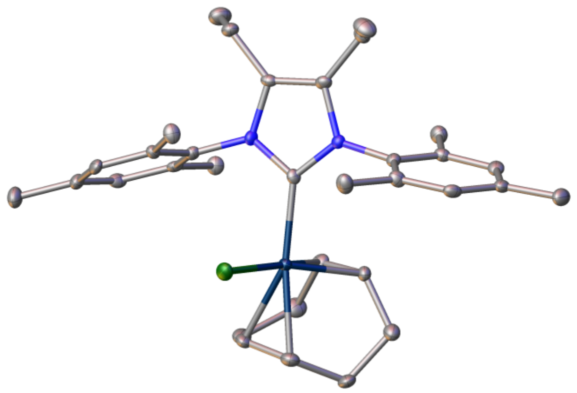

Supplementary Figure 38: ORTEP structure of **20**. Ellipsoids set at 30% probability; hydrogen atoms and solvent molecules have been omitted for clarity. Ir-$C_1$ Bond Length: 2.049 ± 0.005 Å. CCDC: 1820377

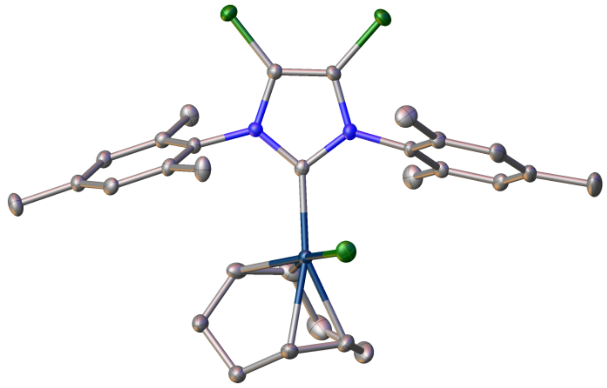

Supplementary Figure 39: ORTEP structure of **21**. Ellipsoids set at 30% probability; hydrogen atoms have been omitted for clarity. Ir-$C_1$ Bond Length: 2.034 ± 0.002 Å. CCDC: 1820378

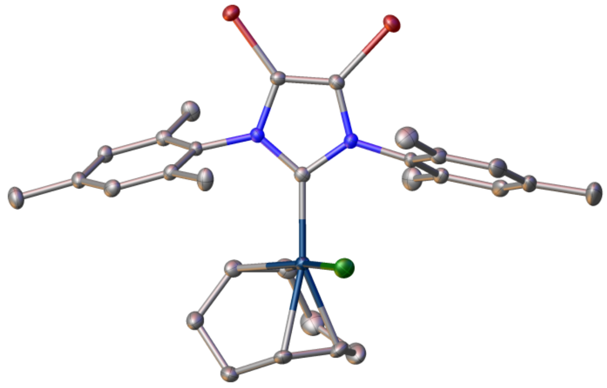

Supplementary Figure 40: ORTEP structure of **22**. Ellipsoids set at 30% probability; hydrogen atoms have been omitted for clarity. Ir-$C_1$ Bond Length: 2.042 ± 0.002 Å. CCDC: 1820379

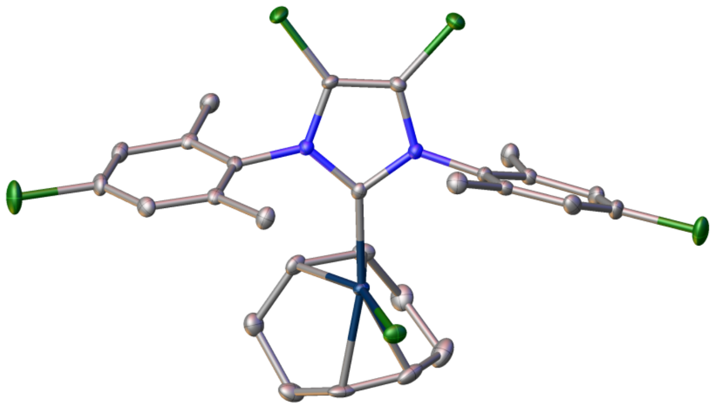

Supplementary Figure 41: ORTEP structure of **23**. Ellipsoids set at 30% probability; hydrogen atoms have been omitted for clarity. Ir-$C_1$ Bond Length: 2.030 ± 0.002 Å. CCDC: 1820380

Supplementary Figure 42 shows that the Ir-$C_1$ Bond Length is proportional to the TEP values calculated previously.

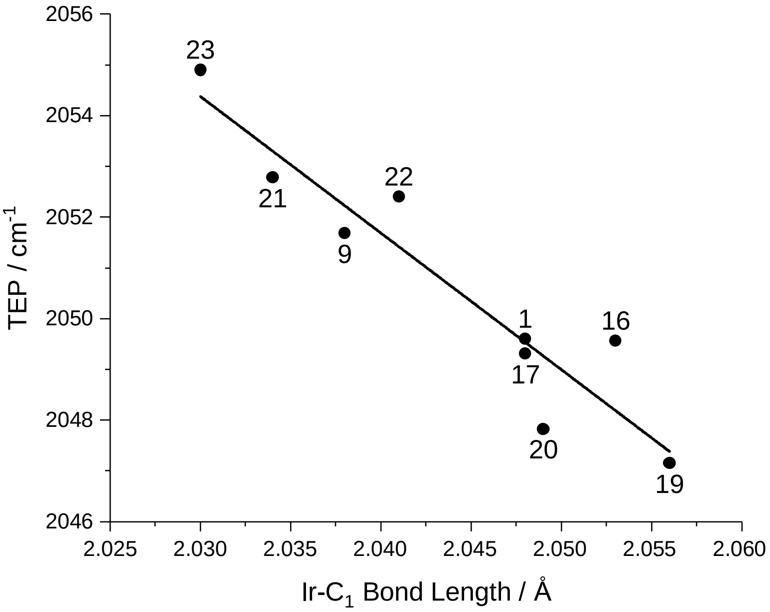


Supplementary Figure 42: Relationship between TEP and Ir-$C_1$ Bond Length

**Determination of Buried Volume**

All density function calculations were undertaken at the GGA level with the Gaussian09 set of programs.[13] The BP86 functional from Becke and Perdew was used for the optimisations[14, 15, 16] and were carried out in the gas phase. The basis sets with polarisation functions from Ahlrichs and co-workers (TZVP keyword in Gaussian09) were used for H, C, N, Cl, O, F and S atoms.[17, 18] For I atoms, the same basis set and associated electronic core potential was taken from the cosmologic website.[19] For the Ir atom, the SDD basis set and associated ECP was used.[20] The geometries obtained were then used to obtain the % buried volume parameters with the SambVca website, created by Cavallo et al.[21] The calculations performed here are at the same level of theory used in the creation of this web tool. Full Cartesian coordinates are available from the York Data Catalogue.

**SABRE Experiments**

A 5 mm J. Young's tap NMR tube containing a 5 mM (unless otherwise stated) solution of [IrCl(COD)(NHC)] and substrate (4 eq.) in methanol-$d_4$ (0.6 mL) was degassed prior to the introduction of $p$-$H_2$ (3 bar unless otherwise stated). Samples were then shaken for 10 s in the specified polarization transfer field before being rapidly transported into the magnet for subsequent interrogation by NMR spectroscopy. For $^{1}H$, the typical polarization transfer field was 65 G, for $^{13}C$ it was 0.5 G and for $^{15}N$ the sample was shaken inside a μ-metal shield with ca. 350-fold shielding.

For calculation of the enhancement of $^{1}H$ NMR signals the following formula was used:

$$E = \frac{SI(pol)}{SI(unpol)}$$

Where, E = enhancement, SI(pol) = signal of polarized sample, SI(unpol) = signal of unpolarized (reference) sample. Experimentally, both spectra were recorded on the same sample using identical acquisition parameters, including the receiver gain. The raw integrals of the relevant resonances in the polarized and unpolarized spectra were then used to determine the enhancement levels.

Heteronuclear enhancement factors were determined by comparison to either spectra obtained of high concentration solutions or spectra obtained under signal averaging. Calculations were made using standard literature methods.[22]

## Determination of Rate of Ligand Loss

The effect of the different NHC ligands on the rate loss of $\mathbf{A_{equ}}$ from the activate catalysts of type $[Ir(H)_2(NHC)(\mathbf{A})_3]Cl$ in methanol-$d_4$was determined through the use EXSY methods. Integrals for the interchanging peaks in the associated $^1H$ EXSY spectra were obtained and converted into a percentage of the total detected signal. An example of the data collected is shown in Supplementary Figure 12.

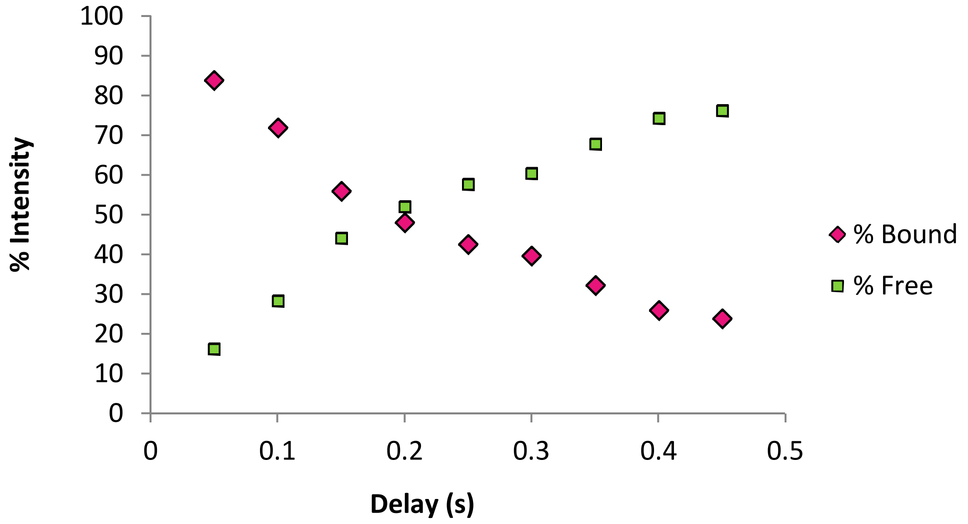


Supplementary Figure 43: Plot of the % intensity of the bound and free NMR signals as the delay between excitation and acquisition is increased.

In order to extract rate constants for nicotinate dissociation, a model must be devised to simulate the dynamic processes that give rise to the experimental data. The exchange pathway given in Supplementary Figure 44 has been derived for pyridine, however this same model can be applied to nicotinate, where $k_a$ is the rate constant of pyridine association, and $k_d$ is the rate constant of pyridine dissociation. In the case of pyridine dissociation from complex A to form complex B, either of the two bound excited pyridine ligands can dissociate. In the case of relaxed pyridine dissociation from complex C to form complex B, only the one bound, relaxed pyridine can dissociate, thus the probability of forming B from C is half that of forming B from A. For this reason, the net rate of dissociation from A to B is $2k_d$ rather than $k_d$.

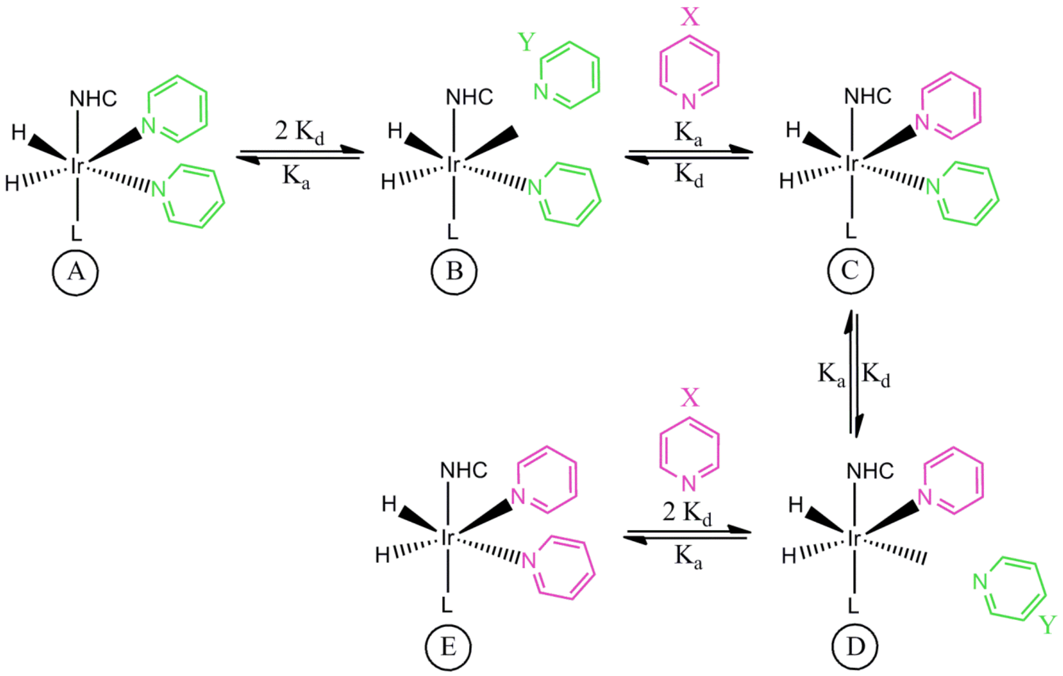


Supplementary Figure 44: Exchange pathways for excited signals corresponding to bound pyridine, where green pyridine is excited and pink pyridine is relaxed.

The equations for the concentration of each species A-E, and X and Y, are given in Supplementary Equations 1-7 for a time $\partial t$ after the initial time $t$ can be derived from the dynamic processes shown in Supplementary Figure 44.

$$[A]_{t+\partial t} = [A]_0 - 2k_d[A]_t\partial t + k_a[B]_t[Y]_t\delta t$$ (Supplementary Equation 1)

$$[B]_t = [B]_0 + 2k_d[A]_t\partial t - k_a[B]_t[Y]_t\delta t + k_d[C]_t - [K_a][B]_t[X]_t\partial t$$ (Supplementary Equation 2)

$$[C]_t = [C]_0 + k_a[B]_t[X]_t\delta t - 2k_d[C]_t + k_a[D]_t[Y]_t\partial t$$ (Supplementary Equation 3)

$$[D]_t = [D]_0 + k_d[C]_t\delta t - k_a[D]_t[Y]_t\delta t + 2k_d[E]_t\delta t - k_a[D]_t[X]_t\partial t$$ (Supplementary Equation 4)

$$[E]_t = [E]_0 + k_a[D]_t[X]_t\delta t - 2k_d[E]_t\partial t$$ (Supplementary Equation 5)

$$[X]_t = [X]_0 + k_d[C]_t\partial t - k_a[B]_t[X]_t\delta t + 2k_d[E]_t\partial t - k_a[D]_t[X]_t\partial t$$ (Supplementary Equation 6)

$$[Y]_t = [Y]_0 + 2k_d[A]_t\partial t - k_a[B]_t[Y]_t\delta t + k_d[C]_t\partial t - k_a[D]_t[Y]_t\partial t$$ (Supplementary Equation 7)

A set of simulated data was created in Microsoft Excel according to the formulae given above, where the values of $[A]_t$ and $[X]_t$ are calculated from their signal intensities in $^1$H

NMR spectra, and the initial values of $k_a$ and $k_d$ are estimated. Time increments were used that are a minimum of four times smaller than those used to collect the experimental data. The percentage intensities of bound and free **A** were calculated from the simulated concentrations of the different species Supplementary Equations 8 and 9.

$$\% \, bound = \frac{2A + C}{2A + C + Y} \times 100$$ (Supplementary Equation 8)

$$\% \, free = \frac{Y}{2A + C + Y} \times 100$$ (Supplementary Equation 9)

Once the simulated data were created, according to estimated values of $k_a$ and $k_d$, they were fitted to the experimental data using Excel's Solver package. It was assumed that each value had the same uncertainty therefore the unweighted least squares method was used. Solver was then used to minimise the value of the sum of the squared residuals ($S$), thereby fitting the simulated data to the experimental data. A plot of the simulated data was compared with the experimental data to ensure a suitable fit. The derived dissociation rates $k_d$ are shown in Supplementary Table 2.

| **Pre-Catalyst** | **Rate of dissociation of A / $s^{-1}$** |
|---|---|
| **1** | 9.8 ± 0.2 |
| **3** | 6.6 ± 0.1 |
| **4** | 14.6 ± 0.3 |
| **5** | 31.6 ± 0.4 |
| **8** | 5.5 ± 0.1 |
| **9** | 3.9 ± 0.1 |
| **10** | 3.4 ± 0.1 |
| **11** | 3.6 ± 0.1 |
| **12** | 4.0 ± 0.1 |
| **13** | 3.7 ± 0.1 |
| **14** | 7.7 ± 0.2 |
| **15** | 3.8 ± 0.1 |
| **16** | 15 ± 0.2 |
| **17** | 10.5 ± 0.1 |
| **18** | 14.9 ± 0.2 |
| **19** | 9.6 ± 0.1 |
| **20** | 12.9 ± 0.2 |
| **21** | 2.7 ± 0.1 |

Supplementary Table 2: Rate of loss of **A** from [Ir(H)$_2$(NHC)(**A**)$_3$] formed from [IrCl(COD)(NHC)]. Errors are fitting errors calculated using a jackknife resampling technique across 10 points.

## Relationship between Rate and TEP

There appears to be a linear relationship between the TEP values for the NHC ligands and the rate of ligand dissociation from [Ir[Ir(H)$_2$(NHC)(**A**)$_3$]. As expected, this indicates that more electron donating ligands exhibit a faster dissociation rate. In contrast, catalyst **16**, which contains *tert*-butyl substituents on the aryl ring of the NHC, exhibits a faster rate of ligand loss than its TEP would predict which we attribute to the steric effects.

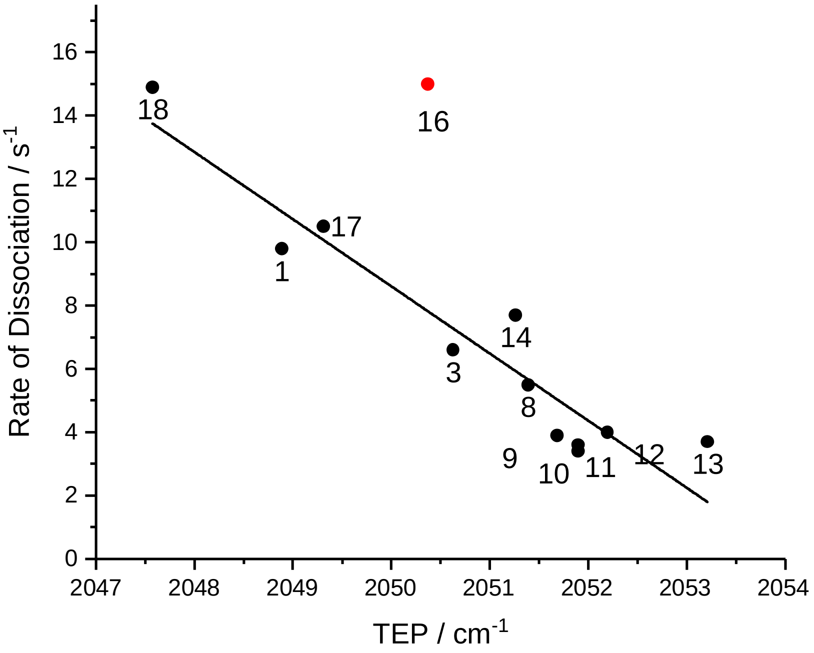


Supplementary Figure 45: Effect of *para* substituted NHC ligands as a function of their TEP on the rate of dissociation of **A** from [Ir(H)$_2$(NHC)(**A**)$_3$]

### $T_1$ relaxation values for equatorially bound A at 243 K

The $T_1$ relaxation values for equatorially bound ligand **A** at 243 K were determined using inversion recovery methods. This data is summarised in Supplementary Table 3 and Supplementary Figure 15.

| Pre-Catalyst | H-2 / $s^{-1}$ | H-4 / $s^{-1}$ |
|---|---|---|
| **1** | 1.91 | 3.12 |
| **3** | 1.89 | 3.62 |
| **4** | 1.62 | 4.28 |
| **5** | 1.64 | 4.2 |
| **8** | 2.61 | 4.58 |
| **9** | 2.55 | 4.58 |
| **10** | 1.93 | 5.07 |
| **11** | 1.92 | 5.64 |
| **12** | 2.92 | 2.50 |
| **13** | 2.94 | 4.32 |
| **14** | 2.29 | 4.33 |
| **15** | 1.78 | 2.24 |
| **16** | 1.23 | 2.84 |
| **17** | 2.2 | 4.76 |
| **18** | 2.62 | 5.20 |
| **19** | 1.23 | 4.30 |
| **20** | 1.03 | 3.61 |
| **21** | 2.42 | 4.16 |

Supplementary Table 3: $T_1$ relaxation values for H-2 and H-5 for equatorially bound **A** at 243 K using [IrCl(COD)(NHC)] (5mM) in methanol-$d_4$ under 3 bar $p$-$H_2$

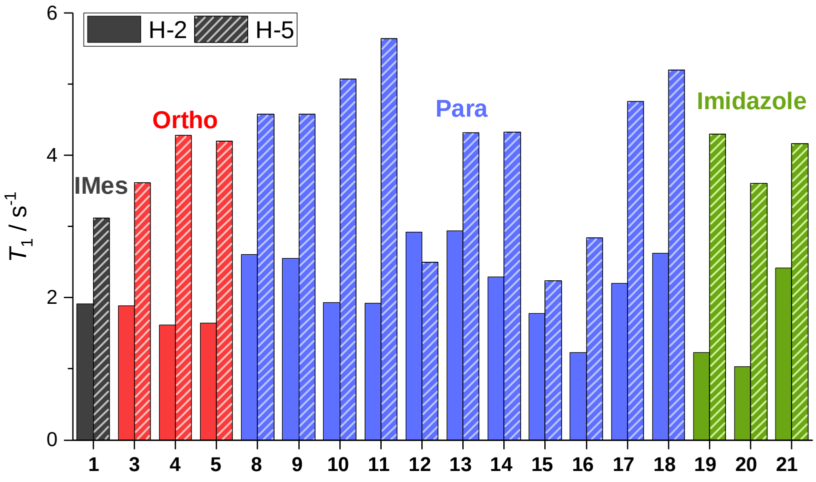


Supplementary Figure 46: $T_1$ relaxation values for H-2 and H-5 for equatorially bound **A** at 243 K using [IrCl(COD)(NHC)] (5mM) in methanol-$d_4$ under 3 bar $p$-$H_2$

## Oscillation Frequency

The selective excitation of the hydride resonance in the corresponding $[Ir(H)_2(NHC)(\mathbf{A})_3]Cl$ complexes using a selective 1D cosy pulse sequence was used conducted as outlined in Supplementary Figure 16. The delay constant, d4, was varied over a minimum of 15 increments between 0.0078 and 0.021 s. Relative integrals values of H-2 of equatorially bound **A** were plotted and fitted to give the delay constant maxima. These values are plotted in Supplementary Figure 17. It is well established that this oscillation frequency is dependent upon the size of the *J* coupling between the nuclei.[23] Variants of this technique have been previously used to measure hyperpolarized couplings previously.[24]

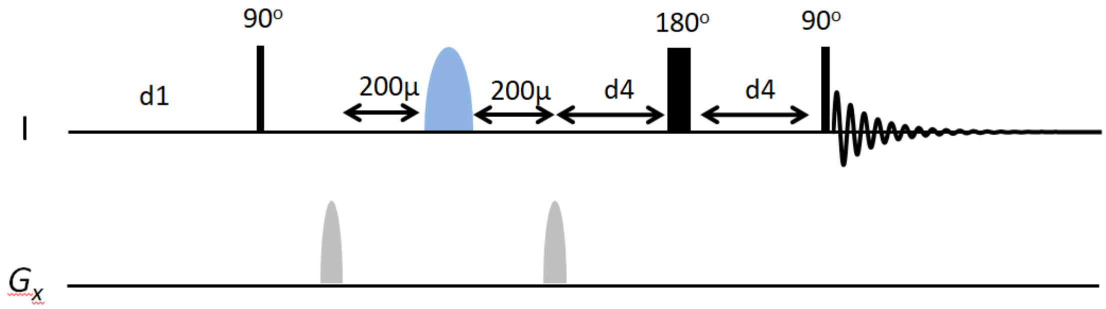


Supplementary Figure 47: The selcogp pulse sequence

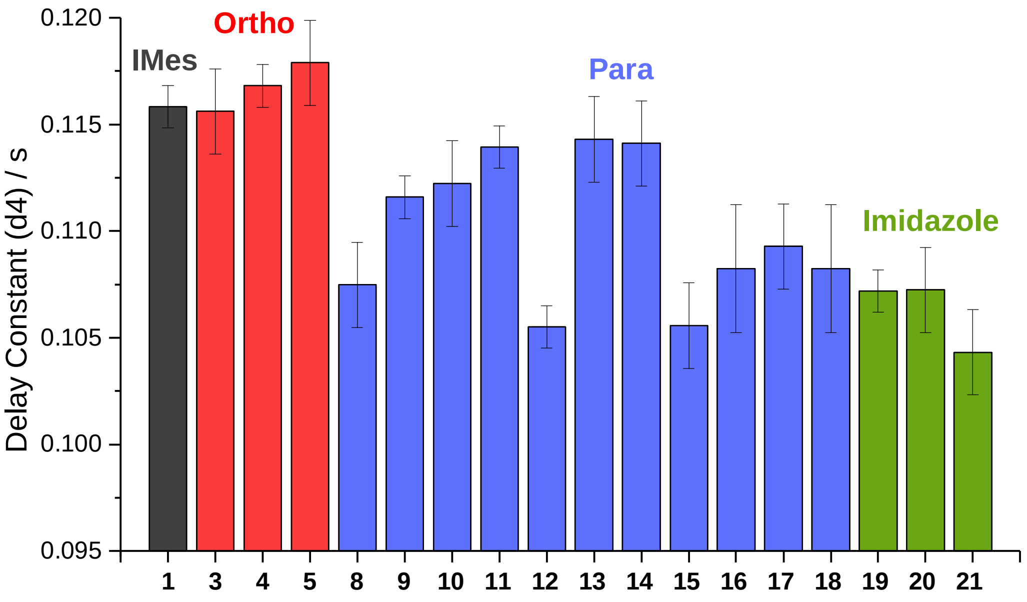


Supplementary Figure 48: Delay constant (d4) with which gave the maximum signal for H-2 of equatorially bound **A** in the selcogp experiment when selectively exciting the hydride resonance in $[Ir(H)_2(NHC)(\mathbf{A})_3]Cl$.

## Substrate Polarization and $T_1$ Relaxation Data

3-nitro pyridine (B)

| Pre-Catalyst | H-2 / % | H-4 / % | H-5 / % | H-6 /% | Average |
|---|---|---|---|---|---|
| **1** | 1.98 | 1.87 | 1.02 | 1.96 | 1.71 |
| **9** | 0.56 | 0.64 | 0.30 | 0.48 | 0.50 |
| **12** | 1.49 | 0.95 | 0.39 | 0.93 | 0.94 |
| **16** | 1.03 | 0.86 | 0.44 | 0.92 | 0.82 |
| **18** | 4.52 | 4.02 | 2.20 | 4.35 | 3.77 |
| **19** | 0.82 | 0.75 | 0.41 | 0.77 | 0.69 |
| **21** | 2.40 | 1.93 | 1.02 | 1.94 | 1.82 |
| $d_{28}$-**18** | 5.90 | 4.96 | 3.23 | 6.32 | 5.10 |

Supplementary Table 4: Polarization (%) for 3-nitro pyridine (**B**) (4 eq.) using [IrCl(COD)(NHC)] (5mM) in methanol-$d_4$ under 3 bar $p$-$H_2$

| Pre-Catalyst | H-2 / s | H-4 / s | H-5 / s | H-6 /s |
|---|---|---|---|---|
| **1** | 17.6 | 13.7 | 8.8 | 6.6 |
| **9** | 13.8 | 6.4 | 5.0 | 11.7 |
| **12** | 10.1 | 8.1 | 3.6 | 3.5 |
| **16** | 12.9 | 11.8 | 8.8 | 5.7 |
| **18** | 18.1 | 12.6 | 9.2 | 8.2 |
| **19** | 17.3 | 10.6 | 4.8 | 12.4 |
| **21** | 13.7 | 10.8 | 6.1 | 5.0 |

Supplementary Table 5: $T_1$ relaxation values for 3-nitro pyridine (**B**) (4 eq.) using [IrCl(COD)(NHC)] (5mM) in methanol-$d_4$ under 3 bar $H_2$

3-Trifluoromethyl pyridine (C)

| Pre-Catalyst | H-2 / % | H-4 / % | H-5 / % | H-6 /% | Average |
|---|---|---|---|---|---|
| **1** | 2.45 | 1.78 | 0.67 | 1.44 | 1.59 |
| **9** | 3.51 | 1.93 | 0.98 | 1.56 | 2.00 |
| **12** | 2.36 | 1.37 | 0.85 | 1.30 | 1.47 |
| **16** | 1.06 | 0.90 | 0.29 | 0.58 | 0.71 |
| **18** | 0.92 | 0.61 | 0.71 | 0.40 | 0.66 |
| **19** | 1.19 | 0.81 | 0.41 | 0.71 | 1.78 |
| **21** | 4.27 | 2.52 | 1.41 | 2.39 | 2.65 |
| $d_{22}$-**21** | 10.88 | 7.90 | 4.66 | 8.91 | 8.08 |

Supplementary Table 6: Polarization (%) for 3-trifluoromethyl pyridine (**C**) (4 eq.) using [IrCl(COD)(NHC)] (5mM) in methanol-$d_4$ under 3 bar $p$-$H_2$

| **Pre-Catalyst** | **H-2 / s** | **H-4 / s** | **H-5 / s** | **H-6 /s** |
|---|---|---|---|---|
| **1** | 5.6 | 6.5 | 6.7 | 6.0 |
| **9** | 5.4 | 5.9 | 6.9 | 5.5 |
| **12** | 4.5 | 5.6 | 4.1 | 3.9 |
| **16** | 6.6 | 6.8 | 6.7 | 4.5 |
| **18** | 9.7 | 9.1 | 10.9 | 6.0 |
| **19** | 8.5 | 7.6 | 9.2 | 7.0 |
| **21** | 6.7 | 7.4 | 6.0 | 5.1 |

Table 7: $T_1$ relaxation values for 3-trifluoromethyl pyridine (**C**) (4 eq.) using [IrCl(COD)(NHC)] (5mM) in methanol-$d_4$ under 3 bar $H_2$

Picoline (D)

| **Pre-Catalyst** | **H-2 / %** | **H-4 / %** | **H-5 / %** | **H-6 /%** | **Average** |
|---|---|---|---|---|---|
| **1** | 1.87 | 1.93 | 0.81 | 1.65 | 1.40 |
| **9** | 5.22 | 6.33 | 0.82 | 6.04 | 4.60 |
| **12** | 7.30 | 5.53 | 2.17 | 6.50 | 5.38 |
| **16** | 0.83 | 0.76 | 0.14 | 0.67 | 0.60 |
| **18** | 1.93 | 1.88 | 0.51 | 3.29 | 1.90 |
| **19** | 0.36 | 0.48 | 0.07 | 0.41 | 0.33 |
| **21** | 2.72 | 2.09 | 0.52 | 2.39 | 1.93 |
| ***d*$_{22}$-12** | 7.04 | 6.01 | 2.92 | 7.20 | 5.79 |

Supplementary Table 8: Polarization (%) for 3-picoline (**D**) (4 eq.) using [IrCl(COD)(NHC)] (5mM) in methanol-$d_4$ under 3 bar $p$-$H_2$

| **Pre-Catalyst** | **H-2 / s** | **H-4 / s** | **H-5 / s** | **H-6 /s** |
|---|---|---|---|---|
| **1** | 5.0 | 5.3 | 6.7 | 3.7 |
| **9** | 3.9 | 3.9 | 4.7 | 3.3 |
| **12** | 4.4 | 4.3 | 5.6 | 3.5 |
| **16** | * | 3.8 | 4.3 | * |
| **18** | 4.6 | 5.3 | 6.6 | 3.4 |
| **19** | * | 4.4 | 5.8 | * |
| **21** | 4.4 | 4.6 | 5.5 | 3.6 |

*unable to be determined due to rapid deuteration.

Supplementary Table 9: $T_1$ relaxation values for 3-picoline (**D**) (4 eq.) using [IrCl(COD)(NHC)] (5mM) in methanol-$d_4$ under 3 bar $H_2$

Methoxy pyridine (E)

| Pre-Catalyst | H-2 / % | H-4 / % | H-5 / % | H-6 /% | Average |
|---|---|---|---|---|---|
| **1** | 3.0 | 4.1 | 3.9 | 4.0 | 3.8 |
| **9** | 6.4 | 3.7 | 7.5 | 4.7 | 5.6 |
| **12** | 3.1 | 0.8 | 2.9 | 3.6 | 2.6 |
| **16** | 1.8 | 0.8 | 1.5 | 1.4 | 1.4 |
| **18** | 2.8 | 3.4 | 2.7 | 3.5 | 3.1 |
| **19** | 1.9 | 0.7 | 1.9 | 2.1 | 1.7 |
| **21** | 6.9 | 2.0 | 6.9 | 5.8 | 5.4 |
| ***d*₁₆-9** | 11.3 | 7.8 | 16.0 | 8.0 | 10.8 |

Supplementary Table 10: Polarization (%) for 3-methoxy pyridine (**E**) (4 eq.) using [IrCl(COD)(NHC)] (5mM) in methanol-$d_4$ under 3 bar $p$-$H_2$

| Pre-Catalyst | H-2 / s | H-4 / s | H-5 / s | H-6 /s |
|---|---|---|---|---|
| **1** | | | | |
| **9** | 3.5 | 3.8 | 3.4 | 2.3 |
| **12** | 4.1 | - | 3.3 | 2.9 |
| **16** | 3.0 | 3.1 | 3.5 | 3.0 |
| **18** | 4.3 | 4.5 | 4.1 | 3.6 |
| **19** | - | 5.2 | 4.3 | - |
| **21** | 4.4 | - | 4.2 | 3.4 |

*unable to be determined due to rapid deuteration.

Supplementary Table 11: $T_1$ relaxation values for 3-methoxy pyridine (**E**) (4 eq.) using [IrCl(COD)(NHC)] (5mM) in methanol-$d_4$ under 3 bar $H_2$

Dimethylamino pyridine (F)

| Pre-Catalyst | H-2 / % | H-4 / % | H-5 / % | H-6 /% | Average |
|---|---|---|---|---|---|
| **1** | 2.68 | 2.42 | 0.27 | 1.68 | 1.76 |
| **9** | 6.30 | 3.52 | 0.41 | 4.21 | 3.61 |
| **12** | 1.43 | 0.98 | 0.16 | 1.07 | 0.91 |
| **16** | 0.34 | 0.36 | 0.09 | 0.21 | 0.25 |
| **18** | 2.31 | 1.23 | 0.03 | 1.67 | 1.31 |
| **19** | 1.07 | 1.15 | 0.00 | 0.52 | 0.69 |
| **21** | 0.13 | 0.38 | 0.12 | 0.08 | 0.18 |
| $d_{16}$-**9** | 13.06 | 4.46 | 8.00 | 6.85 | 8.09 |

Supplementary Table 12: Polarization (%) for 3-dimethylamino pyridine (**F**) (4 eq.) using [IrCl(COD)(NHC)] (5mM) in methanol-$d_4$ under 3 bar $p$-$H_2$

$T_1$ relaxation times of 3-dimethylamino pyridine (F)

| Pre-Catalyst | H-2 / s | H-4 / s | H-5 / s | H-6 /s |
|---|---|---|---|---|
| **1** | 2.1 | 1.8 | 2.9 | 1.6 |
| **9** | 2.3 | 2.1 | 2.5 | 1.6 |
| **12** | 2.2 | 2.0 | 2.7 | 2.0 |
| **16** | 1.5 | 2.2 | 2.2 | 2.1 |
| **18** | 2.1 | 2.1 | 2.6 | 2.8 |
| **19** | 2.4 | 2.5 | 2.6 | 2.9 |
| **21** | 2.3 | 2.1 | 2.1 | 1.9 |

Supplementary Table 13: $T_1$ relaxation values for 3-dimethylamino pyridine (**F**) (4 eq.) using [IrCl(COD)(NHC)] (5mM) in methanol-$d_4$ under 3 bar $H_2$

**Optimisation of Polarization of pyridine-$^{15}$N**

| Pre-Catalyst | Pre-Catalyst Concentration / mM | Initial Pyridine-$^{15}$N concentration / mM | $p$-$H_2$ pressure / bar | Polarization / % |
|---|---|---|---|---|
| **1** | 2.5 | 25 | 3 | 7.1 ± 0.2 |
| **9** | 2.5 | 25 | 3 | 5.8 ± 0.4 |
| **12** | 2.5 | 25 | 3 | 10.6 ± 0.2 |
| **16** | 2.5 | 25 | 3 | 1.6 ± 0.1 |
| **18** | 2.5 | 25 | 3 | 1.8 ± 0.1 |
| **19** | 2.5 | 25 | 3 | 4.1 ± 0.1 |
| **21** | 2.5 | 25 | 3 | 11.0 ± 0.3 |
| ***d*₂₂-21** | 2.5 | 25 | 3 | 15.5 ± 0.8 |
| ***d*₂₂-21** | 2.5 | 25 | 5 | 19.5 ± 0.6 |
| ***d*₂₂-21** | 0.25 | 2.5 | 3 | 19.9 ± 0.7 |
| ***d*₂₂-21** | 0.25 | 2.5 | 5 | 24.9 ± 0.6 |
| ***d*₂₂-21** | 2.5 | 6.25* | 5 | 42.3 ± 0.6 |

Supplementary Table 14: Data of the polarization of pyridine-$^{15}$N using a varying pre-catalysts, concentrations and $p$-$H_2$ pressure.*Contains 18.75 mM of pyridine-$d_5$ as a co-ligand. Errors are standard error across a minimum of 5 observations.

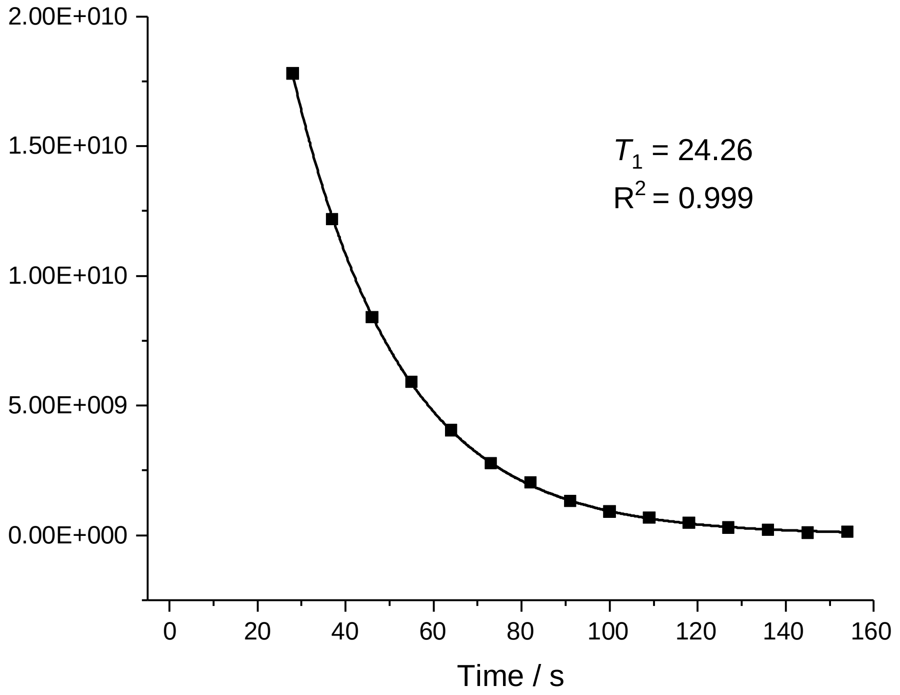


Supplementary Figure 49: Plot of hyperpolarized lifetime of pyridine-$^{15}$N (25 mM) in the presence of **1** (2.5 mM) in methanol-$d_4$ under 3 bar $p$-$H_2$ at 298 K.

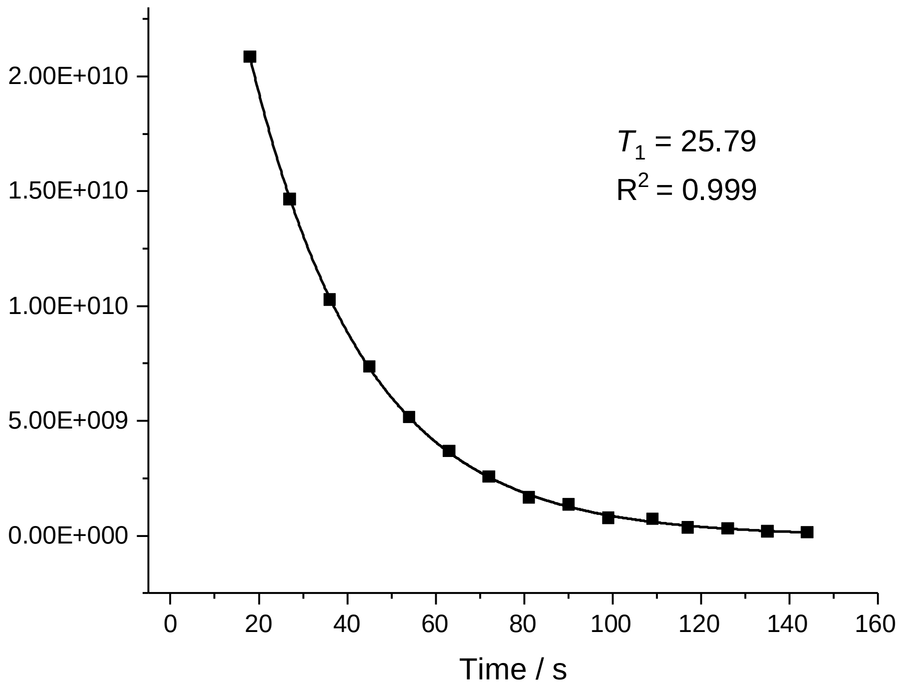


Supplementary Figure 50: Plot of hyperpolarized lifetime of pyridine-$^{15}$N (25 mM) in the presence of **21** (2.5 mM) in methanol-$d_4$ under 3 bar $p$-$H_2$ at 298 K.

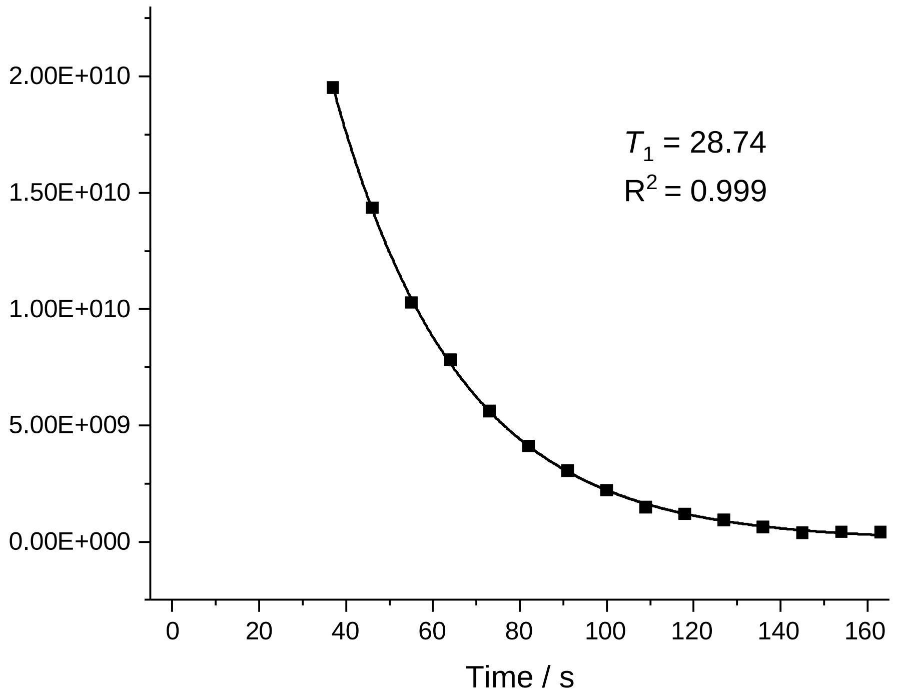


Supplementary Figure 51: Plot of hyperpolarized lifetime of pyridine-$^{15}$N (25 mM) in the presence of ***d*~22~-21** (2.5 mM) in methanol-$d_4$ under 3 bar $p$-$H_2$ at 298 K.

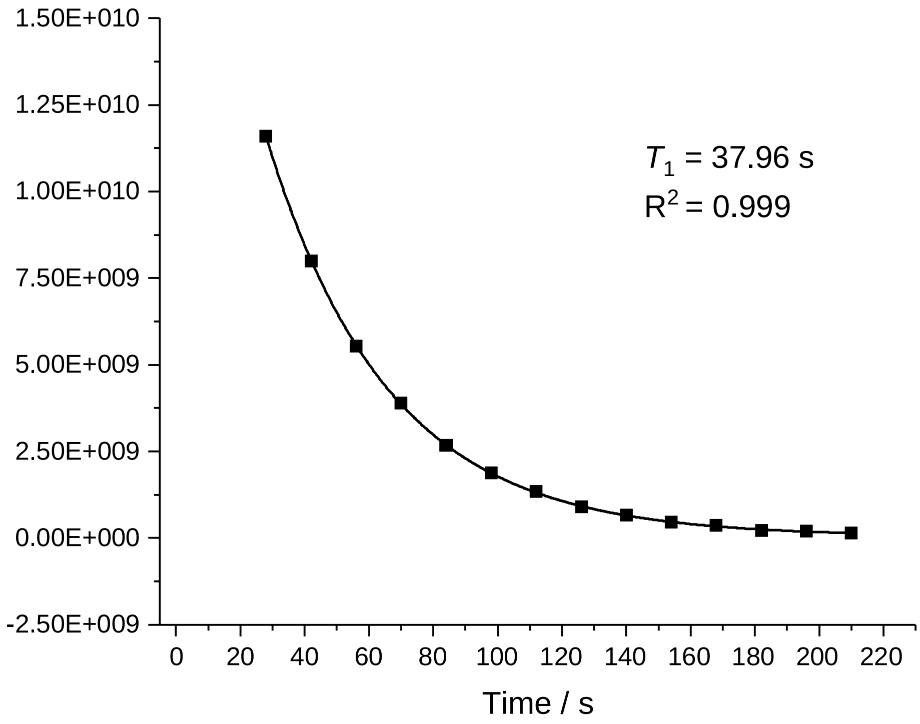


Supplementary Figure 52: Plot of hyperpolarized lifetime of pyridine-$^{15}N$ (6.25 mM) in the presence of ***d*₂₂-21** (2.5 mM) and pyridine-$d_5$ (18.75 mM) in methanol-$d_4$ under 3 bar $p$-$H_2$ at 298 K.

| Pre-Catalyst | Rate of Dissociation / $s^{-1}$ |
|---|---|
| **1** | 11.2 ± 0.1 |
| **21** | 4.8 ± 0.1 |
| ***d*$_{22}$-21** | 4.8 ± 0.1 |

Supplementary Table 15: Rate constant for dissociation of pyridine-$^{15}N$ from $[Ir(H)_2(NHC)(Py)_3]$. Errors are fitting errors calculated using a jackknife resampling technique across 10 points.

As has been previously shown,[25] these values can be further improved by decreasing the sample concentration. For a sample containing 0.25 mM of ***d*$_{22}$-21** and 2.5 mM pyridine-$^{15}N$ the polarization level increased to 19.9% (>60000-fold signal gain). Further improvements were achieved by increasing the pressure of $p$-$H_2$ to 5 bar, which resulted in 24.9% $^{15}N$ polarization (>75000-fold signal gain).

## Optimisation of Polarization of G

| Pre-Catalyst | Pre-Catalyst Concentration / mM | Initial G concentration / mM | $p$-$H_2$ pressure / bar | Polarization / % |
|---|---|---|---|---|
| **1** | 5 | 20 | 3 | 2.6 ± 0.2 |
| **9** | 5 | 20 | 3 | 0.9 ± 0.1 |
| **12** | 5 | 20 | 3 | 0.4 ± 0.1 |
| **16** | 5 | 20 | 3 | 5.6 ± 0.2 |
| **18** | 5 | 20 | 3 | 4.5 ± 0.4 |
| **19** | 5 | 20 | 3 | 3.5 ± 0.2 |
| **21** | 5 | 20 | 3 | 0.9 ± 0.1 |
| $d_{34}$-**16** | 5 | 20 | 3 | 8.2 ± 0.2 |
| $d_{34}$-**16** | 5 | 20 | 5 | 9.6 ± 0.3 |
| $d_{34}$-**16** | 2.5 | 10 | 3 | 8.5 ± 0.2 |
| $d_{34}$-**16** | 2.5 | 10 | 5 | 10.8 ± 0.3 |
| $d_{34}$-**16** | 2.5 | 2.5* | 5 | 25.0 ± 0.7 |

Supplementary Table 16: Data for the polarization of **G** using varying pre-catalysts, concentrations and $p$-$H_2$ pressure. *In the presence of 7.5 mM 4,5-bis(phenyl-$d_5$)-3,6-$d_2$-pyridazine[6] as a co-ligand. Errors are standard error across a minimum of 5 observations.

The rate of ligand dissociation and haptotropic shift of **G** in [Ir(H)$_2$(NHC)(**G**)$_3$] was determined using well established EXSY methods. A full description of the mechanism has been previously reported for pyridazine based substrates.[26]

| Pre-Catalyst | Rate of Dissociation / $s^{-1}$ | Rate of Haptotropic Shift / $s^{-1}$ |
|---|---|---|
| **1** | 0.20 ± 0.02 | 0.26 ± 0.03 |
| **16** | 0.46 ± 0.03 | 0.90 ± 0.05 |

Supplementary Table 17: Effect of catalysts on the rate of dissociation and rate of haptotropic shift with **G**. Errors are fitting errors calculated using a jackknife resampling technique across 10 points.

| Pre-Catalyst | Pre-Catalyst Concentration / mM | Initial G concentration / mM | Hyperpolarized Lifetime / s |
|---|---|---|---|
| **1** | 5 | 20 | 3.21 |
| **16** | 5 | 20 | 1.53 |
| $d_{34}$-**16** | 5 | 20 | 2.23 |
| $d_{34}$-**16** | 2.5 | 10 | 3.58 |
| $d_{34}$-**16** | 2.5 | 2.5* | 3.41 |

Supplementary Table 18: Data for the hyperpolarized lifetimes of **G** using varying pre-catalysts, and concentrations. *In the presence of 7.5 mM 4,5-bis(phenyl-$d_5$)-3,6-$d_2$-pyridazine[6] as a co-ligand.

## Supplementary Figures

### $^{1}$H and $^{13}$C NMR spectra of synthesized compounds

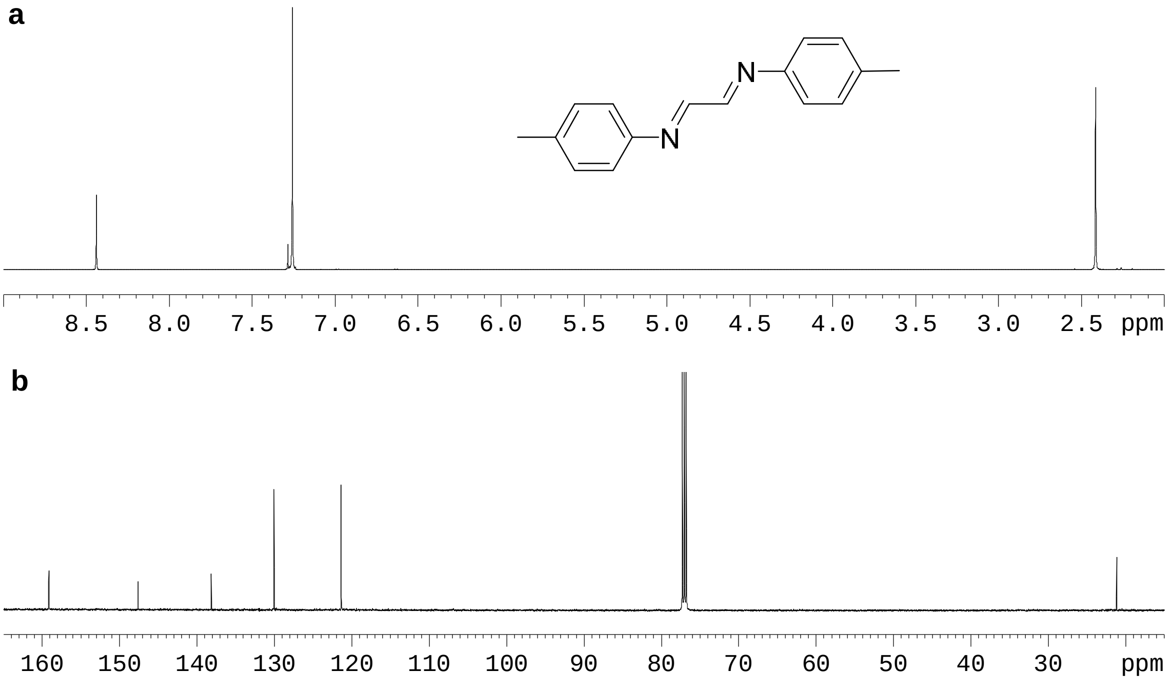


Supplementary Figure 53: NMR spectra of **S1**. **a** $^{1}$H NMR spectrum. **b** $^{13}$C NMR spectrum.

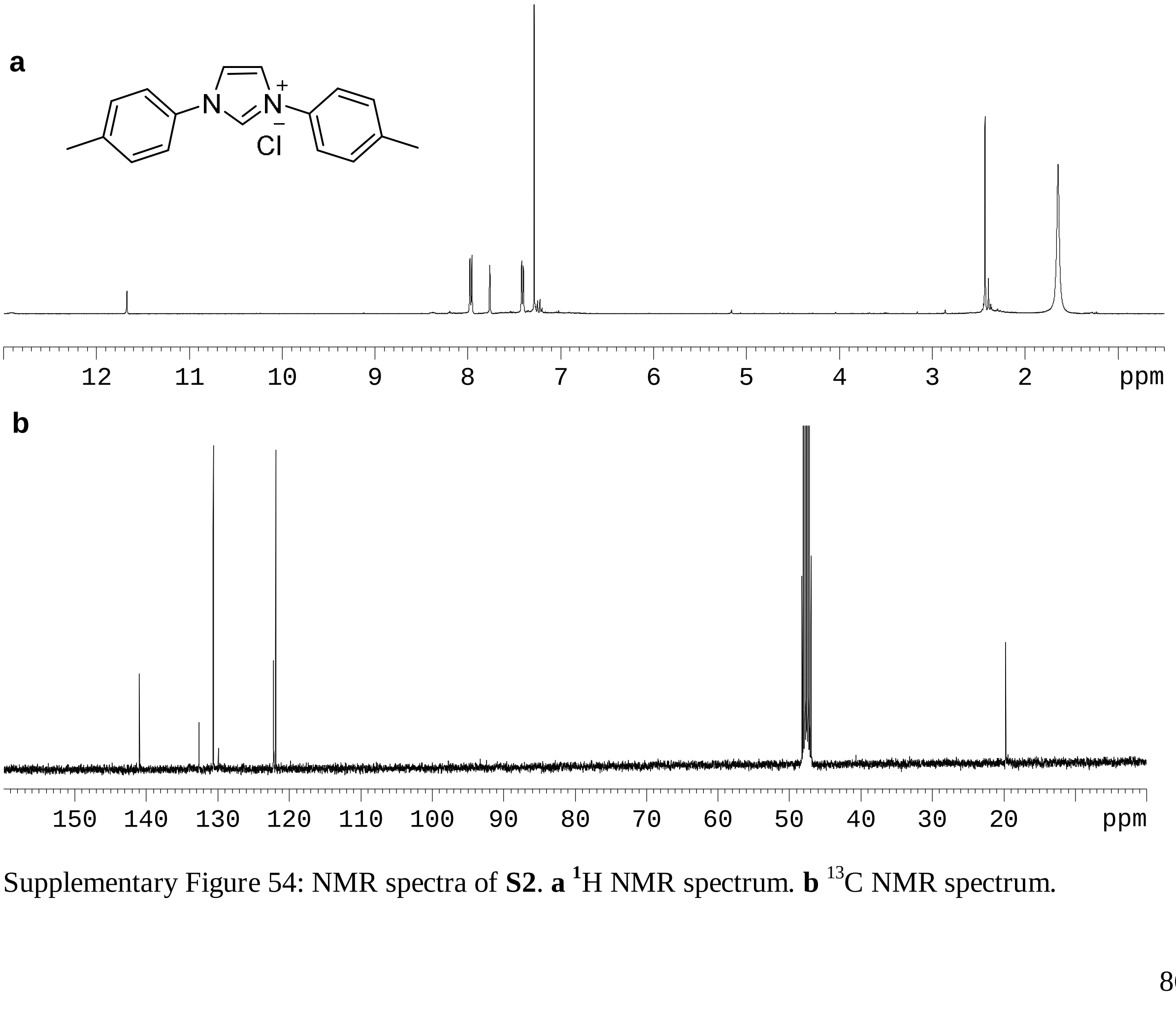


Supplementary Figure 54: NMR spectra of **S2**. **a** $^{1}$H NMR spectrum. **b** $^{13}$C NMR spectrum.

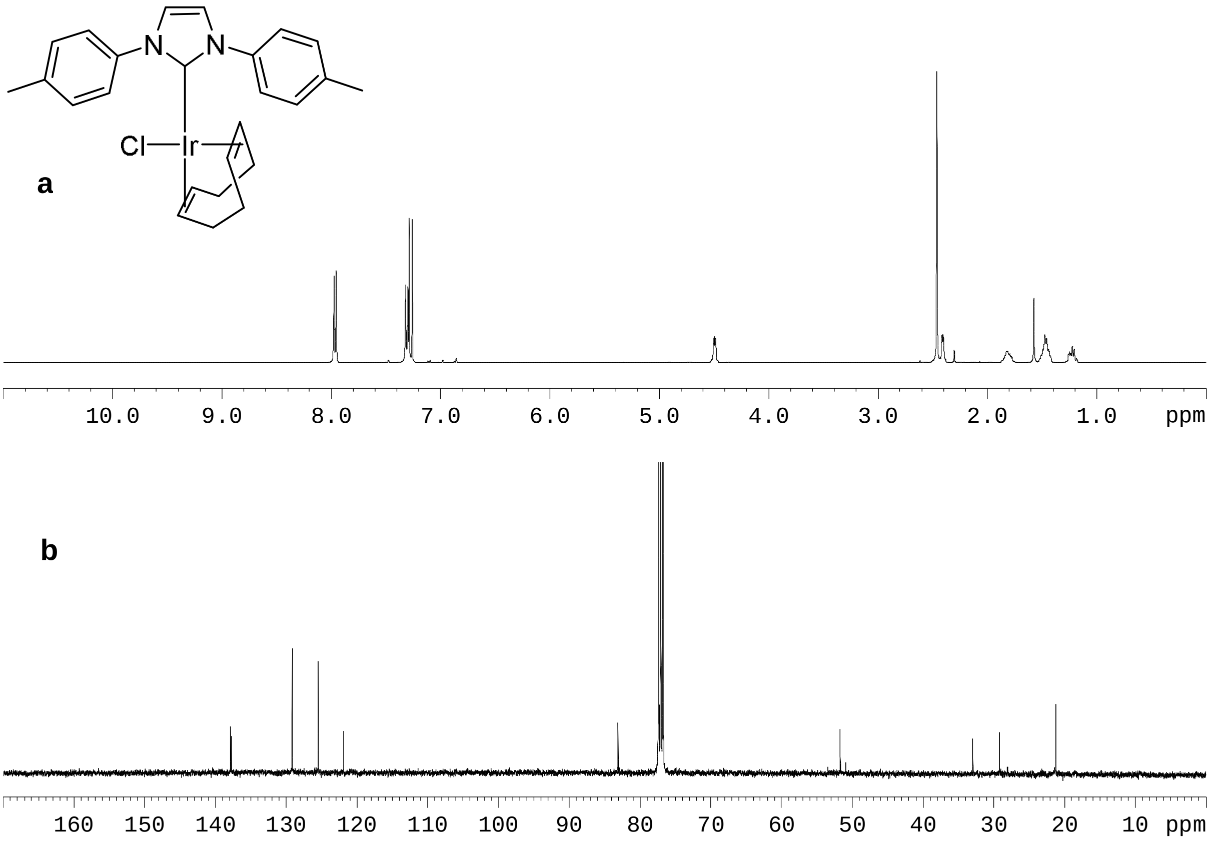


Supplementary Figure 55: NMR spectra of **2**. **a** 1H NMR spectrum. **b** $^{13}$C NMR spectrum.

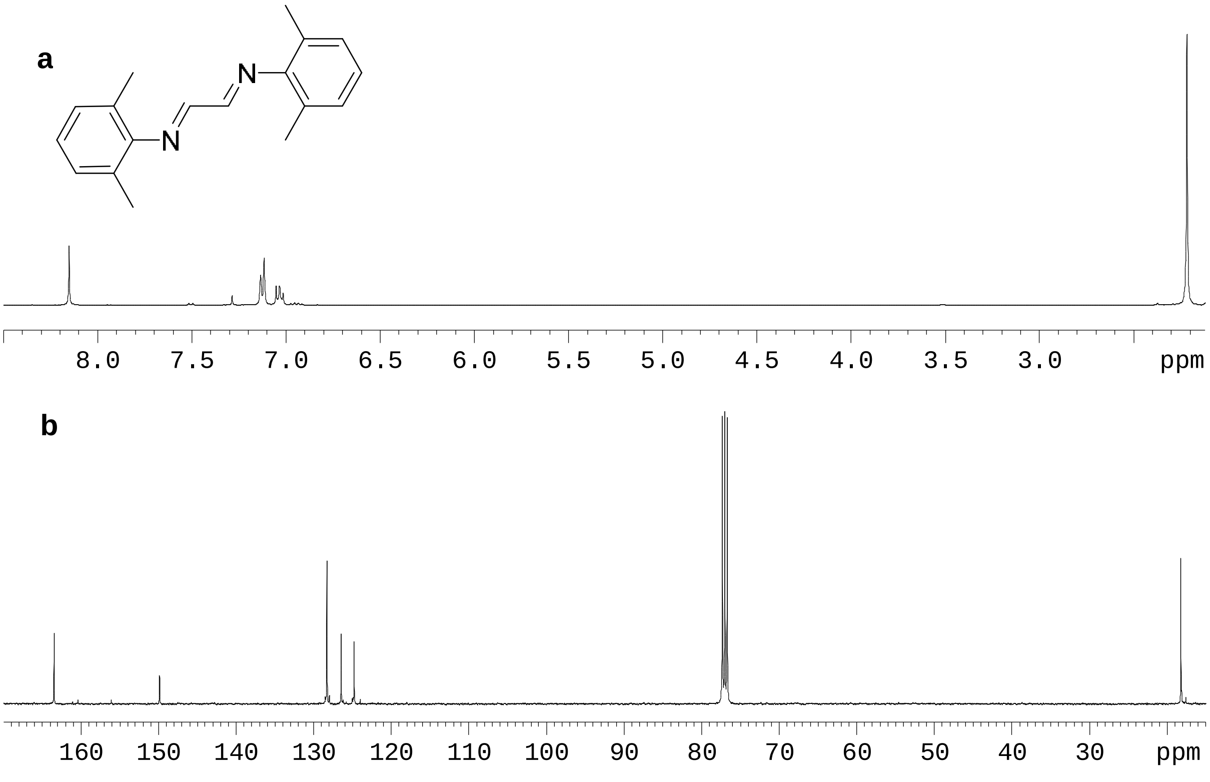


Supplementary Figure 56: NMR spectra of **S3**. **a** $^{1}$H NMR spectrum. **b** $^{13}$C NMR spectrum.

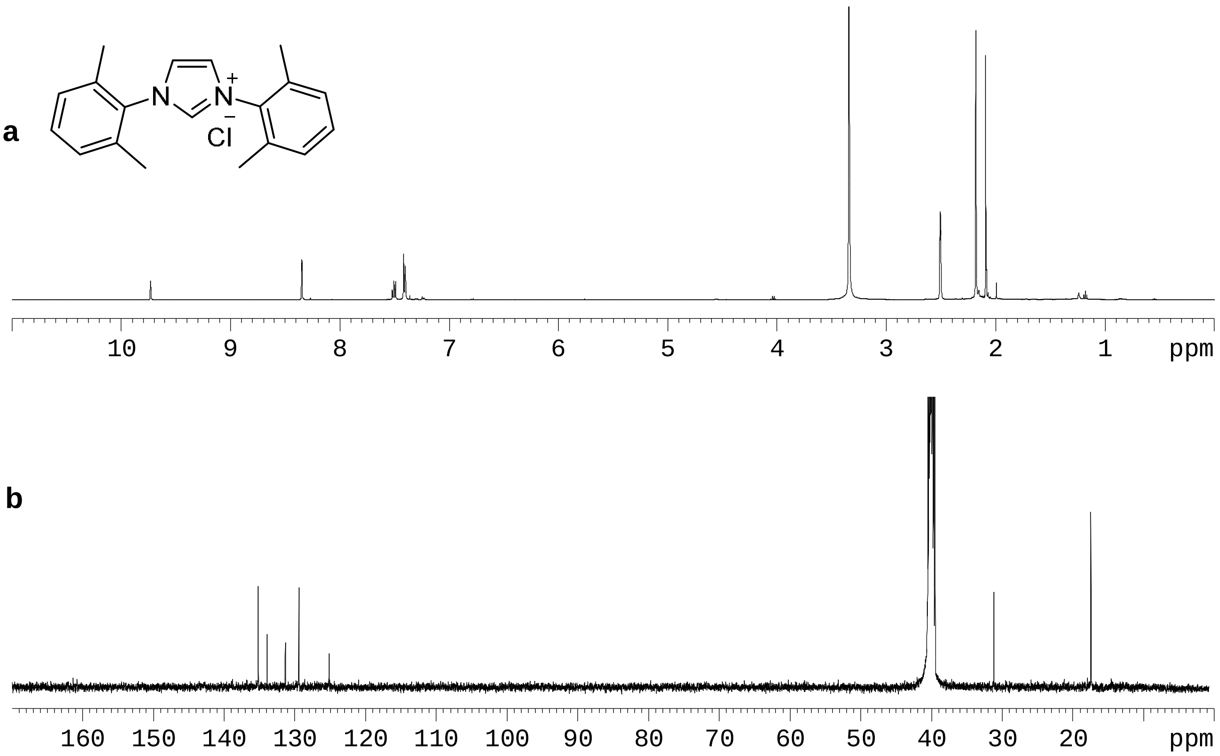


Supplementary Figure 57: NMR spectra of **S4**. **a** $^{1}$H NMR spectrum. **b** $^{13}$C NMR spectrum.

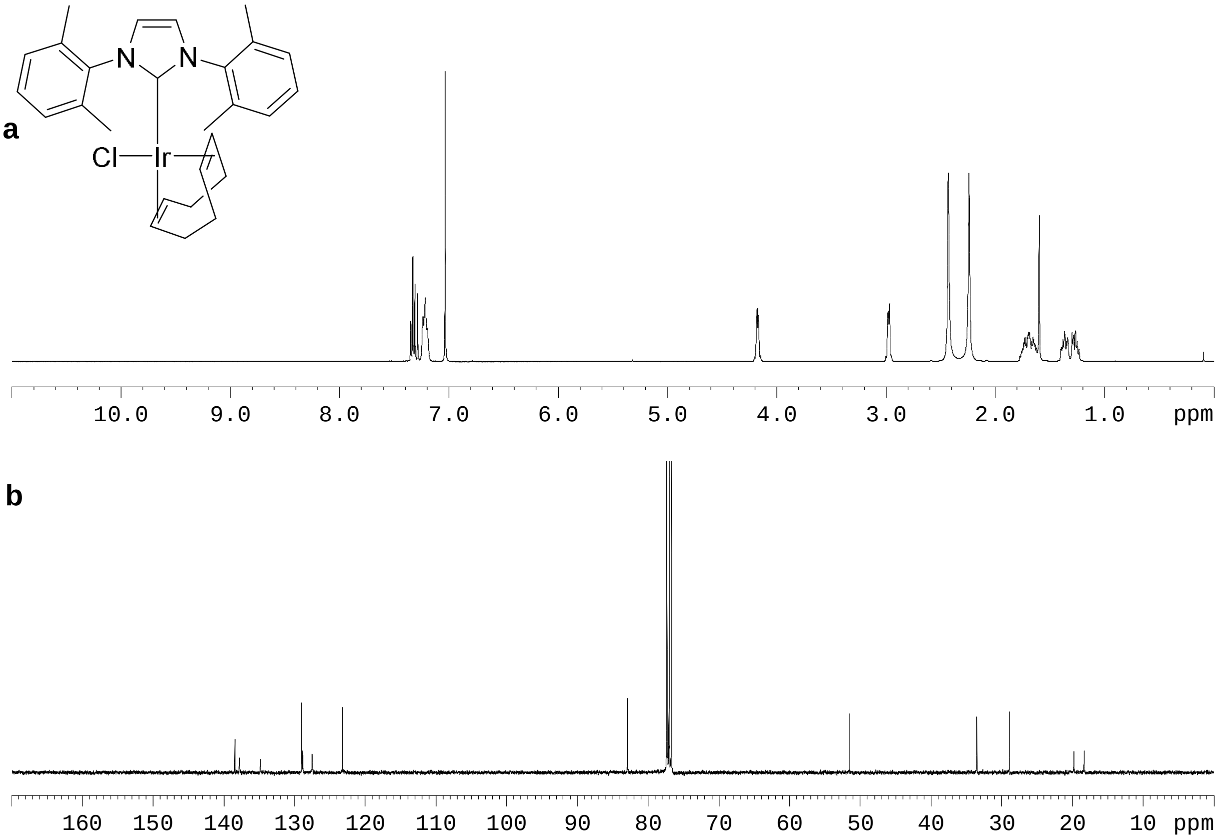


Supplementary Figure 58: NMR spectra of **3**. **a** $^{1}$H NMR spectrum. **b** $^{13}$C NMR spectrum.

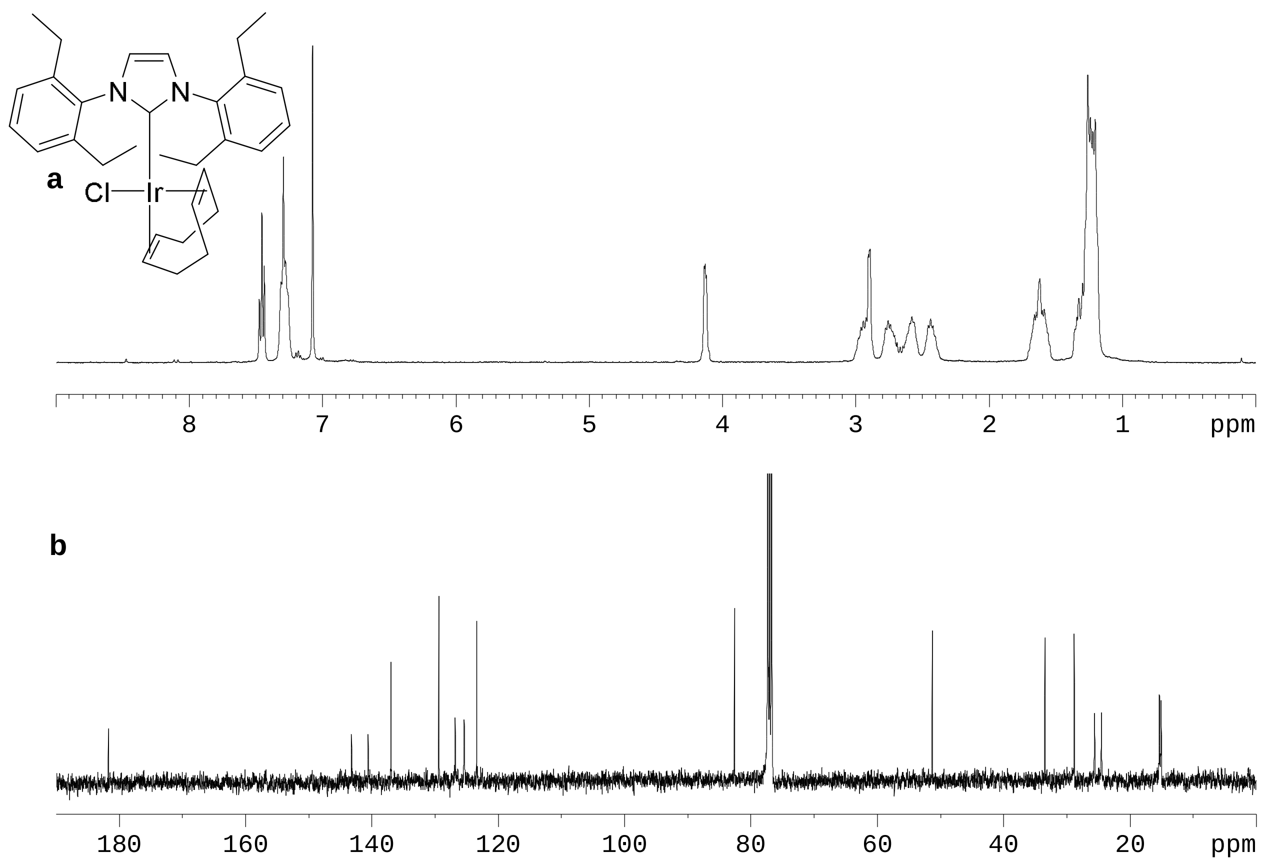


Supplementary Figure 59: NMR spectra of **4**. **a** $^{1}$H NMR spectrum. **b** $^{13}$C NMR spectrum.

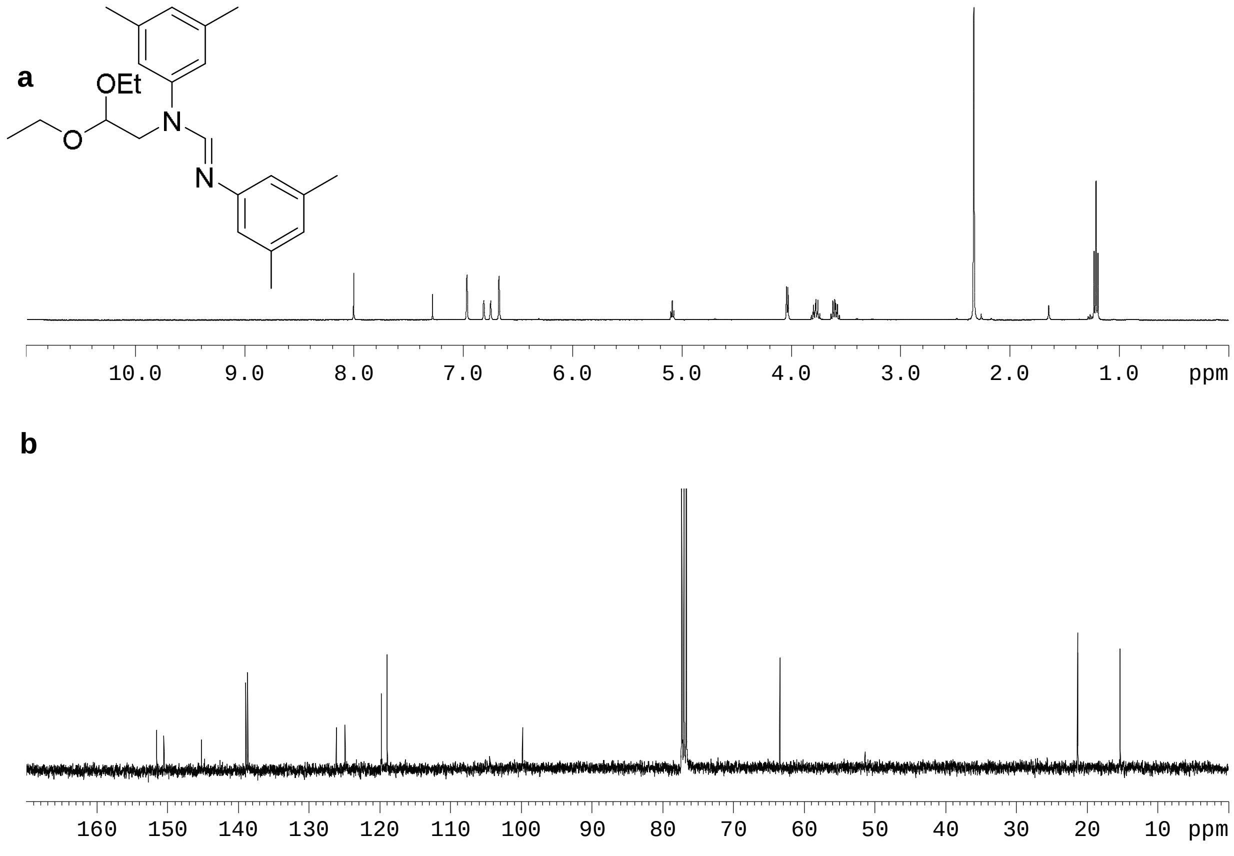


Supplementary Figure 60: NMR spectra of **S6**. **a** $^{1}$H NMR spectrum. **b** $^{13}$C NMR spectrum.

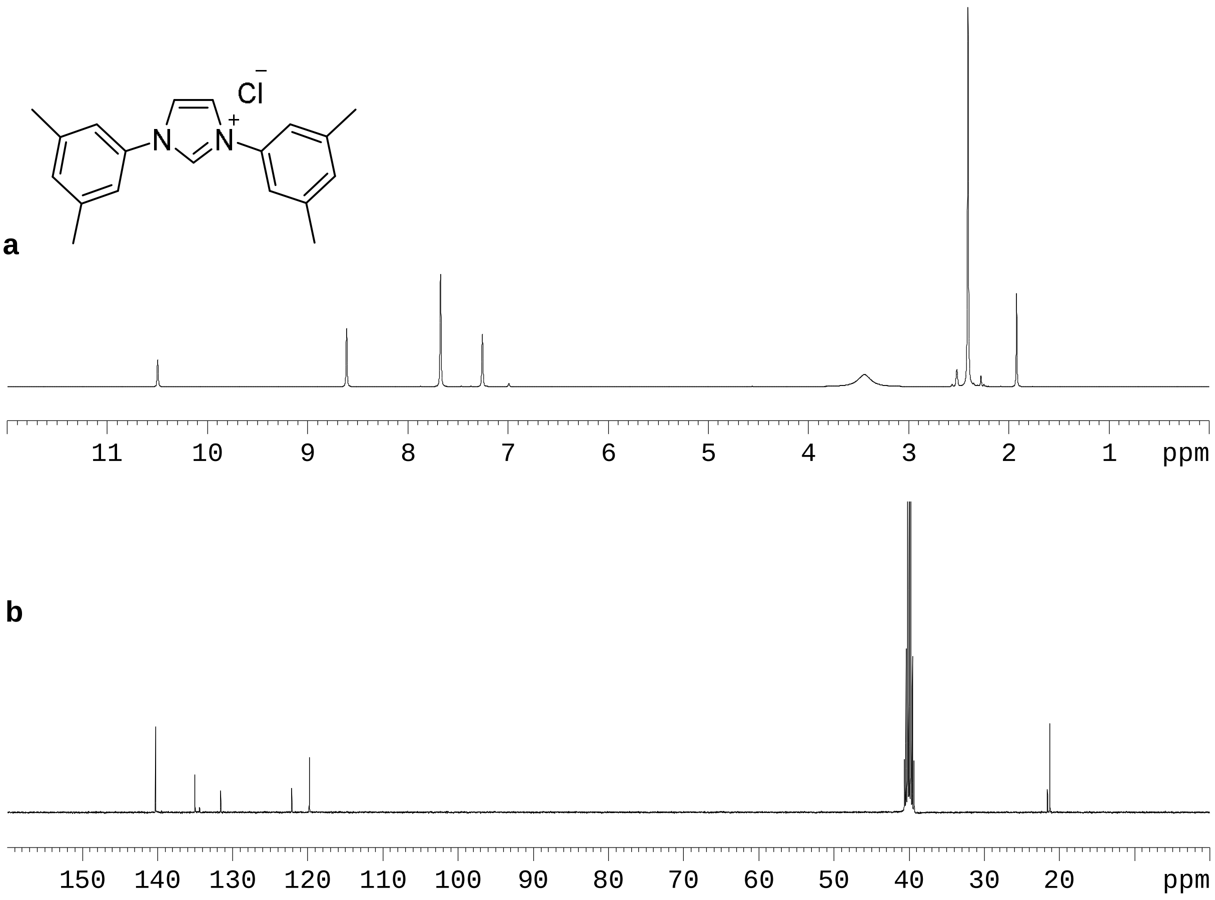


Supplementary Figure 6[1]: NMR spectra of **S8**. **a** $^{1}$H NMR spectrum. **b** $^{13}$C NMR spectrum.

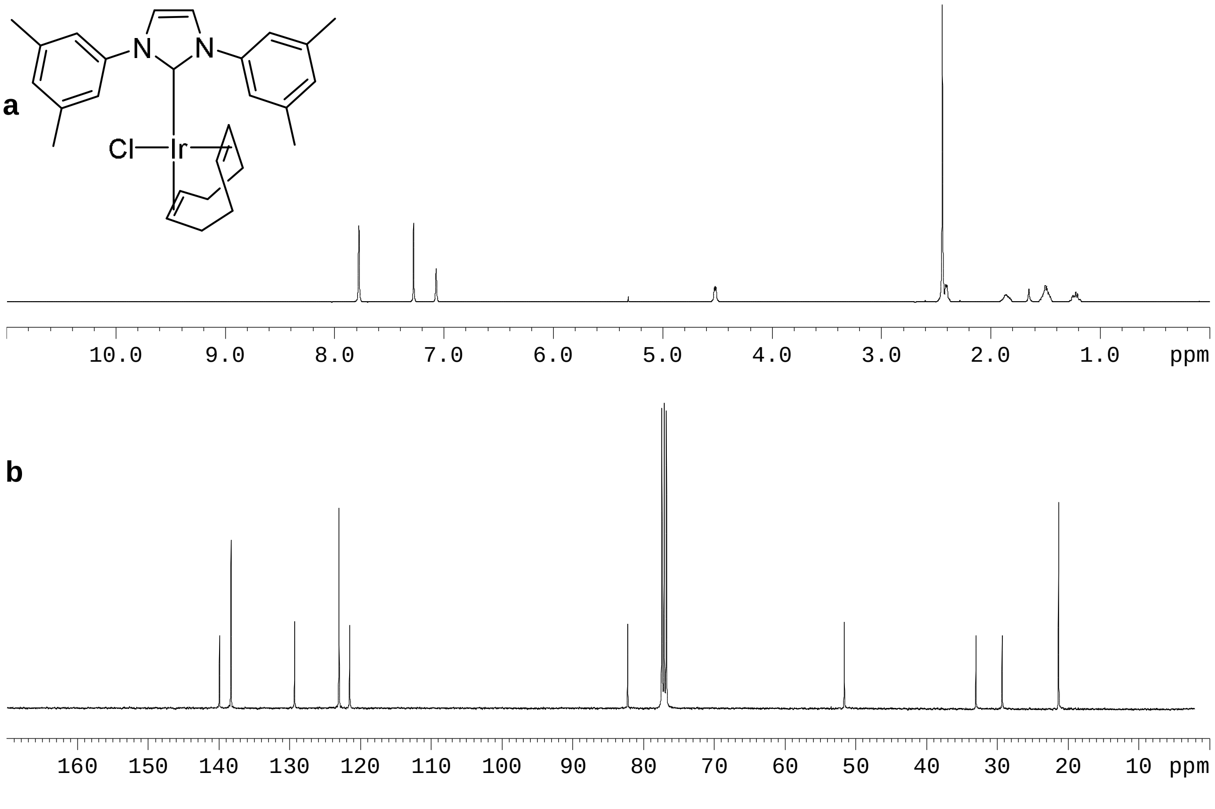


Supplementary Figure 62: NMR spectra of **6**. **a** $^{1}$H NMR spectrum. **b** $^{13}$C NMR spectrum.

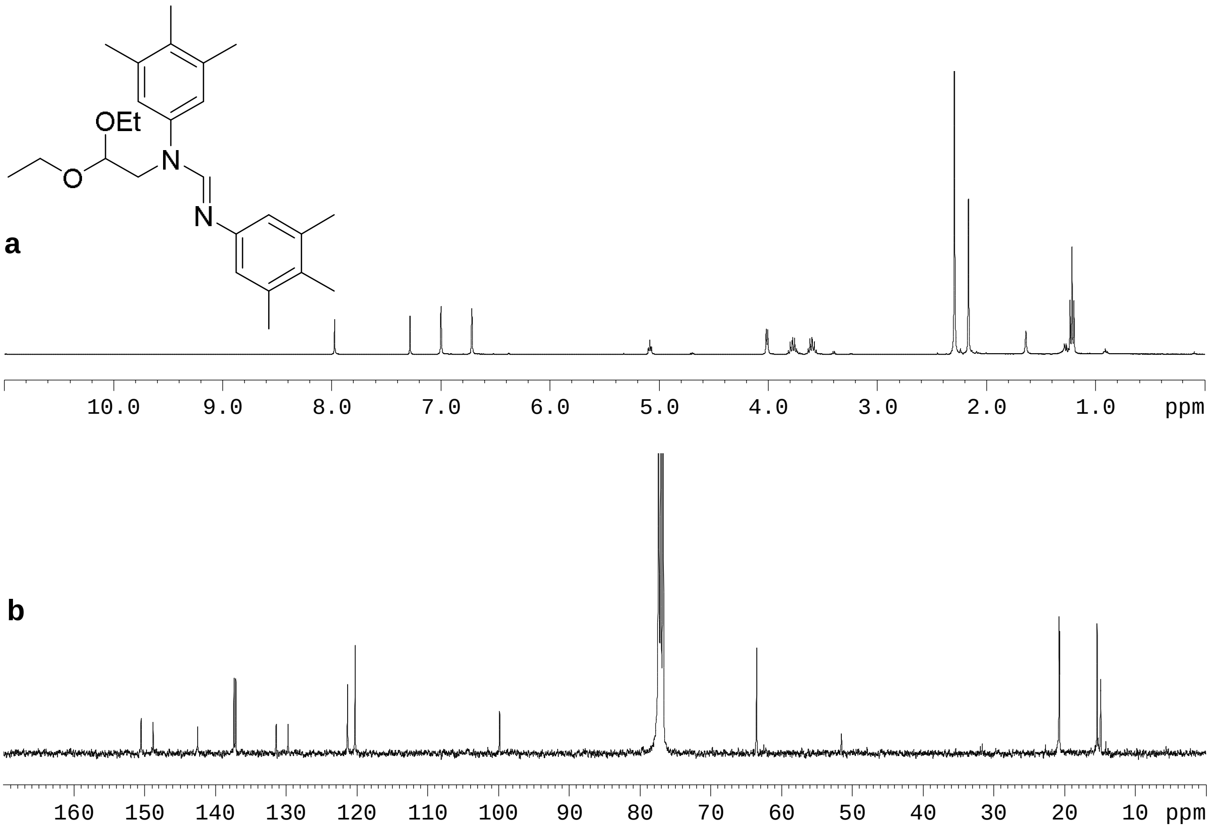


Supplementary Figure 63: NMR spectra of **S10**. **a** $^{1}$H NMR spectrum. **b** $^{13}$C NMR spectrum.

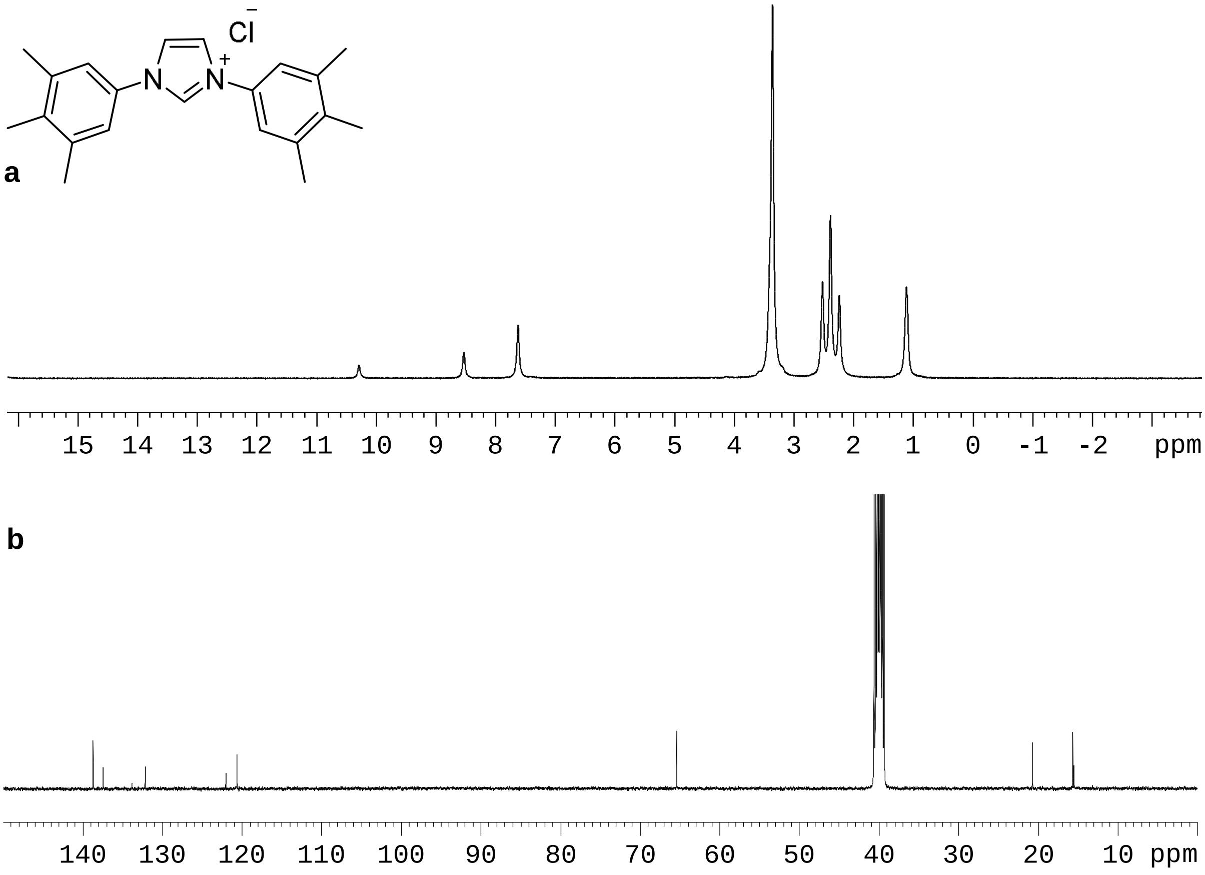


Supplementary Figure 64: NMR spectra of **S11**. **a** $^{1}$H NMR spectrum. **b** $^{13}$C NMR spectrum.

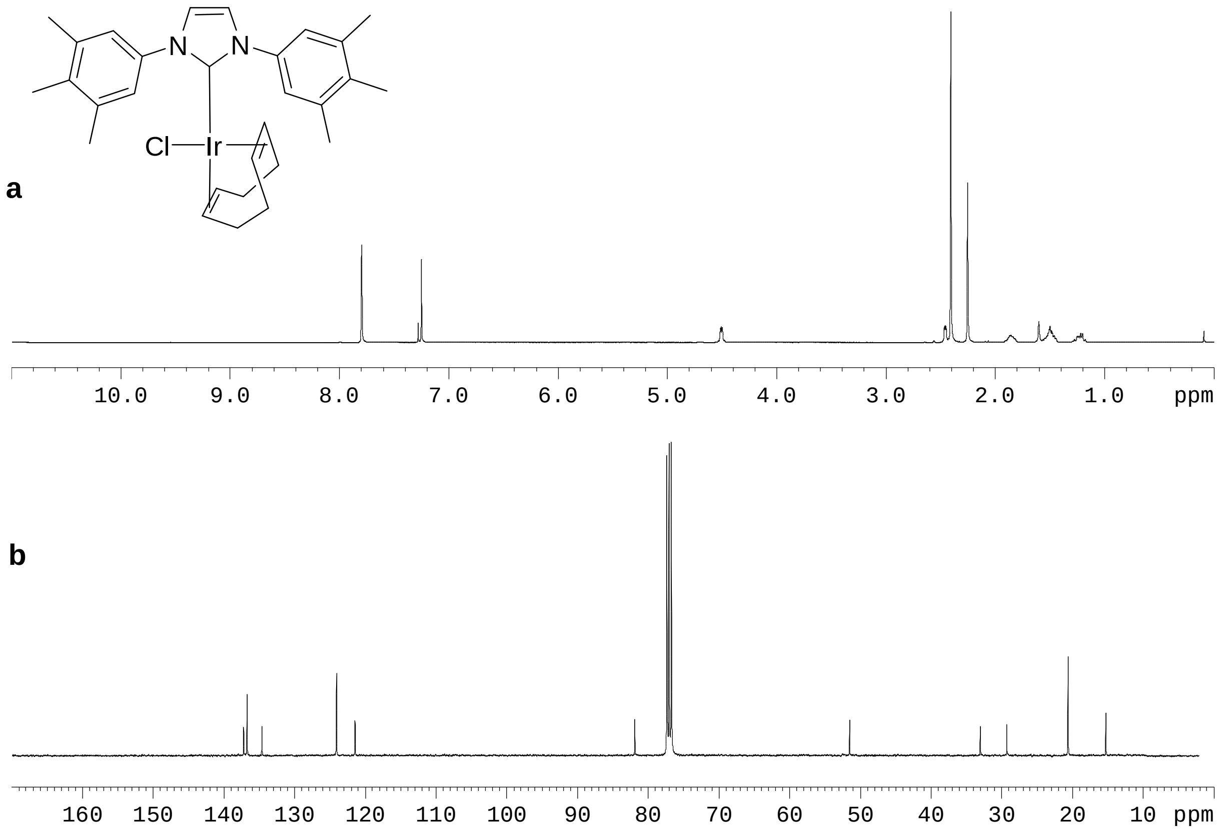


Supplementary Figure 65: NMR spectra of **7**. **a** $^{1}$H NMR spectrum. **b** $^{13}$C NMR spectrum.

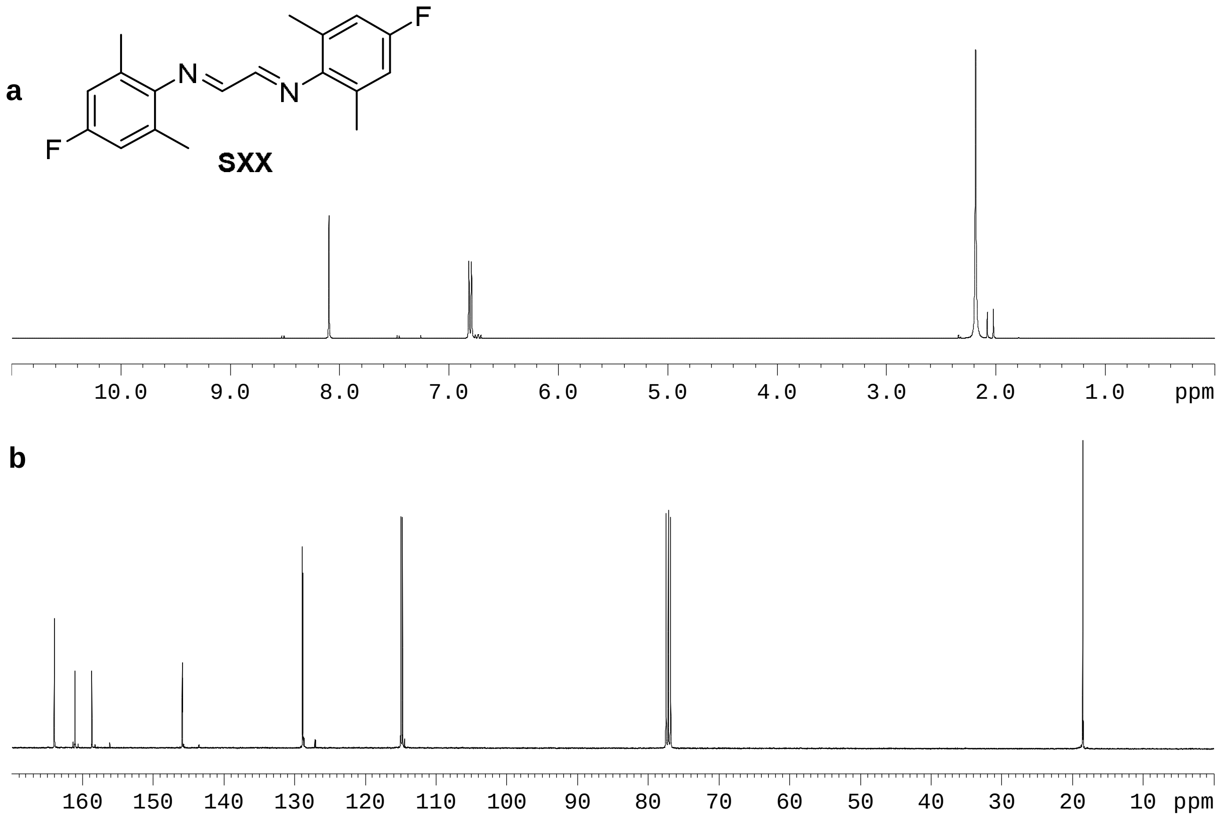


Supplementary Figure 66: NMR spectra of **S12**. **a** $^{1}$H NMR spectrum. **b** $^{13}$C NMR spectrum.

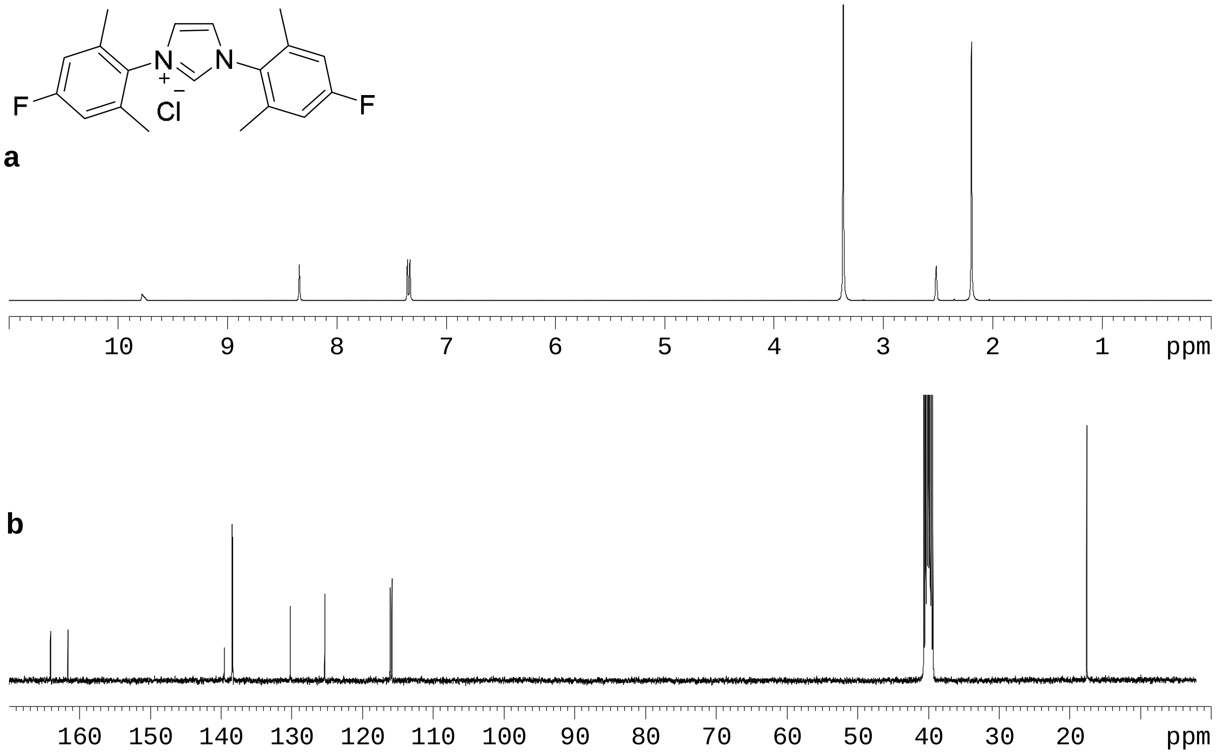


Supplementary Figure 67: NMR spectra of **S13**. **a** $^{1}$H NMR spectrum. **b** $^{13}$C NMR spectrum.

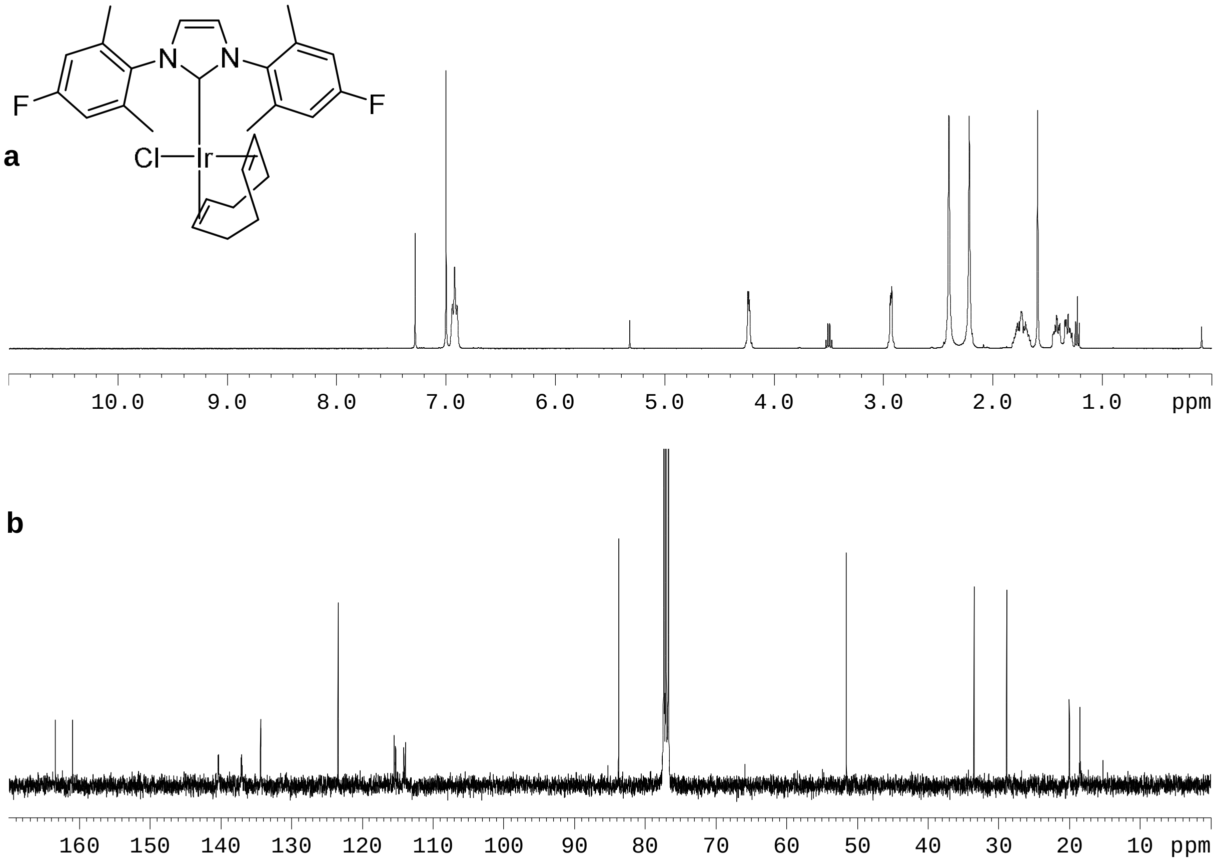


Supplementary Figure 68: NMR spectra of **8**. **a** $^{1}$H NMR spectrum. **b** $^{13}$C NMR spectrum.

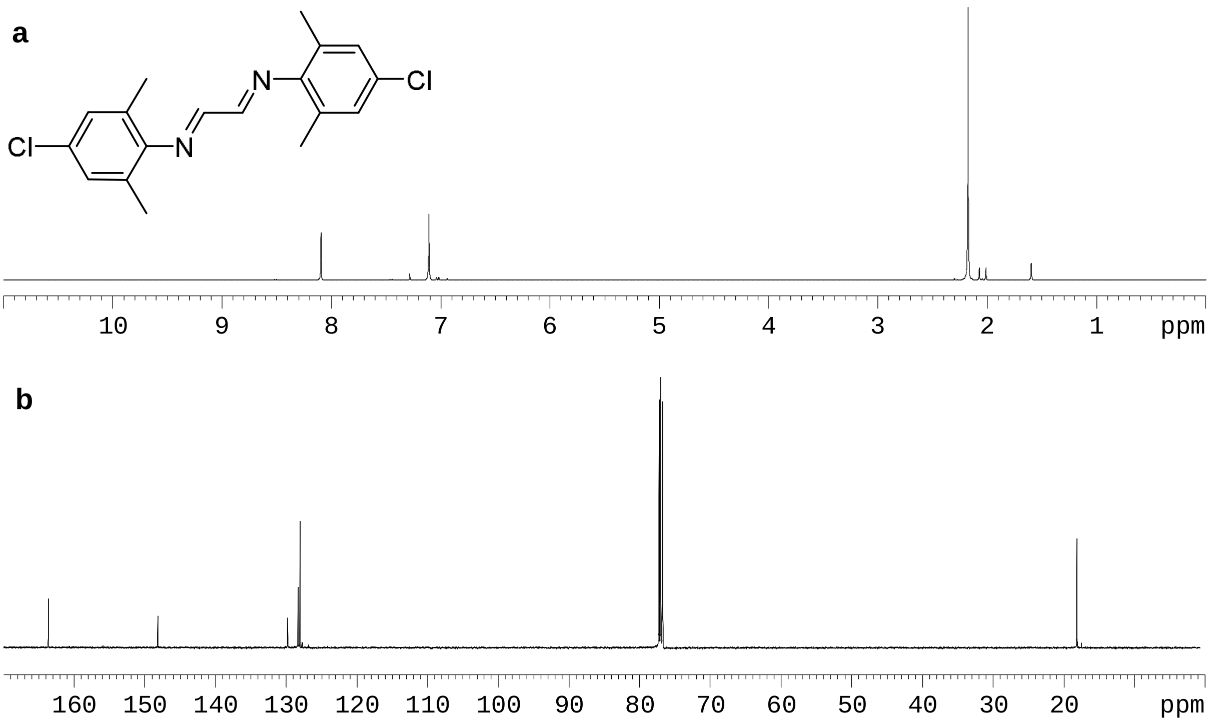


Supplementary Figure 69: NMR spectra of **S14**. **a** $^{1}$H NMR spectrum. **b** $^{13}$C NMR spectrum.

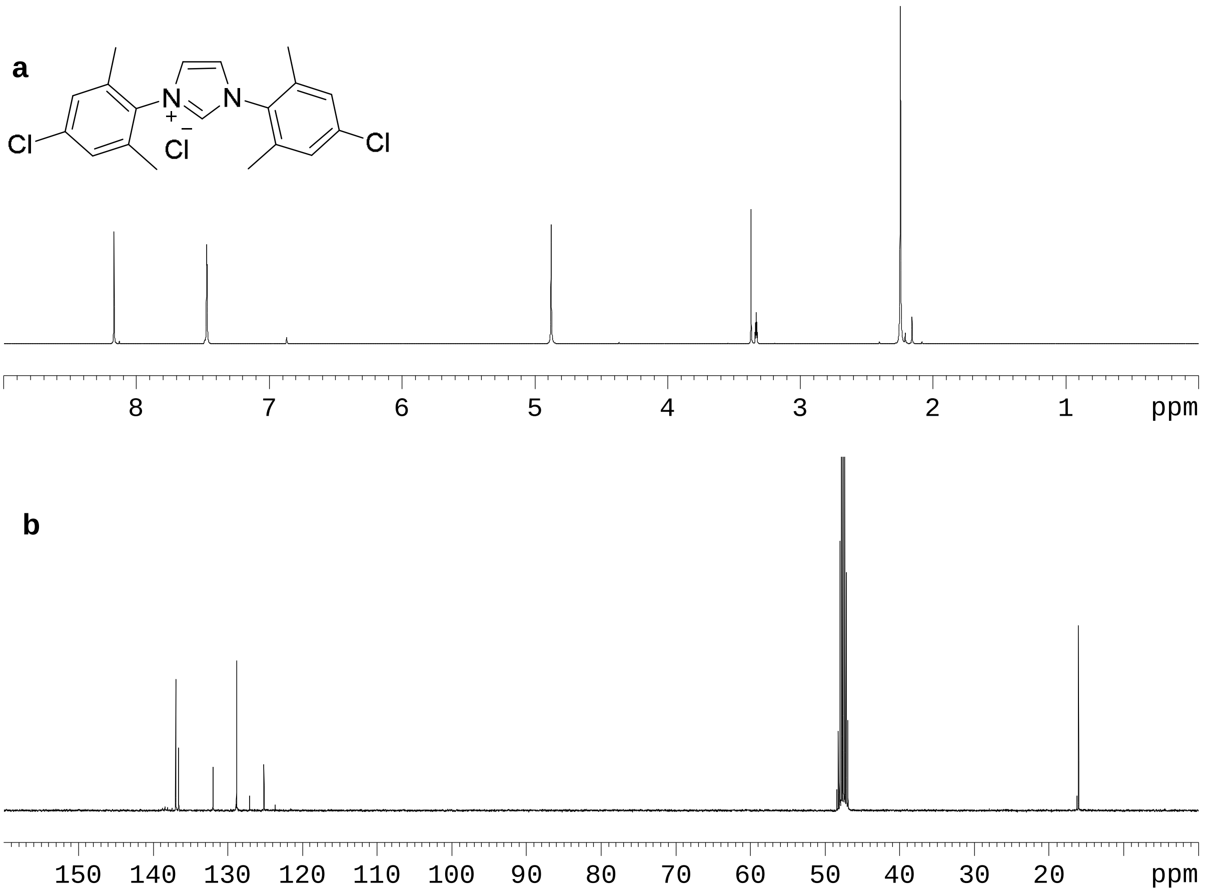


Supplementary Figure 70: NMR spectra of **S15**. **a** $^{1}$H NMR spectrum. **b** $^{13}$C NMR spectrum.

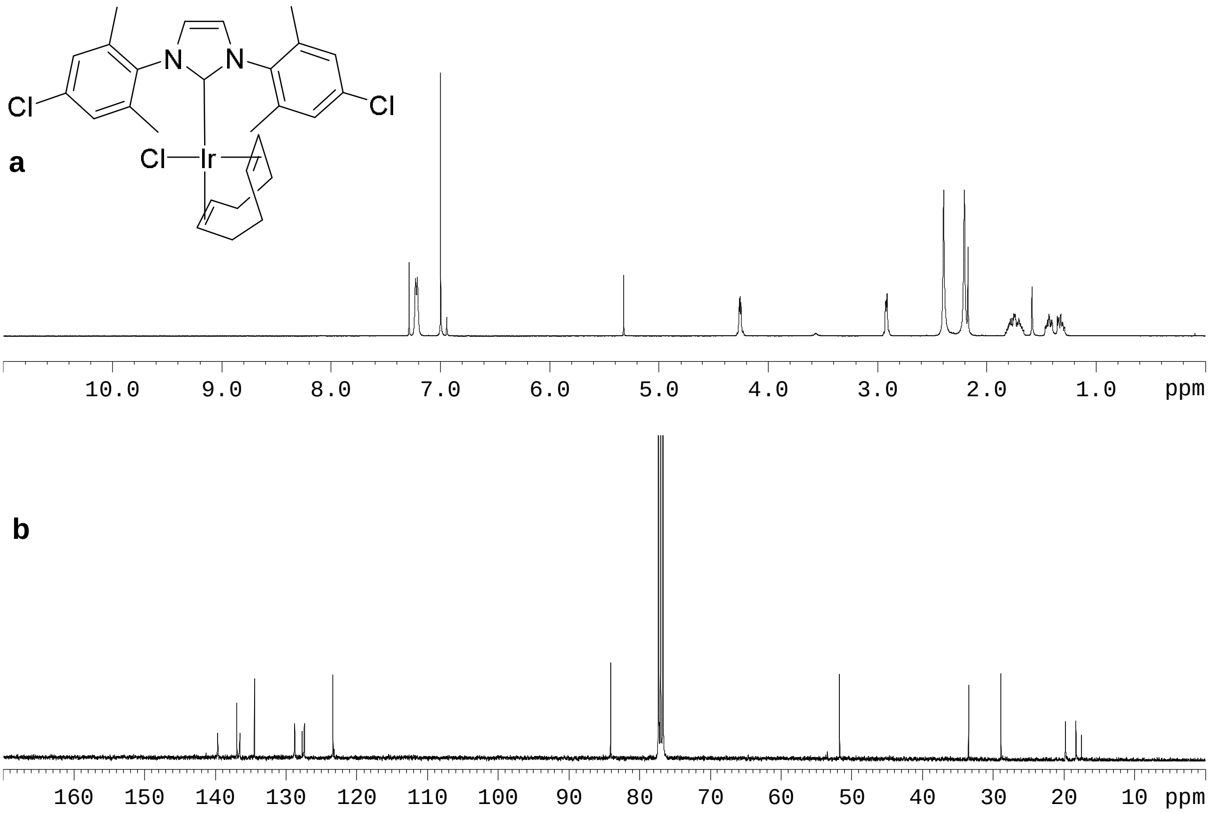


Supplementary Figure 71: NMR spectra of **9**. **a** $^{1}$H NMR spectrum. **b** $^{13}$C NMR spectrum.

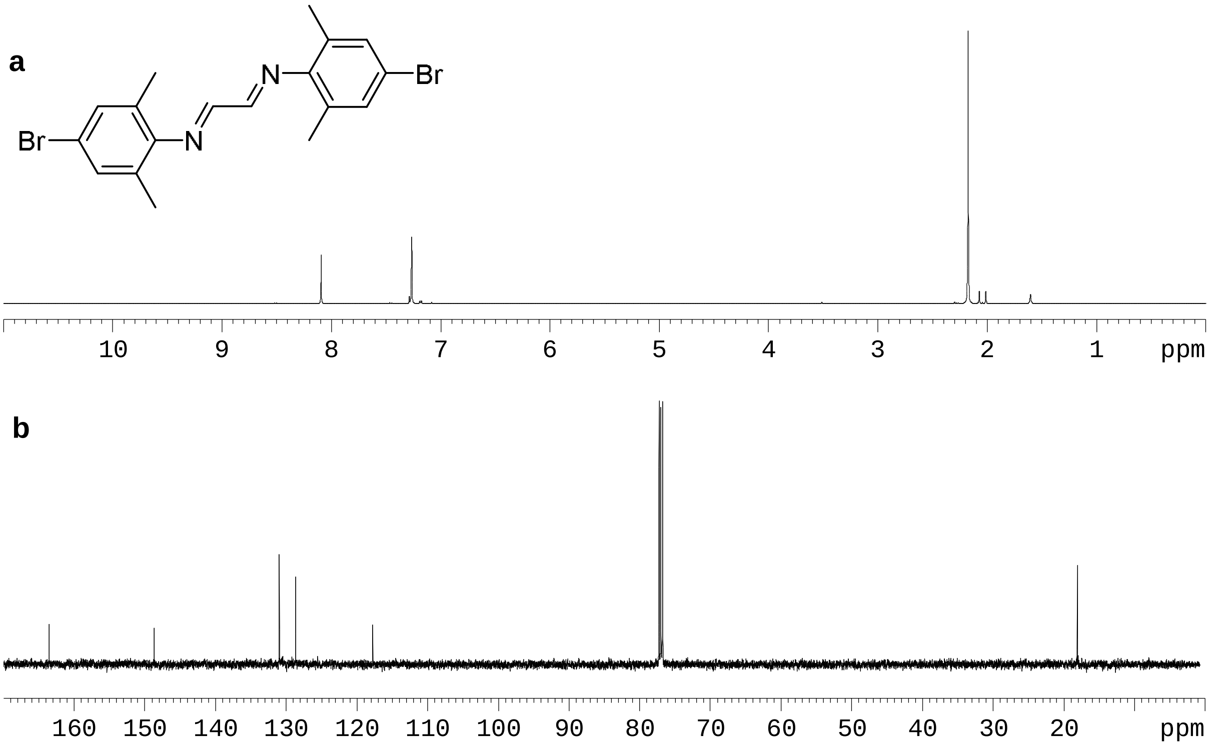


Supplementary Figure 32: NMR spectra of **S16**. **a** $^{1}$H NMR spectrum. **b** $^{13}$C NMR spectrum.

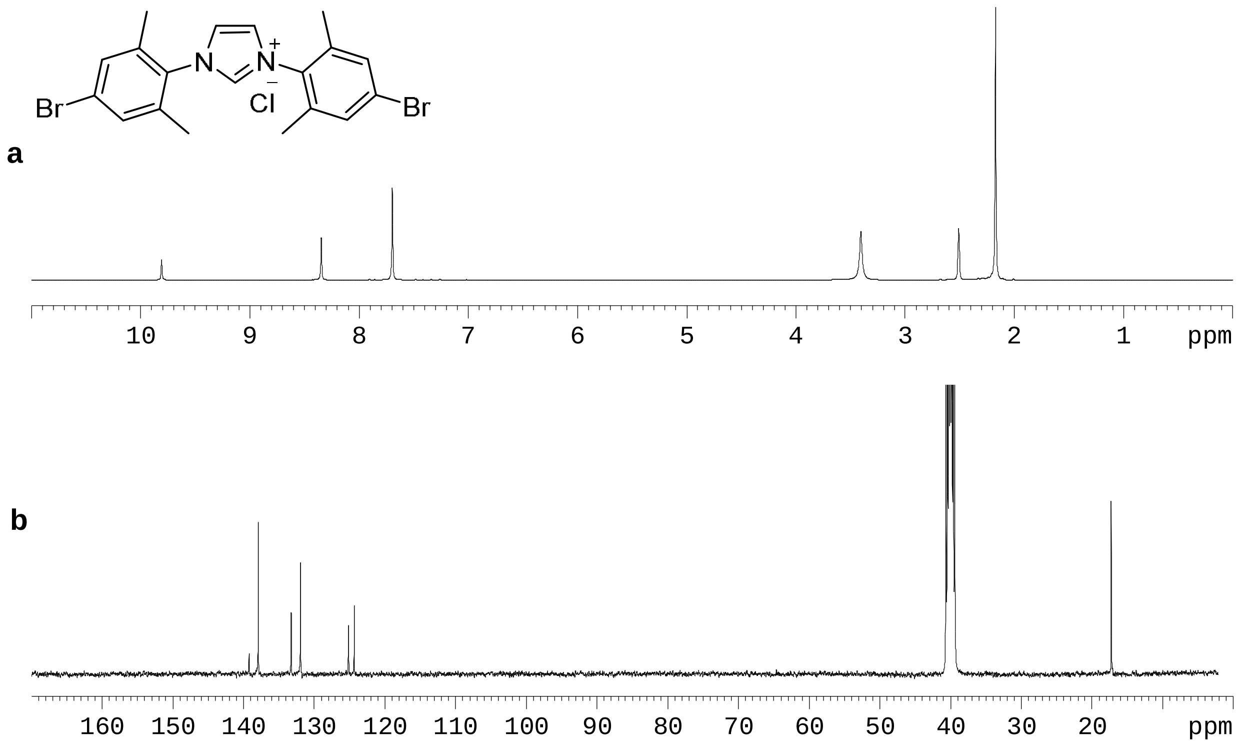


Supplementary Figure 73: NMR spectra of **S17**. **a** $^{1}H$ NMR spectrum. **b** $^{13}C$ NMR spectrum.

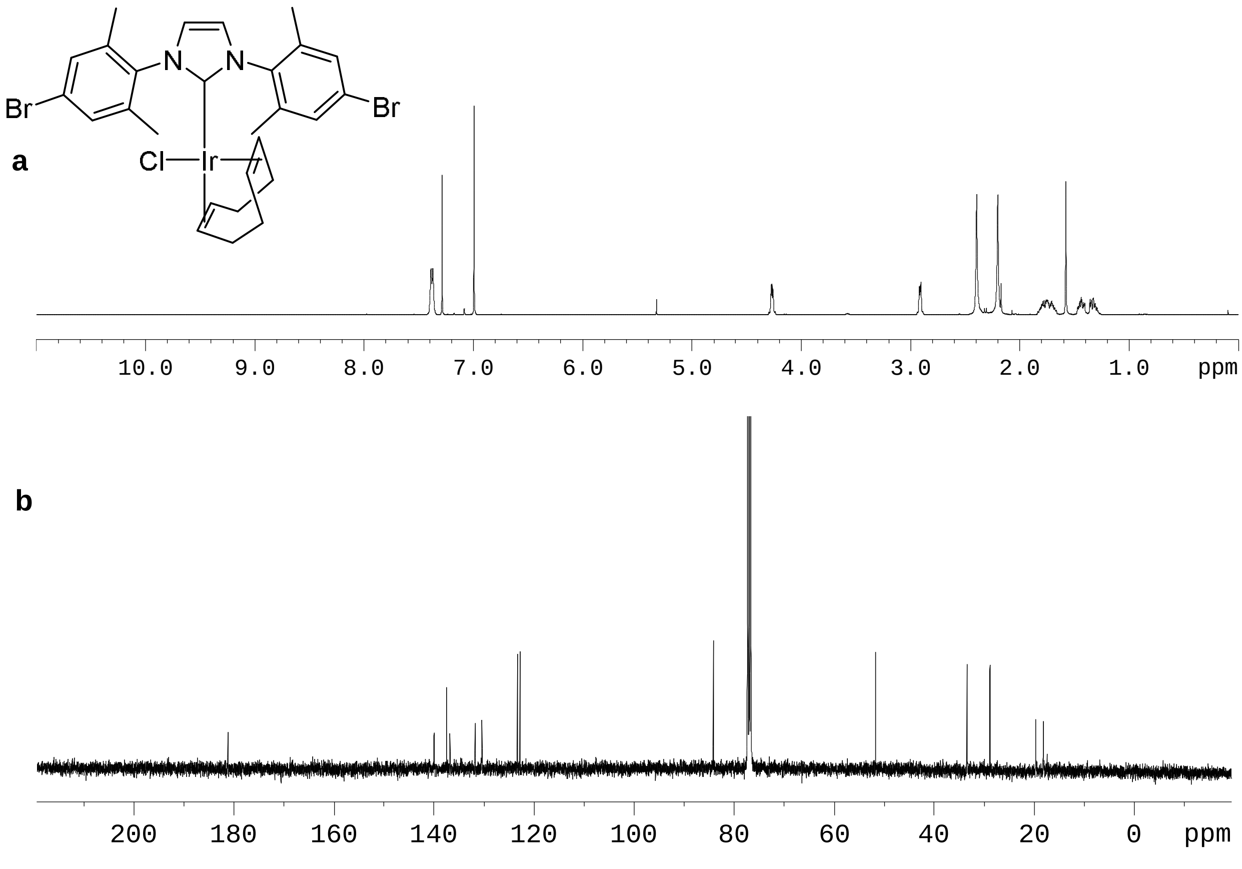


Supplementary Figure 74: NMR spectra of **10**. **a** $^{1}H$ NMR spectrum. **b** $^{13}C$ NMR spectrum.

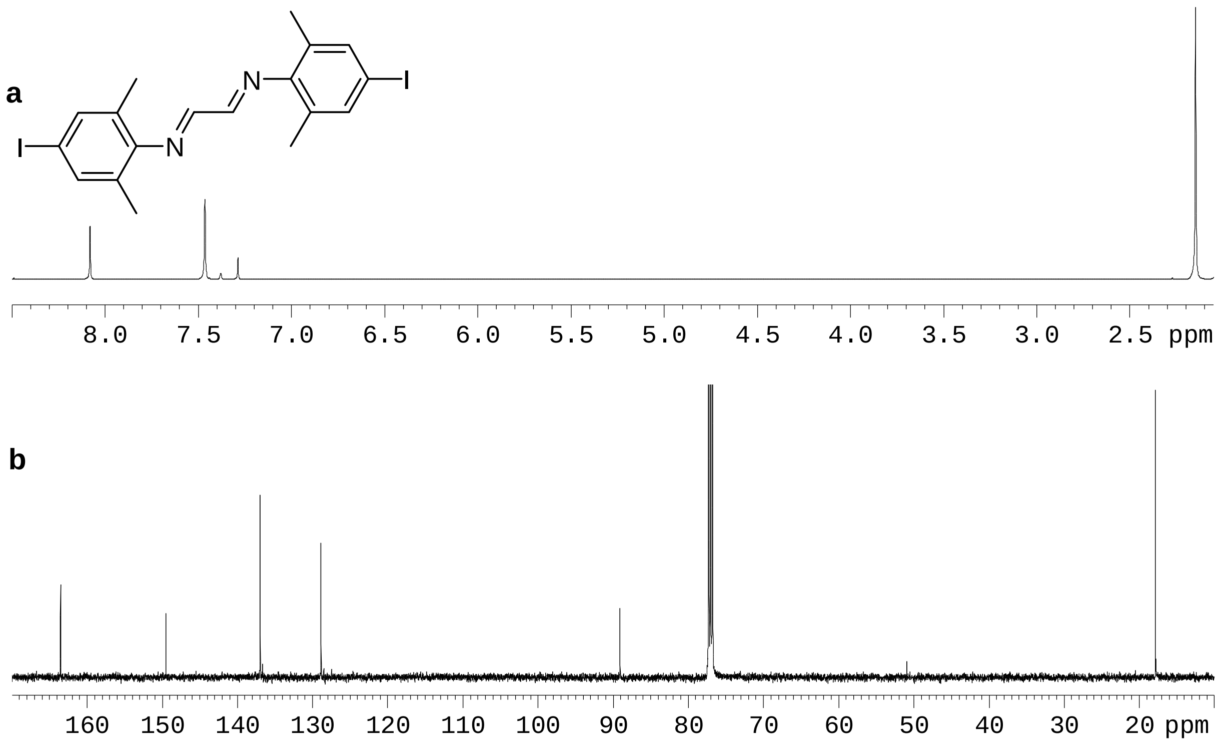


Supplementary Figure 75: NMR spectra of **S18**. **a** $^{1}$H NMR spectrum. **b** $^{13}$C NMR spectrum.

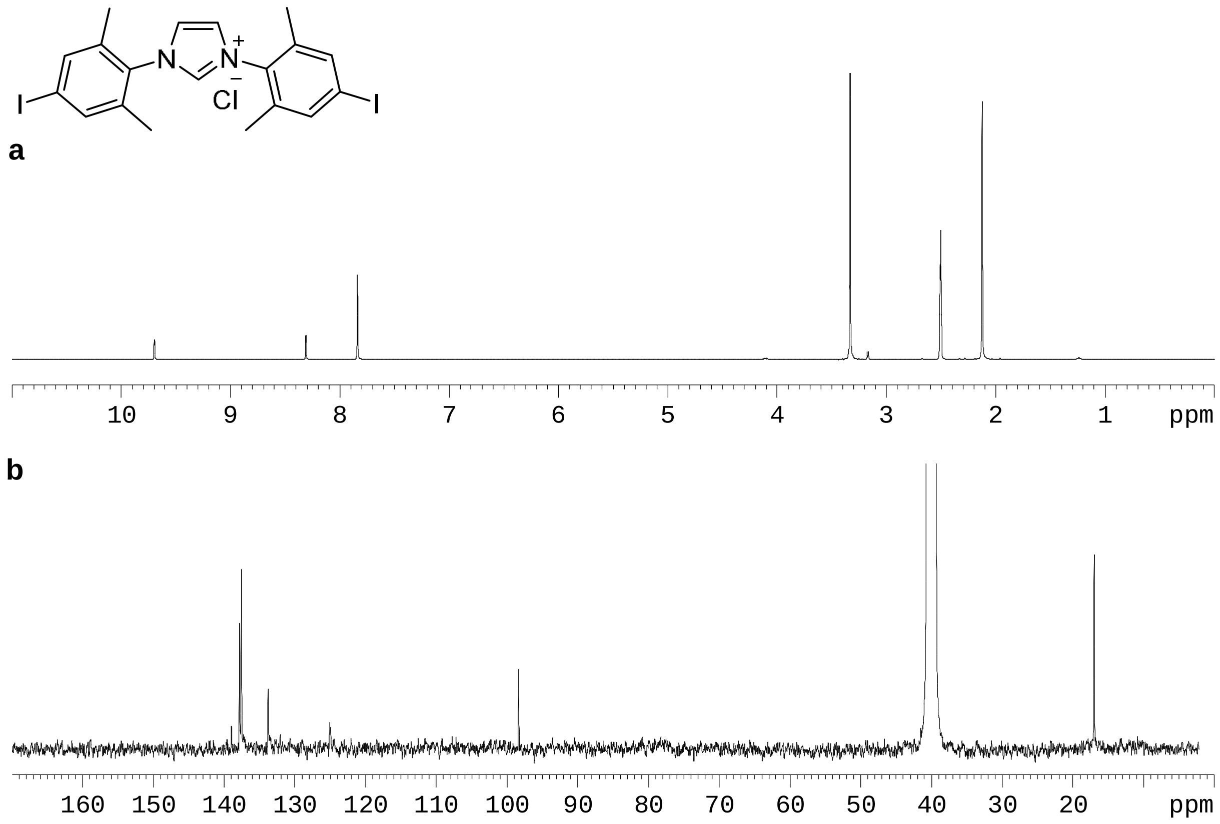


Supplementary Figure 76: NMR spectra of **S19**. **a** $^{1}$H NMR spectrum. **b** $^{13}$C NMR spectrum.

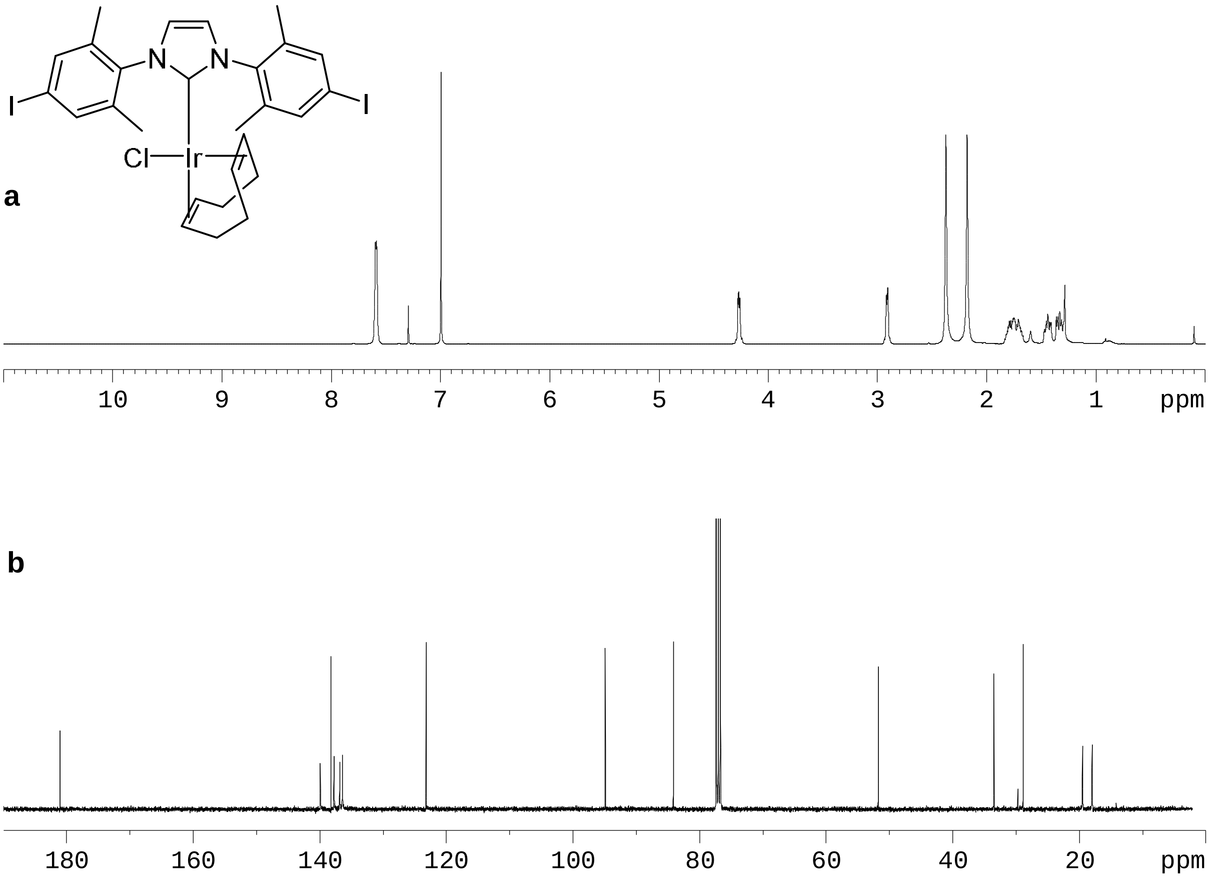


Supplementary Figure 77: NMR spectra of **11**. **a** $^{1}$H NMR spectrum. **b** $^{13}$C NMR spectrum.

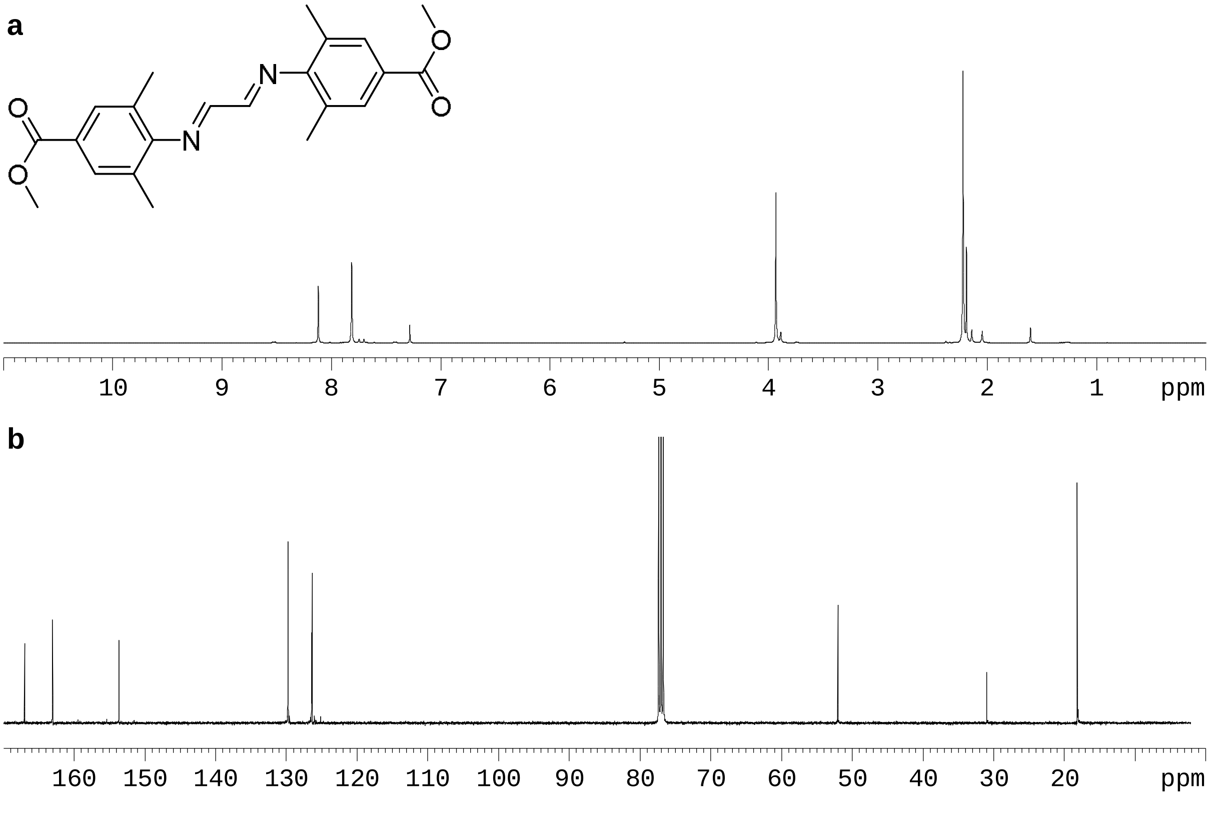


Supplementary Figure 78: NMR spectra of **S20**. **a** $^{1}$H NMR spectrum. **b** $^{13}$C NMR spectrum.

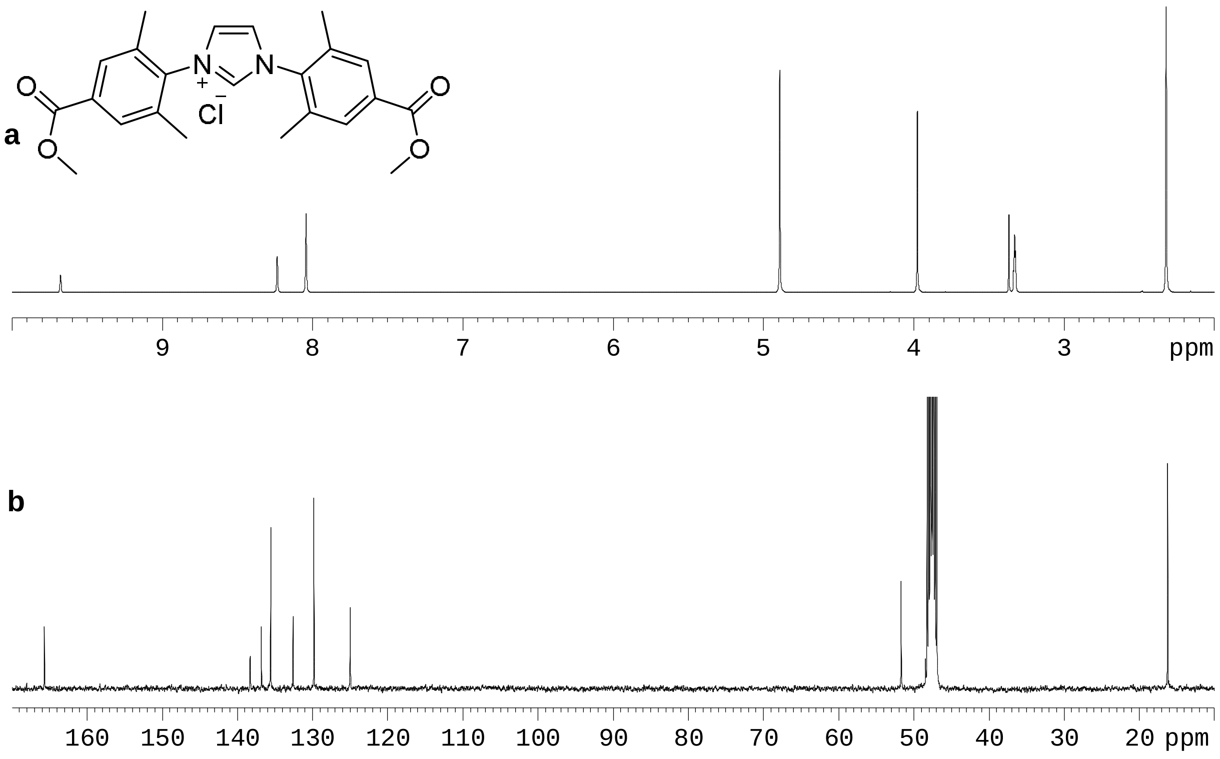


Supplementary Figure 79: NMR spectra of **S21**. **a** $^{1}$H NMR spectrum. **b** $^{13}$C NMR spectrum.

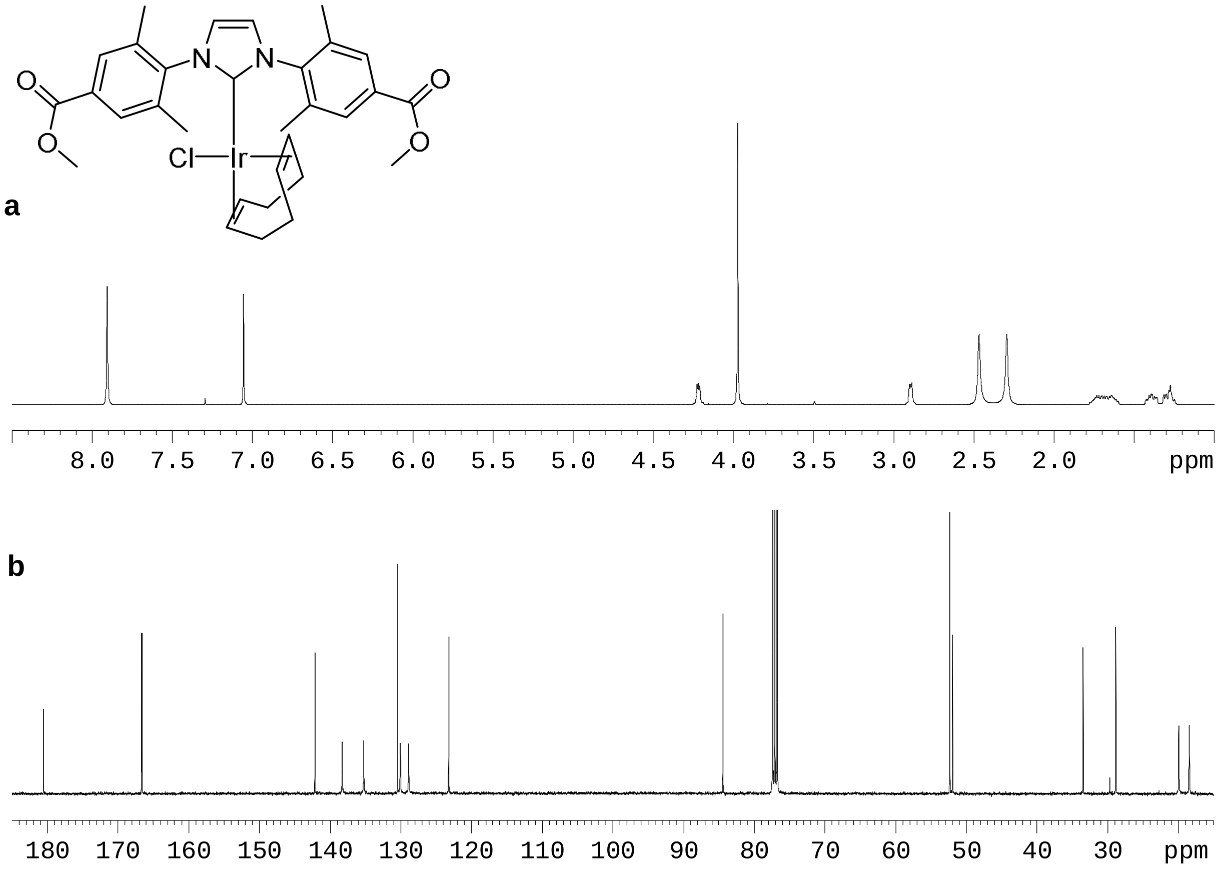


Supplementary Figure 80: NMR spectra of **12**. **a** $^{1}$H NMR spectrum. **b** $^{13}$C NMR spectrum.

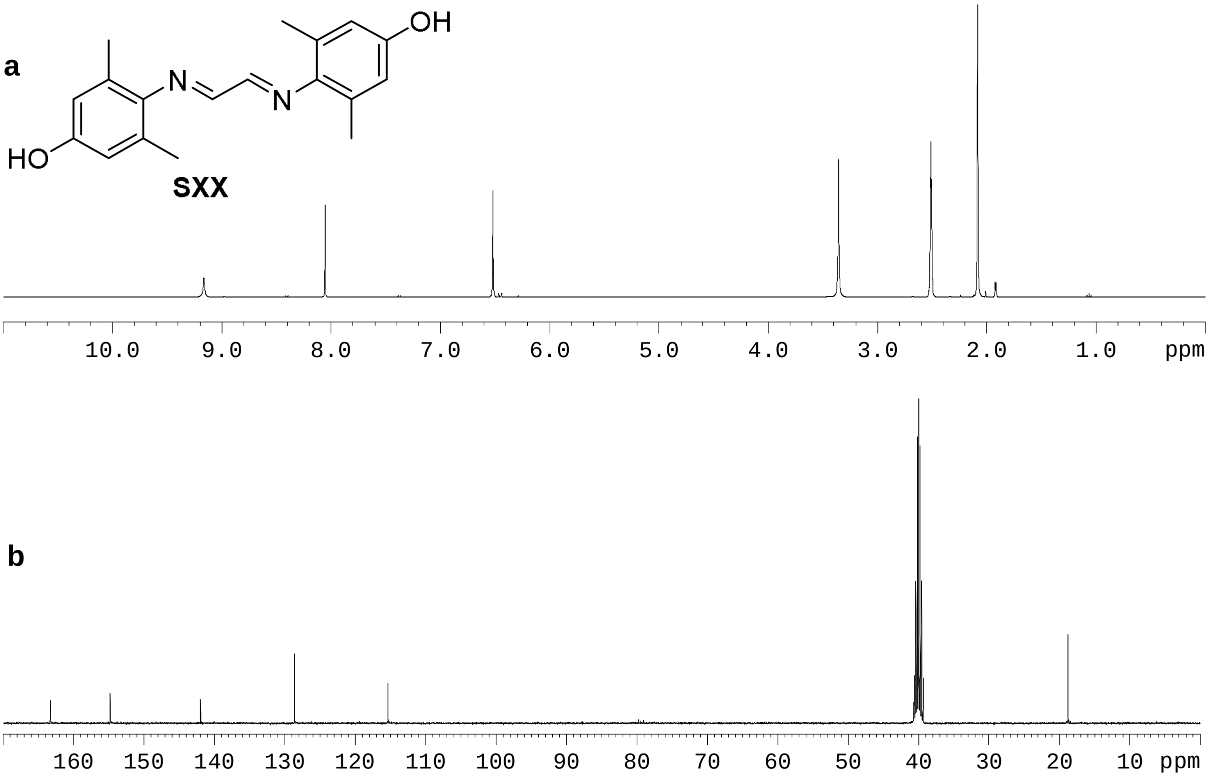


Supplementary Figure 81: NMR spectra of **S22**. **a** $^{1}$H NMR spectrum. **b** $^{13}$C NMR spectrum.

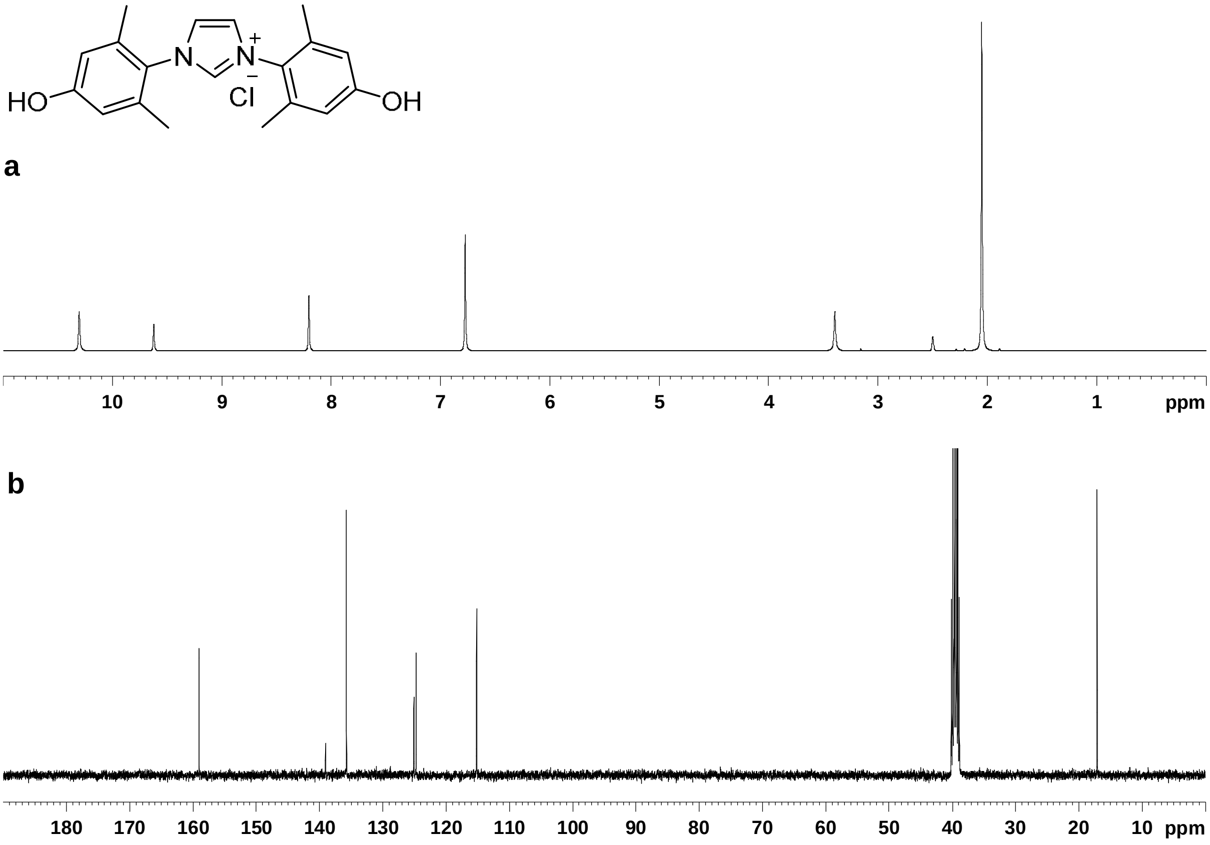


Supplementary Figure 82: NMR spectra of **S23**. **a** $^{1}$H NMR spectrum. **b** $^{13}$C NMR spectrum.

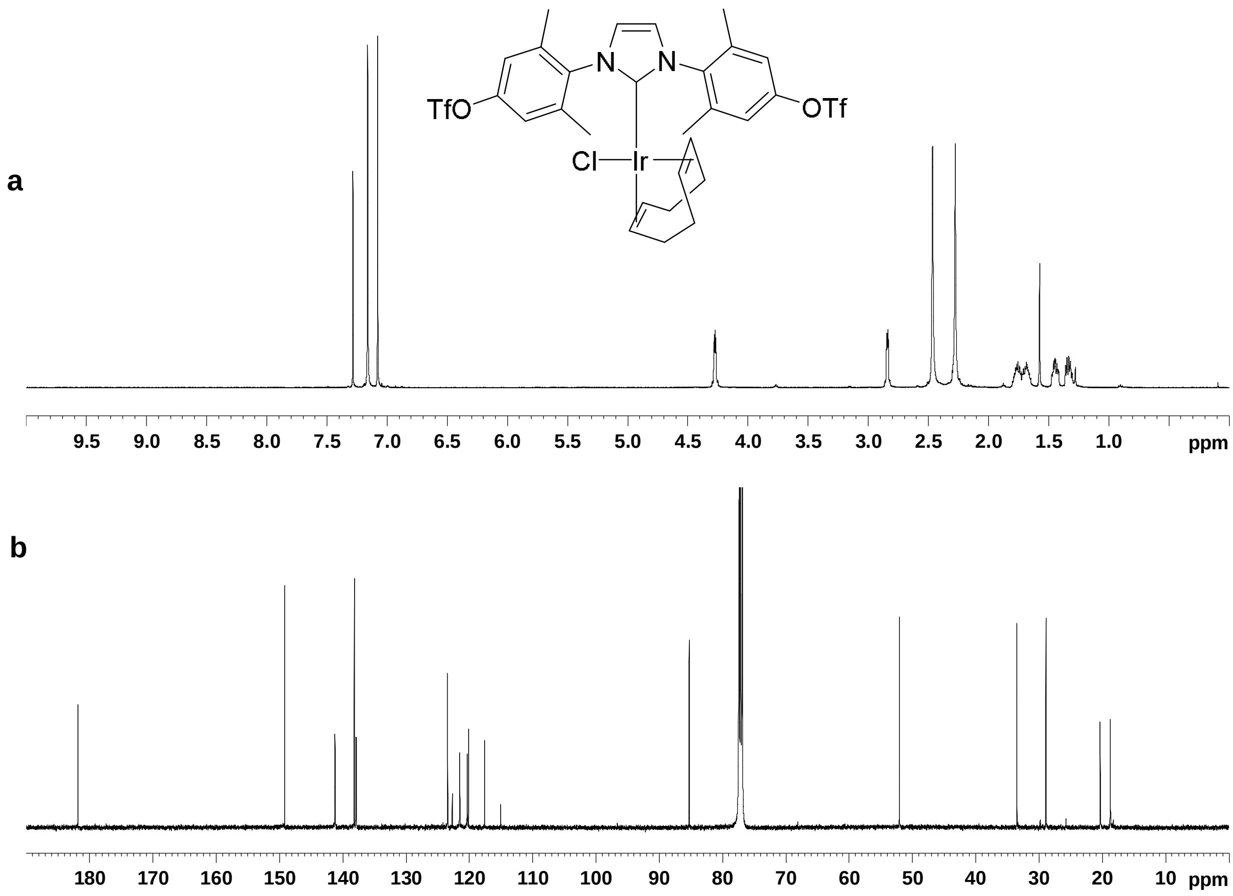


Supplementary Figure 83: NMR spectra of **13**. **a** $^{1}$H NMR spectrum. **b** $^{13}$C NMR spectrum.

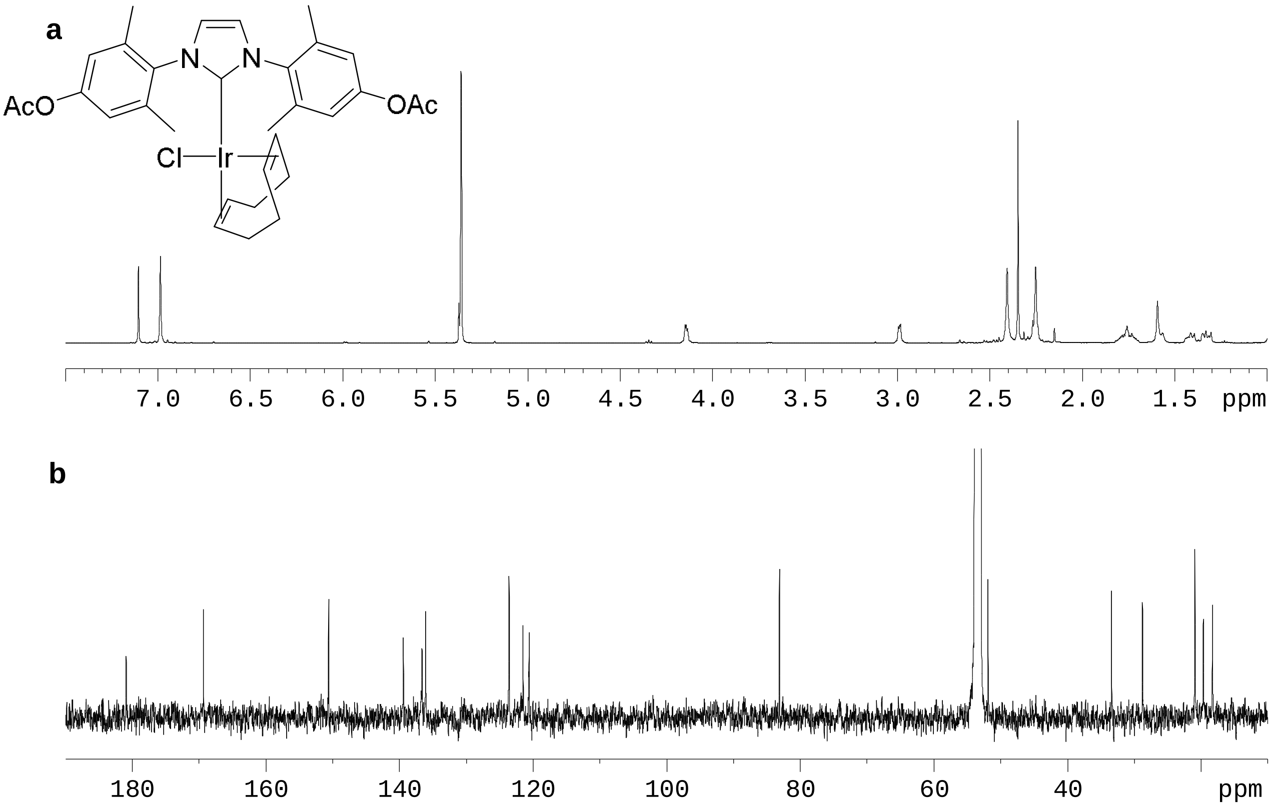


Supplementary Figure 84: NMR spectra of **14**. **a** $^{1}$H NMR spectrum. **b** $^{13}$C NMR spectrum.

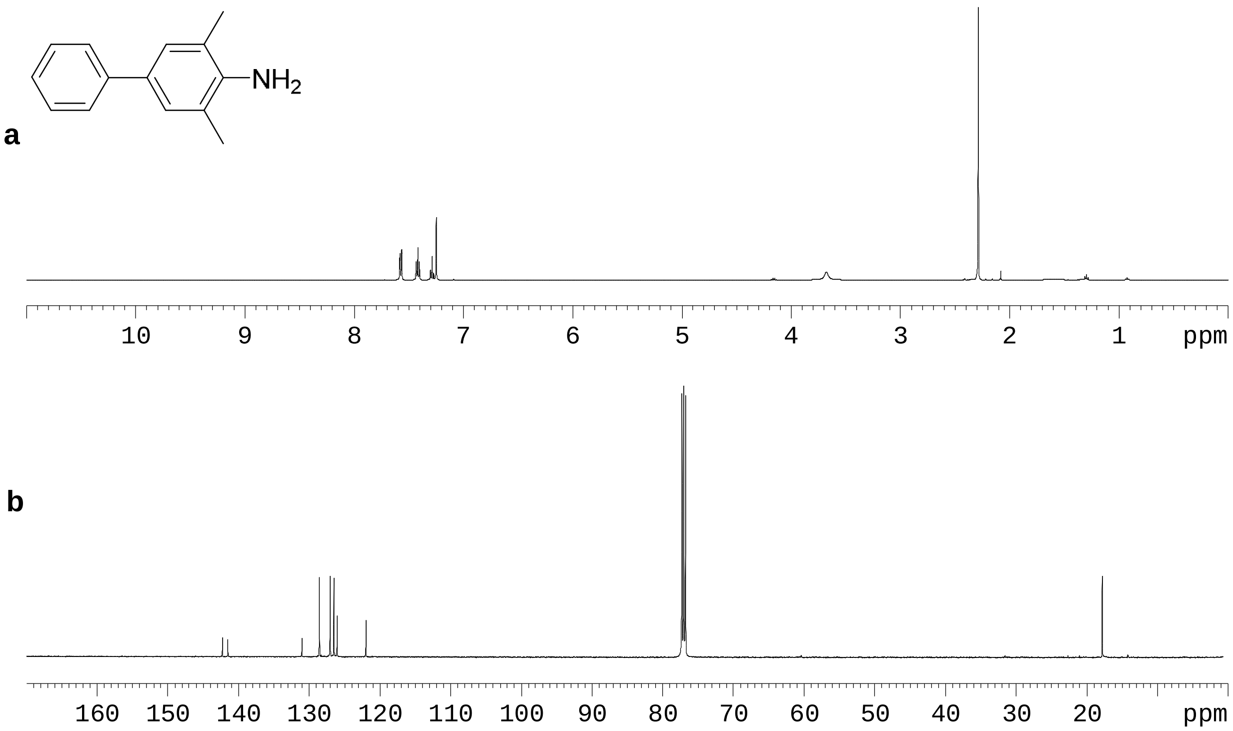


Supplementary Figure 85: NMR spectra of **S26**. **a** $^{1}$H NMR spectrum. **b** $^{13}$C NMR spectrum.

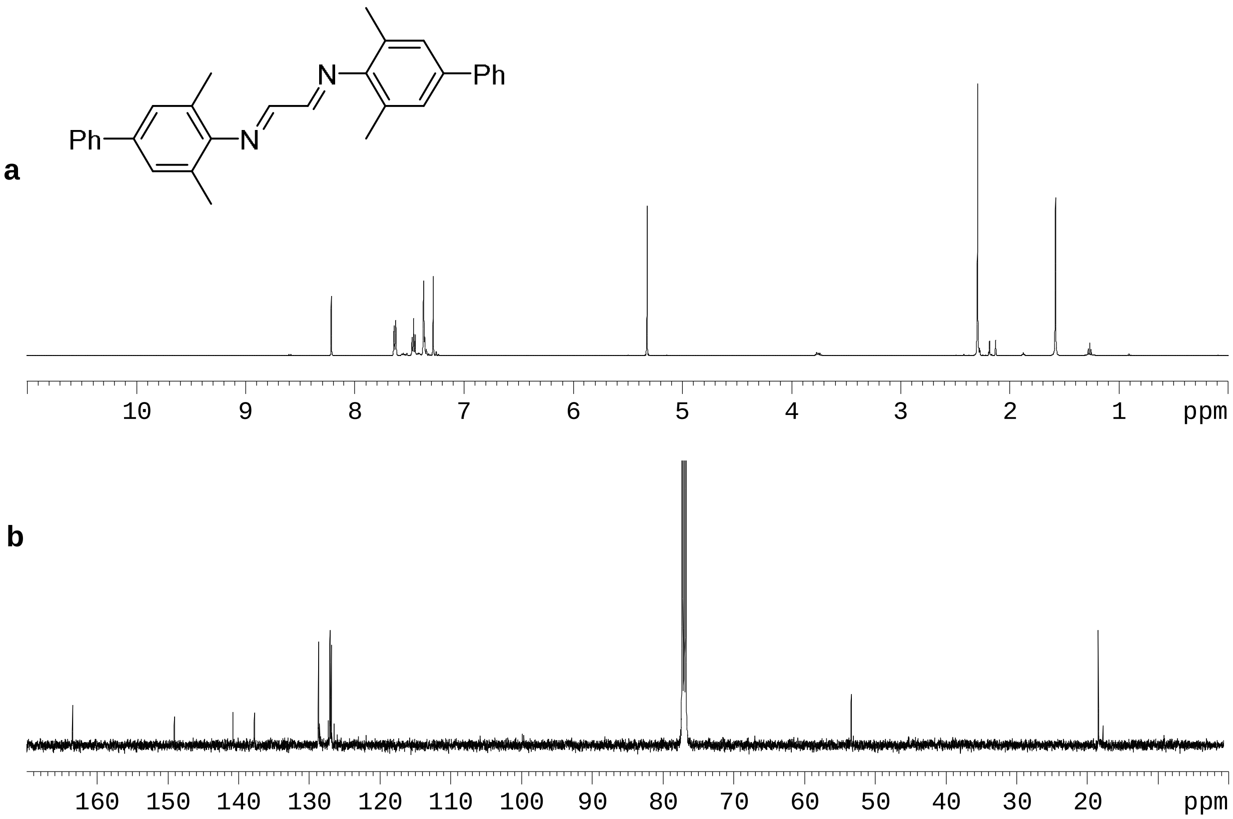


Supplementary Figure 86: NMR spectra of **S27**. **a** $^{1}$H NMR spectrum. **b** $^{13}$C NMR spectrum.

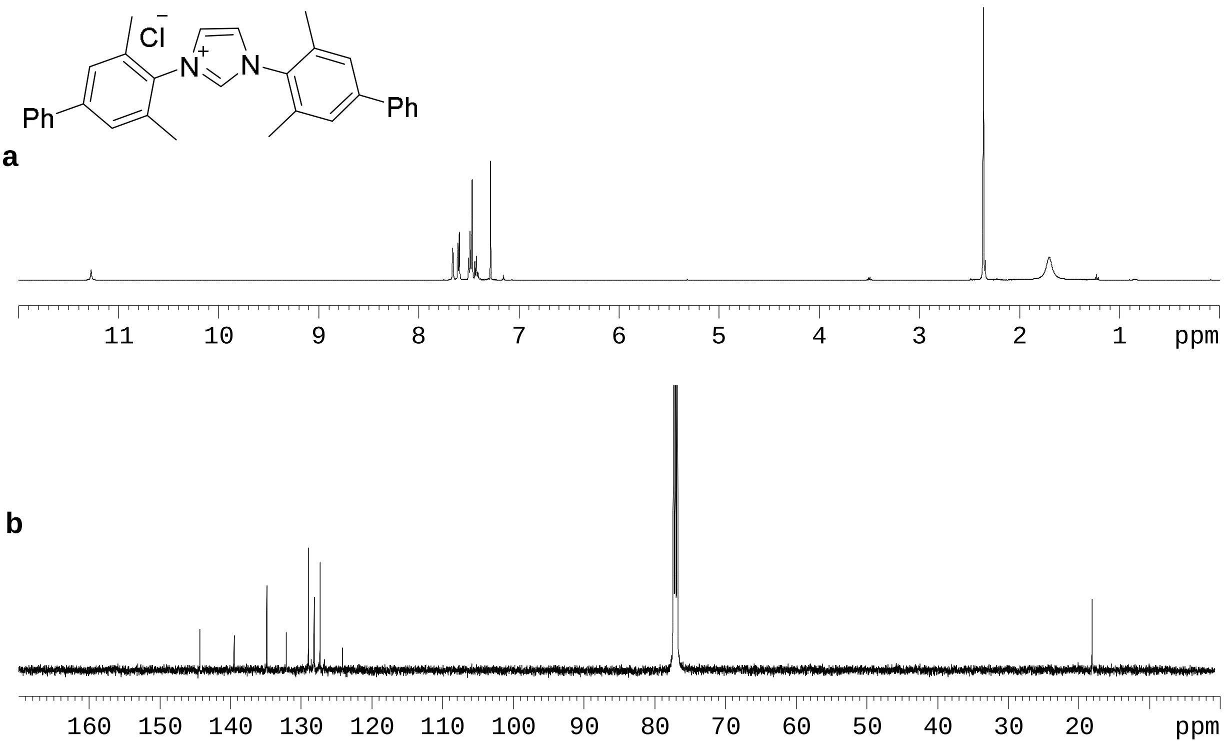


Supplementary Figure 87: NMR spectra of **S28**. **a** $^{1}$H NMR spectrum. **b** $^{13}$C NMR spectrum.

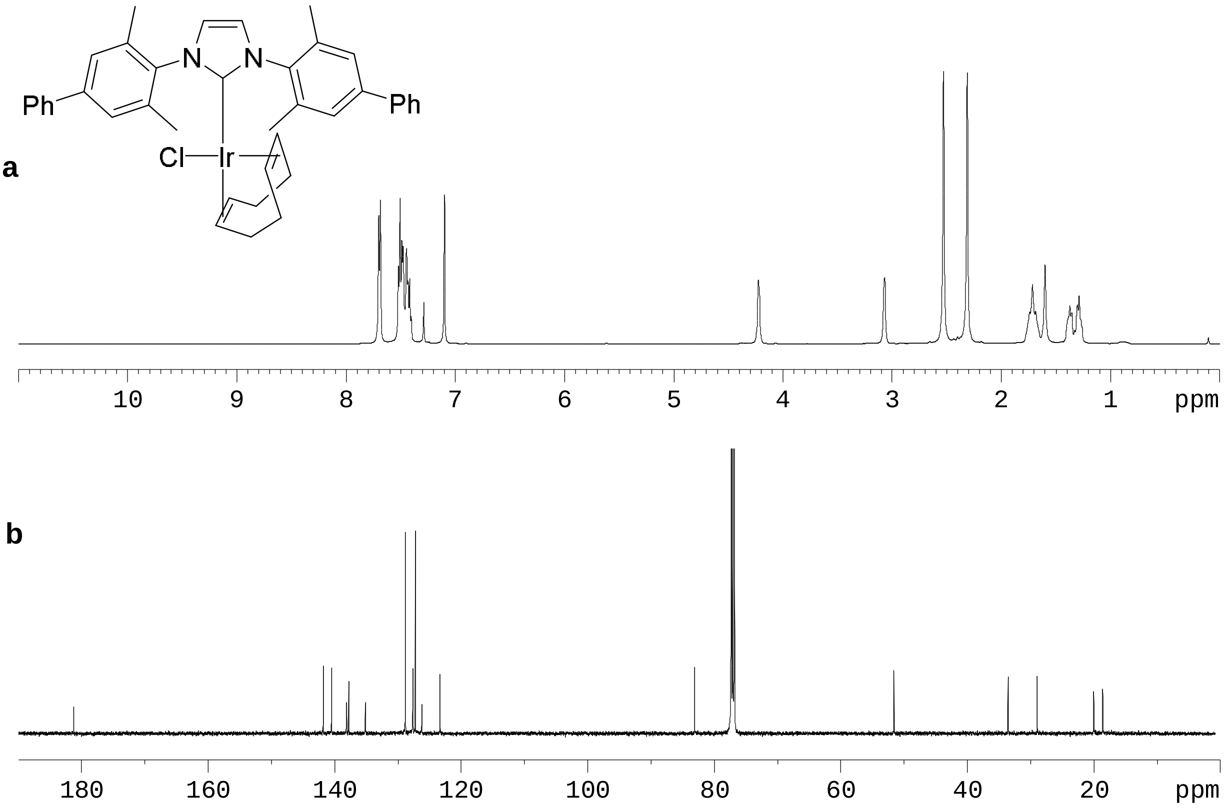


Supplementary Figure 88: NMR spectra of **15**. **a** $^{1}$H NMR spectrum. **b** $^{13}$C NMR spectrum.

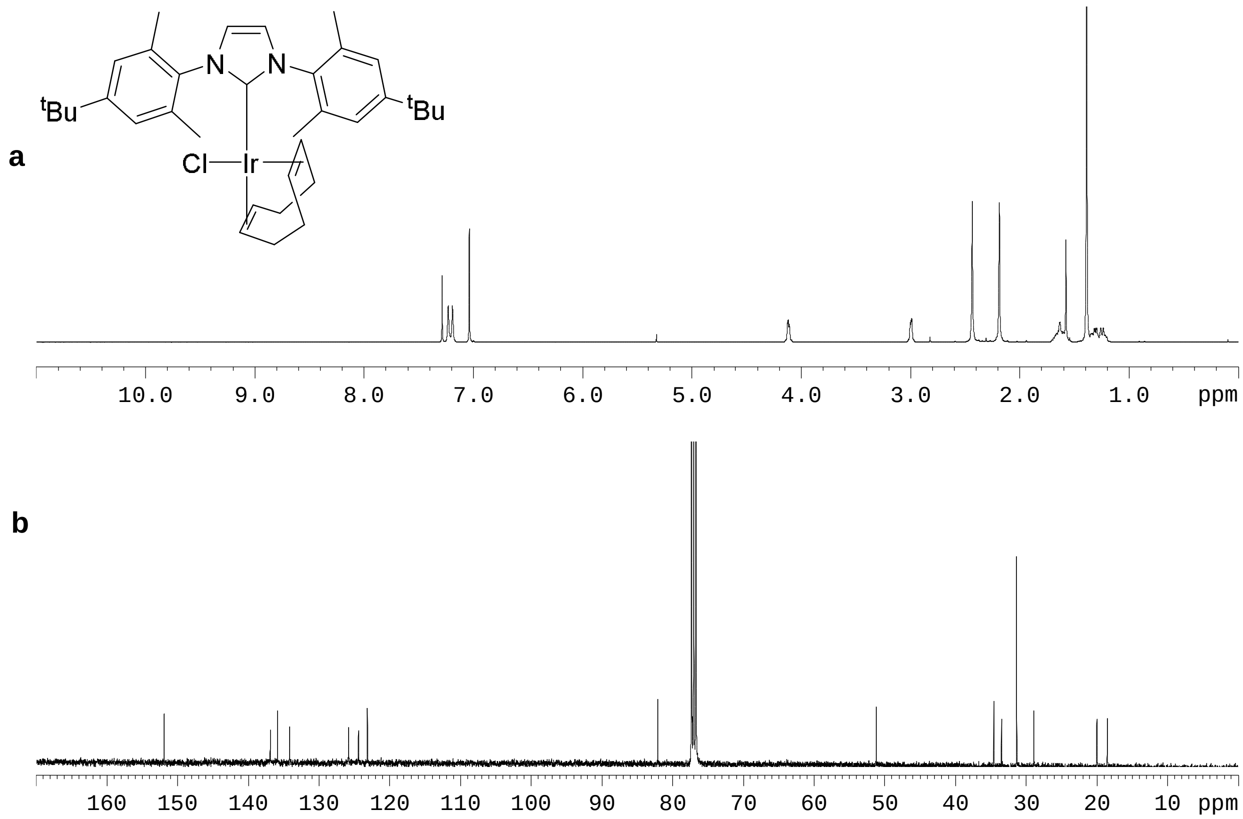


Supplementary Figure 89: NMR spectra of **16**. **a** $^{1}$H NMR spectrum. **b** $^{13}$C NMR spectrum.

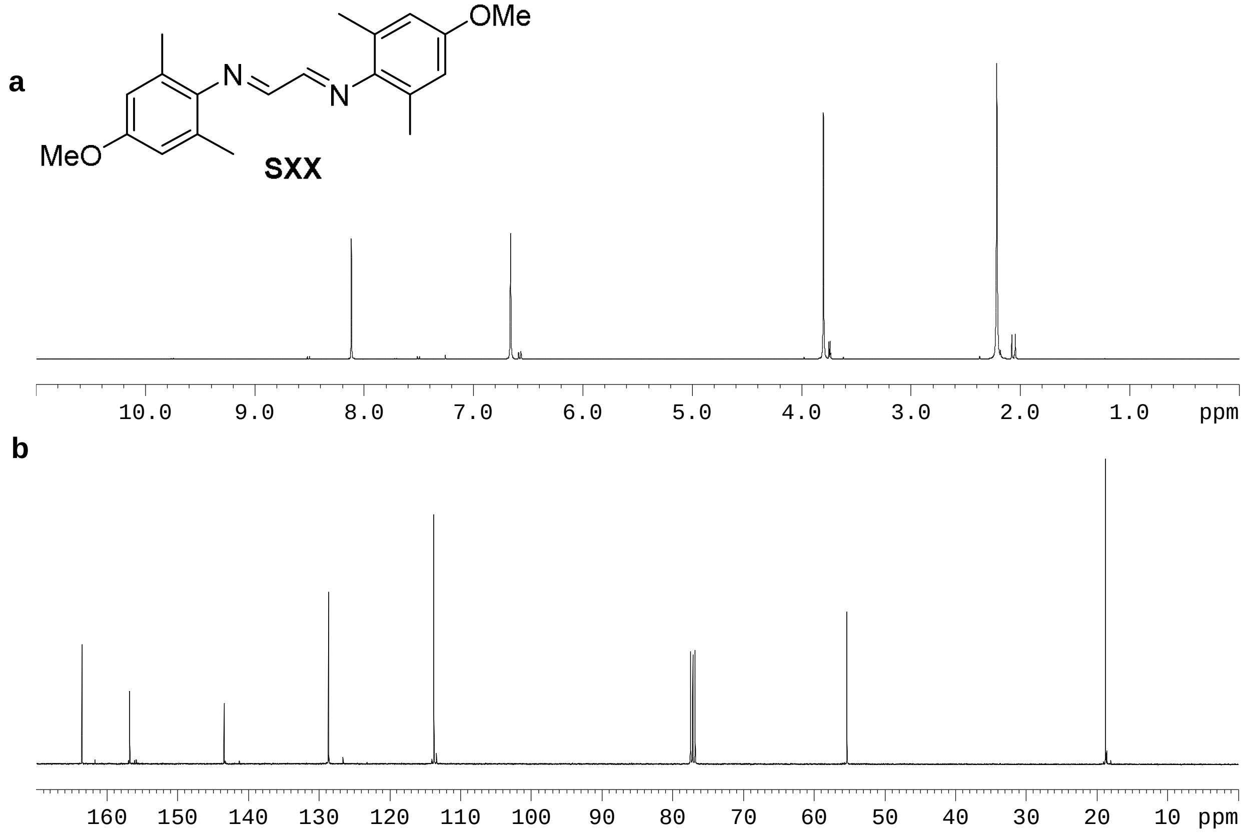


Supplementary Figure 90: NMR spectra of **S31**. **a** $^{1}$H NMR spectrum. **b** $^{13}$C NMR spectrum.

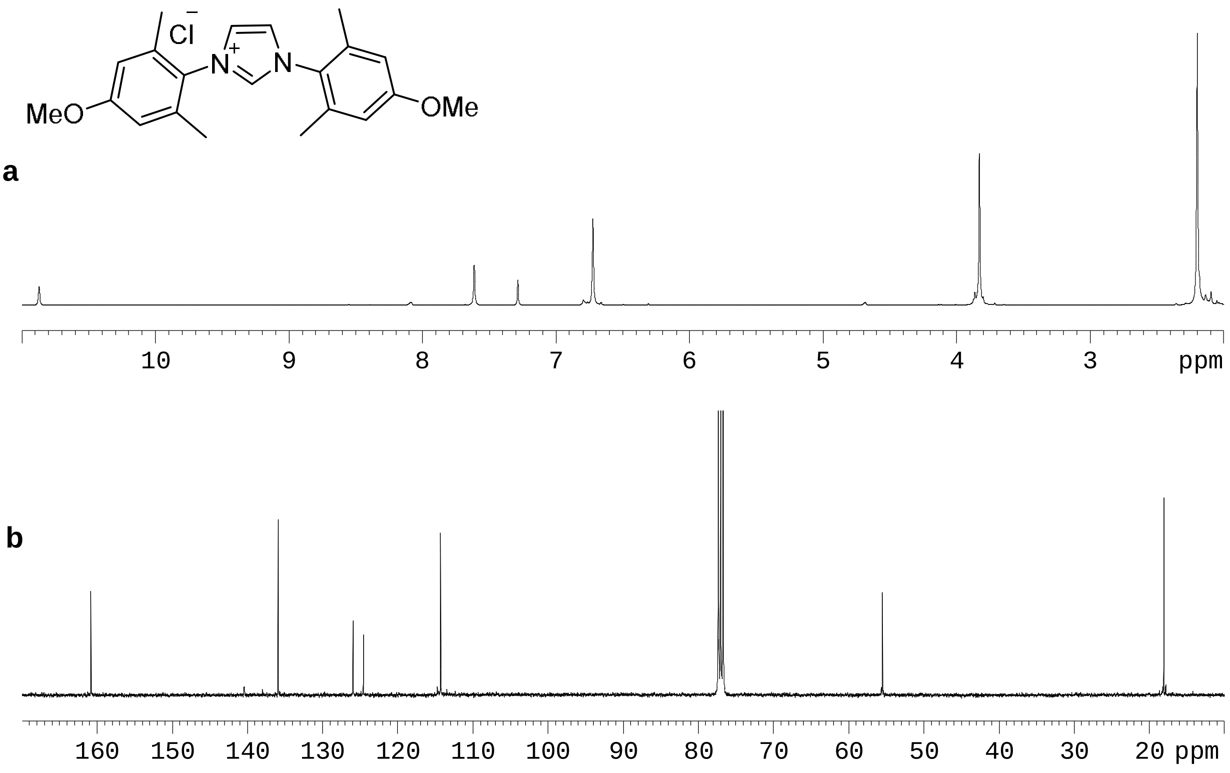


Supplementary Figure 91: NMR spectra of **S32**. **a** $^{1}$H NMR spectrum. **b** $^{13}$C NMR spectrum.

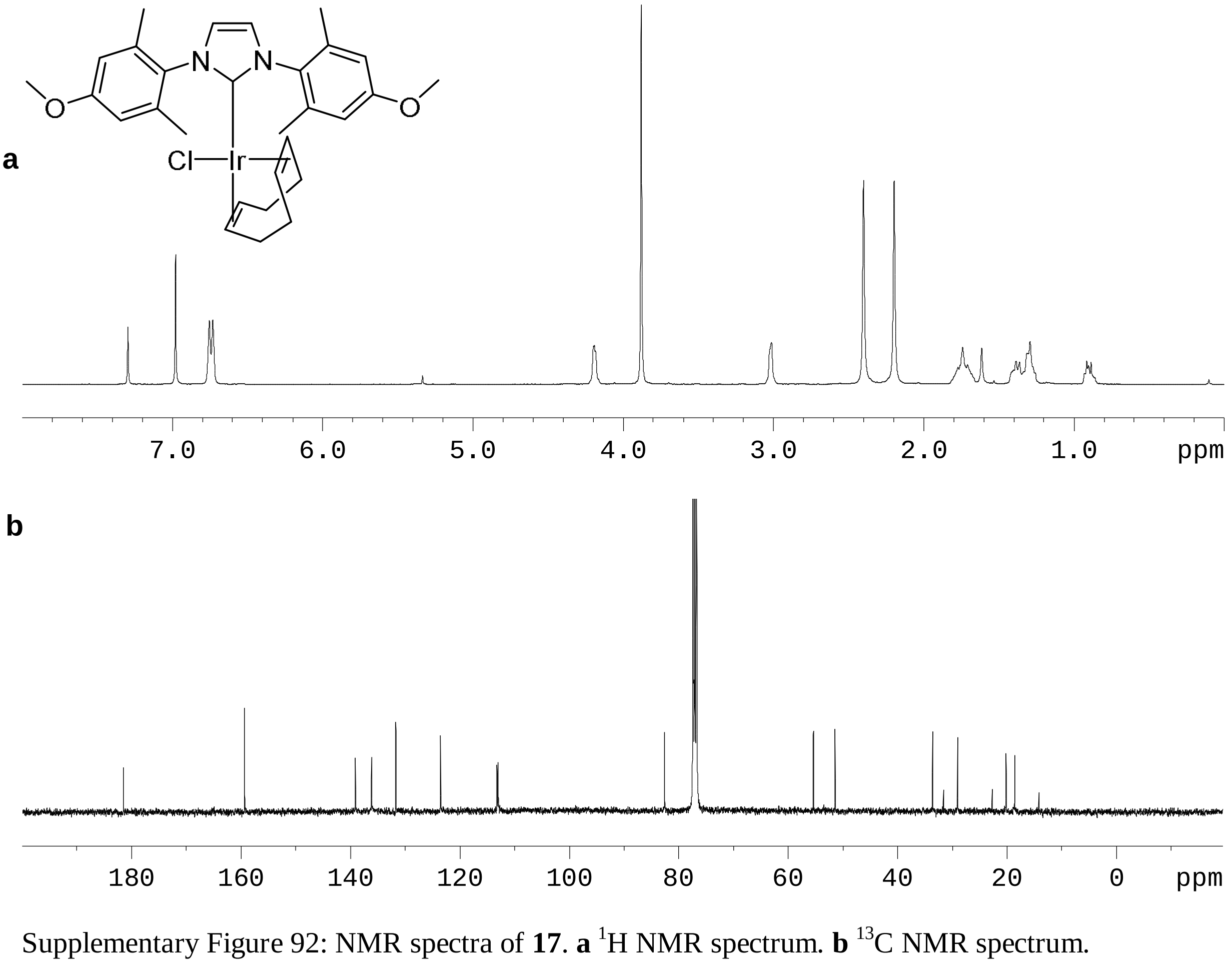


Supplementary Figure 92: NMR spectra of **17**. **a** $^{1}$H NMR spectrum. **b** $^{13}$C NMR spectrum.

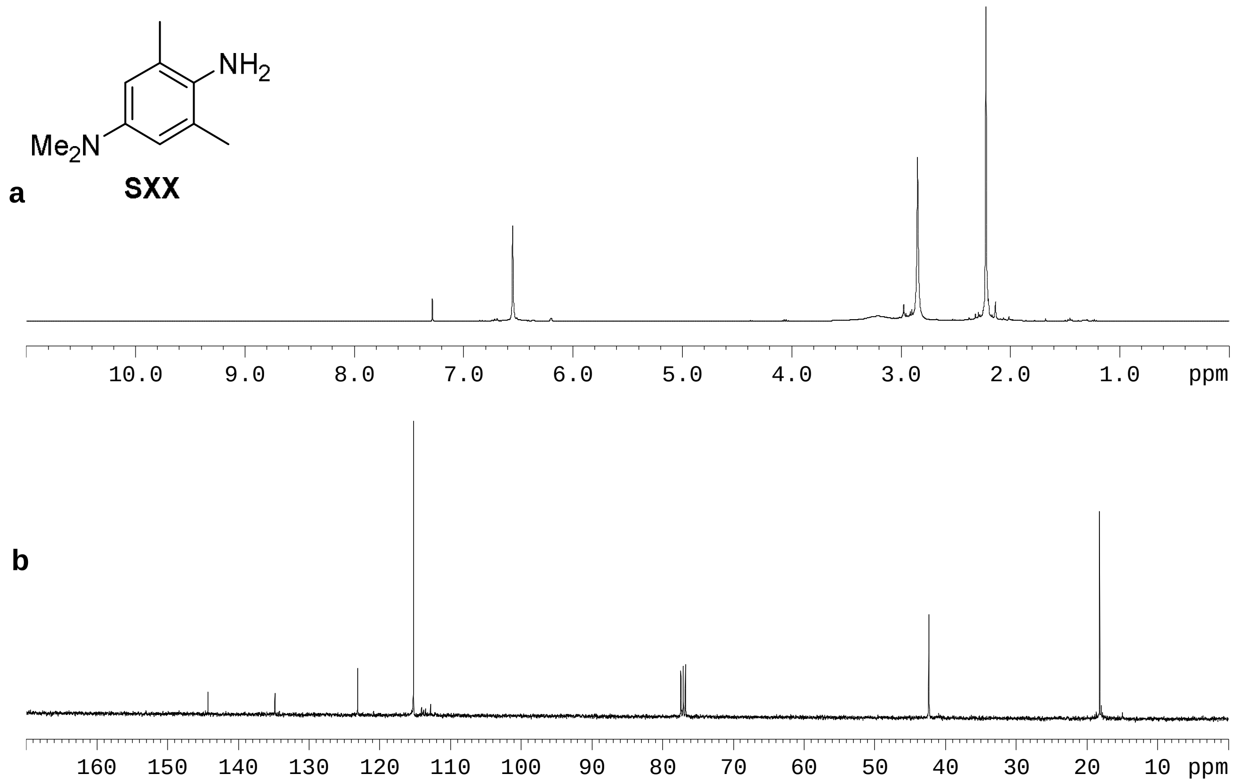


Supplementary Figure 93: NMR spectra of **S33**. **a** $^{1}$H NMR spectrum. **b** $^{13}$C NMR spectrum.

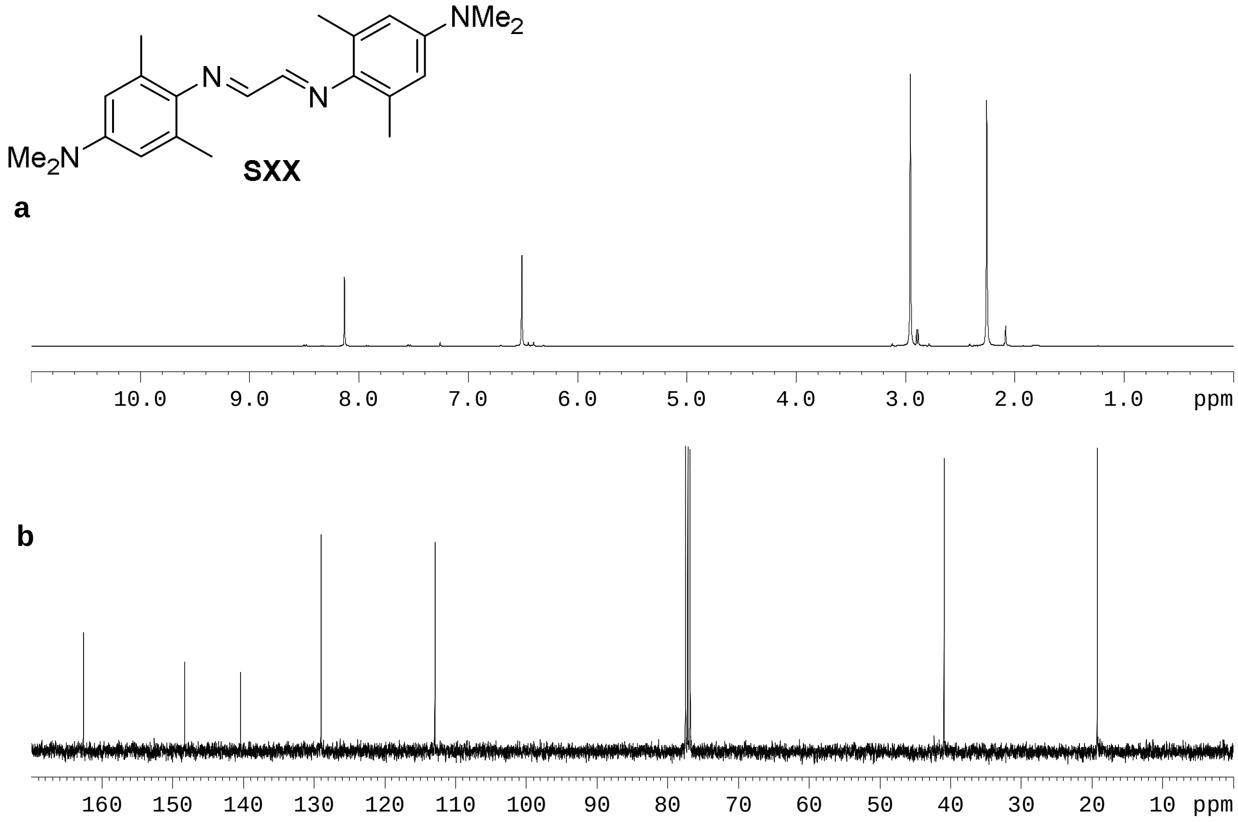


Supplementary Figure 94: NMR spectra of **S34**. **a** $^{1}$H NMR spectrum. **b** $^{13}$C NMR spectrum.

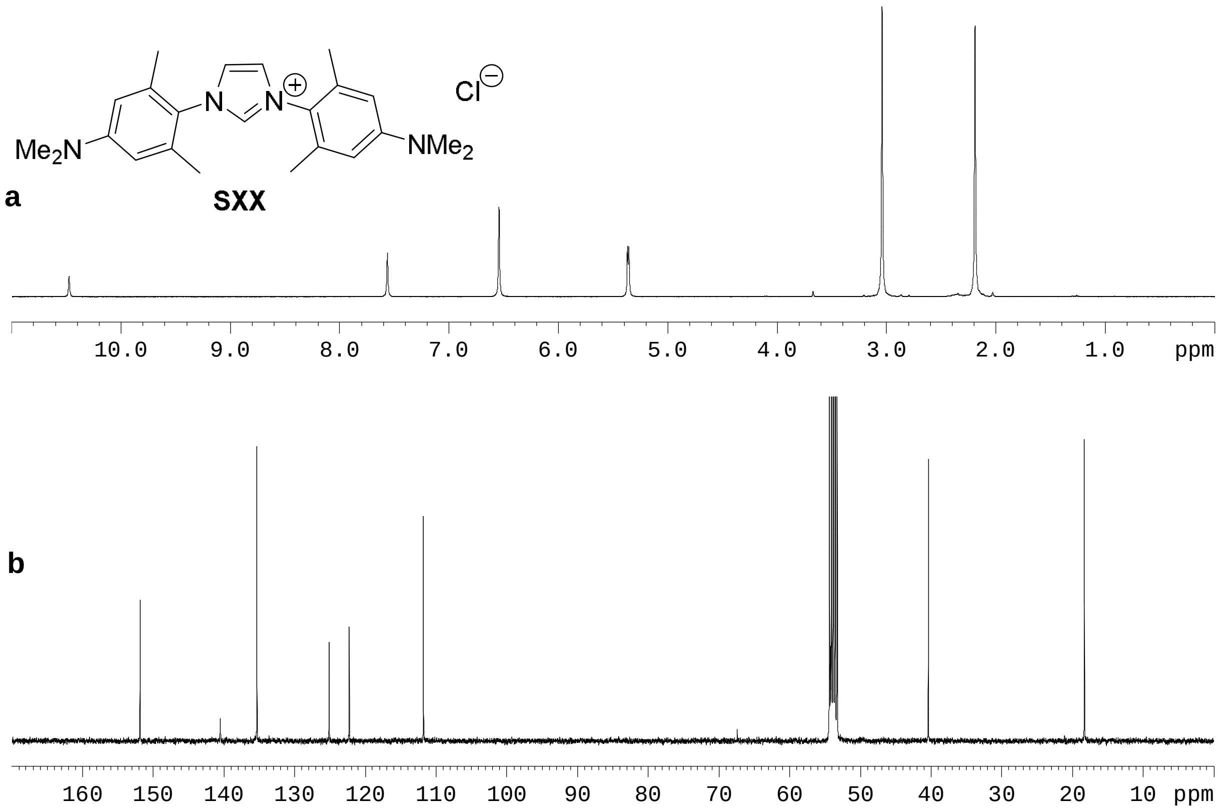


Supplementary Figure 95: NMR spectra of **S35**. **a** $^{1}$H NMR spectrum. **b** $^{13}$C NMR spectrum.

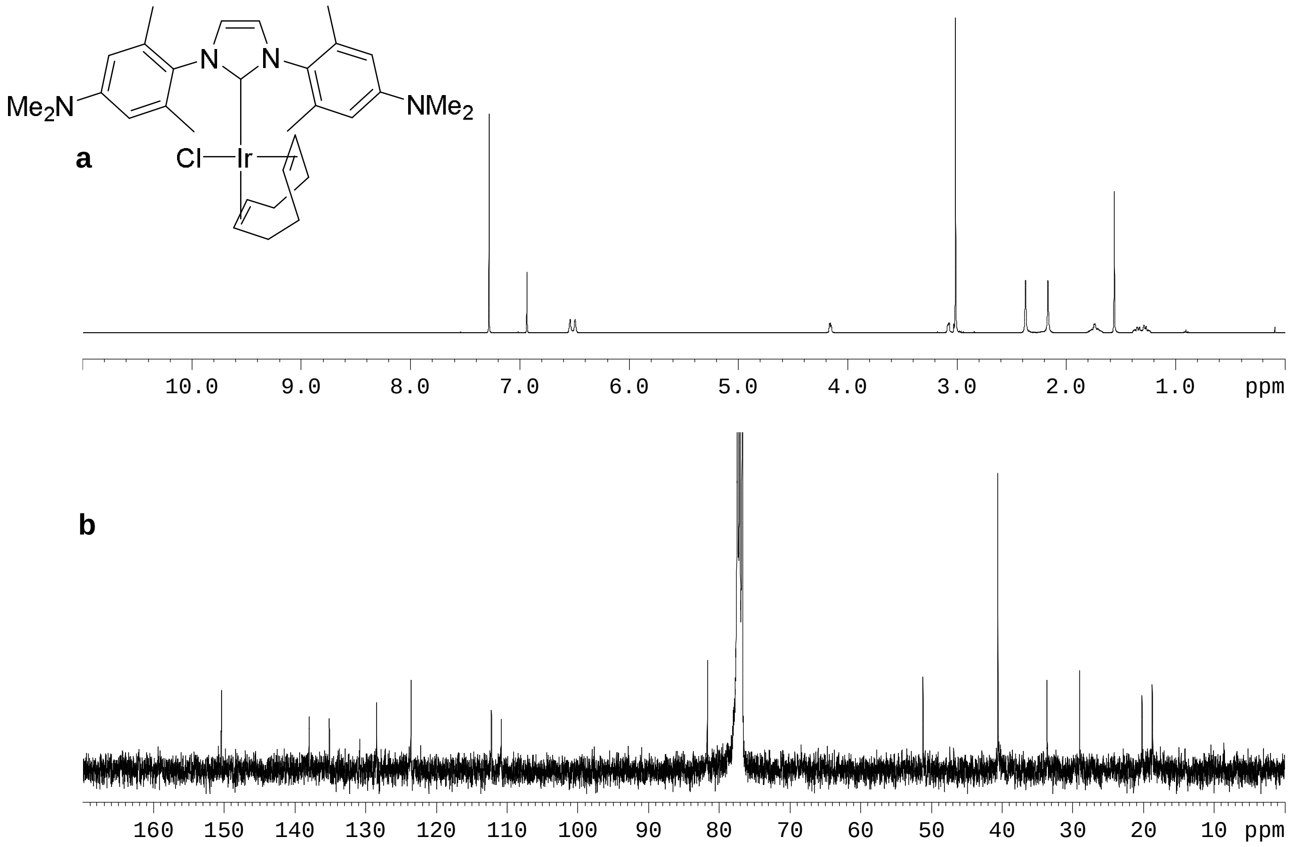


Supplementary Figure 96: NMR spectra of **18**. **a** $^{1}$H NMR spectrum. **b** $^{13}$C NMR spectrum.

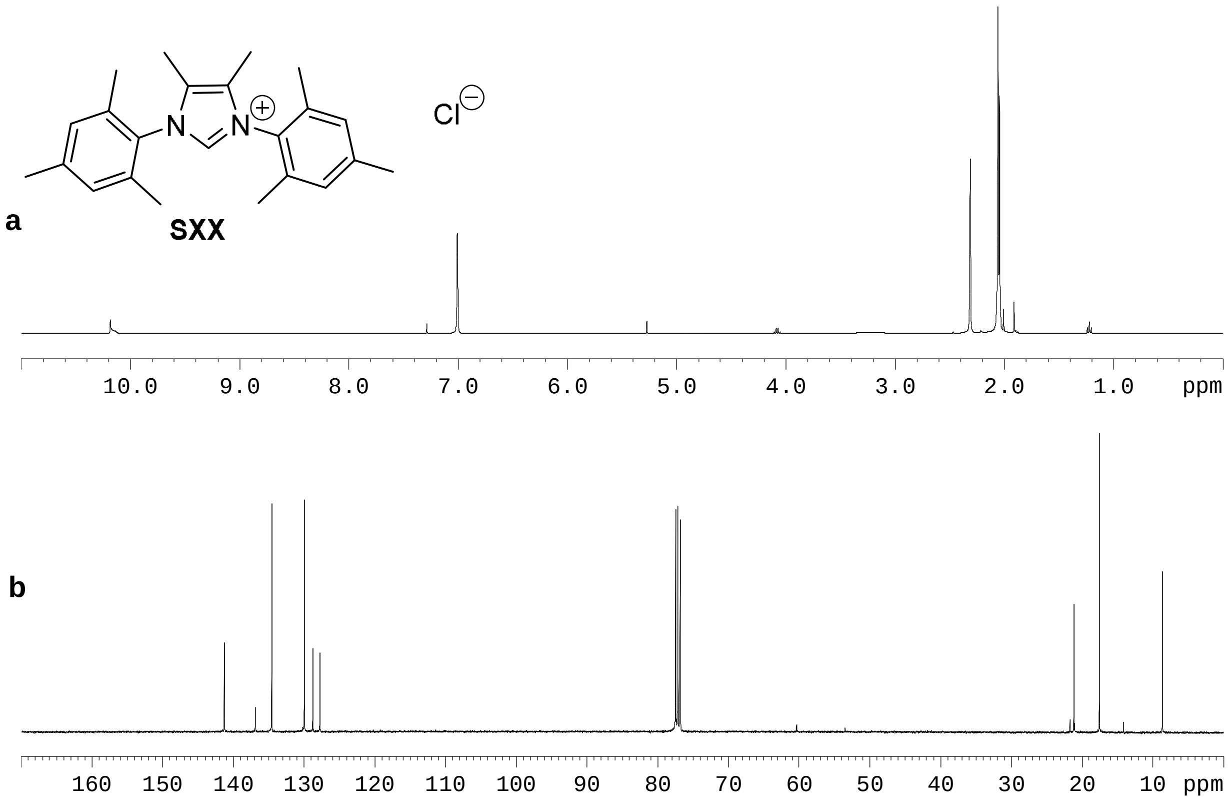


Supplementary Figure 97: NMR spectra of **S37**. **a** $^{1}$H NMR spectrum. **b** $^{13}$C NMR spectrum.

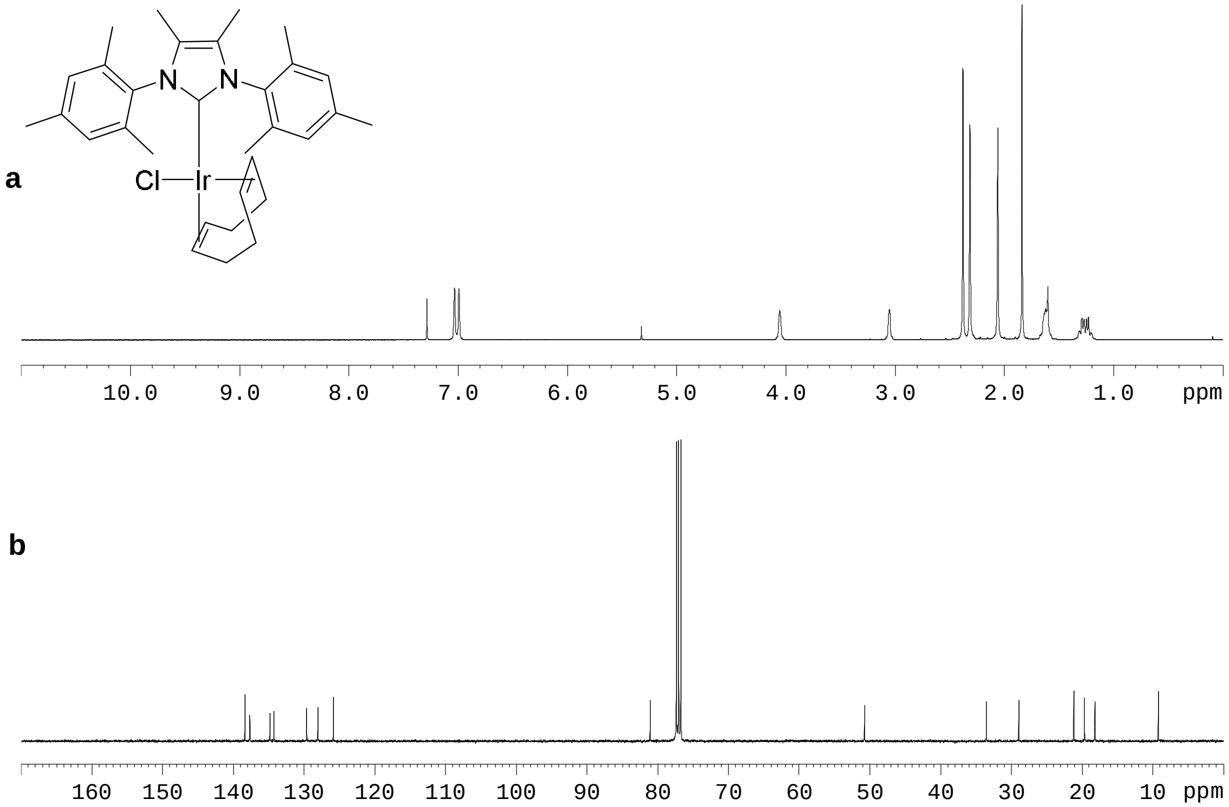


Supplementary Figure 98: NMR spectra of **19**. **a** $^{1}$H NMR spectrum. **b** $^{13}$C NMR spectrum.

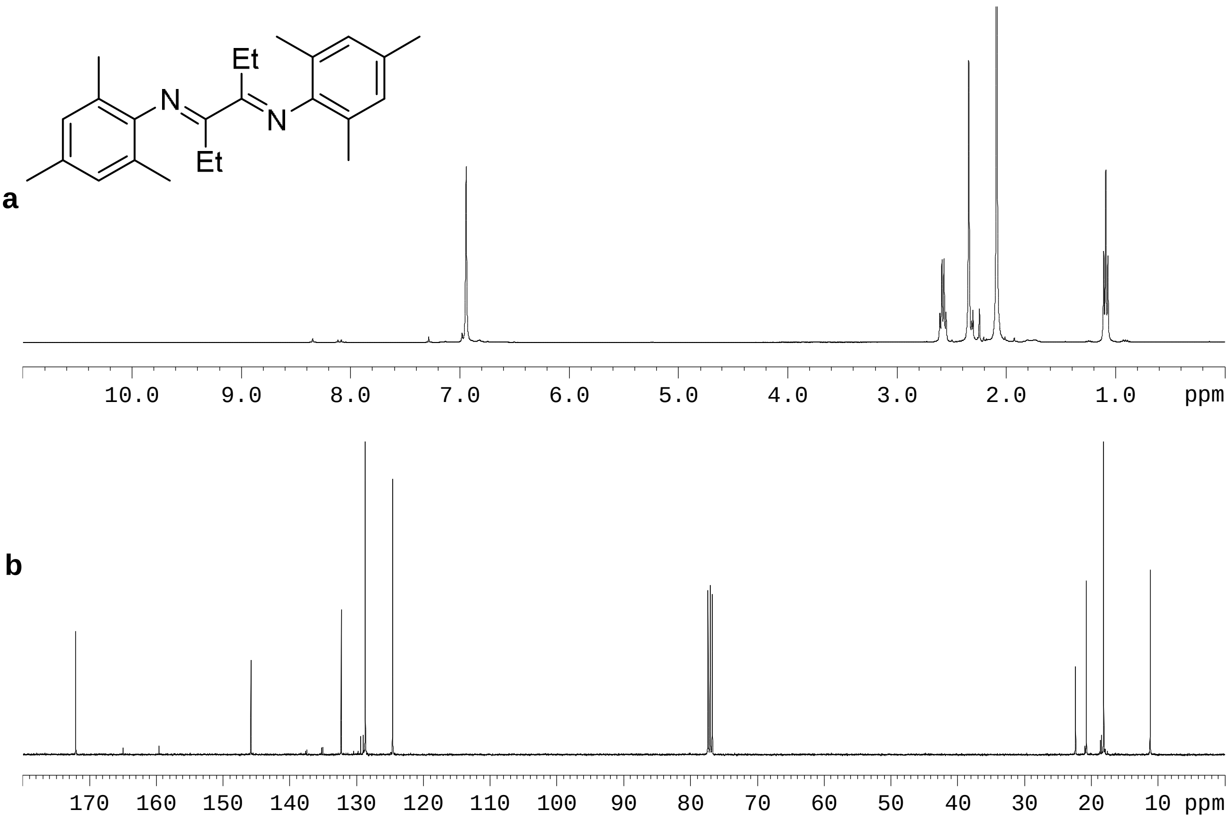


Supplementary Figure 99: NMR spectra of **S38**. **a** $^{1}$H NMR spectrum. **b** $^{13}$C NMR spectrum.

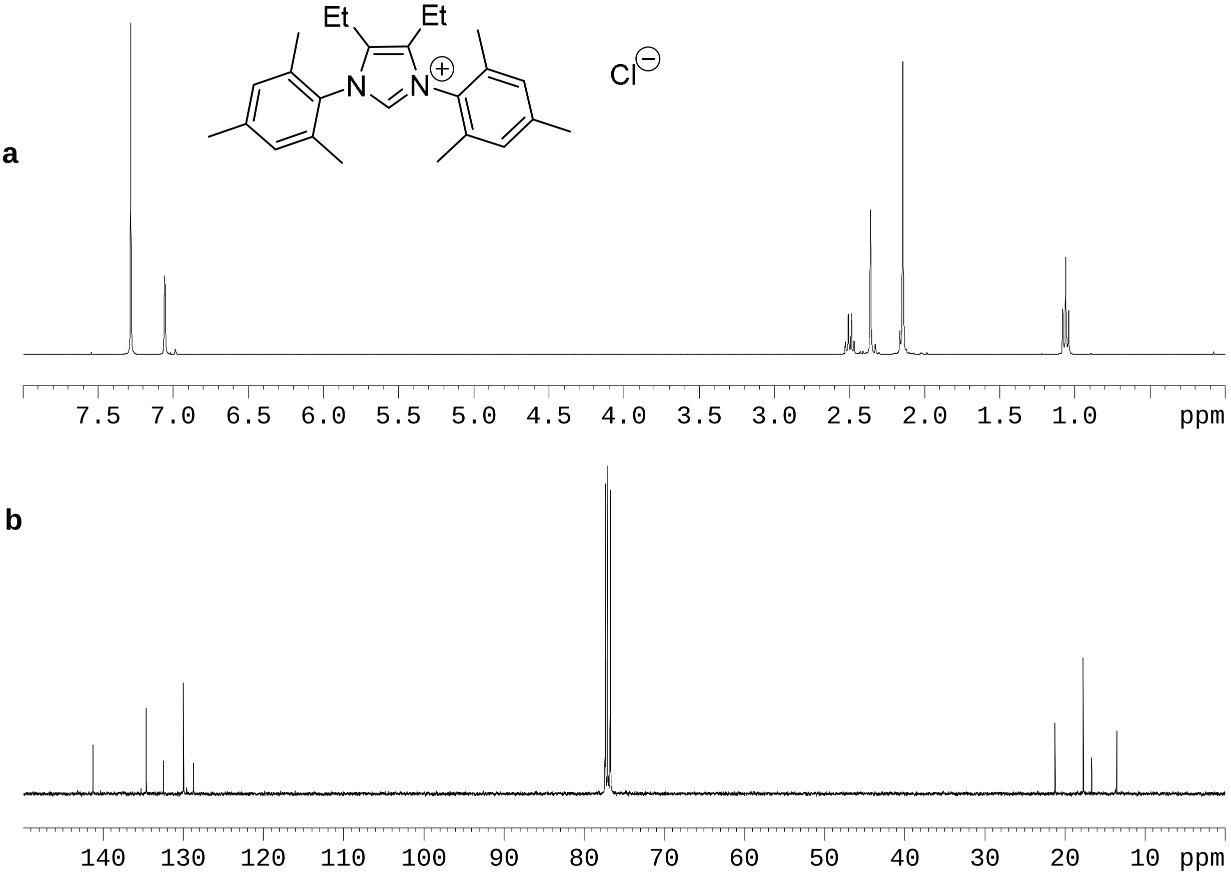


Supplementary Figure 100: NMR spectra of **S39**. **a** $^{1}$H NMR spectrum. **b** $^{13}$C NMR spectrum.

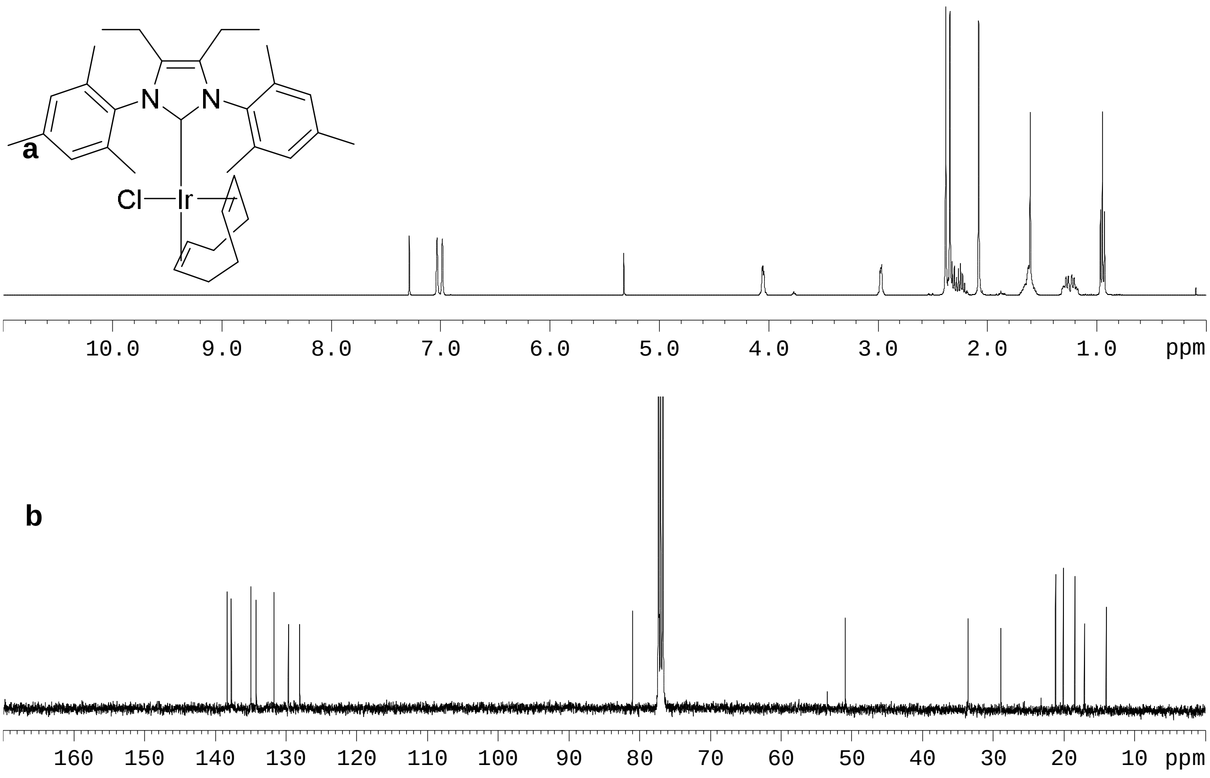


Supplementary Figure 101: NMR spectra of **20**. **a** $^{1}$H NMR spectrum. **b** $^{13}$C NMR spectrum.

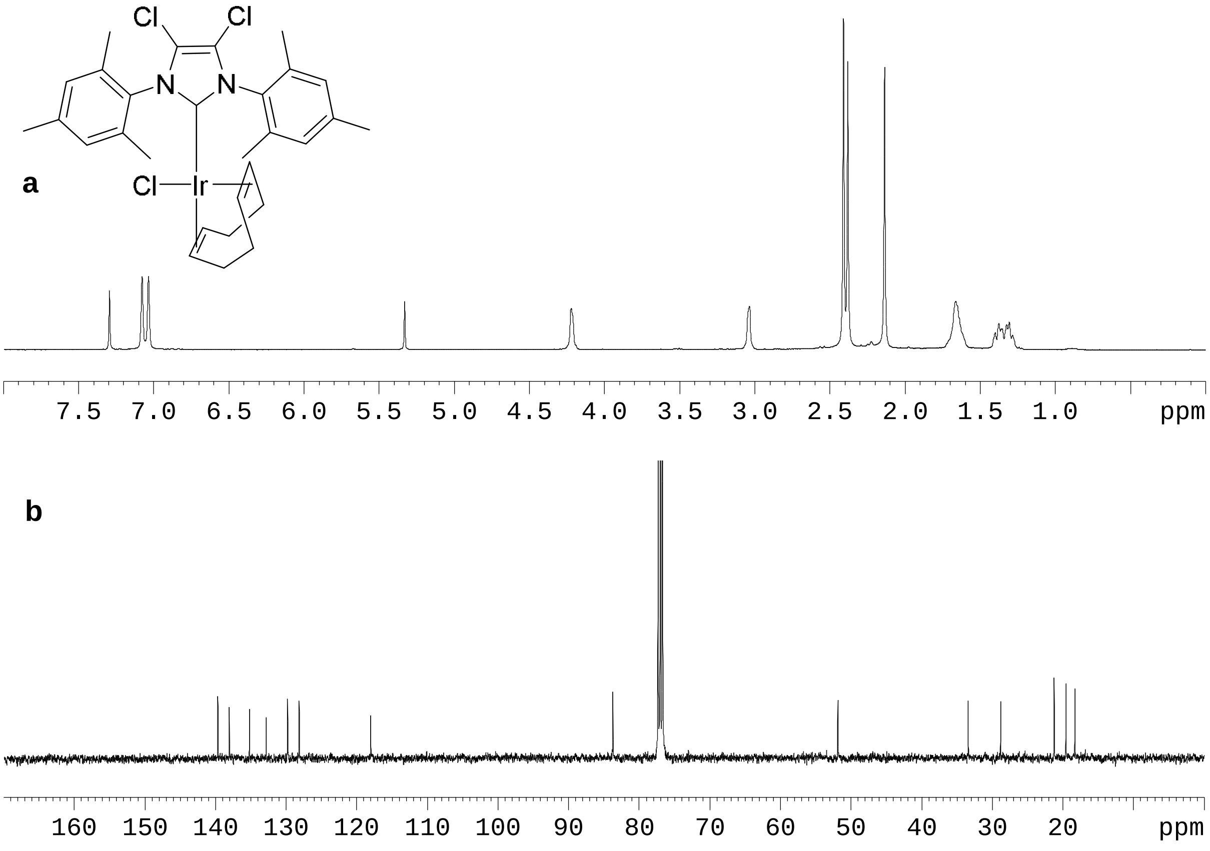


Supplementary Figure 102: NMR spectra of **21**. **a** $^{1}$H NMR spectrum. **b** $^{13}$C NMR spectrum.

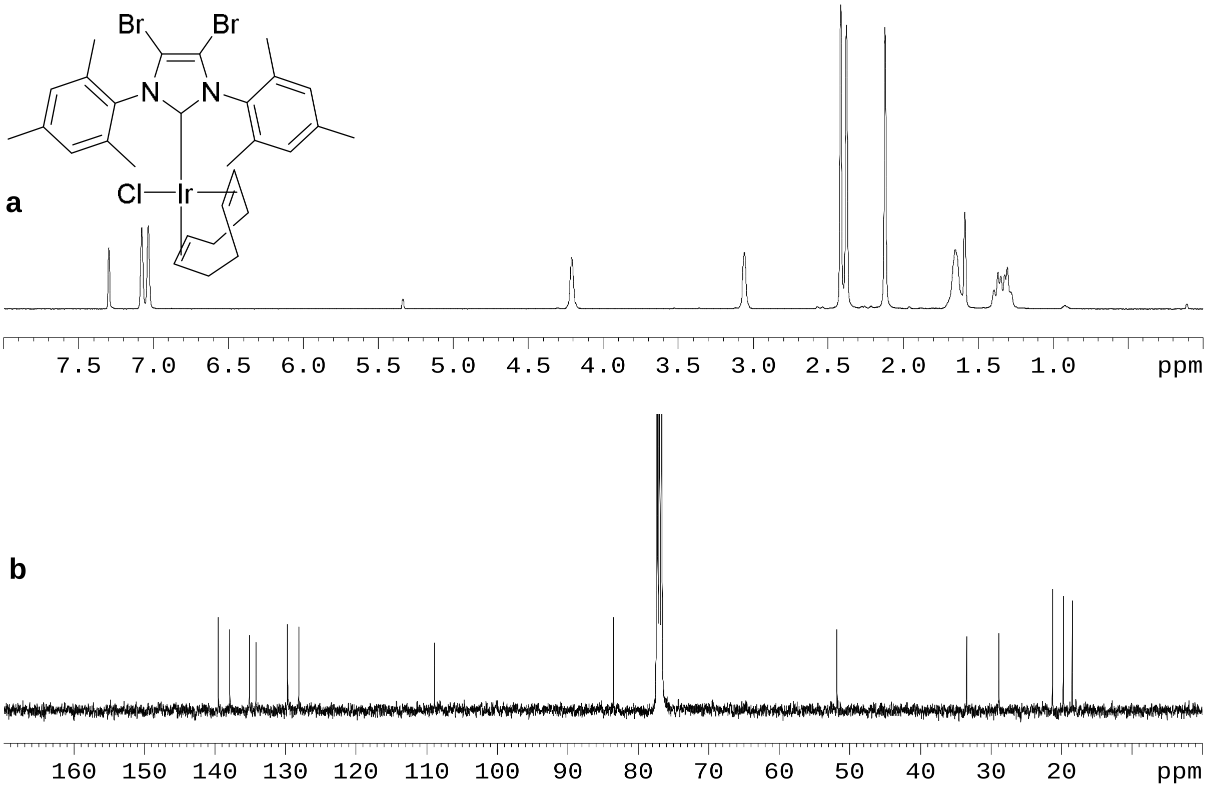


Supplementary Figure 103: NMR spectra of **22**. **a** $^{1}$H NMR spectrum. **b** $^{13}$C NMR spectrum.

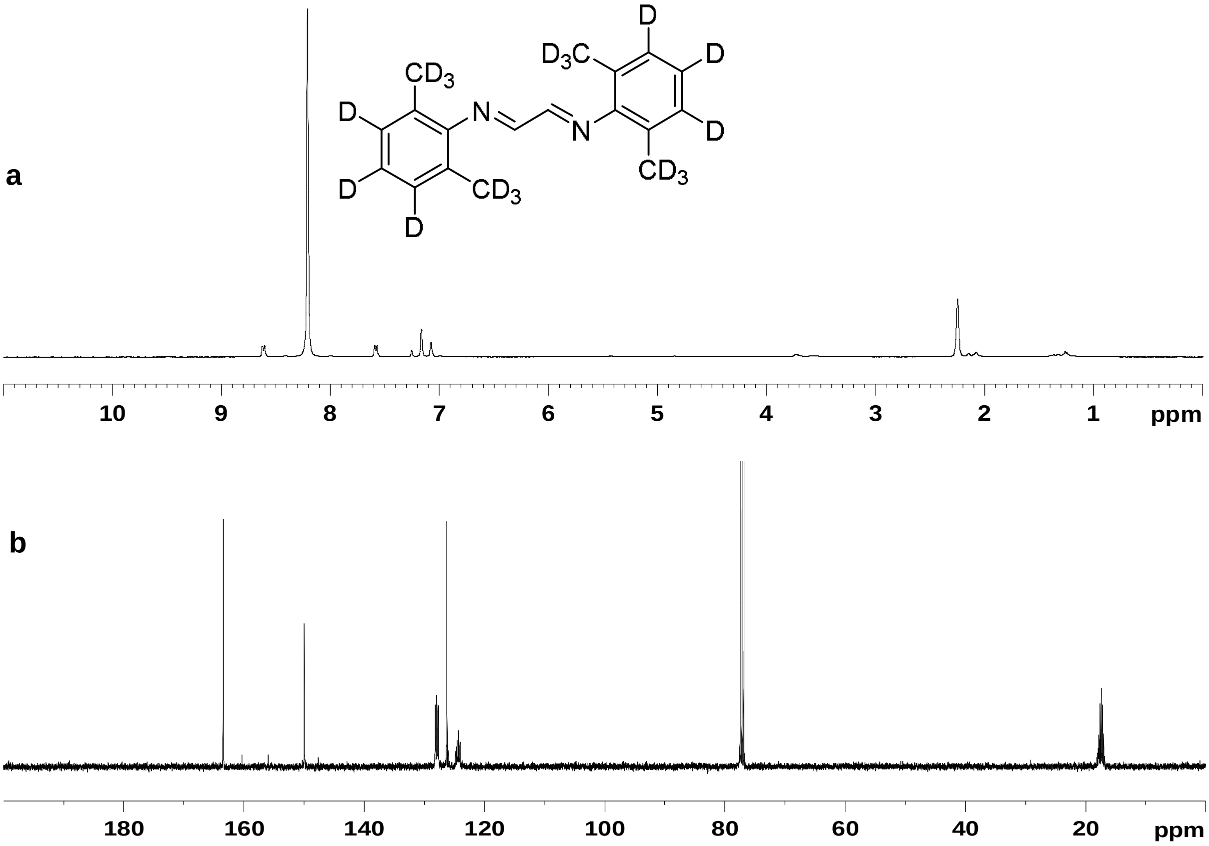


Supplementary Figure 104: NMR spectra of ***d*₁₈-S3**. **a** $^{1}$H NMR spectrum. **b** $^{13}$C NMR spectrum.

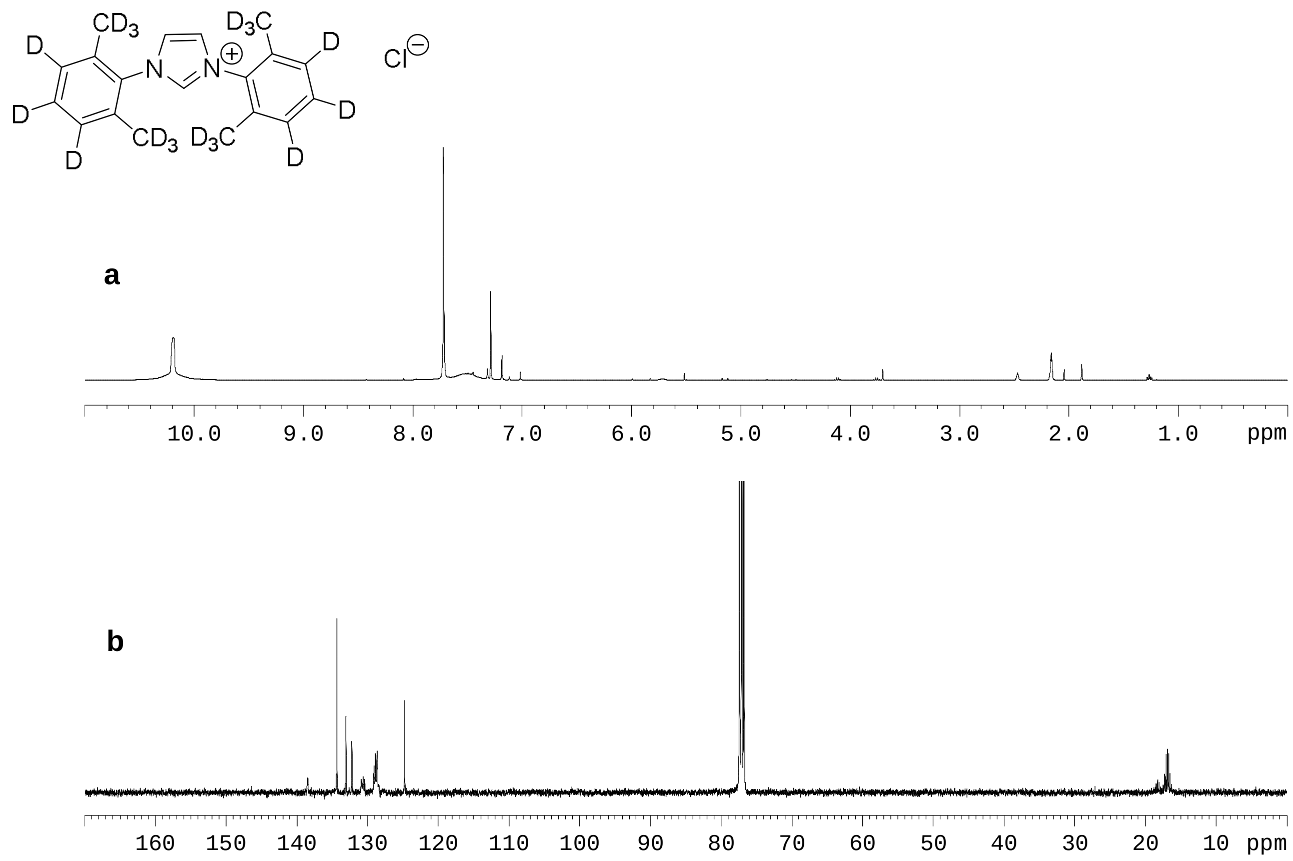


Supplementary Figure 105: NMR spectra of $\boldsymbol{d_{18}}$-**S4**. **a** $^{1}$H NMR spectrum. **b** $^{13}$C NMR spectrum.

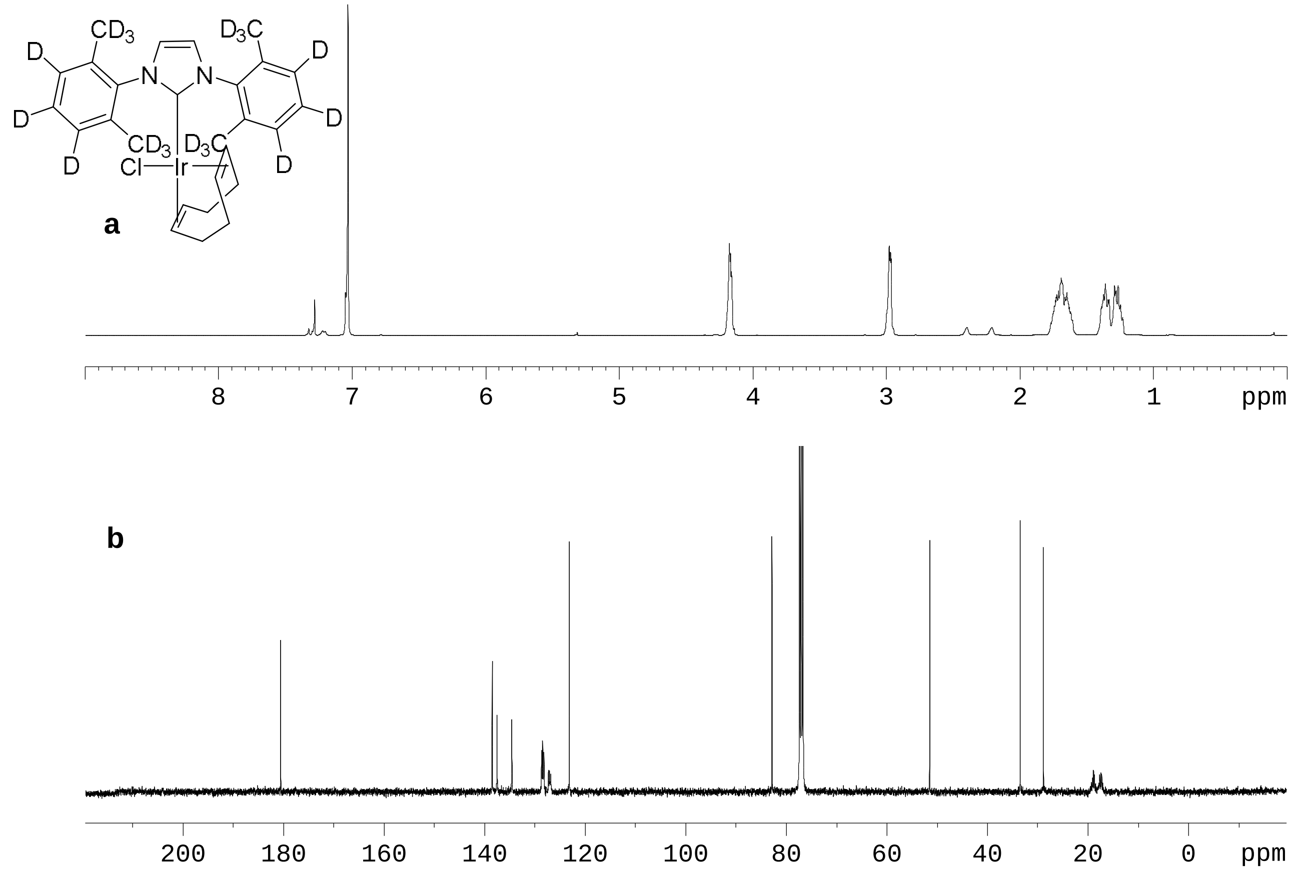


Supplementary Figure 106: NMR spectra of $\boldsymbol{d_{18}}$-**3**. **a** $^{1}$H NMR spectrum. **b** $^{13}$C NMR spectrum.

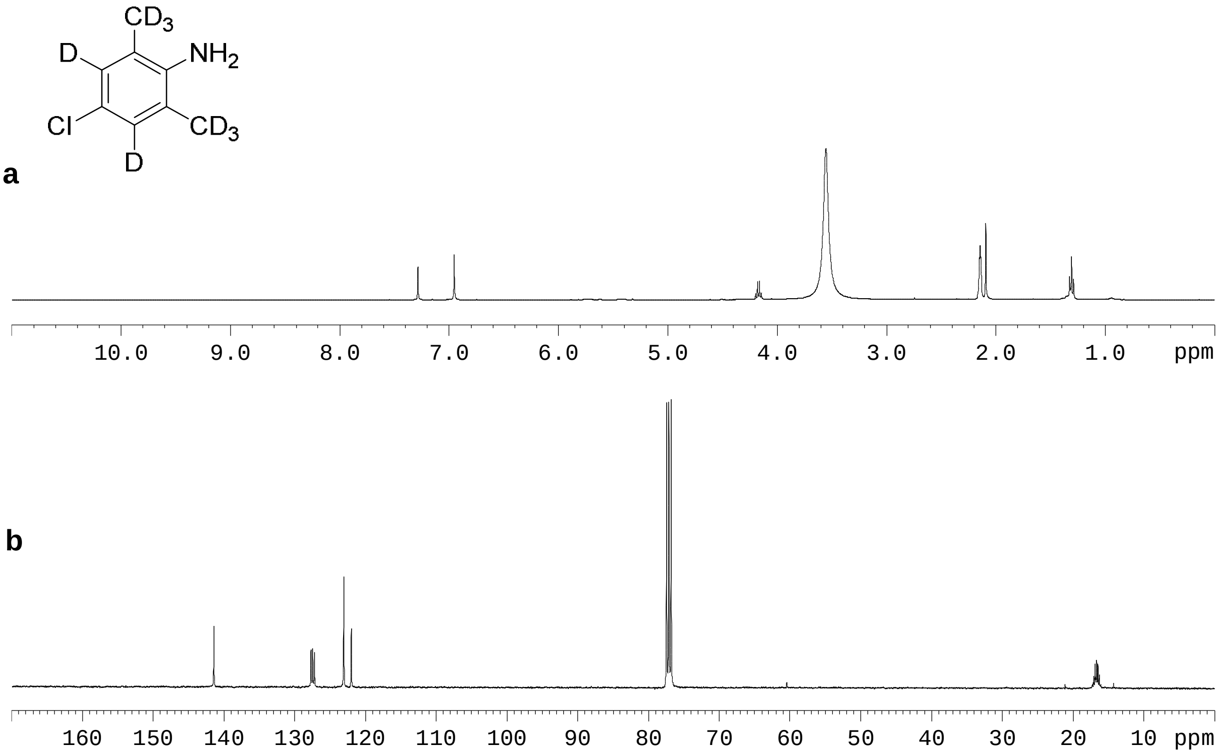


Supplementary Figure 107: NMR spectra of ***d*₈-S44**. **a** $^{1}$H NMR spectrum. **b** $^{13}$C NMR spectrum.

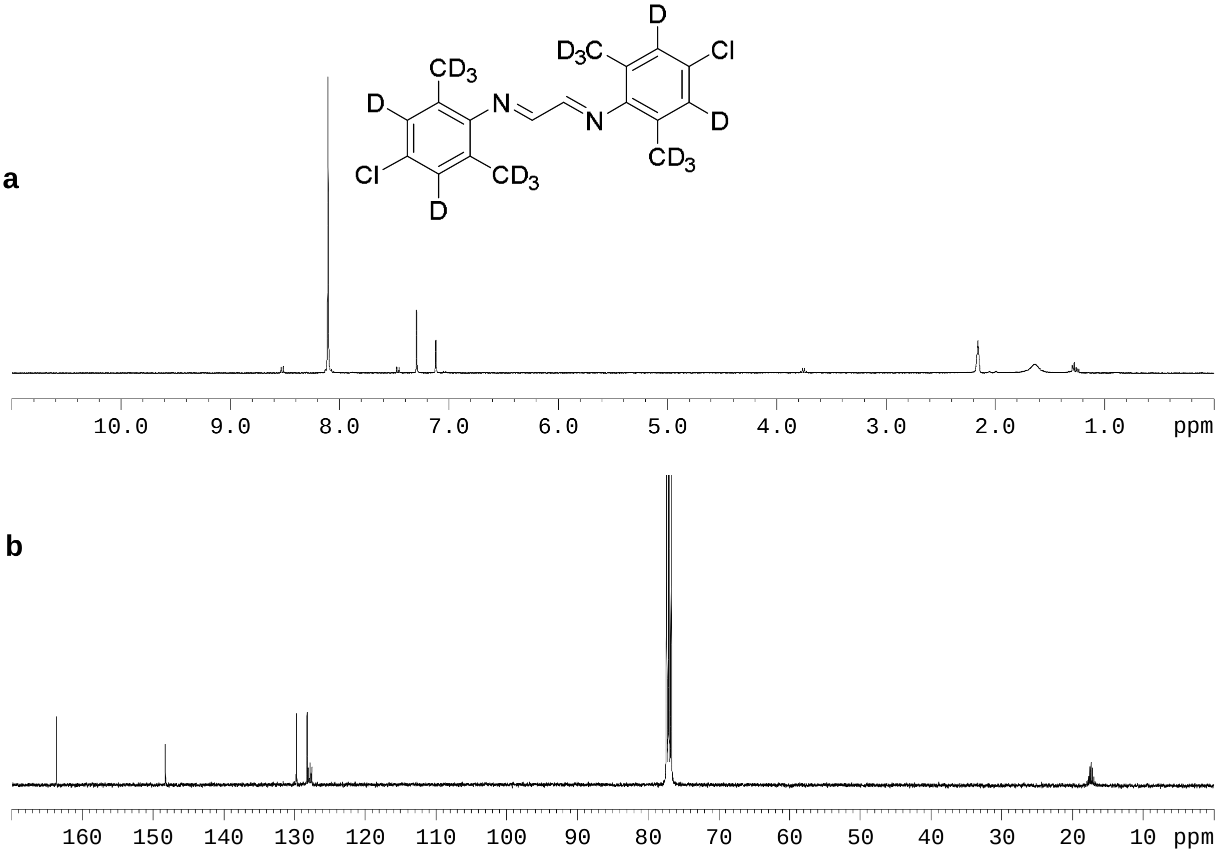


Supplementary Figure 108: NMR spectra of ***d*₁₆-S14**. **a** $^{1}$H NMR spectrum. **b** $^{13}$C NMR spectrum.

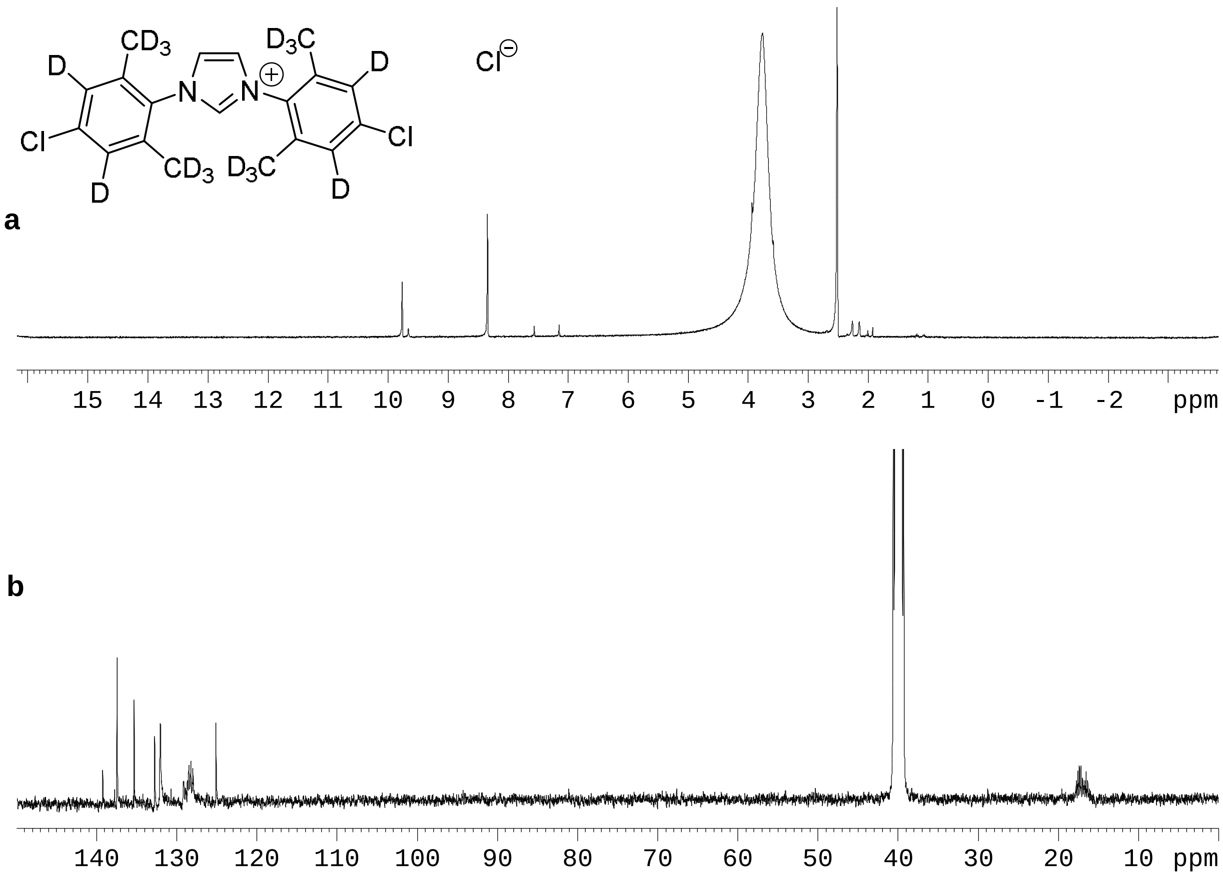


Supplementary Figure 109: NMR spectra of ***d*₁₆-S15**. **a** $^{1}$H NMR spectrum. **b** $^{13}$C NMR spectrum.

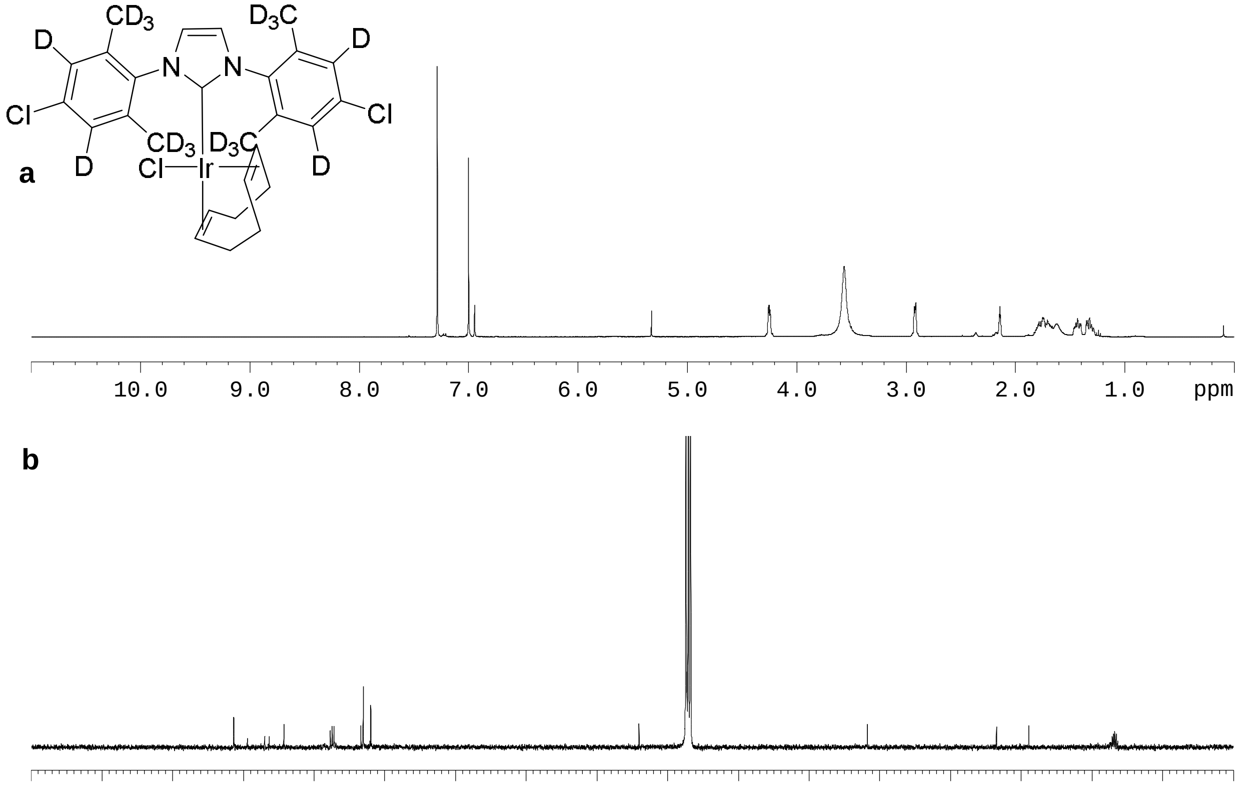


Supplementary Figure 110: NMR spectra of ***d*₁₆-9**. **a** $^{1}$H NMR spectrum. **b** $^{13}$C NMR spectrum.

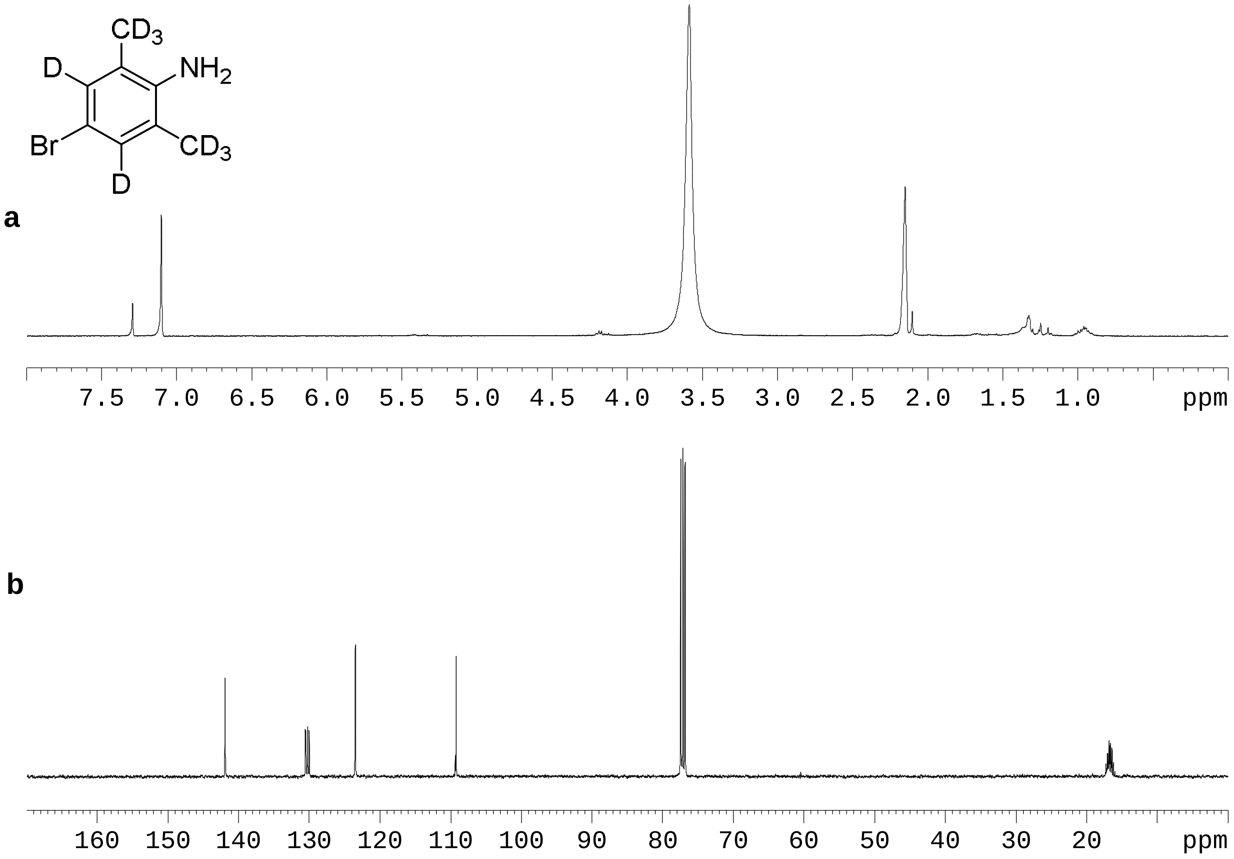


Supplementary Figure 111: NMR spectra of ***d*$_8$-S45**. **a** $^1$H NMR spectrum. **b** $^{13}$C NMR spectrum.

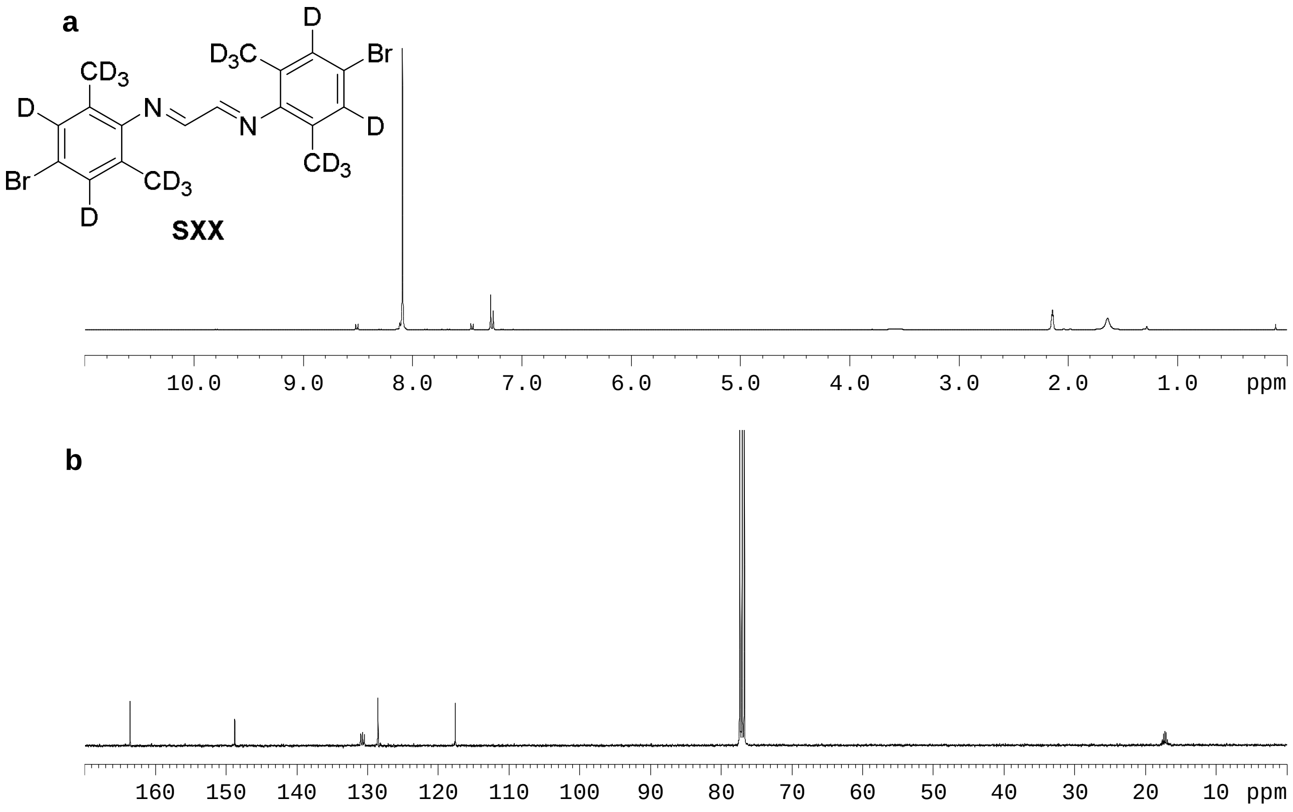


Supplementary Figure 112: NMR spectra of ***d*$_{16}$-S16**. **a** $^1$H NMR spectrum. **b** $^{13}$C NMR spectrum.

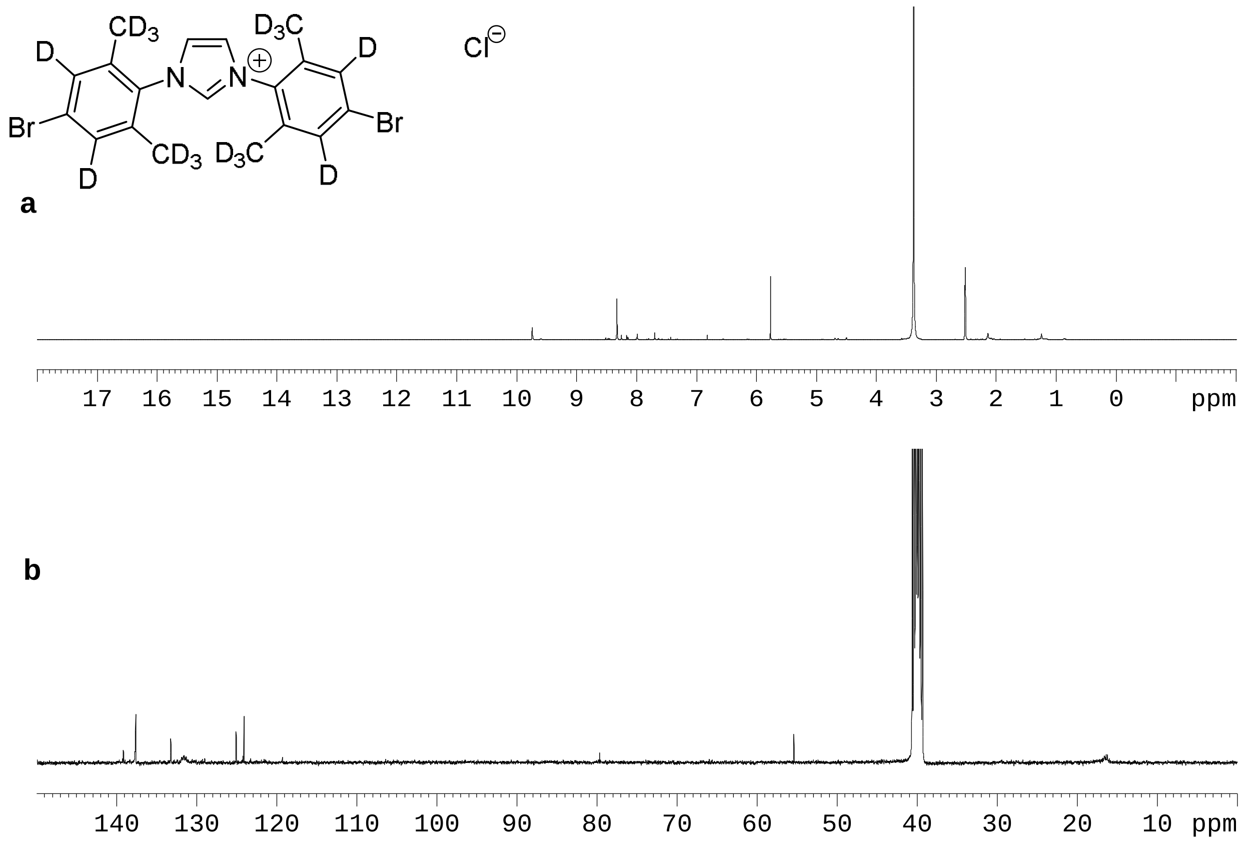


Supplementary Figure 113: NMR spectra of ***d*<sub></sub>**$\boldsymbol{d_{16}}$**-S17**. **a** $^{1}$H NMR spectrum. **b** $^{13}$C NMR spectrum.

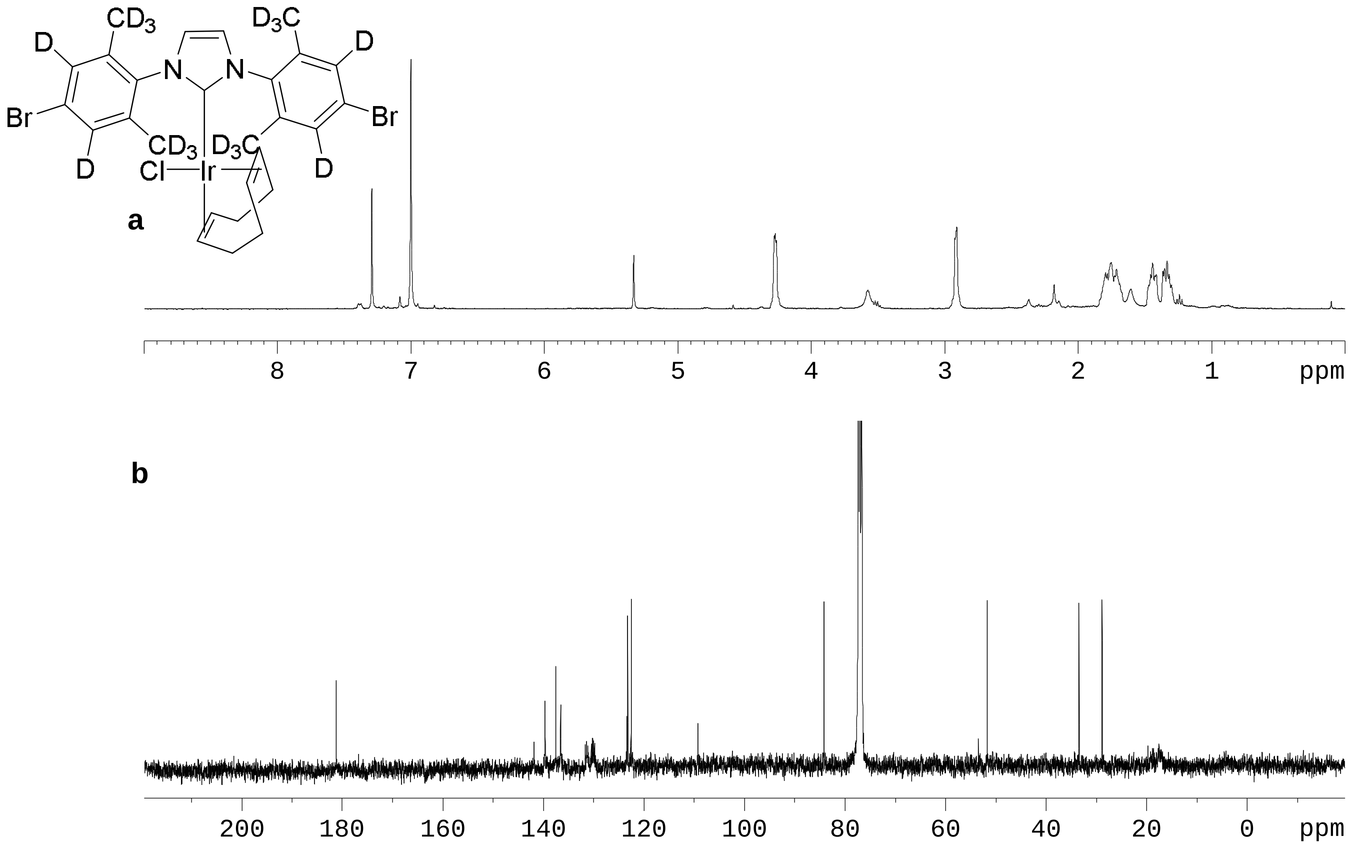


Supplementary Figure 114: NMR spectra of $\boldsymbol{d_{16}}$**-10**. **a** $^{1}$H NMR spectrum. **b** $^{13}$C NMR spectrum.

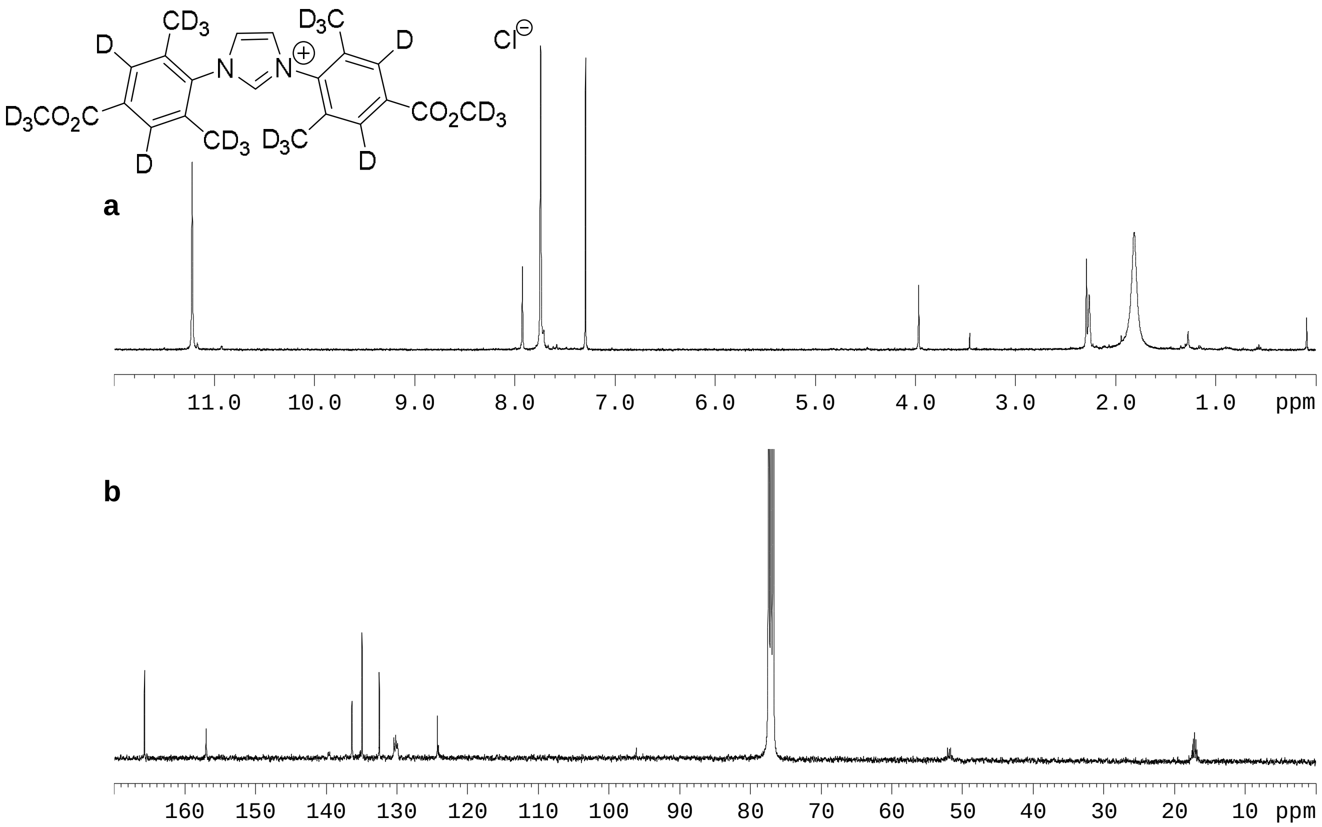


Supplementary Figure 115: NMR spectra of ***d*$_{22}$-S21**. **a** $^{1}$H NMR spectrum. **b** $^{13}$C NMR spectrum.

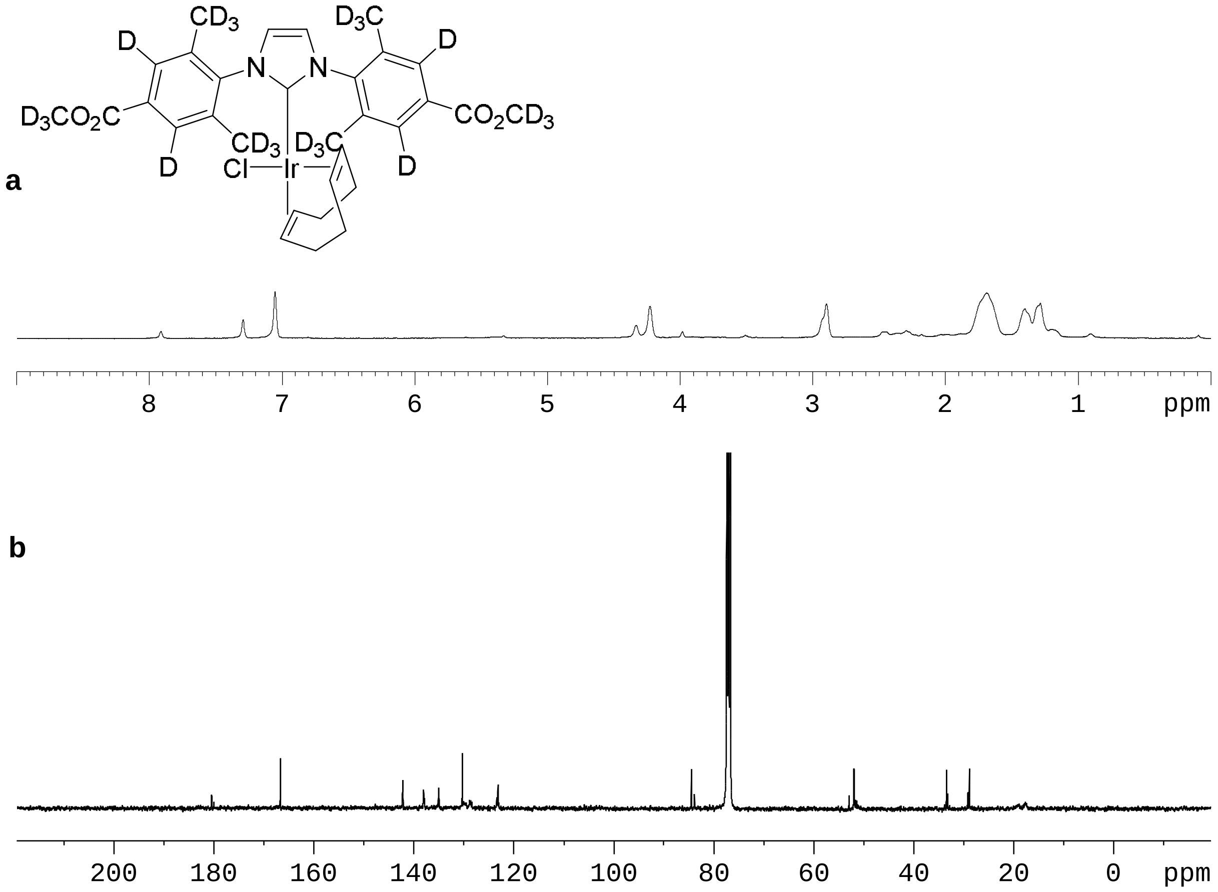


Supplementary Figure 116: NMR spectra of ***d*$_{22}$-12**. **a** $^{1}$H NMR spectrum. **b** $^{13}$C NMR spectrum.

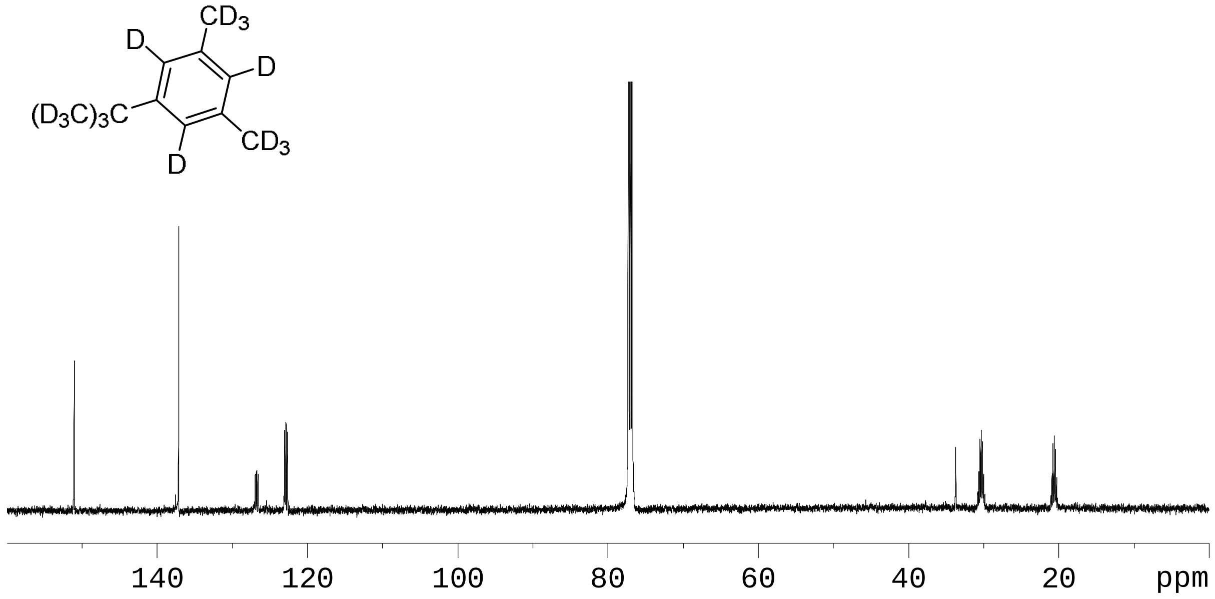


Supplementary Figure 117: $^{13}$C NMR spectrum of ***d*$_{18}$-S46**

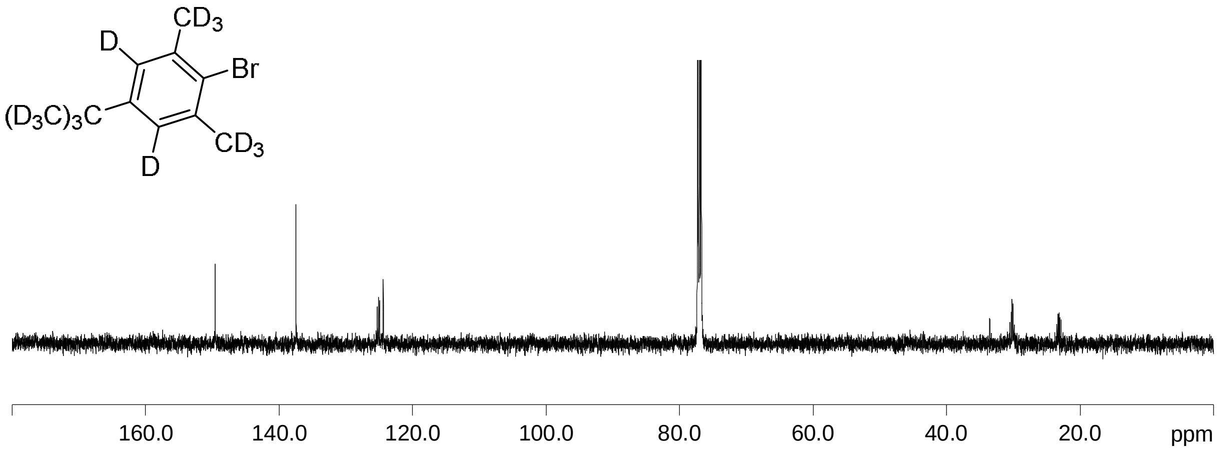


Supplementary Figure 118: $^{13}$C NMR spectrum of ***d*$_{17}$-S47**

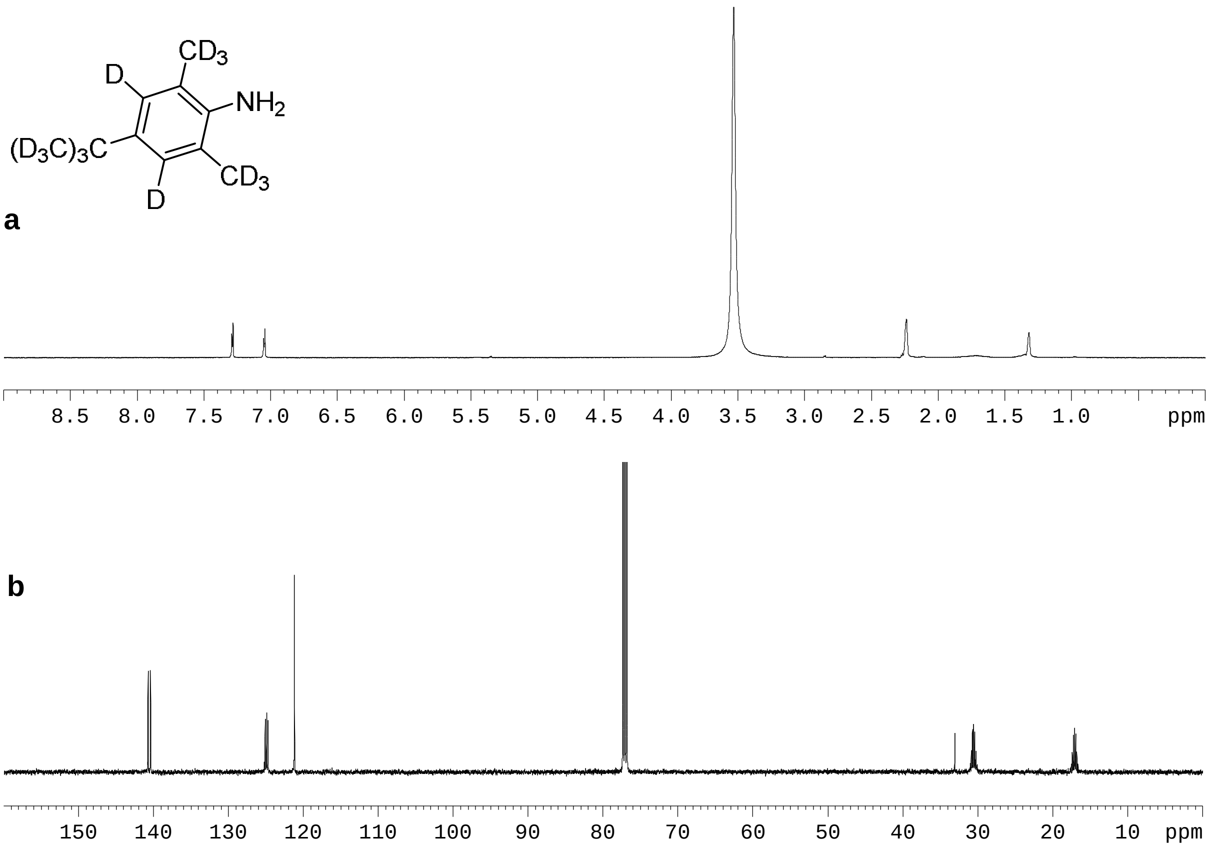


Supplementary Figure 119: NMR spectra of ***d*₁₇-S48**. **a** $^{1}$H NMR spectrum. **b** $^{13}$C NMR spectrum.

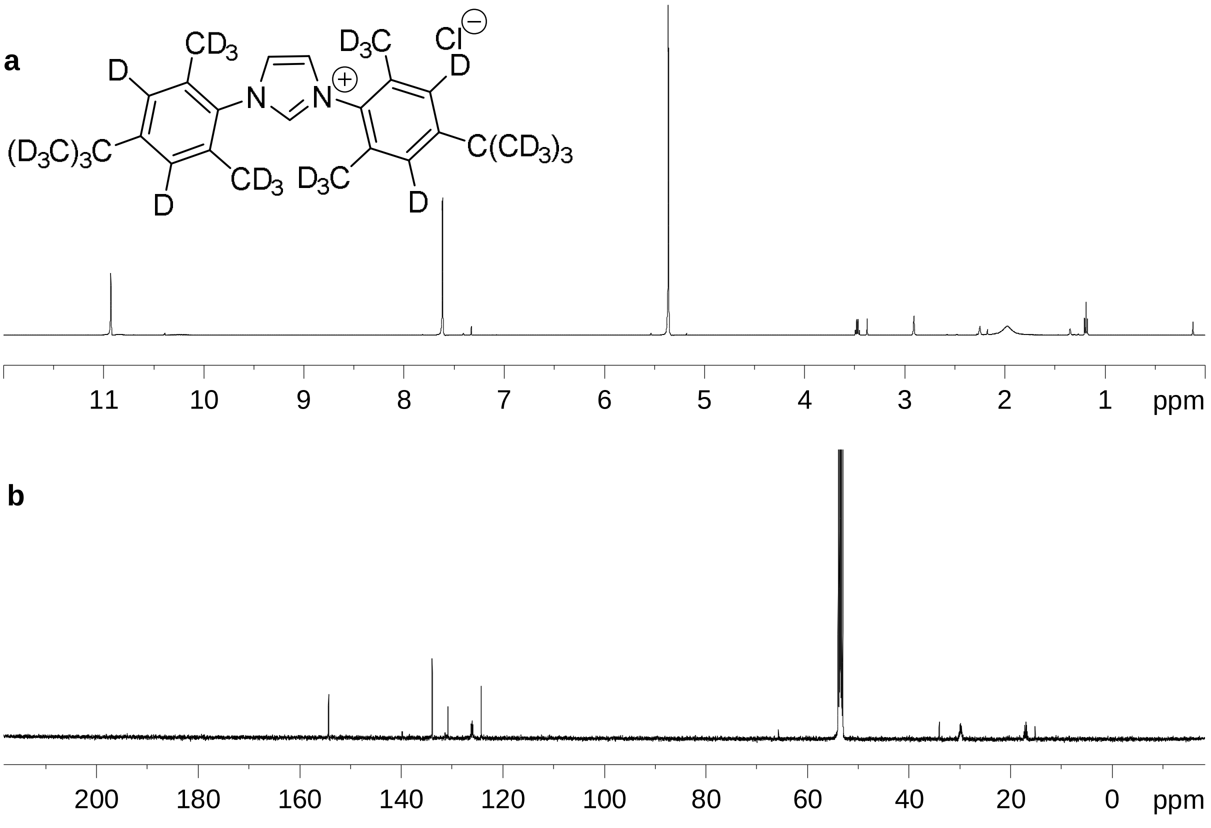


Supplementary Figure 120: NMR spectra of ***d*₃₄-S30**. **a** $^{1}$H NMR spectrum. **b** $^{13}$C NMR spectrum.

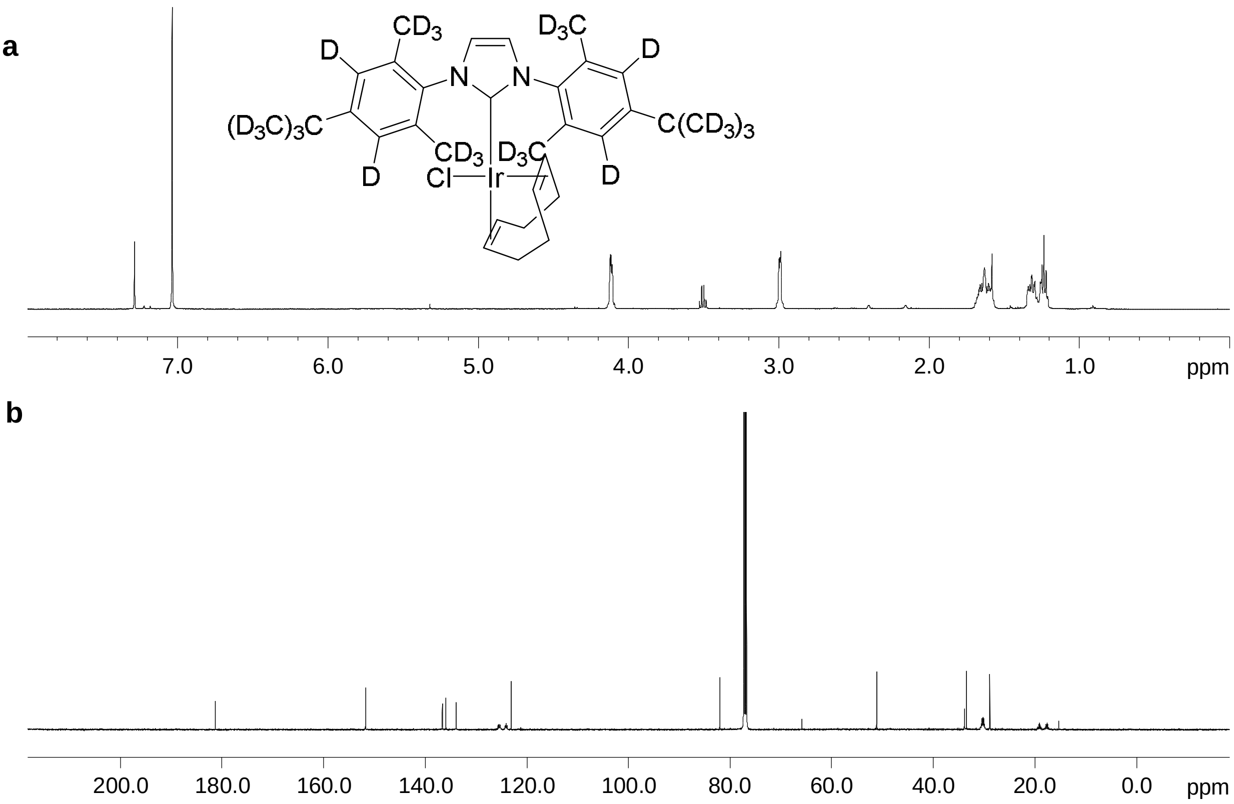


Supplementary Figure 121: NMR spectra of $\boldsymbol{d_{34}}$**-16**. **a** $^{1}$H NMR spectrum. **b** $^{13}$C NMR spectrum.

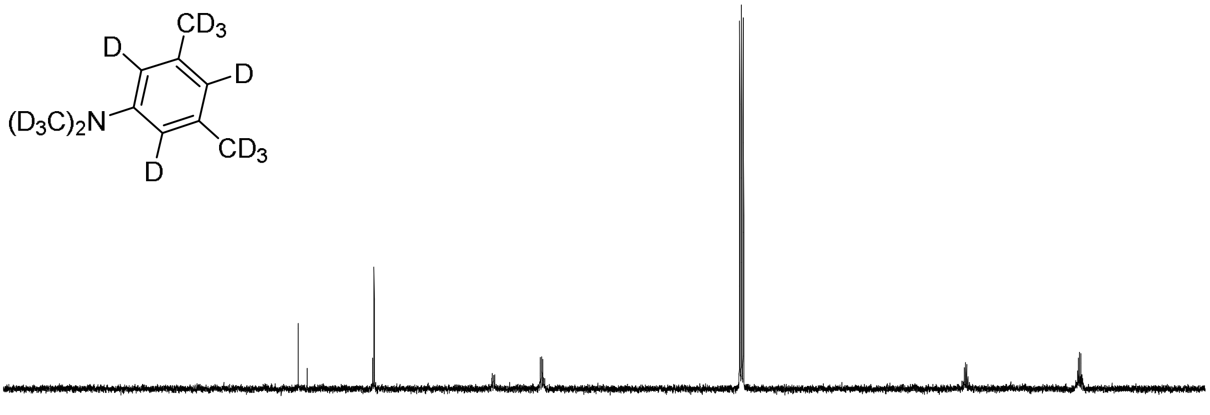


Supplementary Figure 122: $^{13}$C NMR spectrum of $\boldsymbol{d_{15}}$**-49**

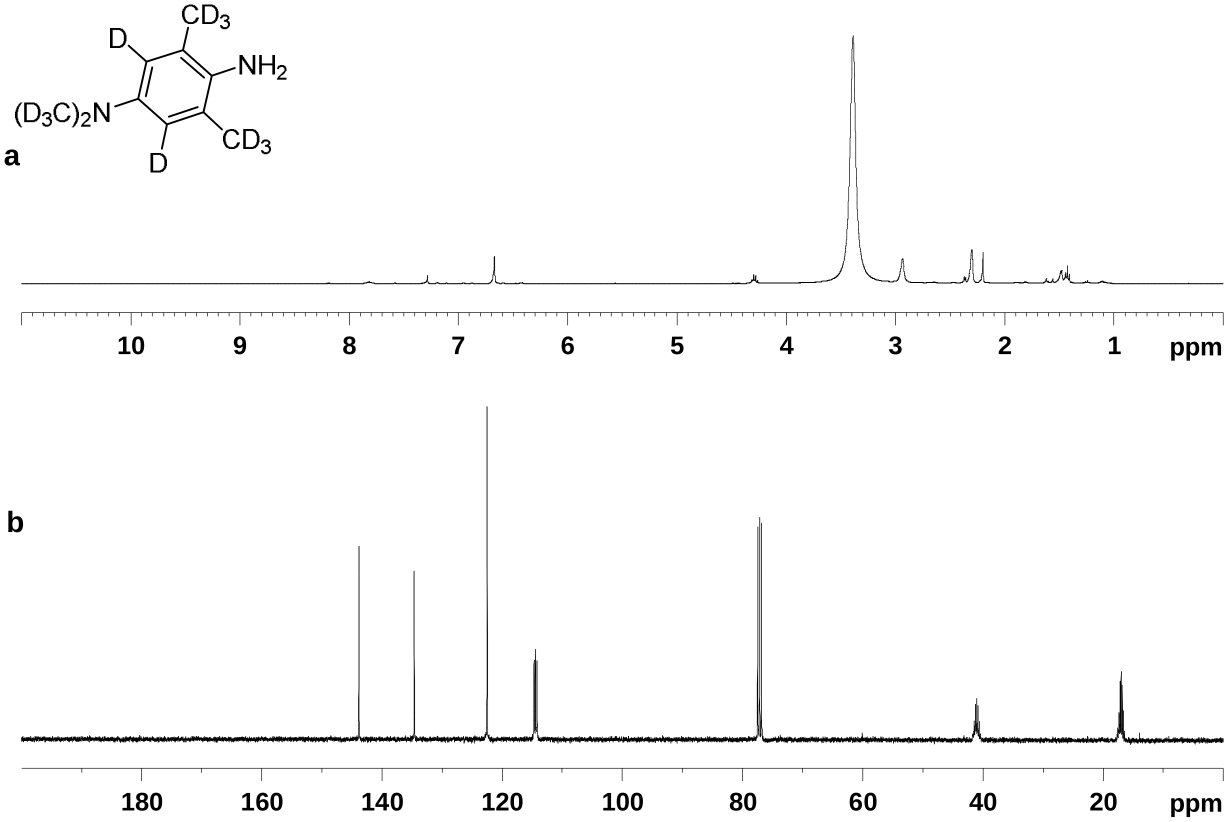


Supplementary Figure 123: NMR spectra of ***d*$_{14}$-S33**. **a** $^{1}$H NMR spectrum. **b** $^{13}$C NMR spectrum.

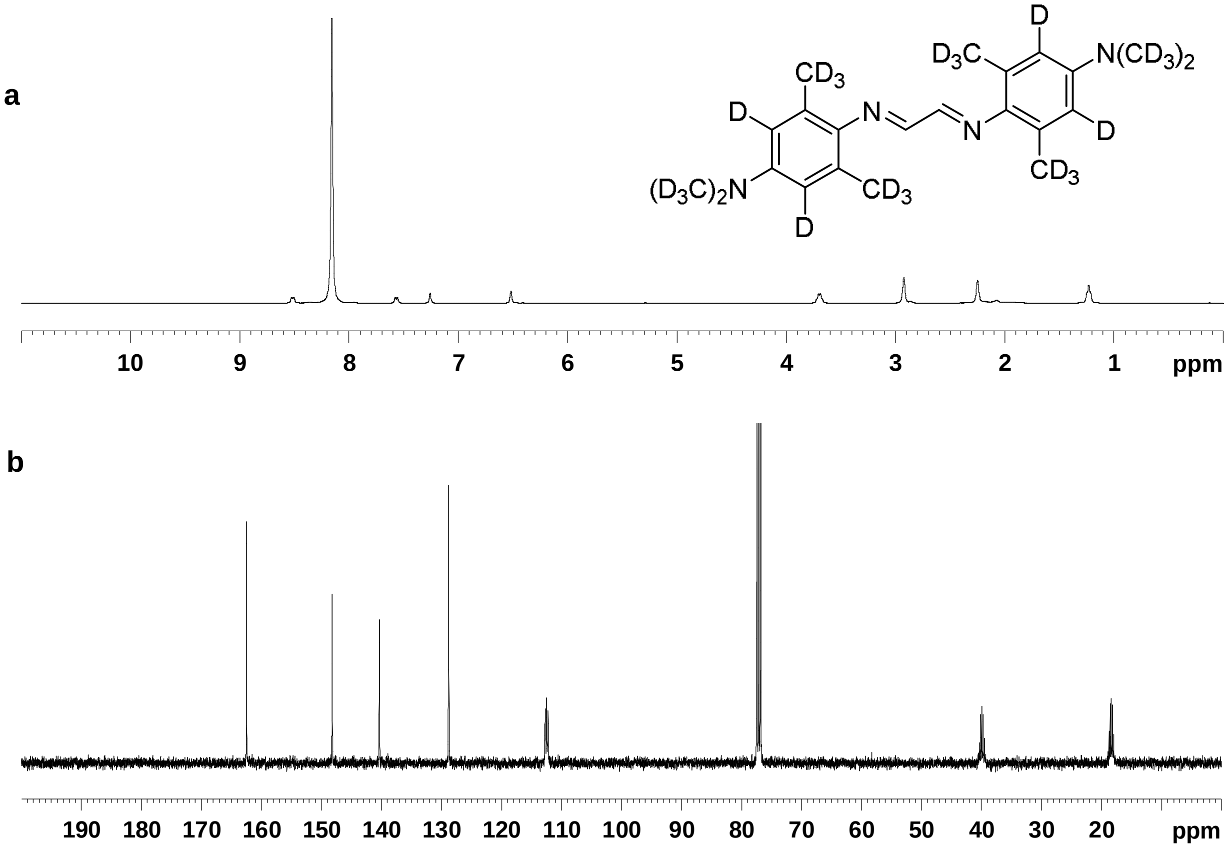


Supplementary Figure 124: NMR spectra of ***d*$_{28}$-S34**. **a** $^{1}$H NMR spectrum. **b** $^{13}$C NMR spectrum.

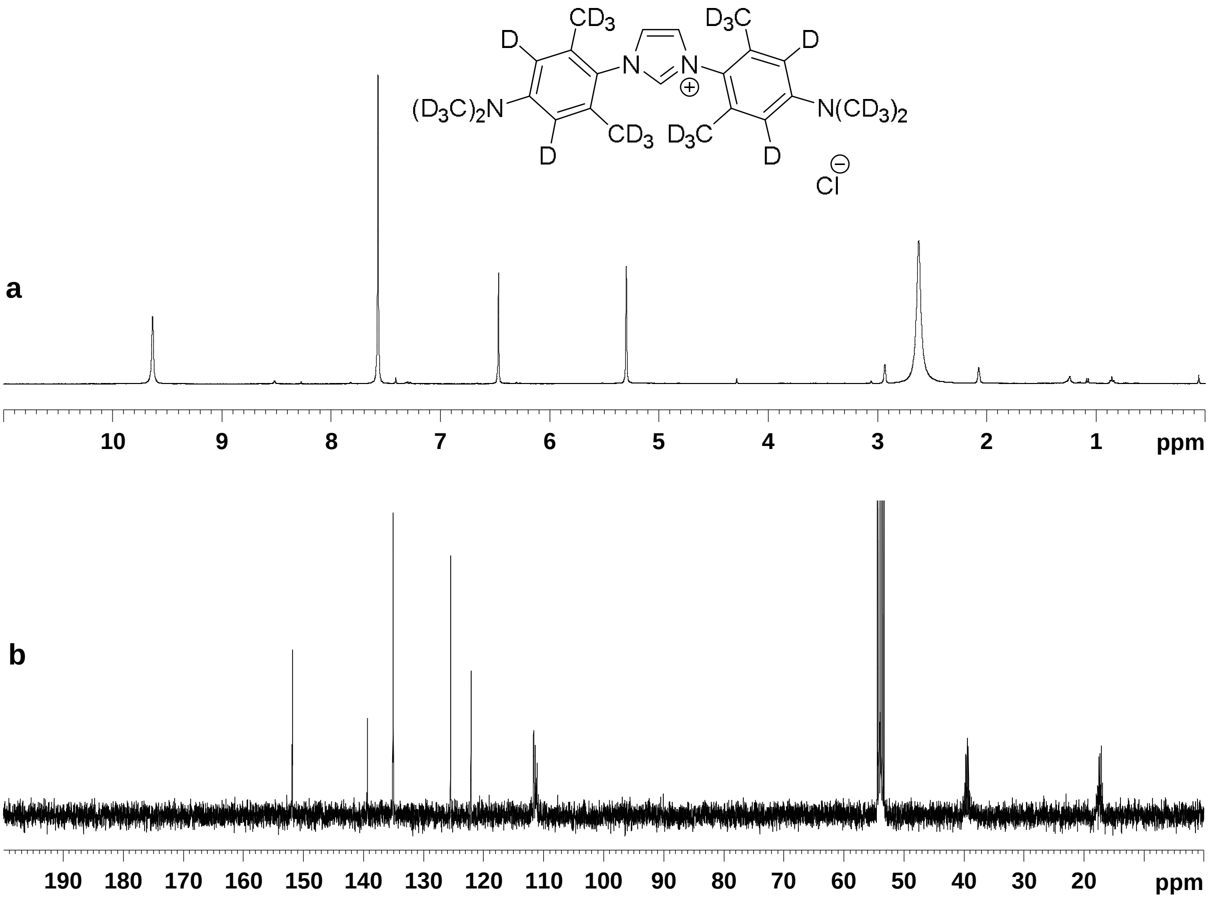


Supplementary Figure 125: NMR spectra of ***d*$_{28}$-S35**. **a** $^{1}$H NMR spectrum. **b** $^{13}$C NMR spectrum.

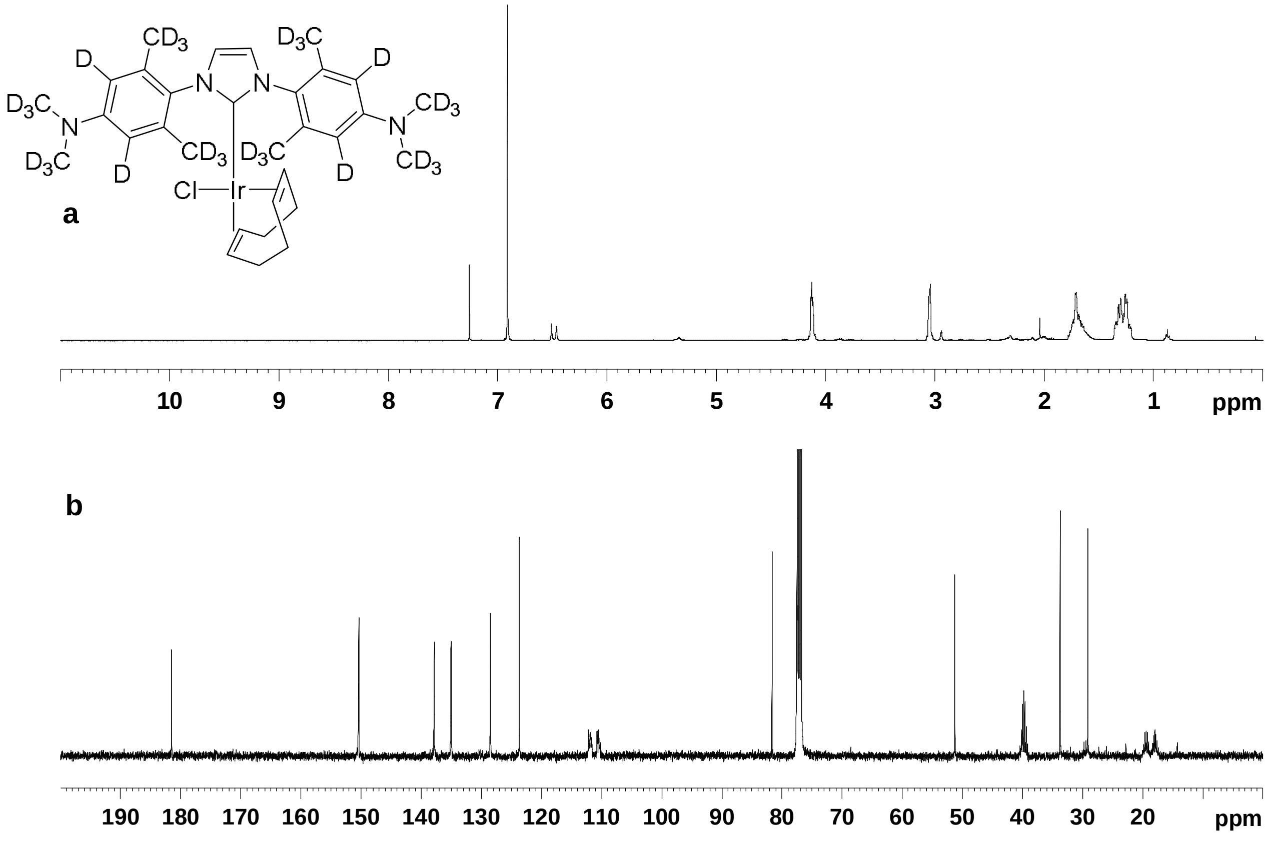


Supplementary Figure 126: NMR spectra of ***d*$_{28}$-18**. **a** $^{1}$H NMR spectrum. **b** $^{13}$C NMR spectrum.

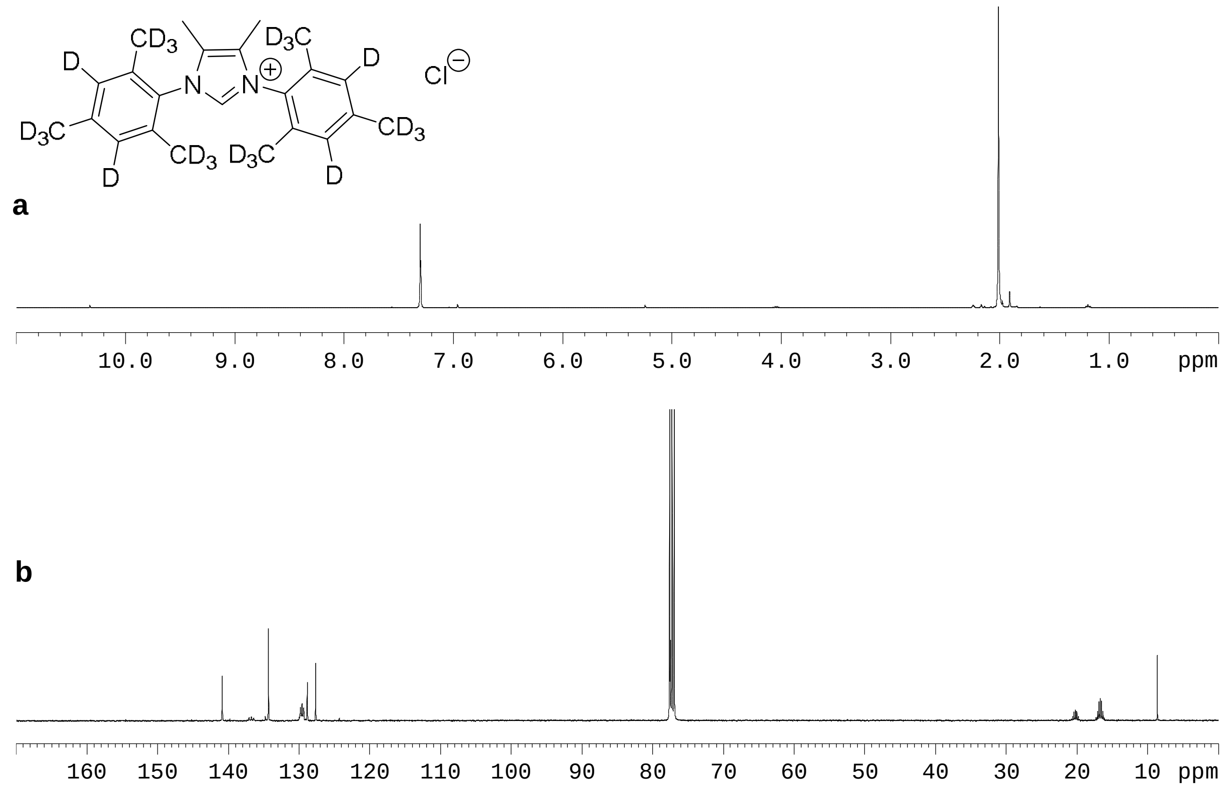


Supplementary Figure 127: NMR spectra of ***d*$_{22}$-S37**. **a** $^{1}$H NMR spectrum. **b** $^{13}$C NMR spectrum.

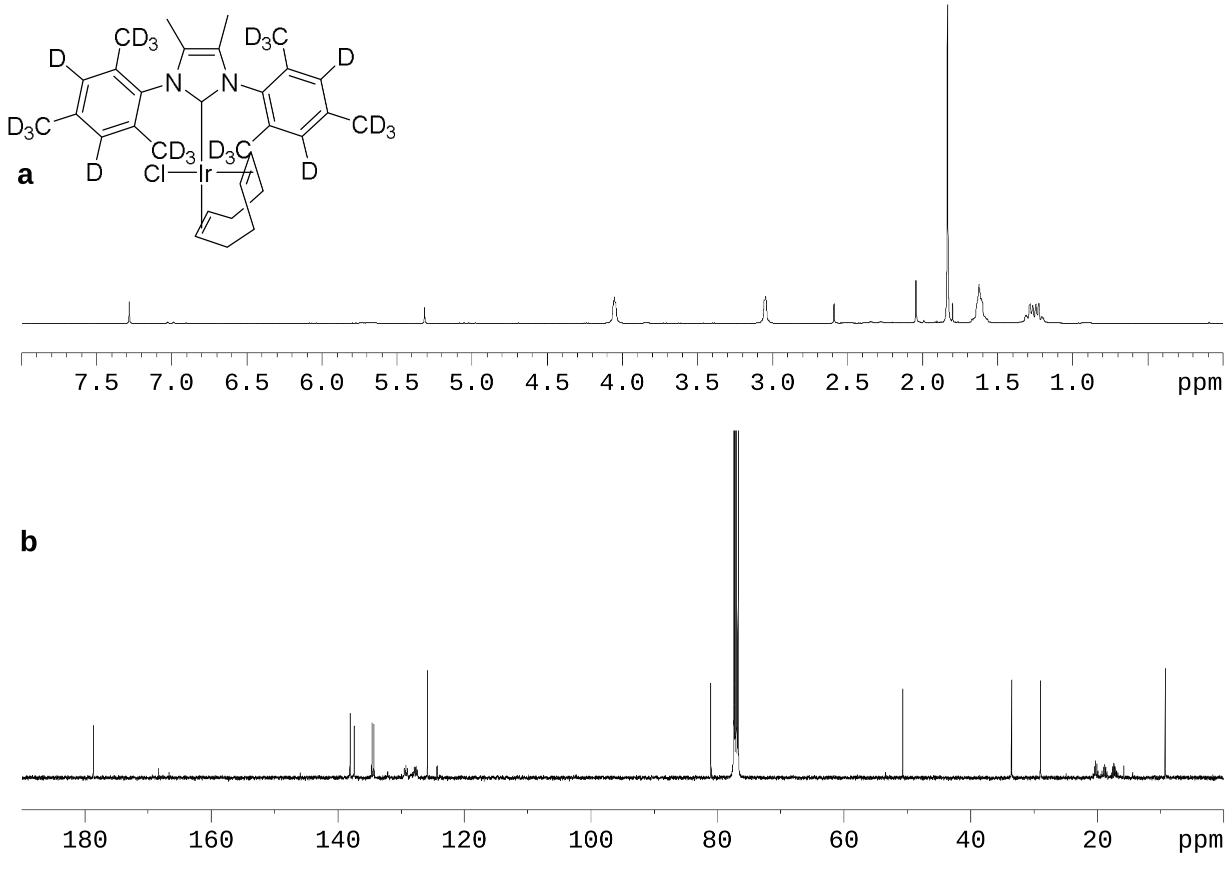


Supplementary Figure 128: NMR spectra of ***d*$_{22}$-19**. **a** $^{1}$H NMR spectrum. **b** $^{13}$C NMR spectrum.

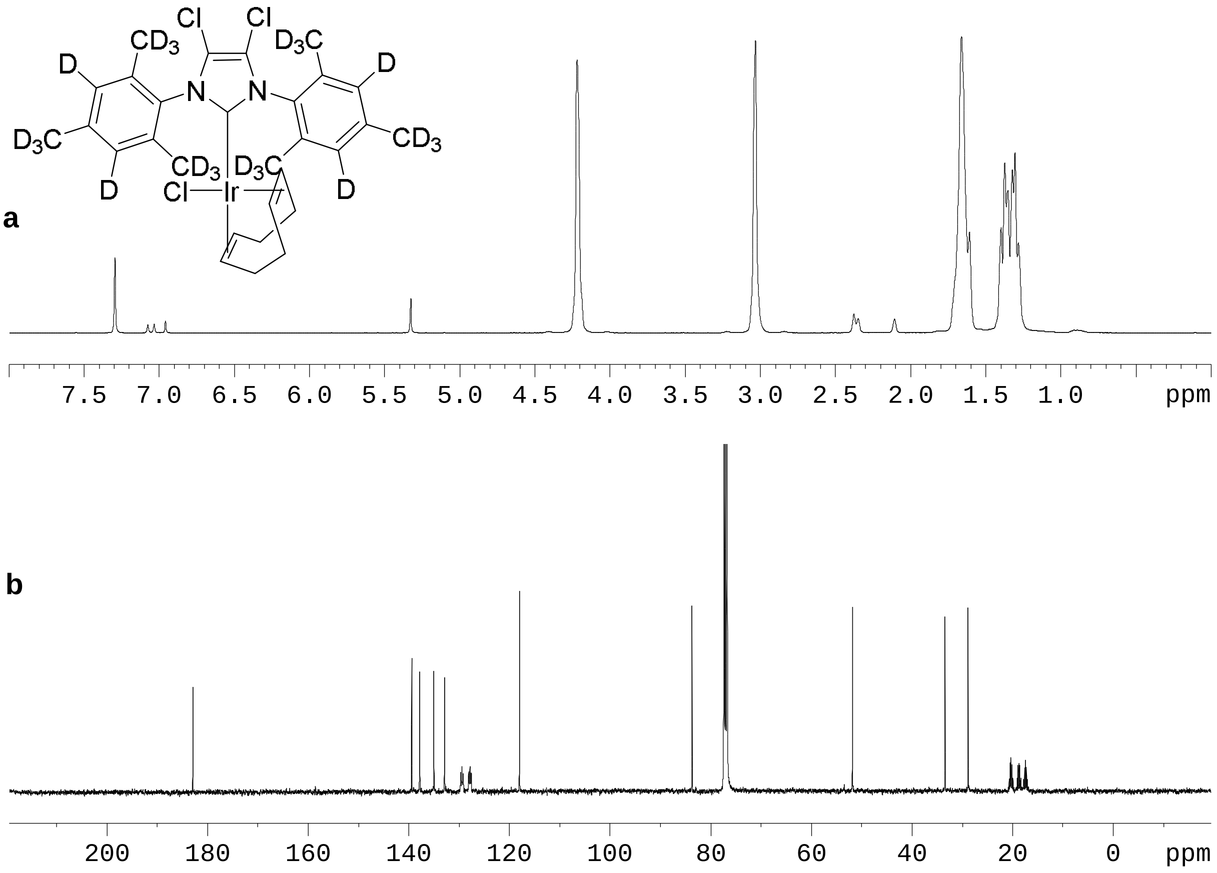


Supplementary Figure 129: NMR spectra of ***d*$_{22}$-21**. **a** $^{1}$H NMR spectrum. **b** $^{13}$C NMR spectrum.

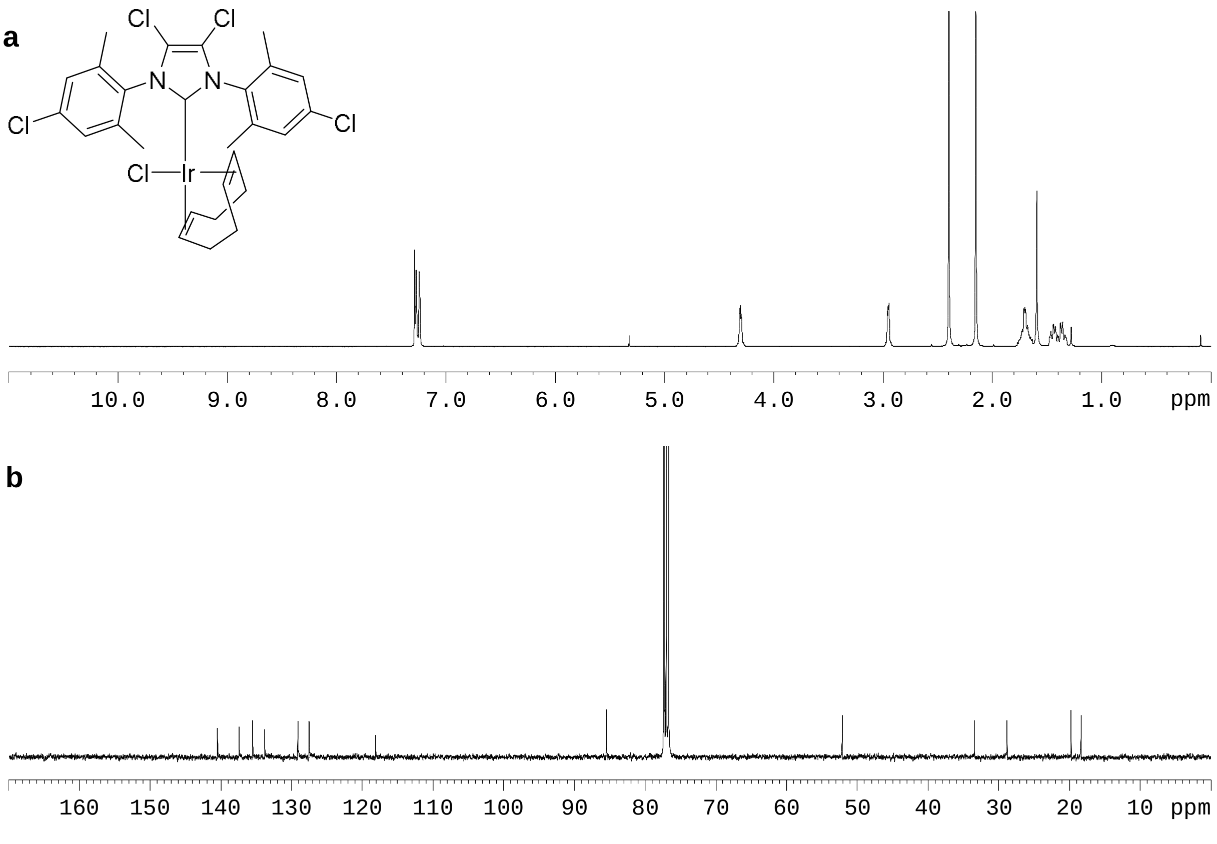


Supplementary Figure 130: NMR spectra of **23**. **a** $^{1}$H NMR spectrum. **b** $^{13}$C NMR spectrum.

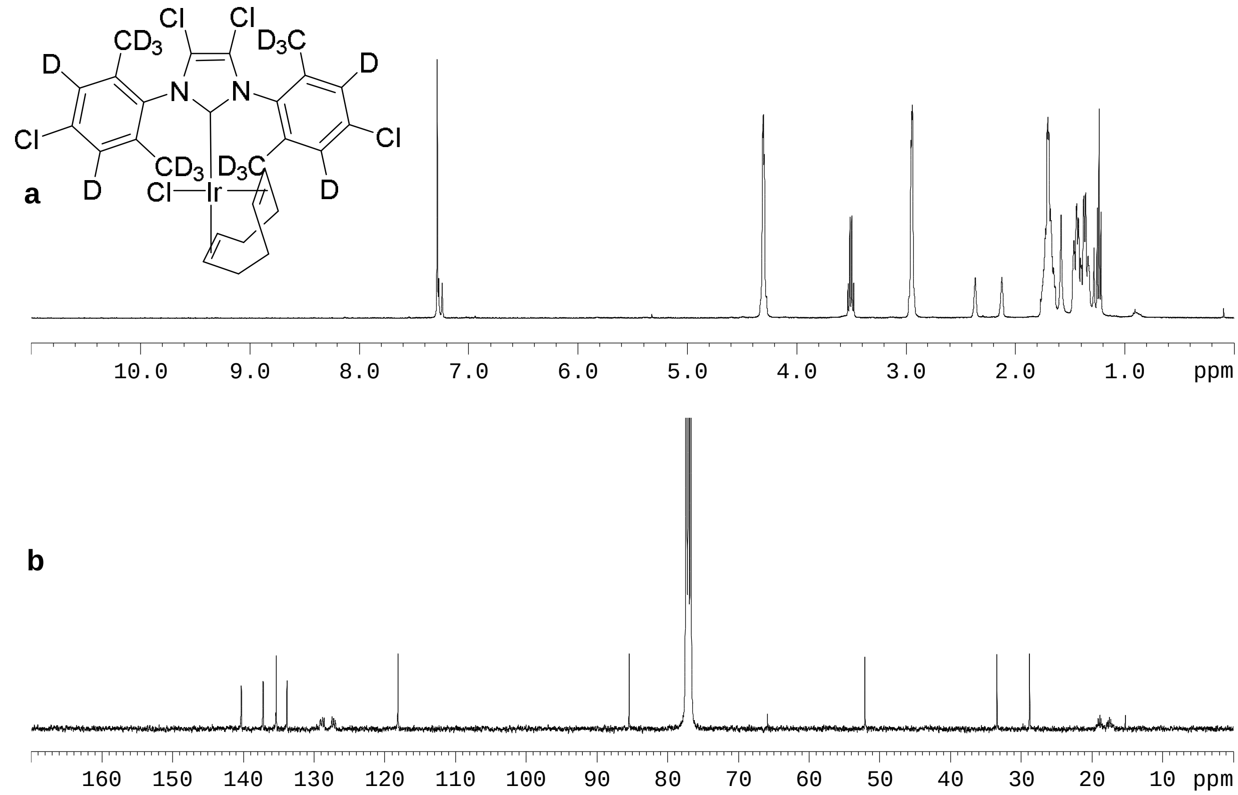


Supplementary Figure 131: NMR spectra of ***d*$_{16}$-23**. **a** $^1$H NMR spectrum. **b** $^{13}$C NMR spectrum.

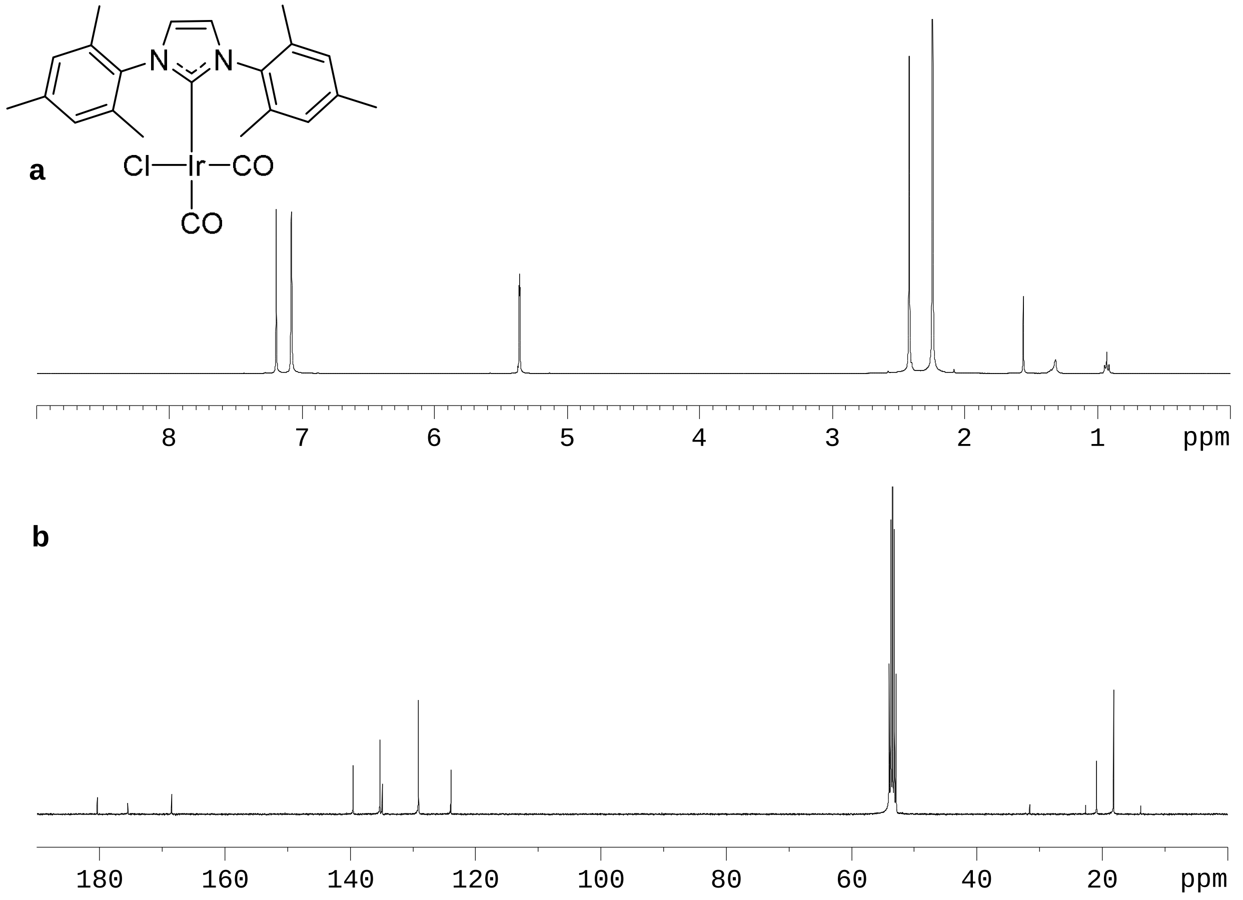


Supplementary Figure 132: NMR spectra of **S53**. **a** $^1$H NMR spectrum. **b** $^{13}$C NMR spectrum.

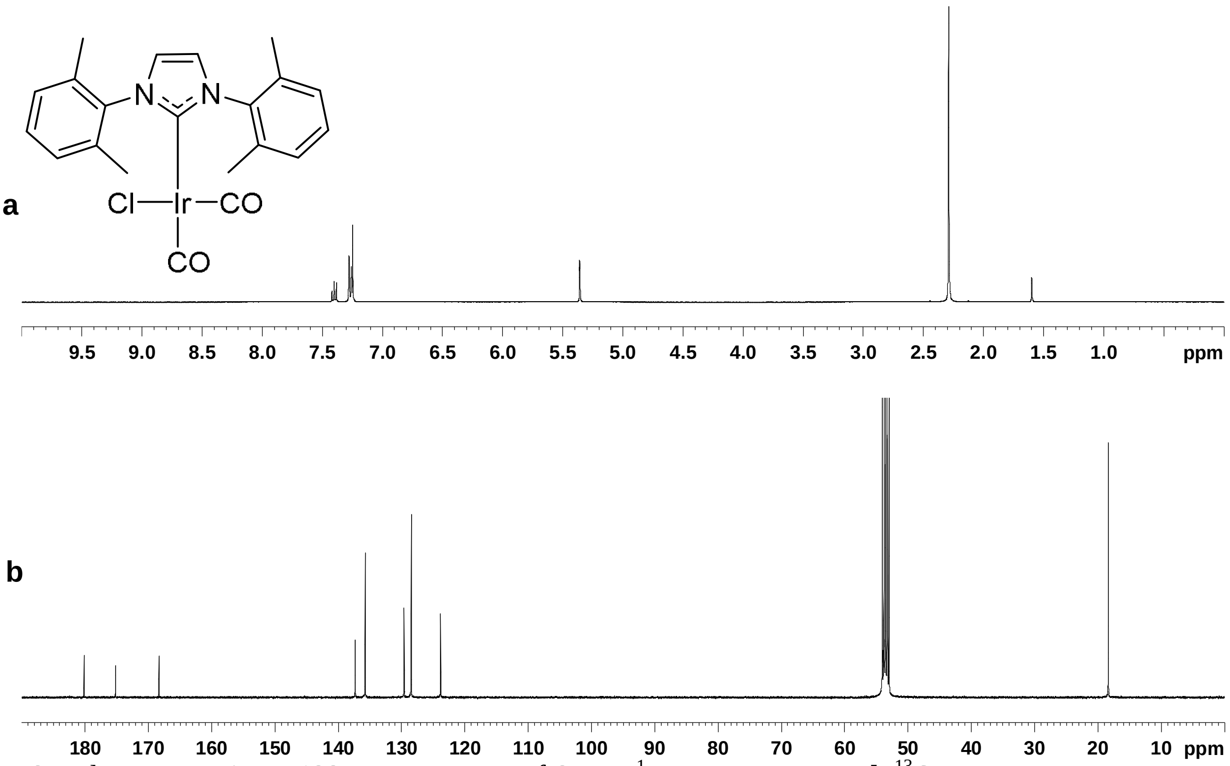


Supplementary Figure 133: NMR spectra of **S55**. **a** $^{1}H$ NMR spectrum. **b** $^{13}C$ NMR spectrum.

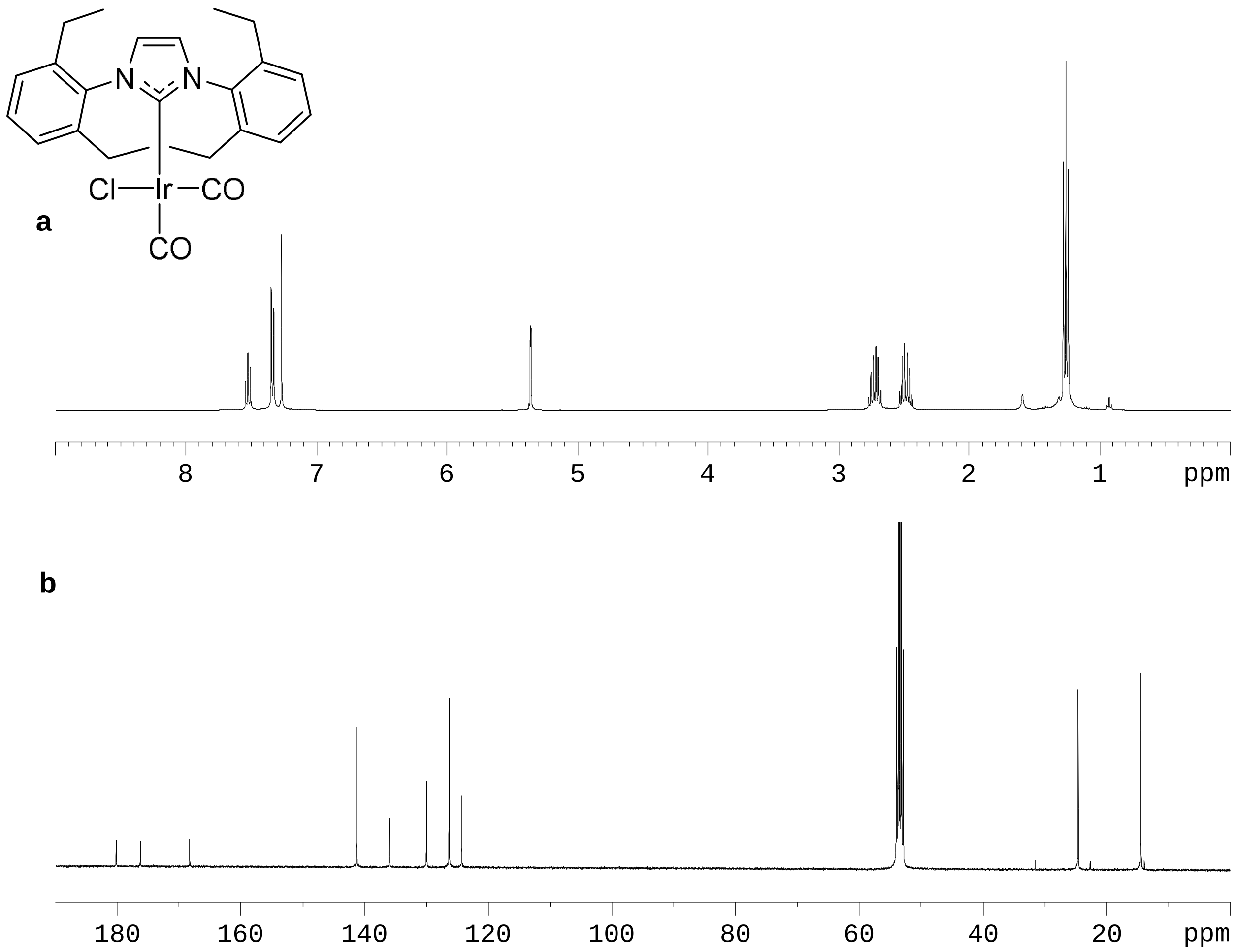


Supplementary Figure 134: NMR spectra of **S56**. **a** $^{1}H$ NMR spectrum. **b** $^{13}C$ NMR spectrum.

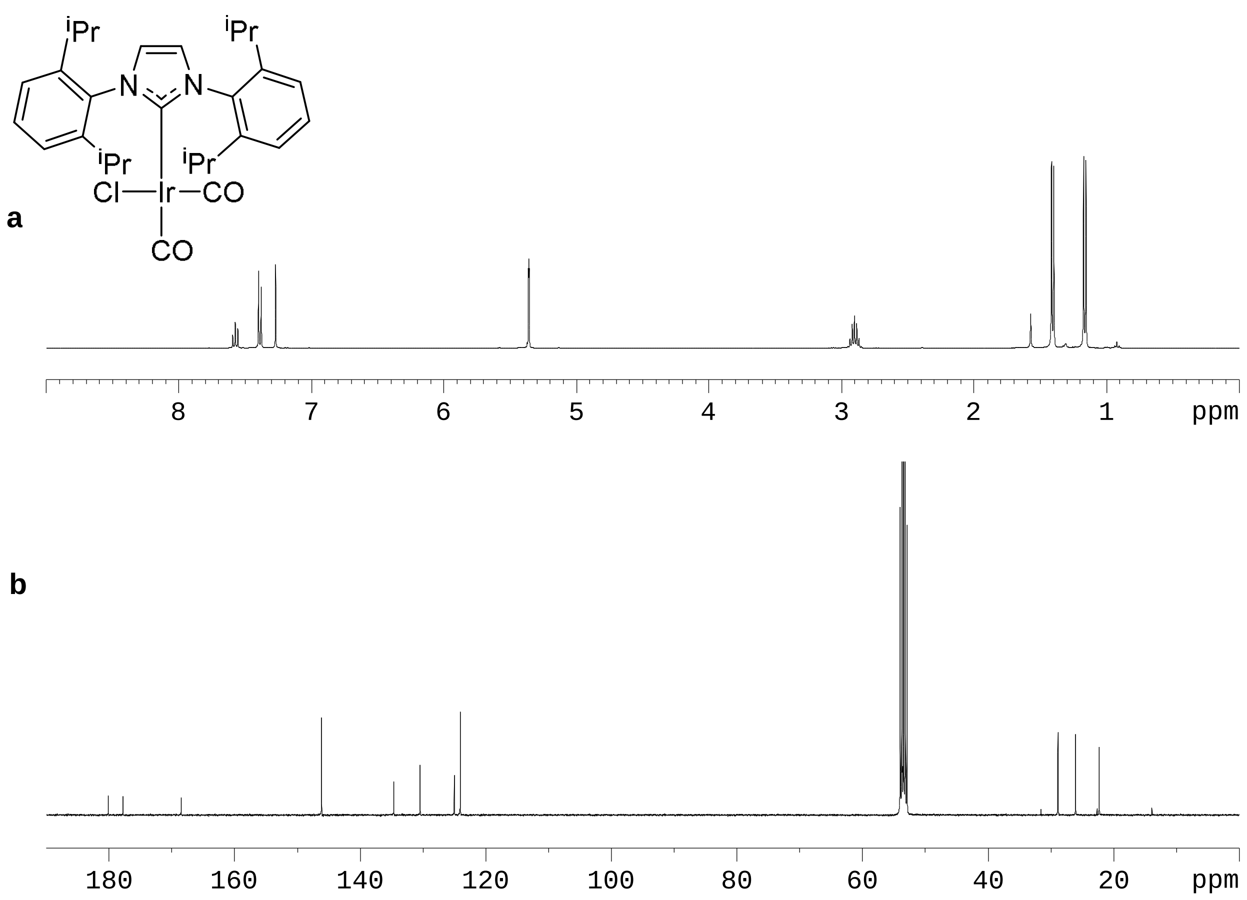


Supplementary Figure 135: NMR spectra of **S57**. **a** $^{1}$H NMR spectrum. **b** $^{13}$C NMR spectrum.

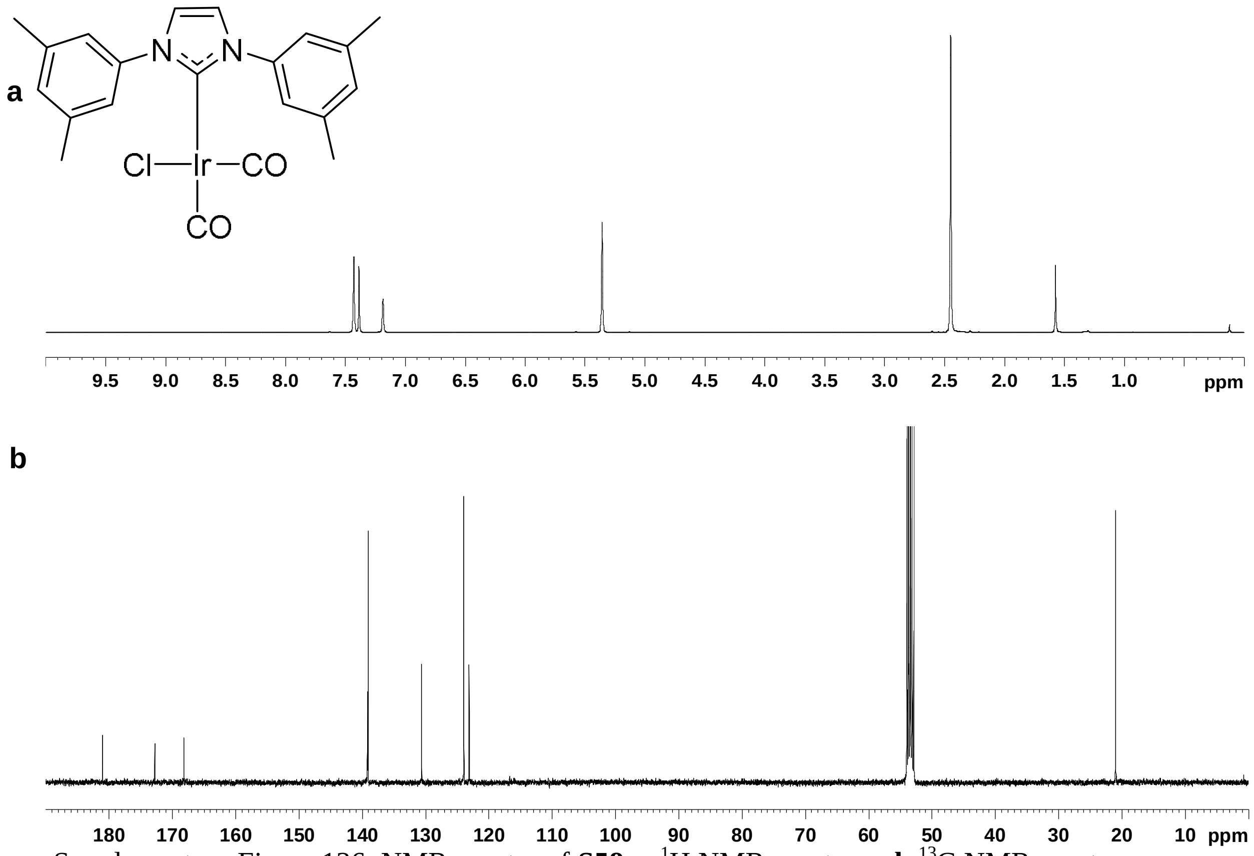


Supplementary Figure 136: NMR spectra of **S58**. **a** $^{1}$H NMR spectrum. **b** $^{13}$C NMR spectrum.

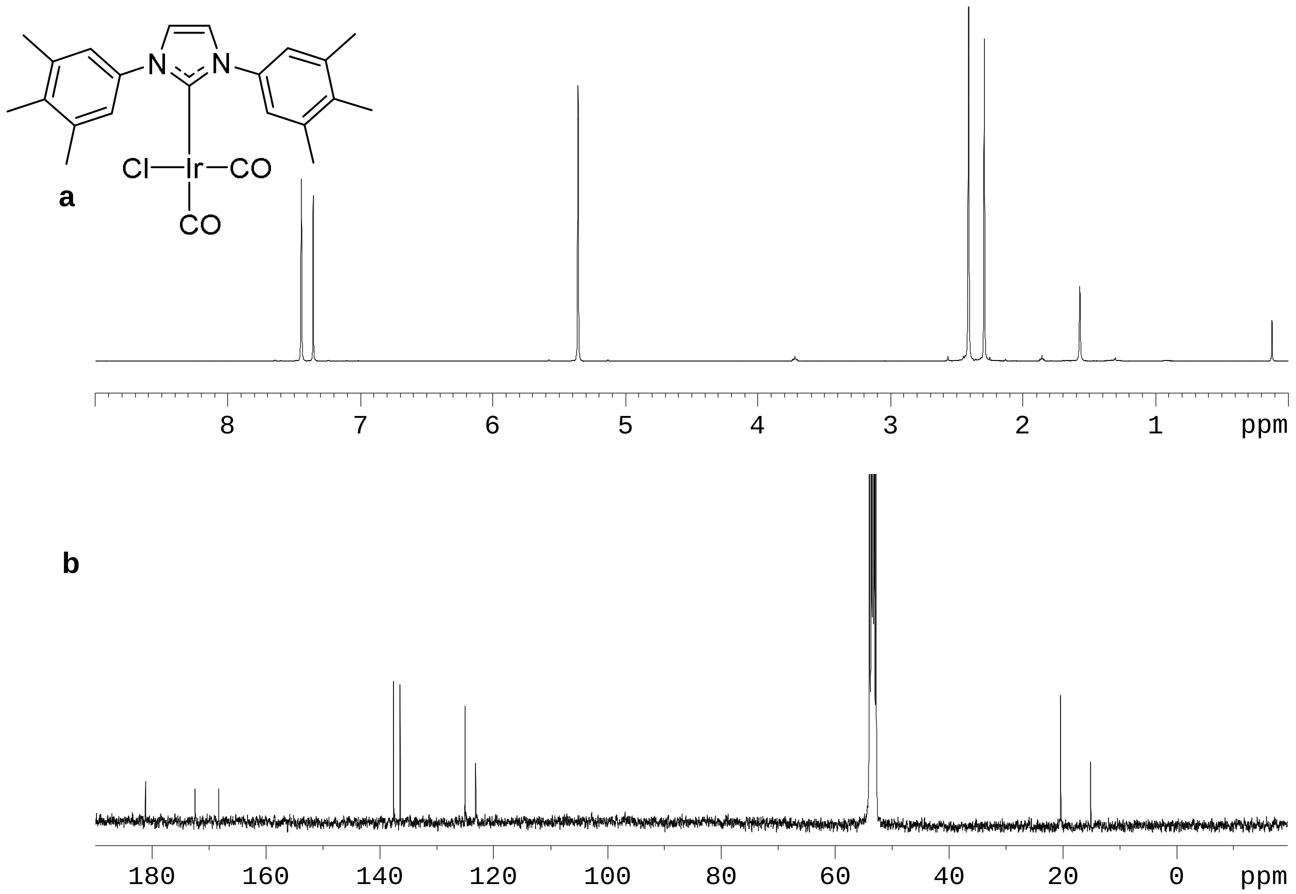


Supplementary Figure 137: NMR spectra of **S59**. **a** $^{1}H$ NMR spectrum. **b** $^{13}C$ NMR spectrum.

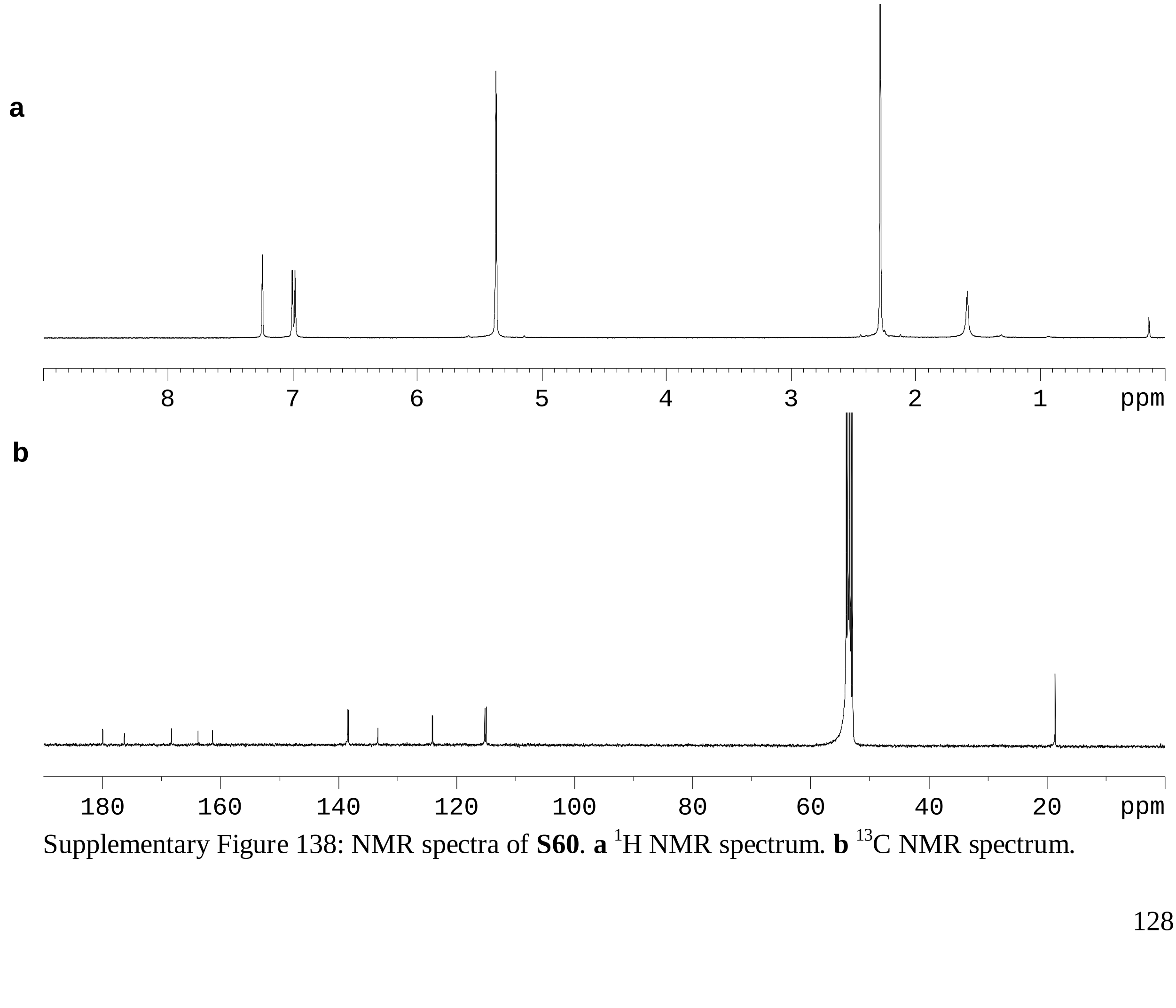


Supplementary Figure 138: NMR spectra of **S60**. **a** $^{1}H$ NMR spectrum. **b** $^{13}C$ NMR spectrum.

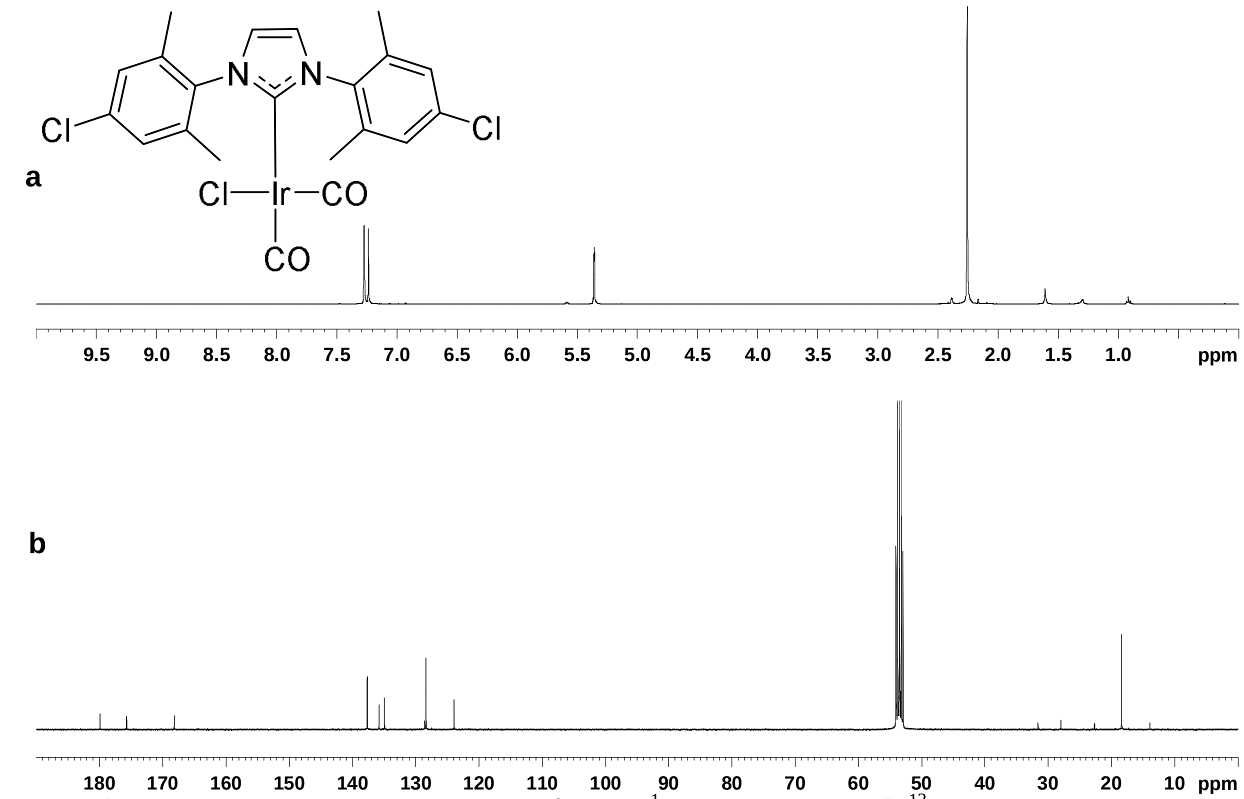


Supplementary Figure 139: NMR spectra of **S61**. **a** $^{1}H$ NMR spectrum. **b** $^{13}C$ NMR spectrum.

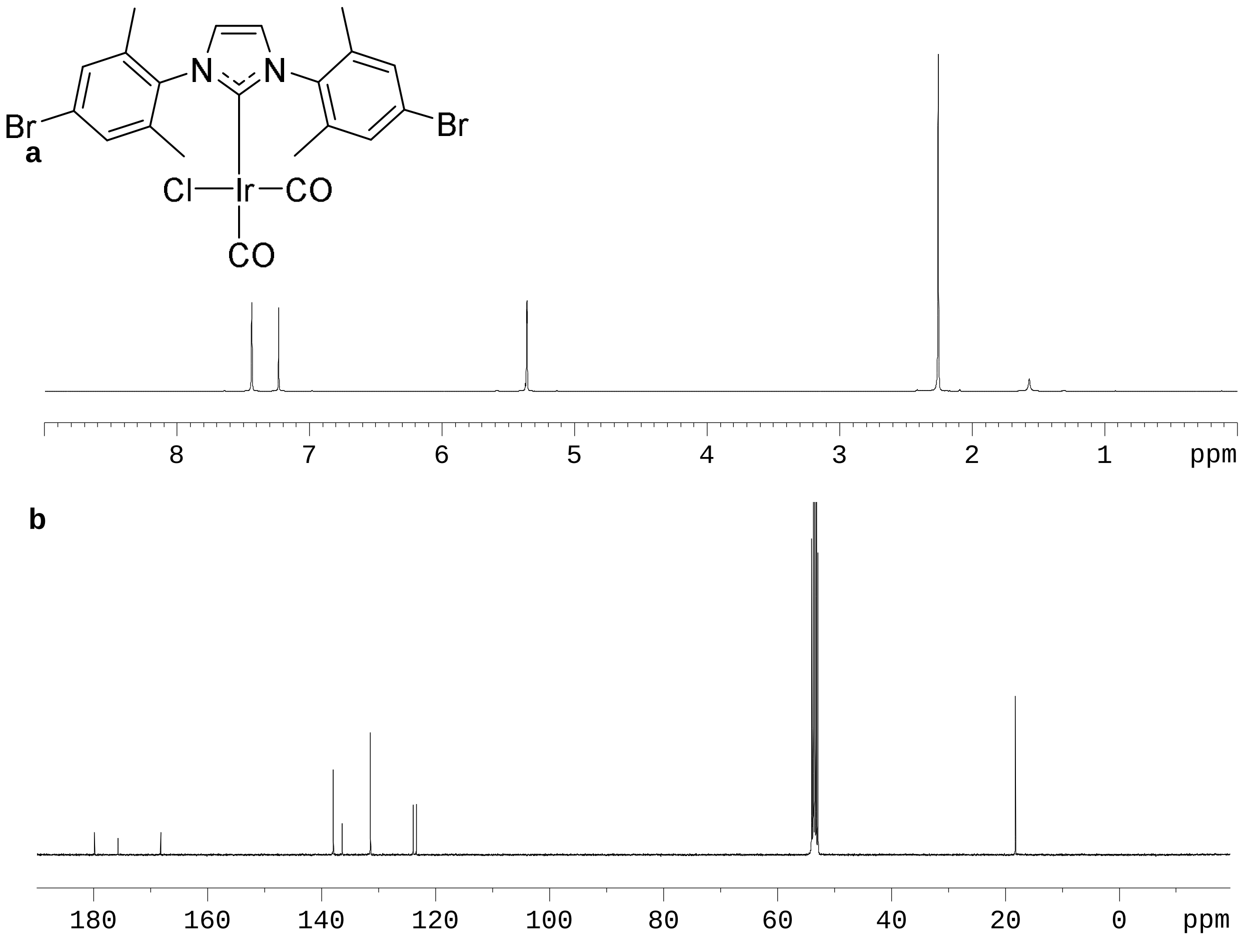


Supplementary Figure 140: NMR spectra of **S62**. **a** $^{1}H$ NMR spectrum. **b** $^{13}C$ NMR spectrum.

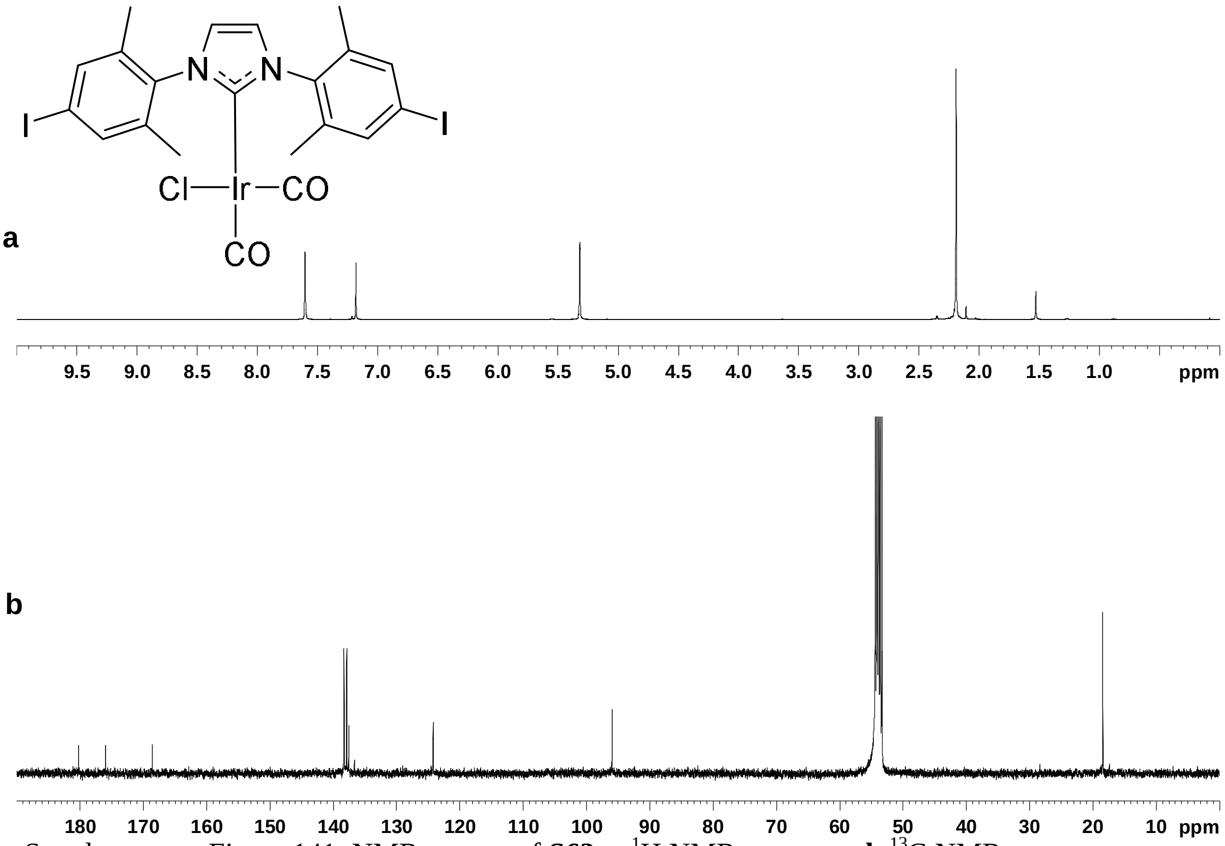


Supplementary Figure 141: NMR spectra of **S63**. **a** $^{1}$H NMR spectrum. **b** $^{13}$C NMR spectrum.

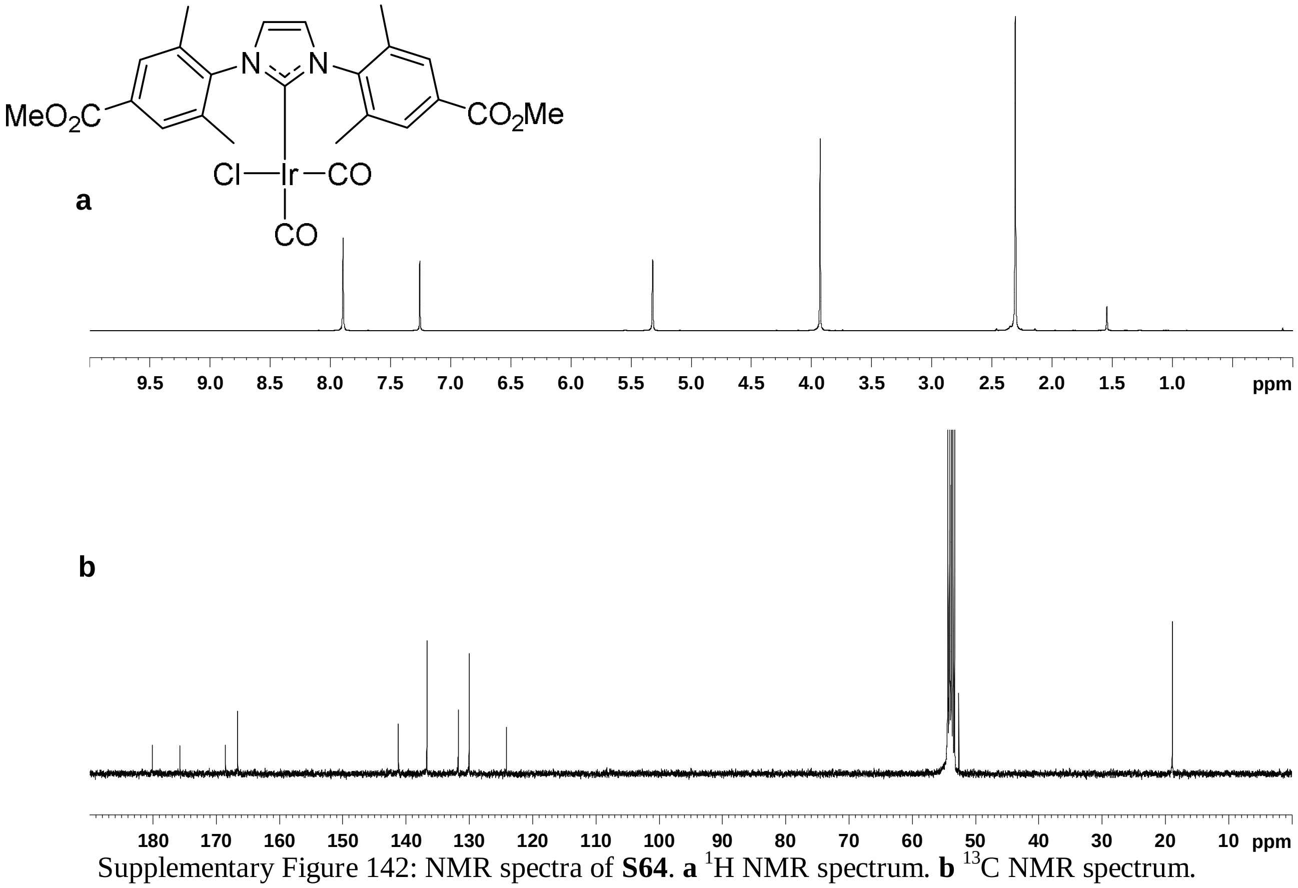


Supplementary Figure 142: NMR spectra of **S64**. **a** $^{1}$H NMR spectrum. **b** $^{13}$C NMR spectrum.

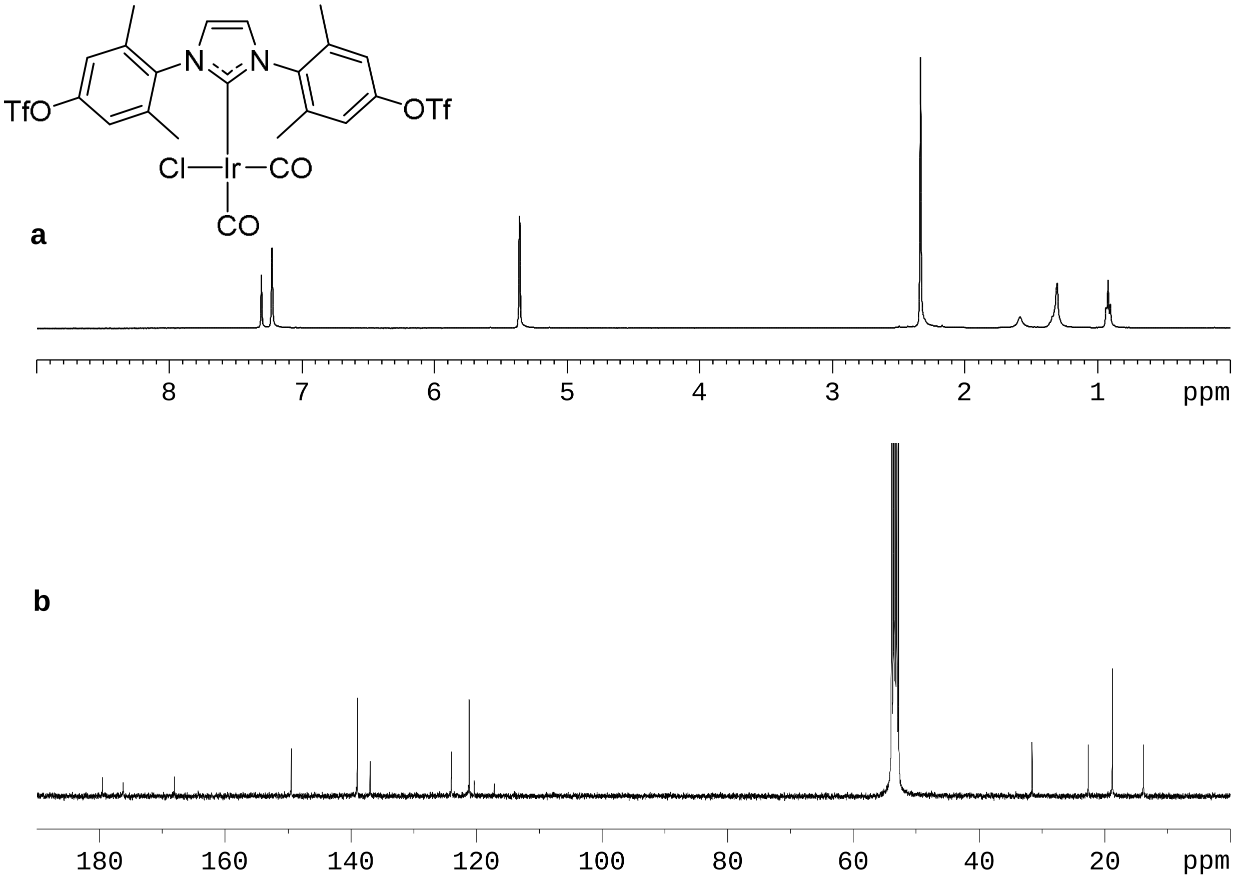


Supplementary Figure 143: NMR spectra of **S65**. **a** $^{1}$H NMR spectrum. **b** $^{13}$C NMR spectrum.

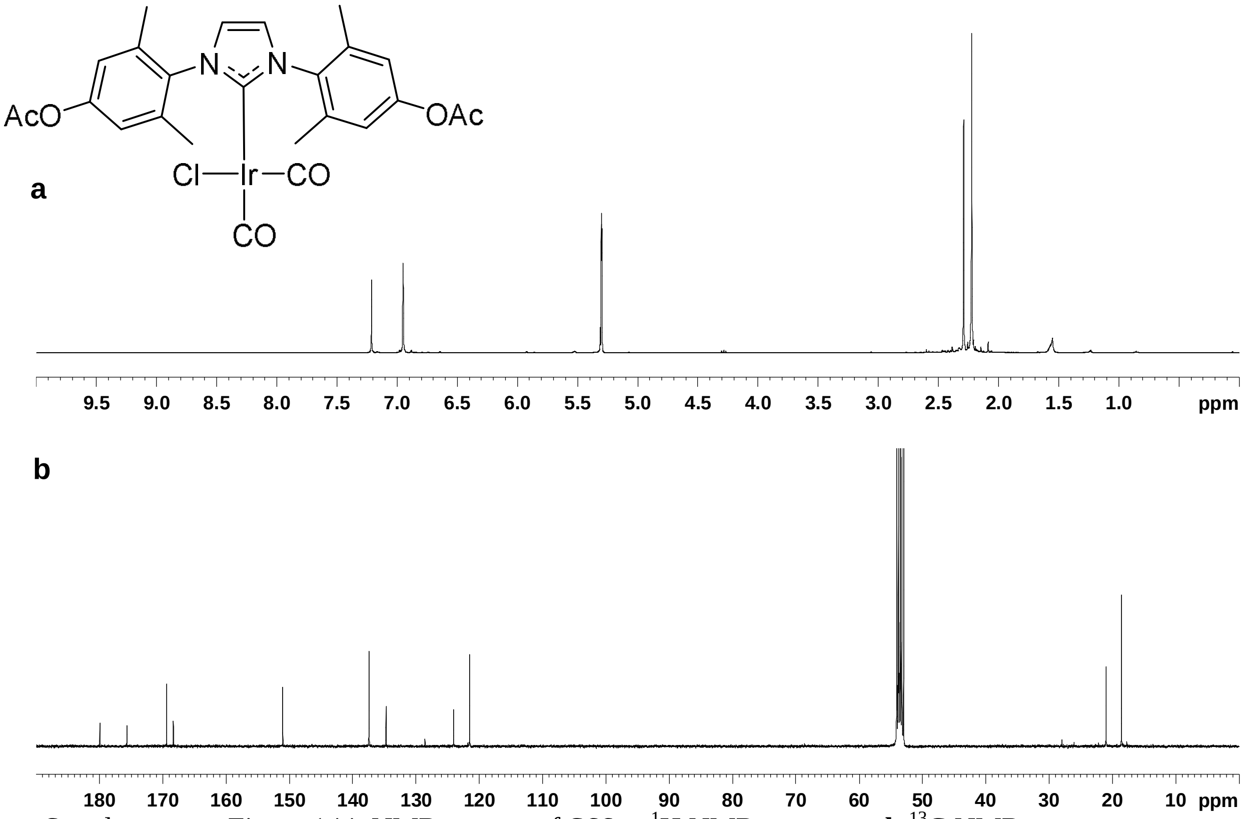


Supplementary Figure 144: NMR spectra of **S66**. **a** $^{1}$H NMR spectrum. **b** $^{13}$C NMR spectrum.

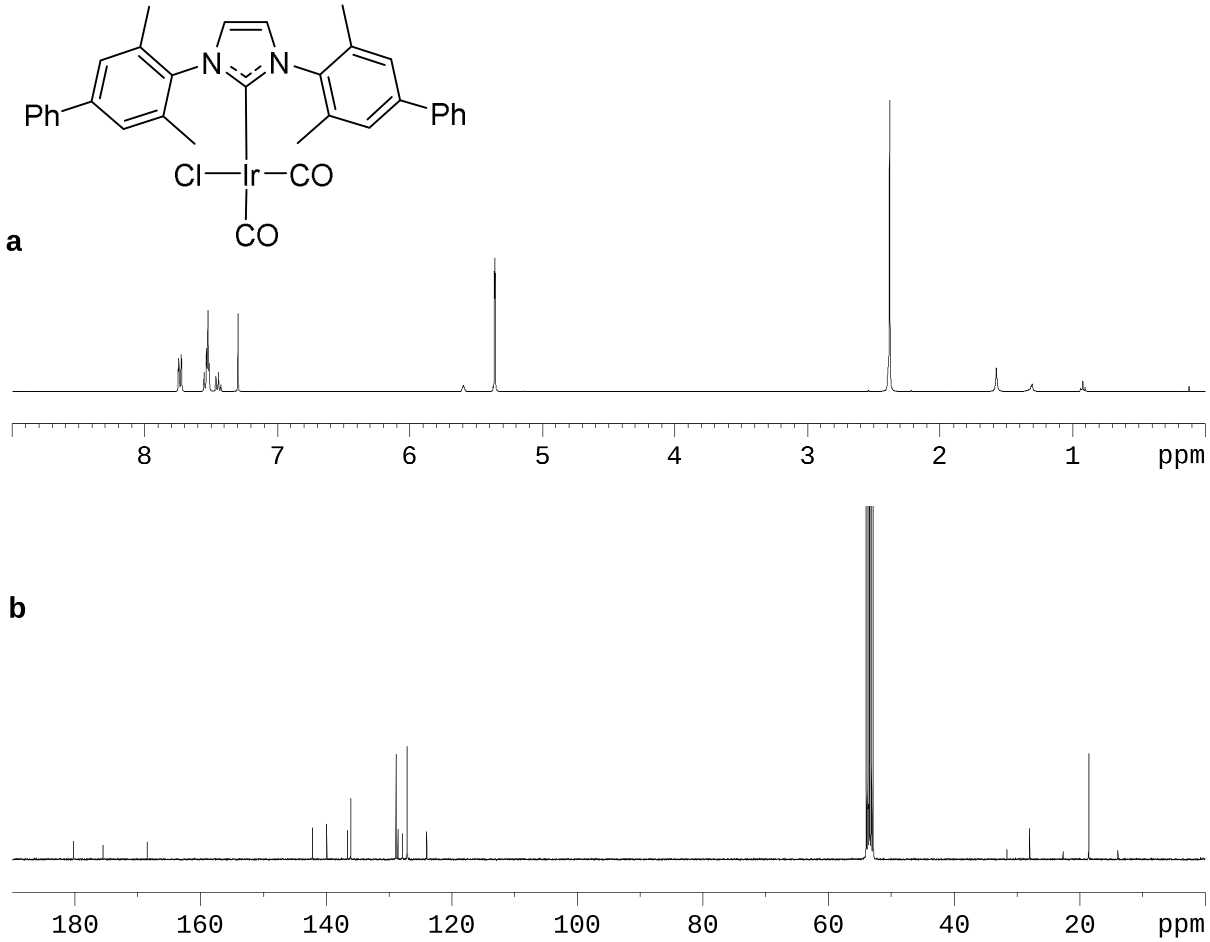


Supplementary Figure 145: NMR spectra of **S67**. **a** $^{1}H$ NMR spectrum. **b** $^{13}C$ NMR spectrum.

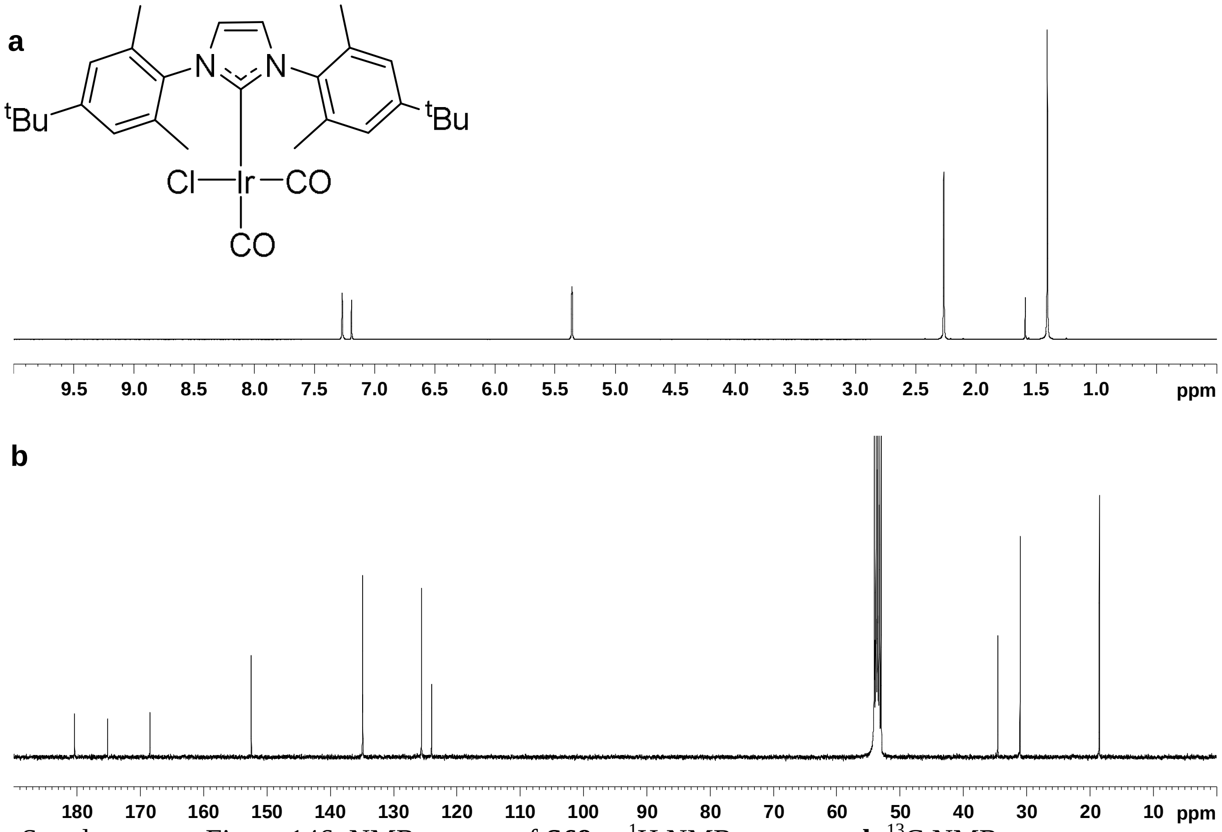


Supplementary Figure 146: NMR spectra of **S68**. **a** $^{1}H$ NMR spectrum. **b** $^{13}C$ NMR spectrum.

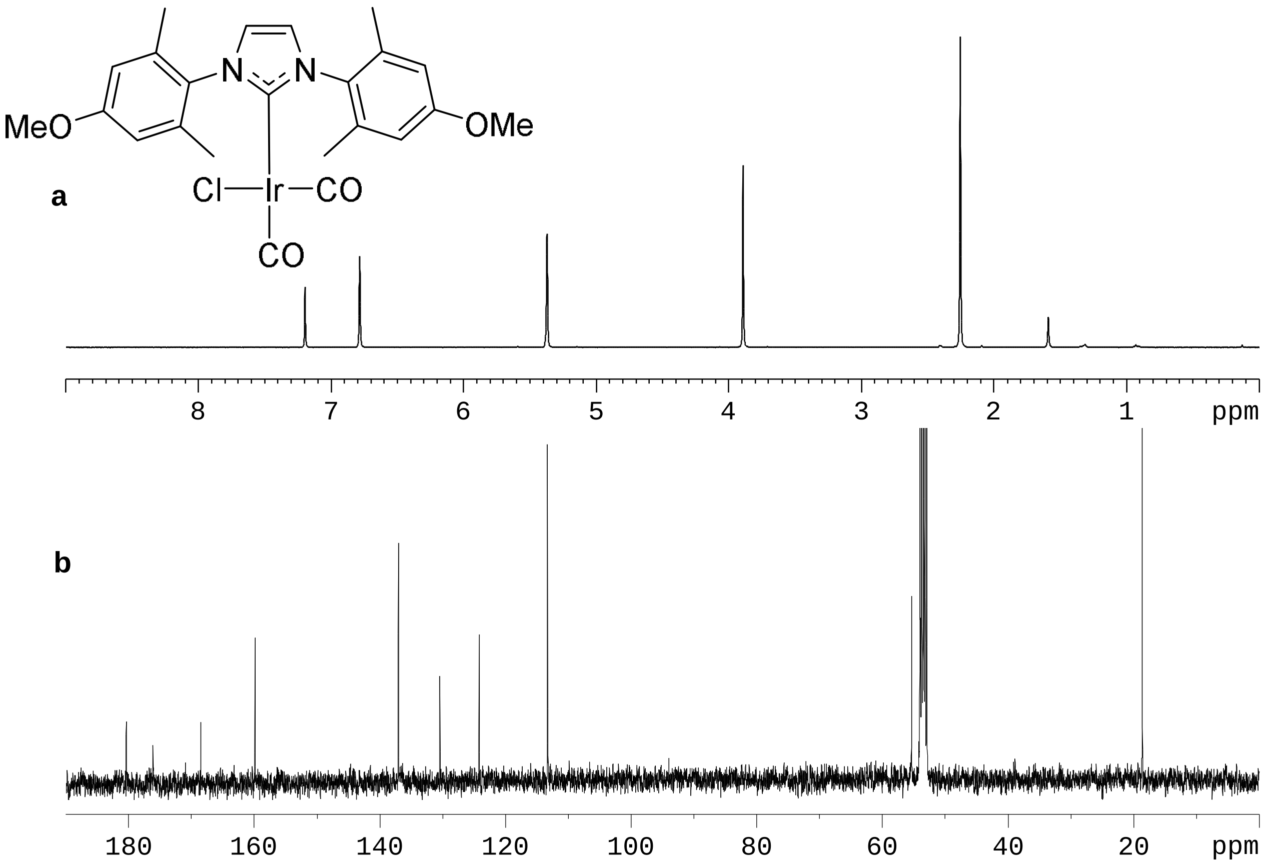


Supplementary Figure 147: NMR spectra of **S69**. **a** $^{1}H$ NMR spectrum. **b** $^{13}C$ NMR spectrum.

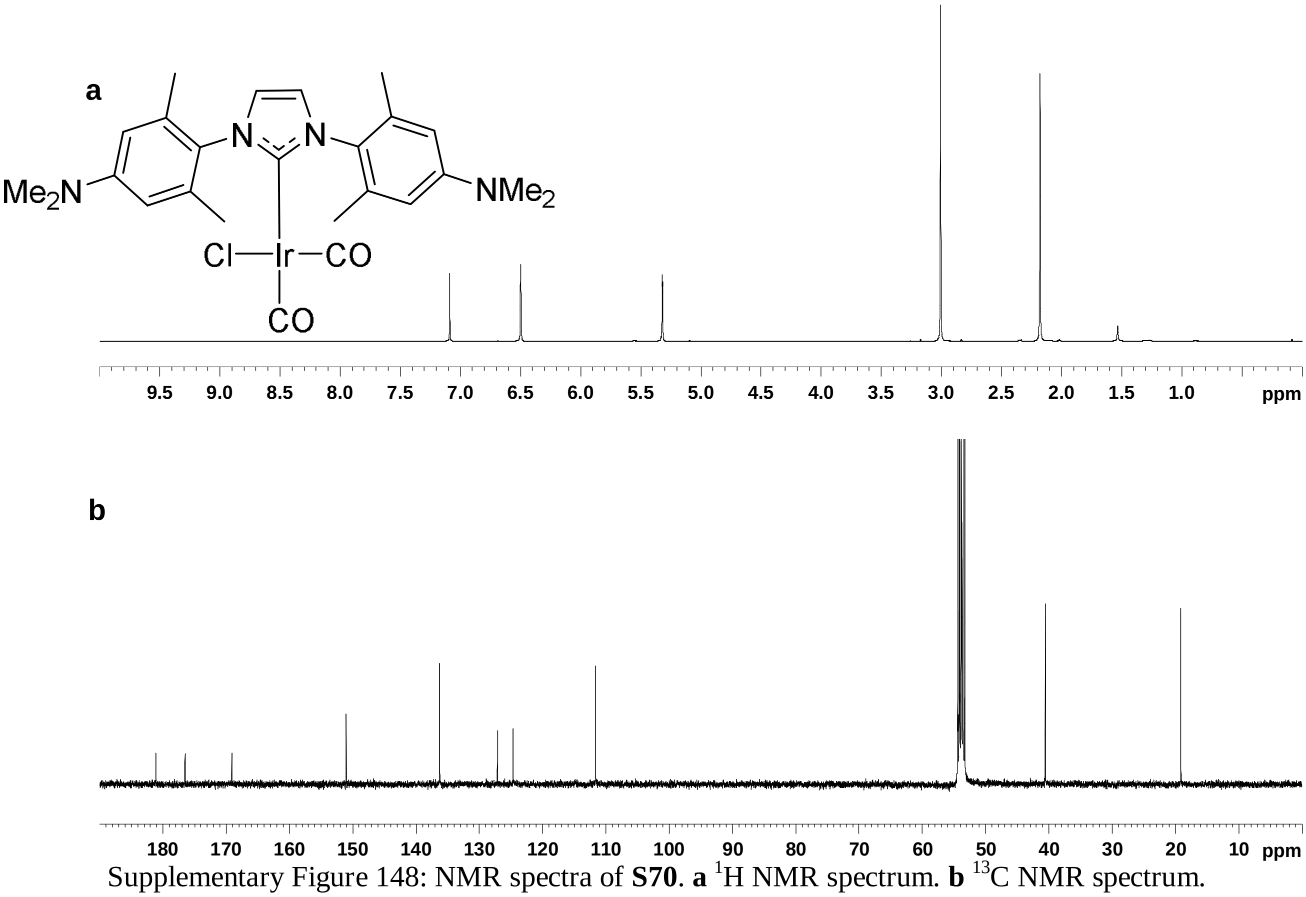


Supplementary Figure 148: NMR spectra of **S70**. **a** $^{1}H$ NMR spectrum. **b** $^{13}C$ NMR spectrum.

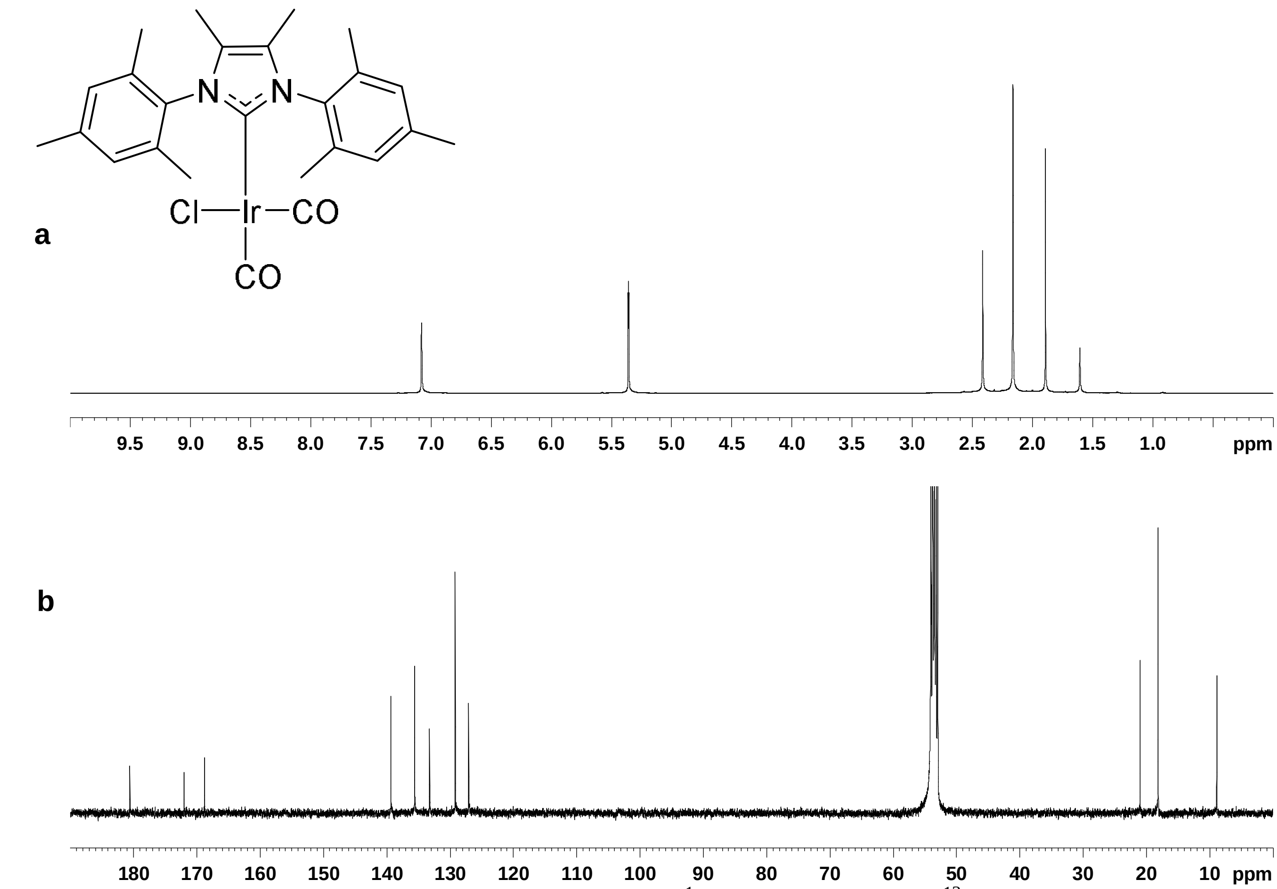


Supplementary Figure 149: NMR spectra of **S71**. **a** $^{1}$H NMR spectrum. **b** $^{13}$C NMR spectrum.

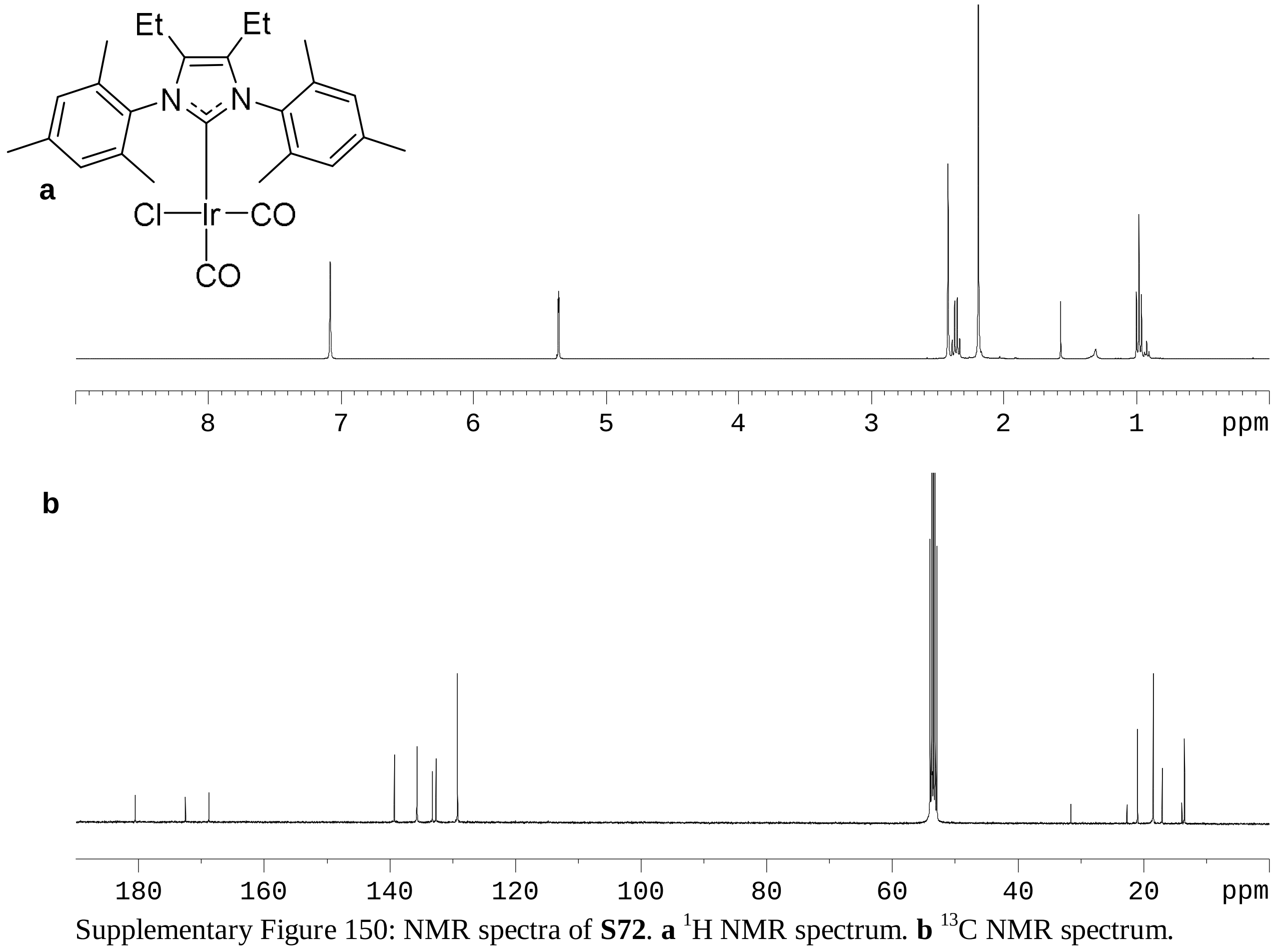


Supplementary Figure 150: NMR spectra of **S72**. **a** $^{1}$H NMR spectrum. **b** $^{13}$C NMR spectrum.

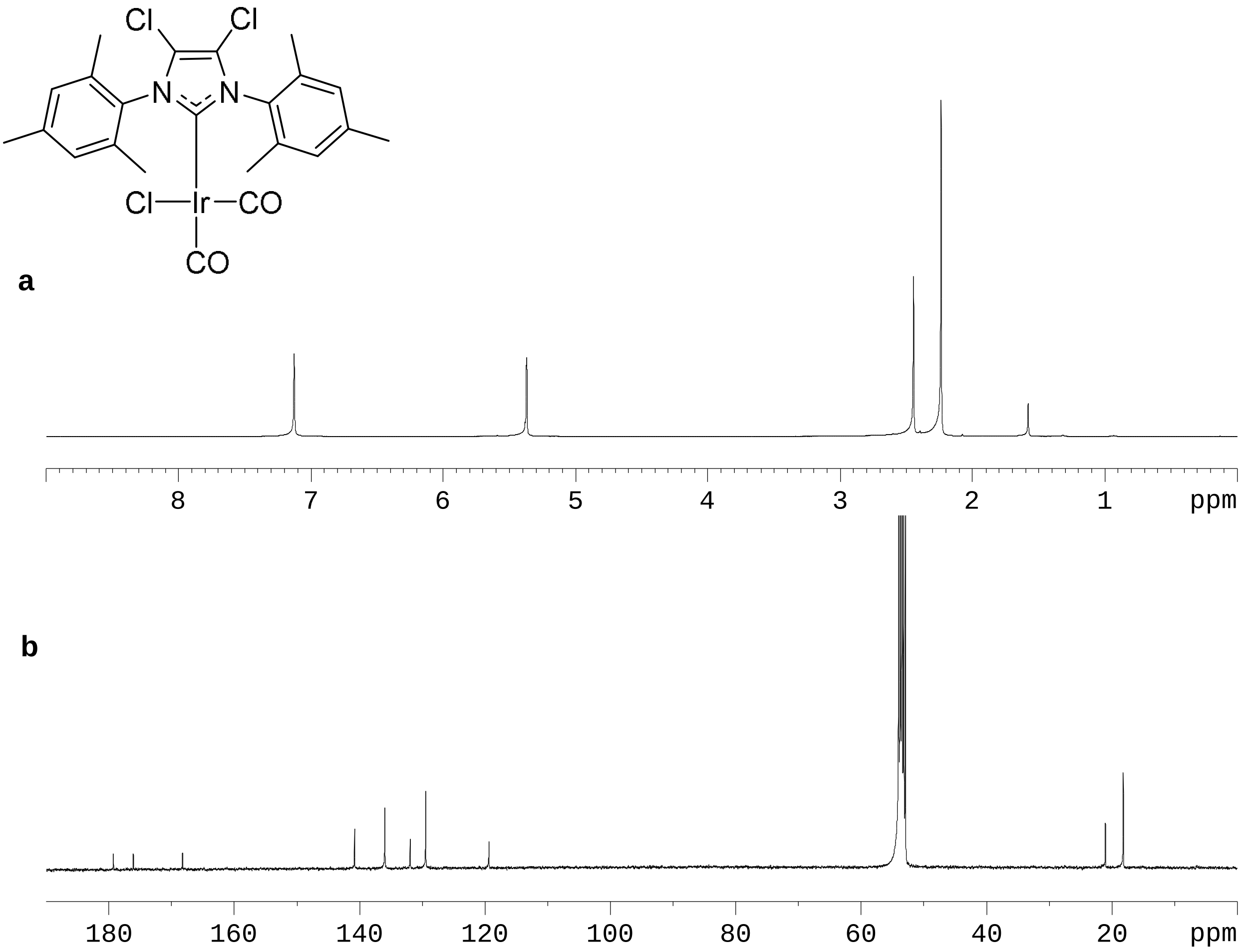


Supplementary Figure 151: NMR spectra of **S73**. **a** $^{1}$H NMR spectrum. **b** $^{13}$C NMR spectrum.

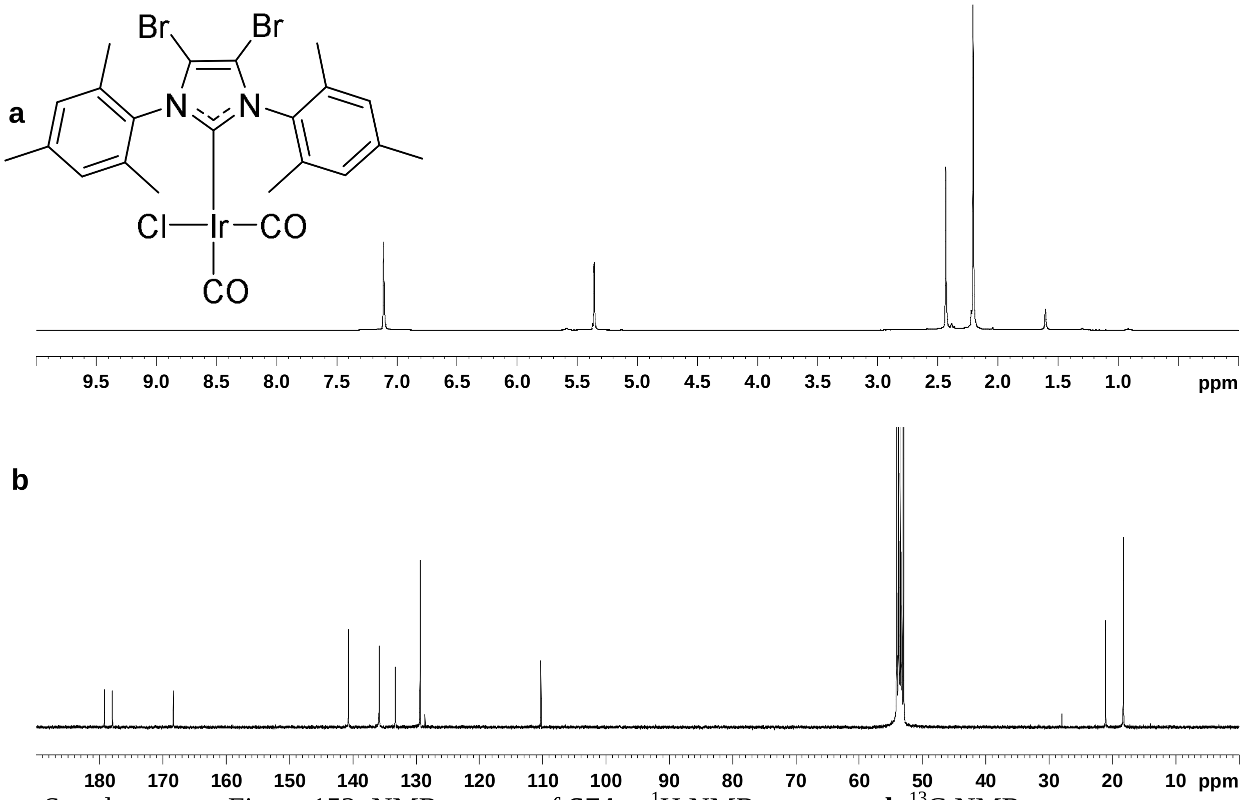


Supplementary Figure 152: NMR spectra of **S74**. **a** $^{1}$H NMR spectrum. **b** $^{13}$C NMR spectrum.

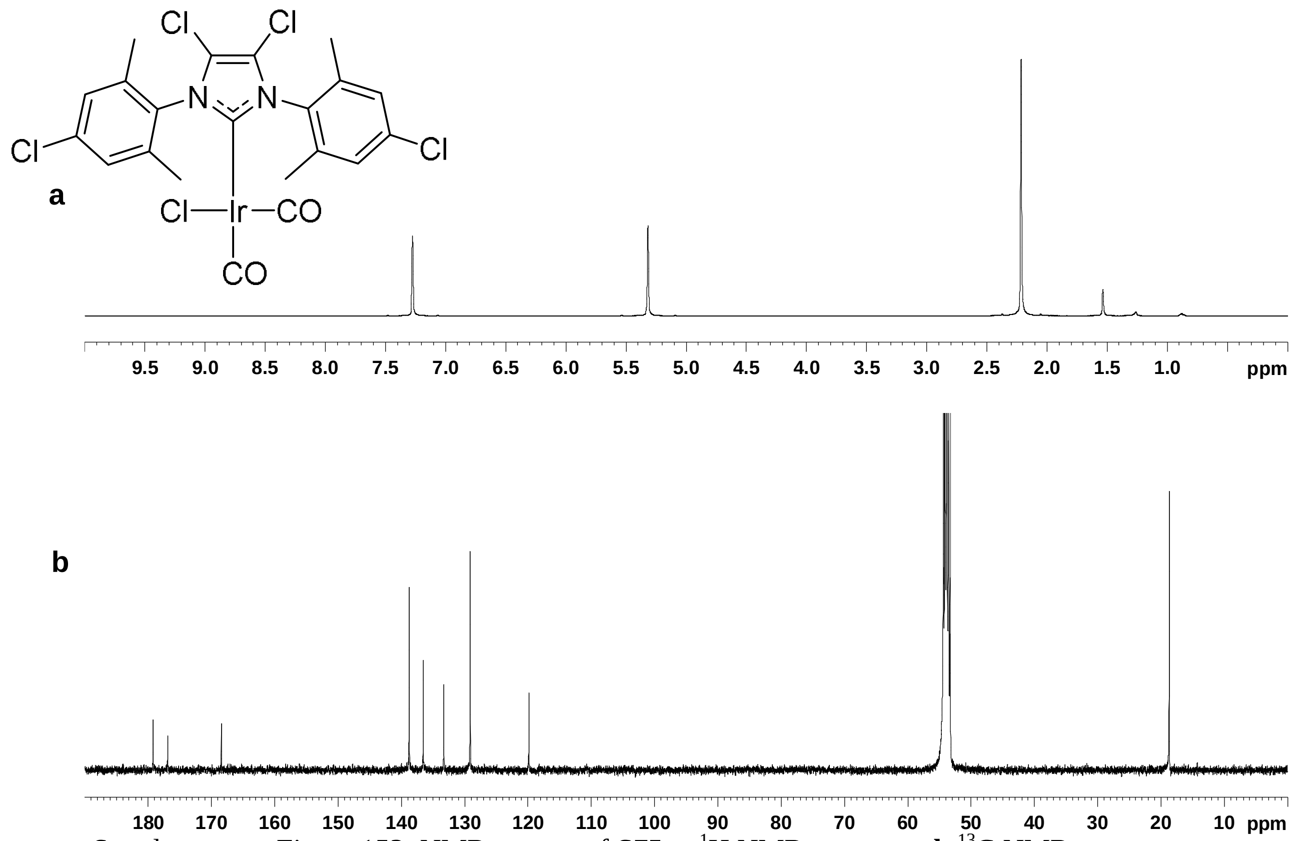

Supplementary Figure 153: NMR spectra of **S75**. **a** $^{1}$H NMR spectrum. **b** $^{13}$C NMR spectrum.

## Supplementary References


1. Rayner P. J., Burns M. J., Olaru A. M., Norcott P., Fekete M., Green G. G. R., *et al.*, Delivering strong 1H nuclear hyperpolarization levels and long magnetic lifetimes through signal amplification by reversible exchange, *Proc Natl Acad Sci USA,* **114,** E3188-E3194 (2017).

2. Vazquez-Serrano L. D., Owens B. T., Buriak J. M., The search for new hydrogenation catalyst motifs based on N-heterocyclic carbene ligands, *Inorg Chim Acta,* **359,** 2786-2797 (2006).

3. Savka R., Plenio H., Facile synthesis of [(NHC)MX(cod)] and [(NHC)MCl(CO)2] (M = Rh, Ir; X = Cl, I) complexes, *Dalton Trans,* **44,** 891-893 (2015).

4. Fekete M., Bayfield O., Duckett S. B., Hart S., Mewis R. E., Pridmore N., *et al.*, Iridium(III) Hydrido N-Heterocyclic Carbene–Phosphine Complexes as Catalysts in Magnetization Transfer Reactions, *Inorg Chem,* **52,** 13453-13461 (2013).

5. Bernardi T., Badel S., Mayer P., Groelly J., de Frémont P., Jacques B., *et al.*, High-Throughput Screening of Metal-N-Heterocyclic Carbene Complexes against Biofilm Formation by Pathogenic Bacteria, *ChemMedChem,* **9,** 1140-1144 (2014).

6. Roy S. S., Norcott P., Rayner P. J., Green G. G. R., Duckett S. B., A Simple Route to Strong Carbon-13 NMR Signals Detectable for Several Minutes, *Chem - Eur J,* **23,** 10496-10500 (2017).

7. Fuller J., Carlin R. T., Structural and electrochemical characterization of 1,3-bis-(4-methylphenyl)imidazolium chloride, *J Chem Crystallogr,* **24,** 489-493 (1994).

8. Micksch M., Tenne M., Strassner T., Synthesis of 1,2-Diaryl- and 1-Aryl-2-alkylimidazoles with Sterically Demanding Substituents, *Eur J Org Chem,* **2013,** 6137-6145 (2013).

9. Kinuta H., Tobisu M., Chatani N., Rhodium-Catalyzed Borylation of Aryl 2-Pyridyl Ethers through Cleavage of the Carbon–Oxygen Bond: Borylative Removal of the Directing Group, *J Am Chem Soc,* **137,** 1593-1600 (2015).

10. Heravi M. M., Abdolhosseini N., Oskooie H. A., Regioselective and high-yielding bromination of aromatic compounds using hexamethylenetetramine–bromine, *Tetrahedron Lett,* **46,** 8959-8963 (2005).

11. Kelly III R. A., Clavier H., Giudice S., Scott N. M., Stevens E. D., Bordner J., *et al.*, Determination of N-Heterocyclic Carbene (NHC) Steric and Electronic Parameters using the [(NHC)Ir(CO)2Cl] System, *Organometallics,* **27,** 202-210 (2008).

12. Nelson D. J., Nolan S. P., Quantifying and understanding the electronic properties of N-heterocyclic carbenes, *Chem Soc Rev,* **42,** 6723-6753 (2013).

13. M. J. Frisch G. W. T., H. B. Schlegel, G. E. Scuseria, , M. A. Robb J. R. C., G. Scalmani, V. Barone, B. Mennucci, , G. A. Petersson H. N., M. Caricato, X. Li, H. P. Hratchian, , A. F. Izmaylov J. B., G. Zheng, J. L. Sonnenberg, M. Hada, , M. Ehara K. T., R. Fukuda, J. Hasegawa, M. Ishida, T. Nakajima, , Y. Honda O. K., H. Nakai, T. Vreven, J. A. Montgomery, Jr., , *et al.* Gaussian 09, Revision D.01. 2010.

14. Becke A. D., DENSITY-FUNCTIONAL EXCHANGE-ENERGY APPROXIMATION WITH CORRECT ASYMPTOTIC-BEHAVIOR, *Physical Review A,* **38,** 3098-3100 (1988).

15. Perdew J. P., DENSITY-FUNCTIONAL APPROXIMATION FOR THE CORRELATION-ENERGY OF THE INHOMOGENEOUS ELECTRON-GAS, *Physical Review B,* **33,** 8822-8824 (1986).

16. Perdew J. P., Erratum: Density-functional approximation for the correlation energy of the inhomogeneous electron gas, *Physical Review B,* **34,** 7406-7406 (1986).

17. Schafer A., Horn H., Ahlrichs R., Fully Optimized Contracted Gaussian-Basis Sets For Atoms Li To Kr, *J Chem Phys,* **97,** 2571-2577 (1992).

18. Schafer A., Huber C., Ahlrichs R., Fully optimized contracted Gaussian basis sets of triple zeta valence quality for atoms Li to Kr, *The Journal of Chemical Physics,* **100,** 5829-5835 (1994).

19. Baldes A. [cited 21/02/2018]Available from: http://www.cosmologic-services.de/basis-sets/basissets.php

20. Andrae D., Haussermann U., Dolg M., Stoll H., Preuss H., Energy-Adjusted Abinitio Pseudopotentials For The 2nd And 3rd Row Transition-Elements, *Theoretica Chimica Acta,* **77,** 123-141 (1990).

21. Poater A., Cosenza B., Correa A., Giudice S., Ragone F., Scarano V., *et al.*, SambVca: A Web Application for the Calculation of the Buried Volume of N-Heterocyclic Carbene Ligands, *Eur J Inorg Chem,* **2009,** 1759-1766 (2009).

22. Shchepin R. V., Jaigirdar L., Theis T., Warren W. S., Goodson B. M., Chekmenev E. Y., Spin Relays Enable Efficient Long-Range Heteronuclear Signal Amplification by Reversible Exchange, *J Phys Chem C,* **121,** 28425-28434 (2017).

23. Kessler H., Mronga S., Gemmecker G., Multi-dimensional NMR experiments using selective pulses, *Magn Reson Chem,* **29,** 527-557 (1991).

24. Eshuis N., Aspers R. L. E. G., van Weerdenburg B. J. A., Feiters M. C., Rutjes F. P. J. T., Wijmenga S. S., *et al.,* Determination of long-range scalar (1)H-(1)H coupling constants responsible for polarization transfer in SABRE, *Journal of magnetic resonance (San Diego, Calif : 1997),* **265,** 59-66 (2016).

25. Theis T., Truong M. L., Coffey A. M., Shchepin R. V., Waddell K. W., Shi F., *et al.,* Microtesla SABRE Enables 10% Nitrogen-15 Nuclear Spin Polarization, *J Am Chem Soc,* **137,** 1404-1407 (2015).

26. Appleby K. M., Mewis R. E., Olaru A. M., Green G. G. R., Fairlamb I. J. S., Duckett S. B., Investigating pyridazine and phthalazine exchange in a series of iridium complexes in order to define their role in the catalytic transfer of magnetisation from para-hydrogen, *Chemical Science,* **6,** 3981-3993 (2015).
